# Supplementary material for: Decoding the Avian Missing Gene Mystery: Dot Chromosomes Unmask Extensive Gene Loss and Novel Genetic Instability
Source: Genome Biol Evol. 2026 Feb 17;18(3):evag038. doi: 10.1093/gbe/evag038 (PMC12954441; doi:10.1093/gbe/evag038)
Supplement: evag038_Supplementary_Data [file evag038_supplementary_data.zip › Supplementary_figures.pdf]

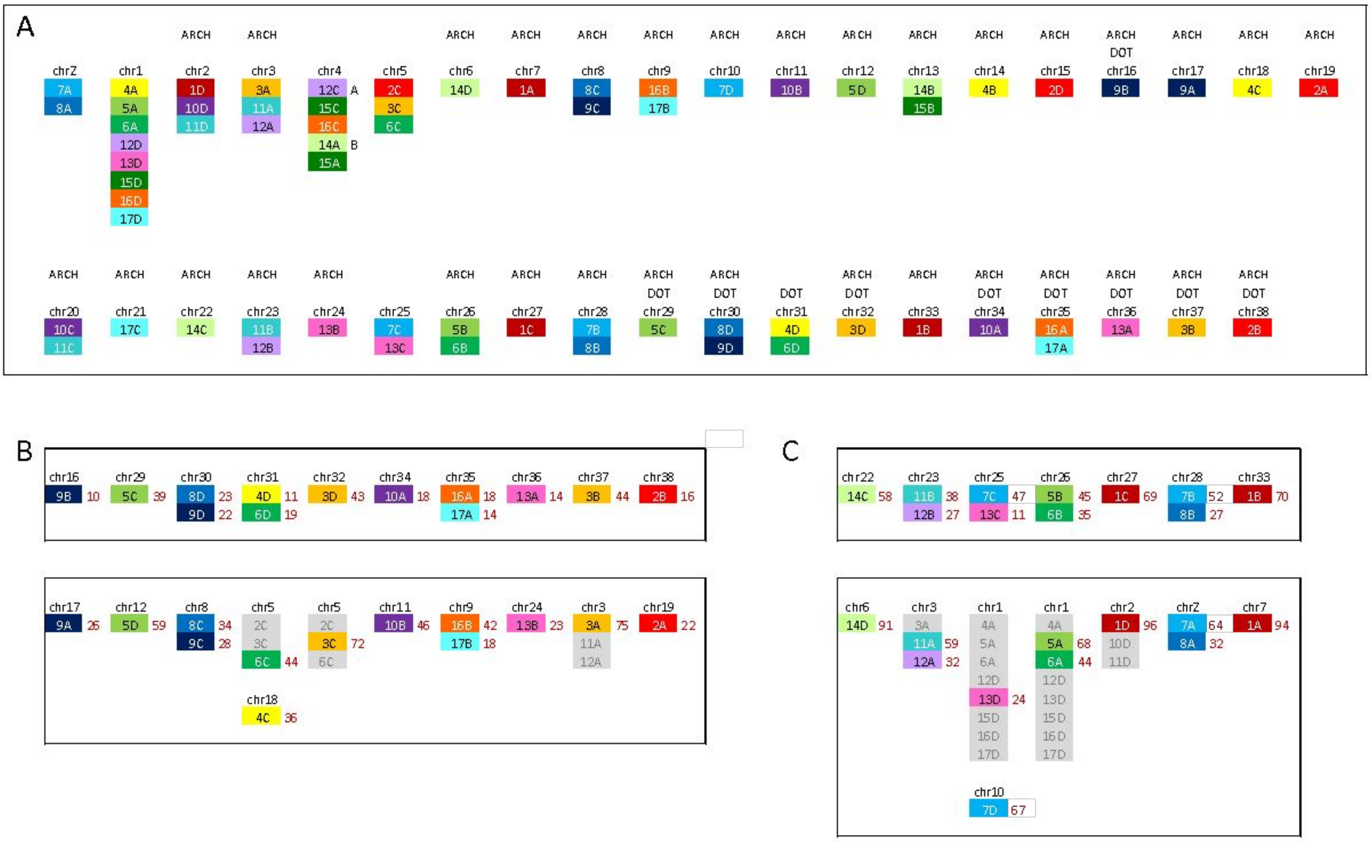

Supplementary Figure 1. Paralogon maps. System of 68 paralogons (17 prevertebrate paralogons, each quadrupled) as defined by Lamb 2021 (1). (A) Paralogon map of chicken genome. Thirty nine chicken chromosomes (2) composed of 38 autosomes and sex chromosome Z (chrW omitted) with corresponding paralogons shown as color-coded rectangles. Dot chromosomes (DOT) and archeochromosomes (ARCH) are denoted above specific chromosomes. Archeochromosomes as defined by Lamb 2021 (1) are chromosomes that did not undergo any chromosome-scale fusion or fission that changed the number of chromosomes in the karyotype from the time of the last common ancestor of bony vertebrates. Chicken chr4 is formed by fusion of two chromosomes (A:12C-15C-16C, B:14A-15A) that were originally separate and as separate chromosomes exist in many avian species (1, 3). Paralogons are arranged on the schematic picture of each chromosome alphanumerically and not by the chromosomal positions of their genes. (B) Paralogon map of 10 chicken dot chromosomes with chromosomes carrying corresponding isoparalogons shown below. On the right side of each rectangle representing a paralogon is a number of ohnologs used in the analysis presented in Fig. 1, Suppl. Tab. 1, and Suppl. Tab. 2. (C) Paralogon map of non-Dot chromosomes smaller than 7Mb with chromosomes carrying corresponding isoparalogons shown below. On the right side of each rectangle representing a paralogon is a number of ohnologs used in the analysis presented in Suppl. Fig. 3.

1. Lamb, Trevor D. 2021. "Analysis of Paralogons, Origin of the Vertebrate Karyotype, and Ancient Chromosomes Retained in Extant Species." Genome Biology and Evolution 13 (4). https://doi.org/10.1093/gbe/evab044.
2. Huang, Zhen, Zaoxu Xu, Hao Bai, Yongji Huang, Na Kang, Xiaoting Ding, Jing Liu, et al. 2023. "Evolutionary Analysis of a Complete Chicken Genome." Proceedings of the National Academy of Sciences of the United States ofAmerica 120 (8): e2216641120.
3. O'Connor, Rebecca E., Rafael Kretschmer, Michael N. Romanov, Darren K. Griffin. 2024. "A Bird's-Eye View of Chromosomic Evolution in the Class Aves." Cells 13 (4): 310.

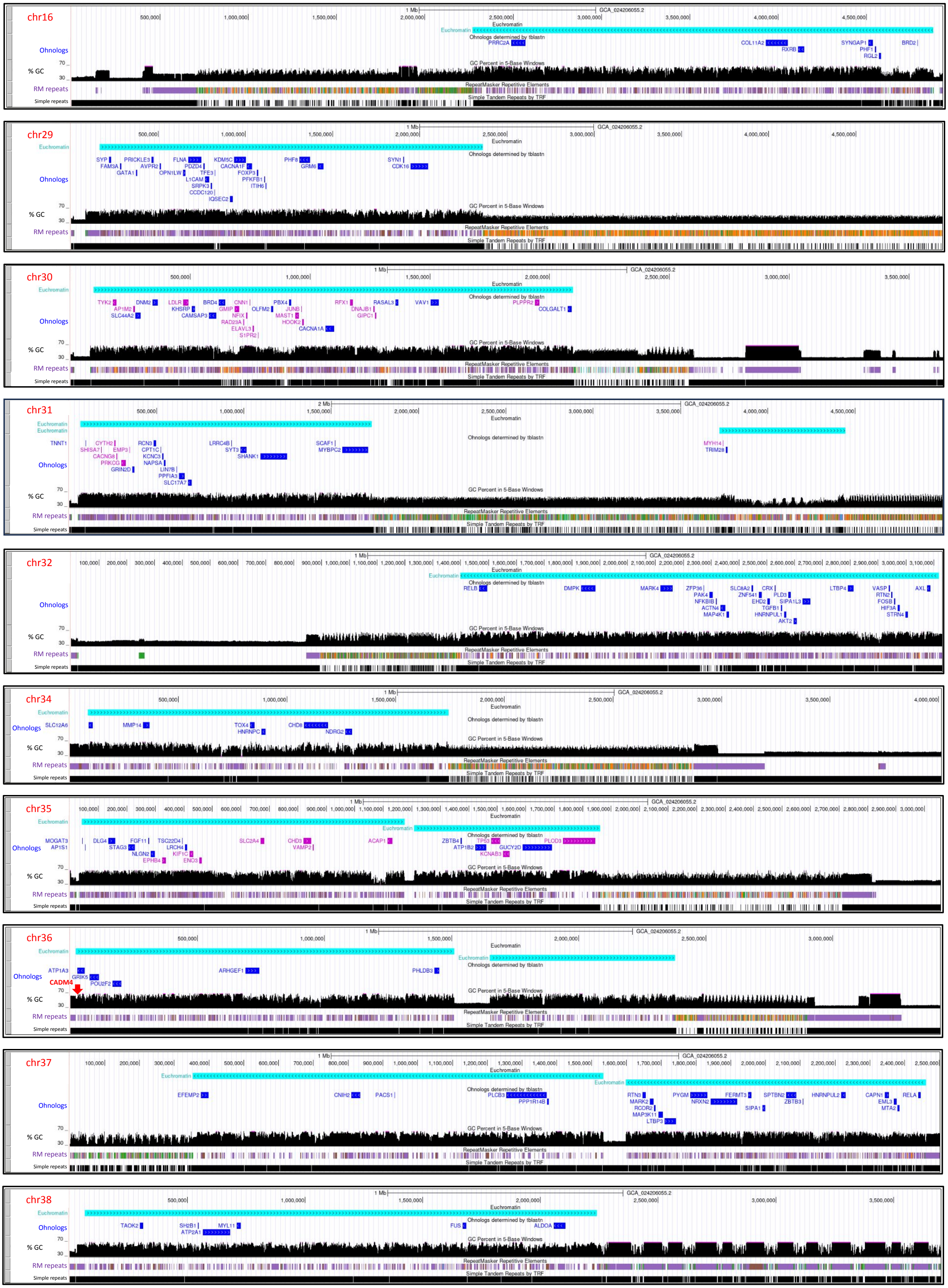

Suppl. Fig. 2 See the legend on the next page.

Supplementary Figure 2. Location of ohnologs found on chicken dot chromosomes relative to positions of euchromatin regions. The figure shows screenshots of chicken huxu dot chromosomes visualized in UCSC genome browser (https://genome.ucsc.edu/). If the dot chromosome was longer than 5 Mb (chr29, chr31), only the first 5Mb of the sequence are shown. Tracks showing % of GC content, repeats detected by Repeat Masker (RM repeats) and repeats detected by Tandem Repeat Finder are internal tracks provided by UCSC genome browser. The BED file of ohnologs shows coding sequences including intervening introns. The positions of these regions were determined by blasting protein sequences of avian orthologs identified by NCBI ortholog database against GGswu genome assembly using tblastn. Regions are shown in blue ink if ohnologs came from the same paralogon. If there are ohnologs from two paralogons on the same chromosome, blue and violet inks are used to differentiate the origin of the ohnolog. A short region of genomic sequence that contains the CADM4 gene is missing from chromosome 36 in the GGswu assembly of huxu genome. The position of missing DNA stretch is indicated by a red arrow. Positions of putative euchromatin are shown by cyan blue rectangles positioned based on the published schema of dot chromosome compartmentalization (1). The positioning was further refined using specific repeat content of dot euchromatin regions (high simple tandem repeats excluding long subtelomere stretches, low content of interspersed repeats).

1. Huang, Zhen, Zaoxu Xu, Hao Bai, Yongji Huang, Na Kang, Xiaoting Ding, Jing Liu, et al. 2023. “Evolutionary Analysis of a Complete Chicken Genome.” Proceedings of the National Academy of Sciences of the United States of America 120 (8): e2216641120.

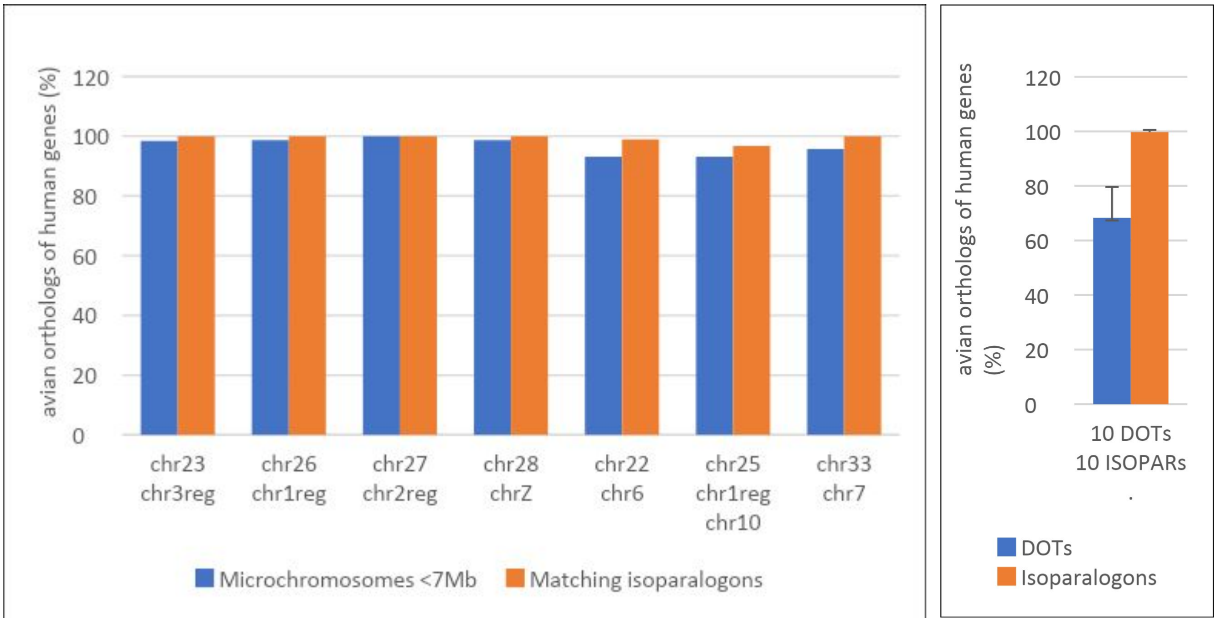

Supplementary Figure 3. Survival of ohnologs in avian paralogons mapping to chicken non-dot chromosomes smaller than 7Mb. Survival in matching isoparalogons on other chromosomes is shown as well. The figure also shows comparison with the average ohnolog survival on 10 dot chromosomes (10 DOTs) and in their matching isoparalogons on other chromosomes (ISOPARs). Error bars represent standard deviation. Designations of individual small non-dot chromosomes are indicated as well as designations of chromosomes with matching isoparalogons shown below. If isoparalogons don‘t cover the entire chromosome, suffix “reg” (region) is added, e.g. chr3reg.

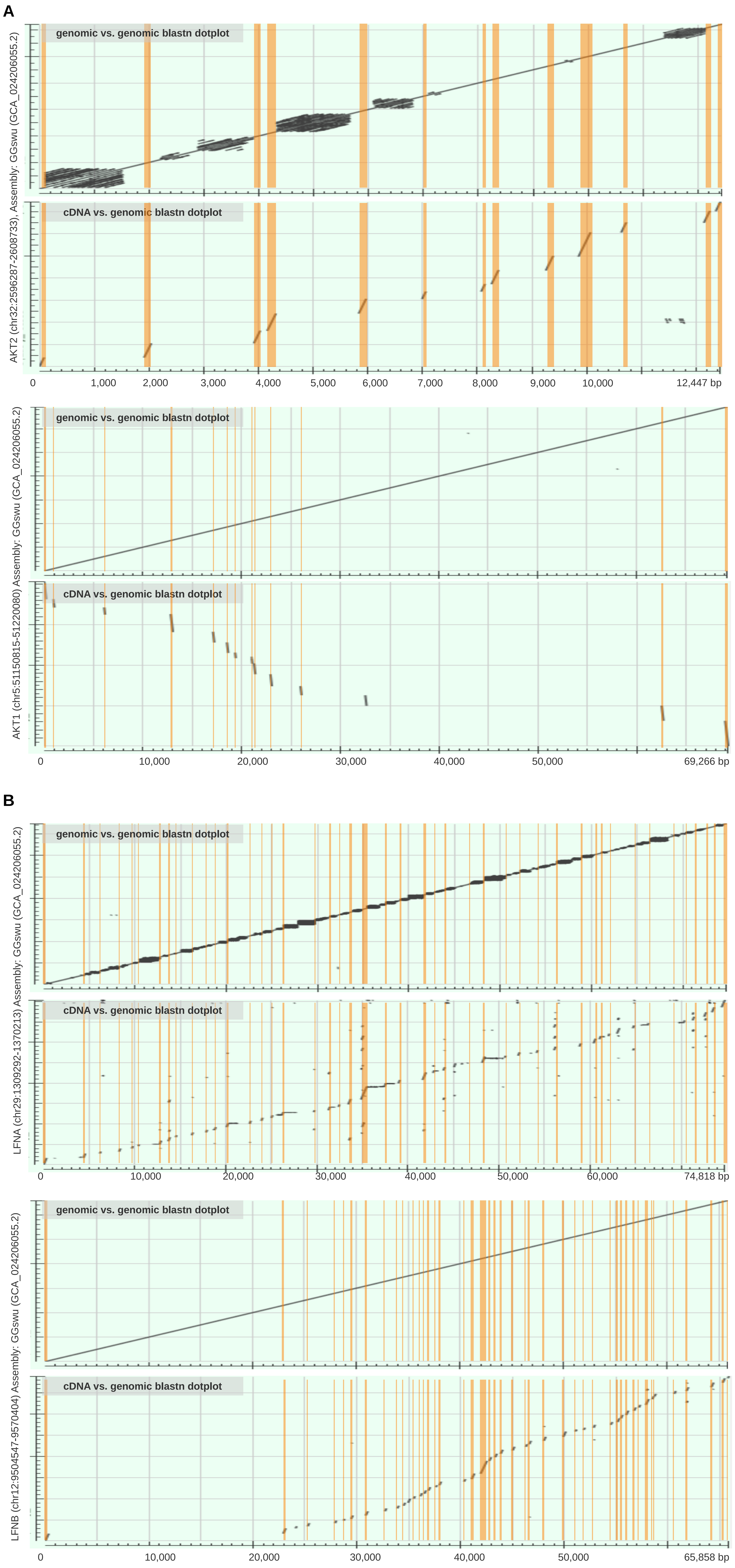

Supplementary Figure 4. Example of local repeat expansion in chicken genes and their ohnologs. (A) Gene AKT2 located on dot chromosome 32 and its ohnolog AKT1 located on chromosome 5. (B) Gene LFNA located on dot chromosome 29 and its ohnolog LFNB located on chromosome 12. (A, B) Sequence dot plots are shown for blastn search of genomic sequence vs. itself (upper schemes), and cDNA vs. genomic sequence (lower schemes). Dark regions in the plots represent blastn hits. Exon positions are manually curated and indicated by an orange background.

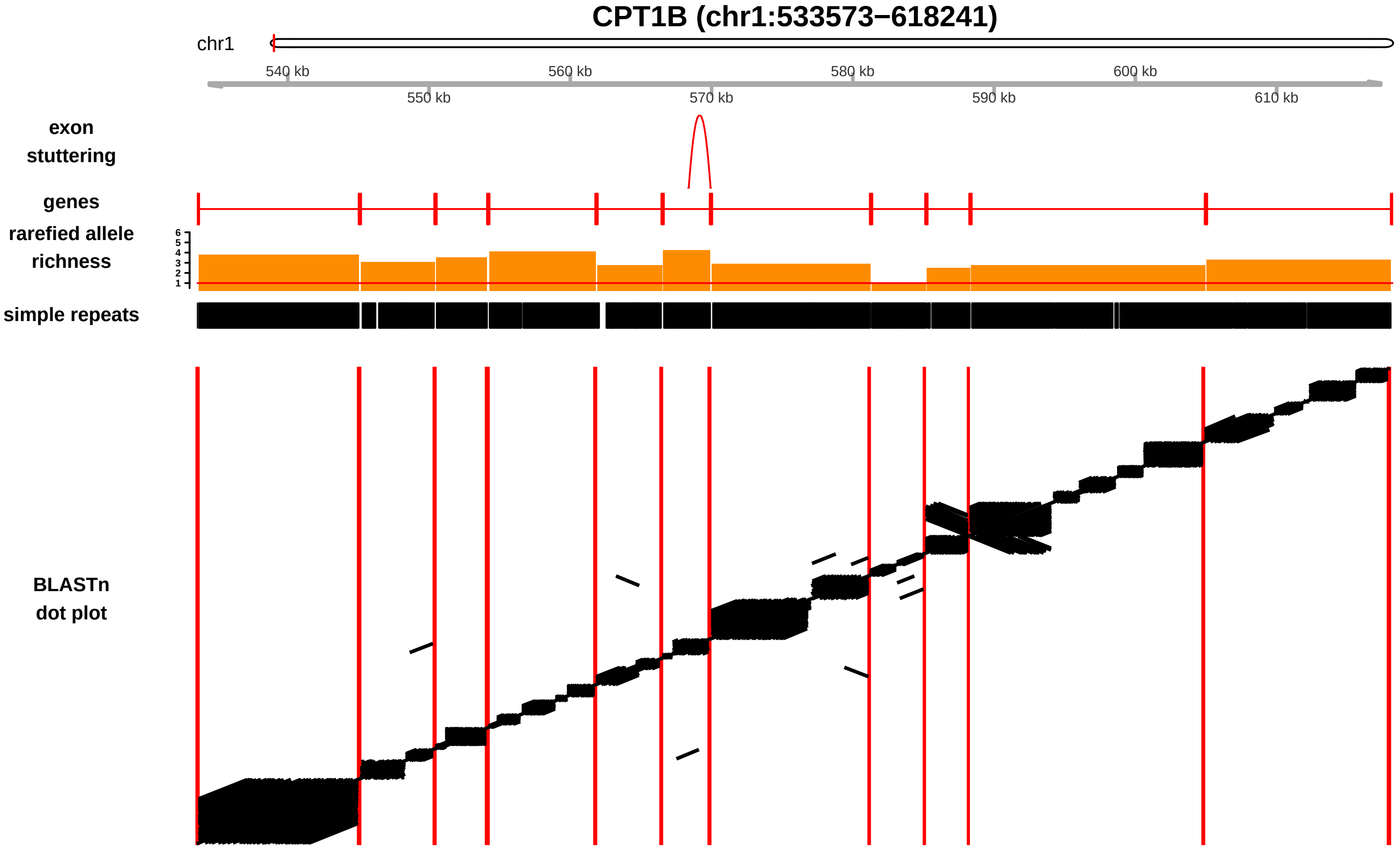
CPT1B (chr1:533573−618241)
chr1
540 kb
560 kb
580 kb
600 kb
550 kb
570 kb
590 kb
610 kb
exon
stuttering
genes
rarefied allele
richness
6
5
4
3
2
1
simple repeats
BLASTn
dot plot

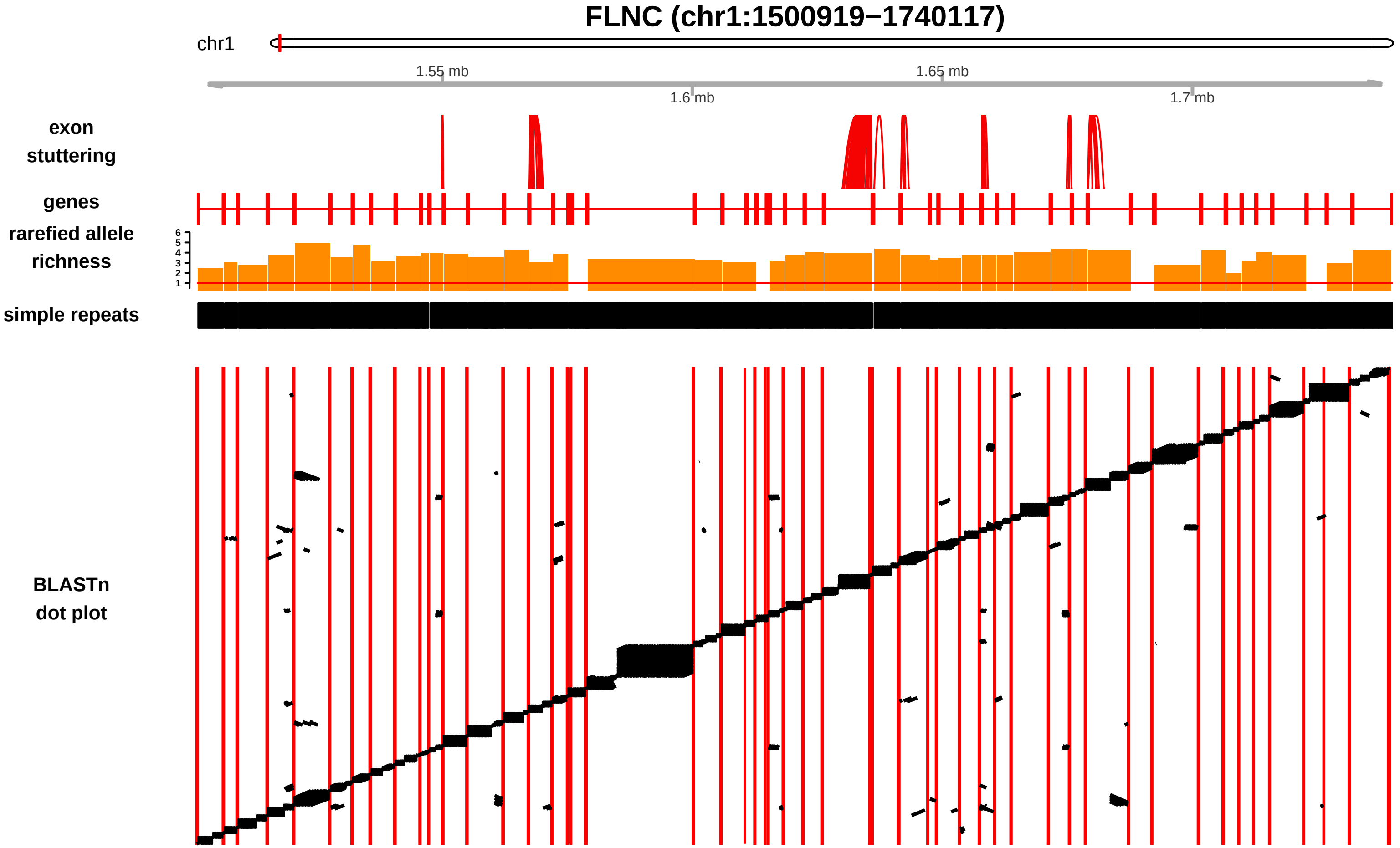
FLNC (chr1:1500919−1740117)
chr1
1.55 mb
1.6 mb
1.65 mb
1.7 mb
exon stuttering
genes
rarefied allele richness
6
5
4
3
2
1
simple repeats
BLASTn dot plot

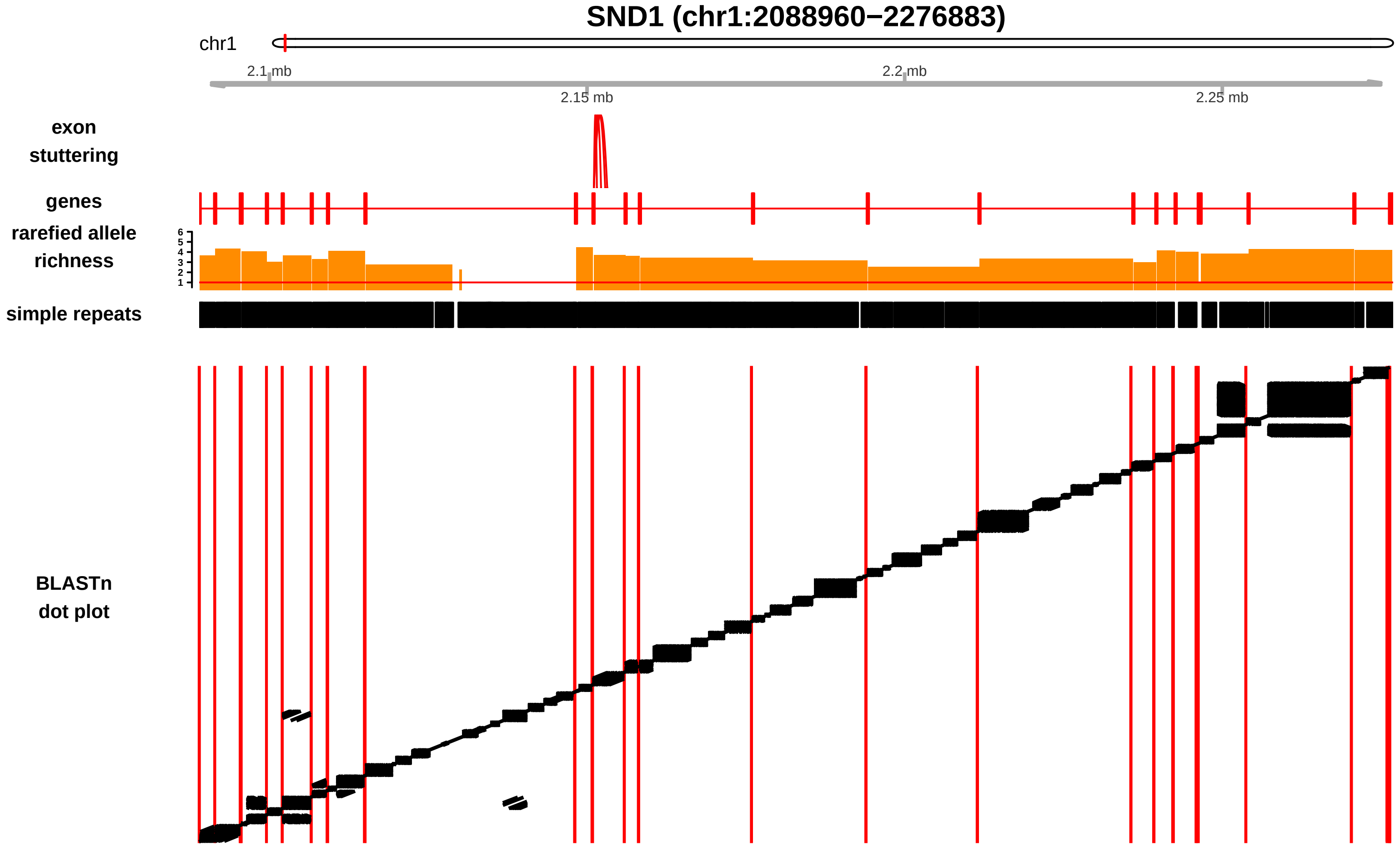

SND1 (chr1:2088960−2276883)
chr1
2.1 mb
2.15 mb
2.2 mb
2.25 mb
exon stuttering
genes
rarefied allele richness
6
5
4
3
2
1
simple repeats
BLASTn dot plot

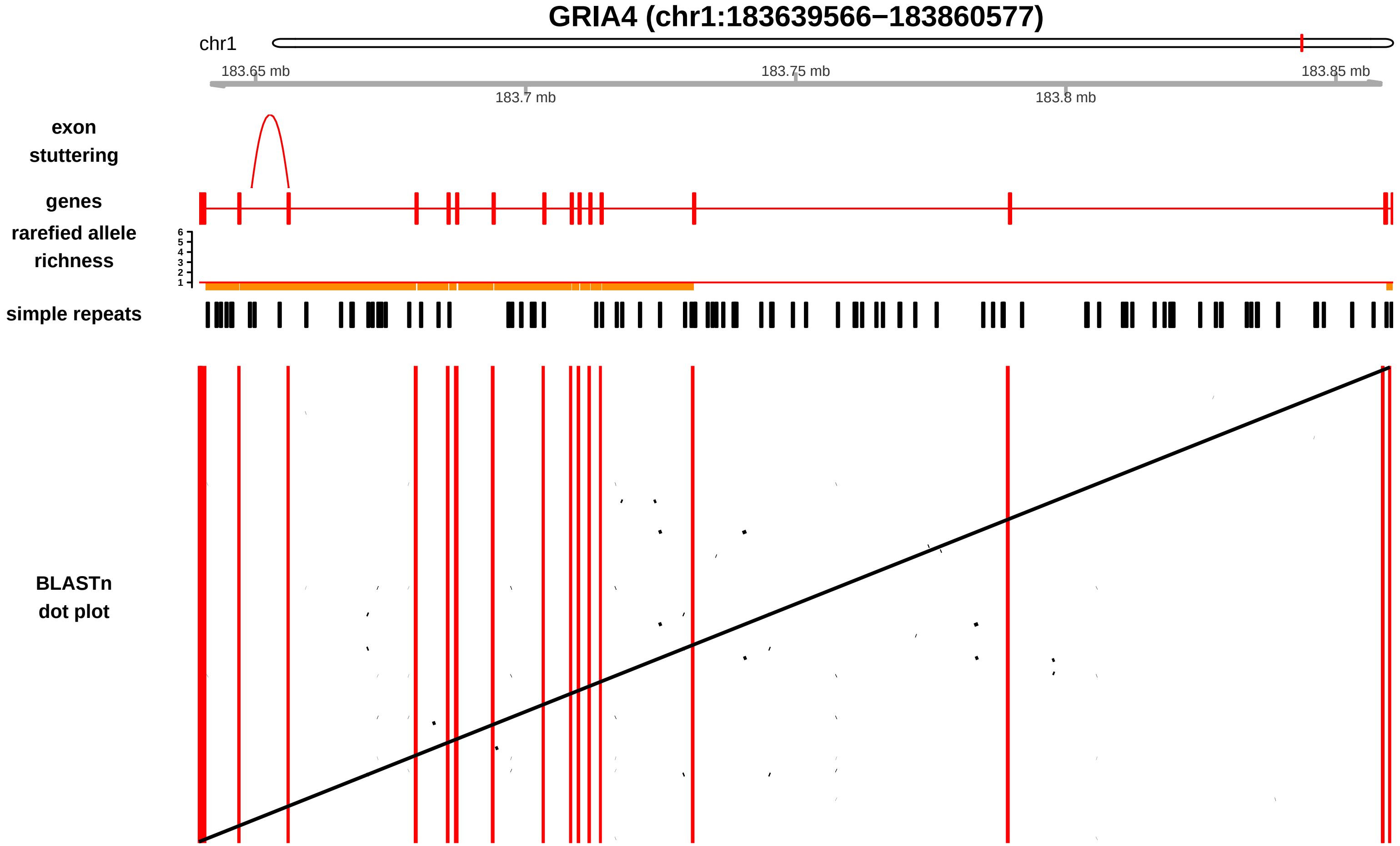

GRIA4 (chr1:183639566−183860577)
chr1
183.65 mb
183.75 mb
183.85 mb
183.7 mb
183.8 mb
exon
stuttering
genes
rarefied allele
richness
6
5
4
3
2
1
simple repeats
BLASTn
dot plot

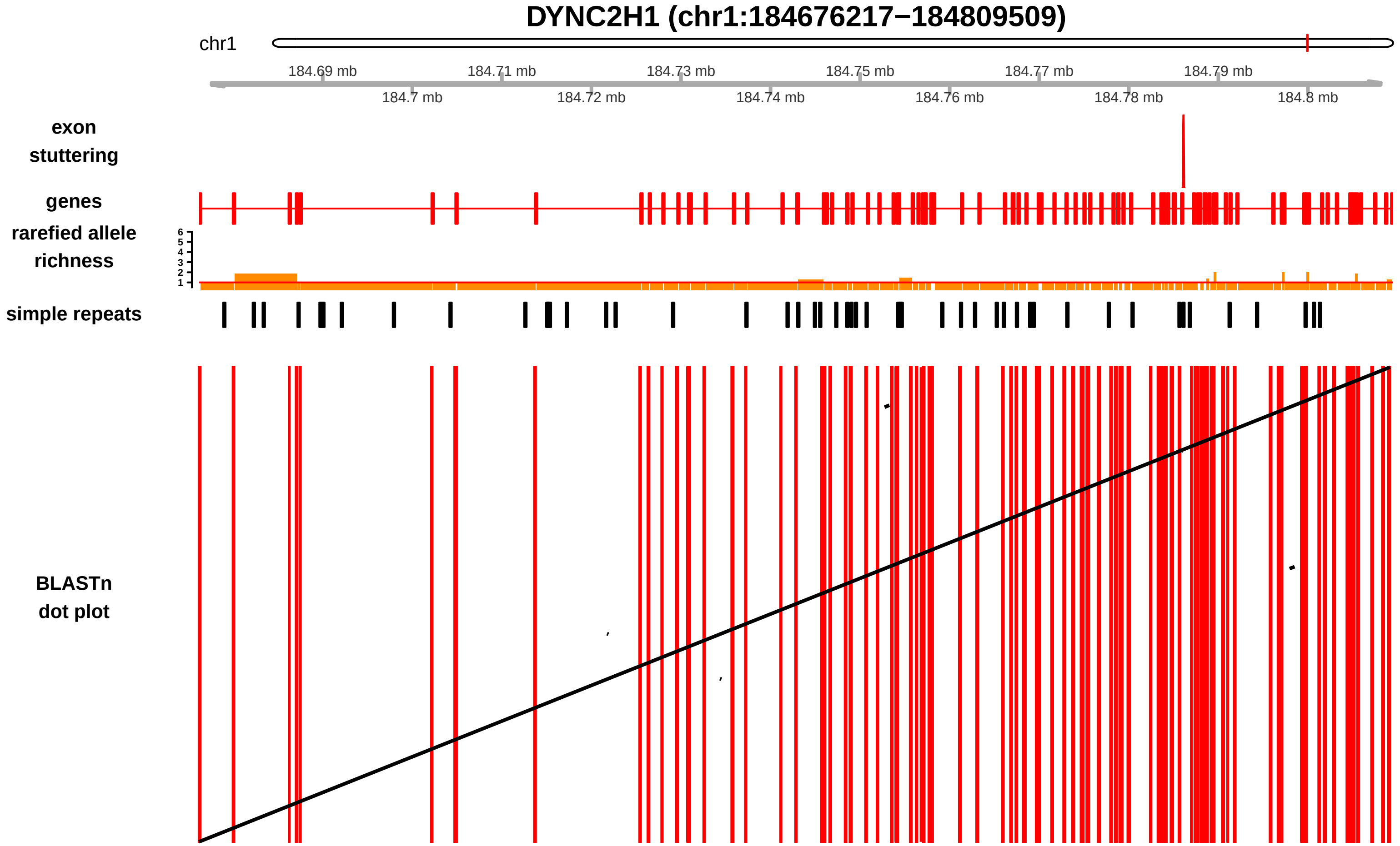

DYNC2H1 (chr1:184676217−184809509)
chr1
184.69 mb
184.71 mb
184.73 mb
184.75 mb
184.77 mb
184.79 mb
184.7 mb
184.72 mb
184.74 mb
184.76 mb
184.78 mb
184.8 mb
exon stuttering
genes
rarefied allele richness
6
5
4
3
2
1
simple repeats
BLASTn dot plot

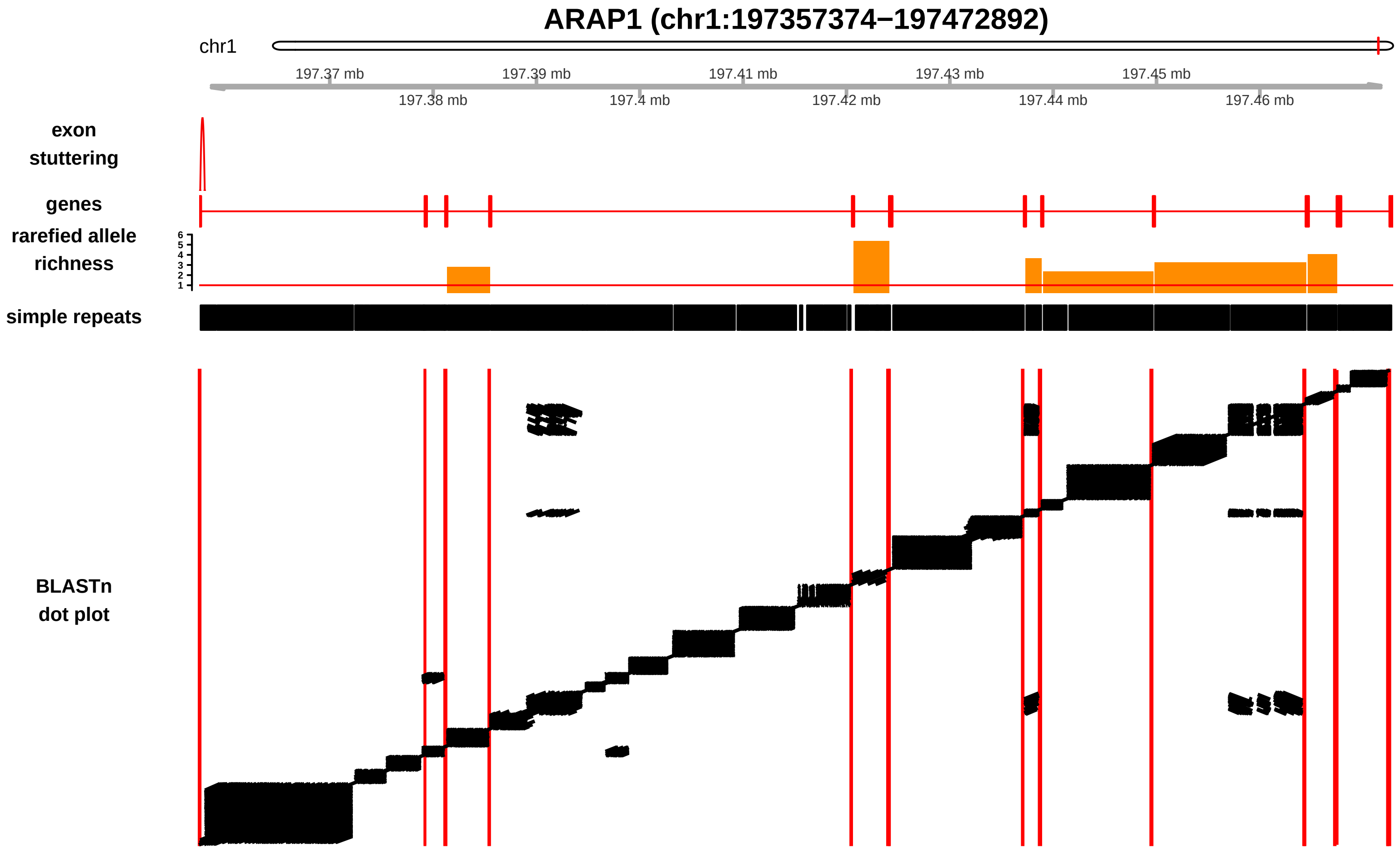
ARAP1 (chr1:197357374−197472892)
chr1
197.37 mb
197.39 mb
197.41 mb
197.43 mb
197.45 mb
197.38 mb
197.4 mb
197.42 mb
197.44 mb
197.46 mb
exon stuttering
genes
rarefied allele richness
6
5
4
3
2
1
simple repeats
BLASTn dot plot

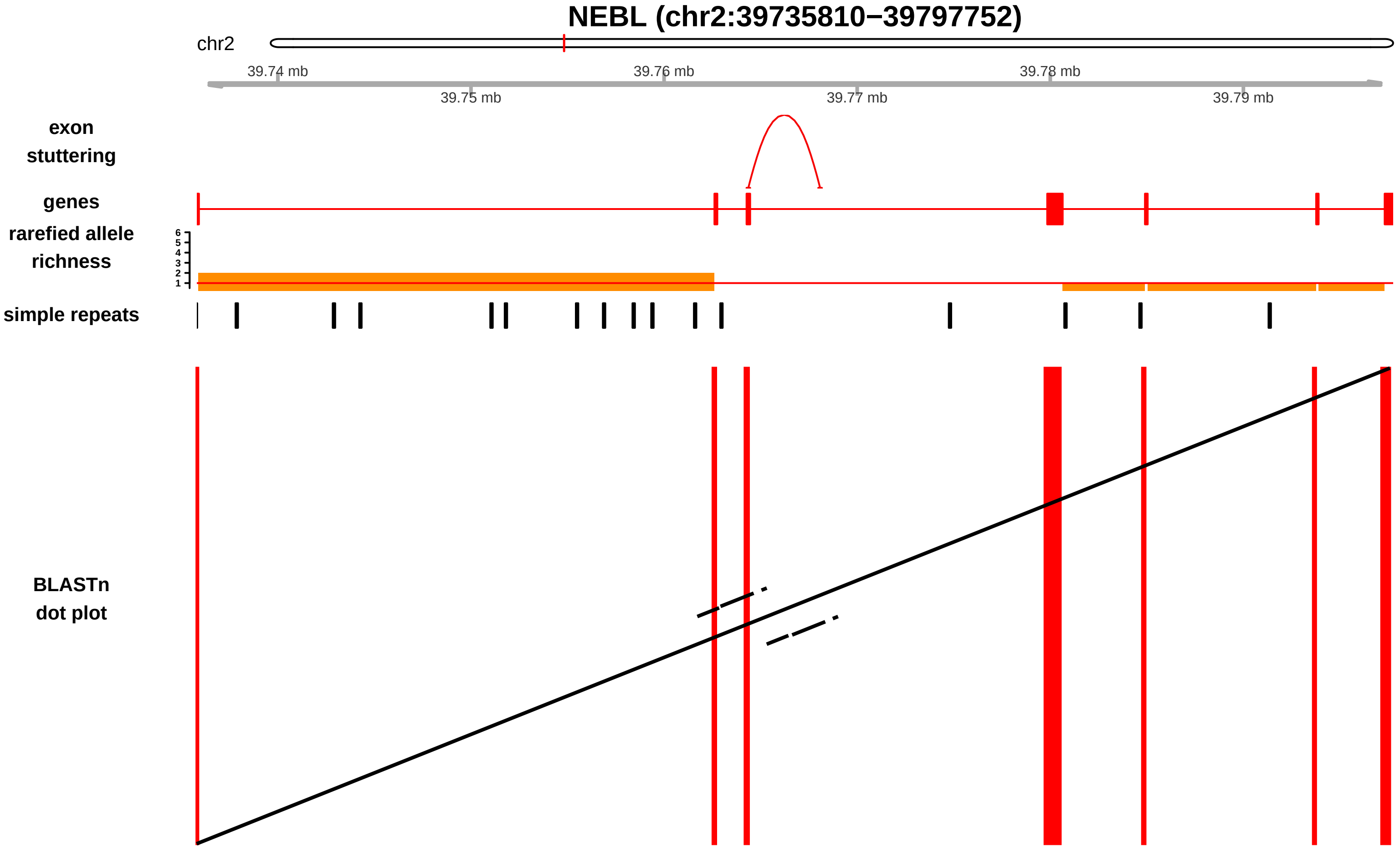

NEBL (chr2:39735810−39797752)
chr2
39.74 mb
39.75 mb
39.76 mb
39.77 mb
39.78 mb
39.79 mb
exon stuttering
genes
rarefied allele richness
6
5
4
3
2
1
simple repeats
BLASTn dot plot

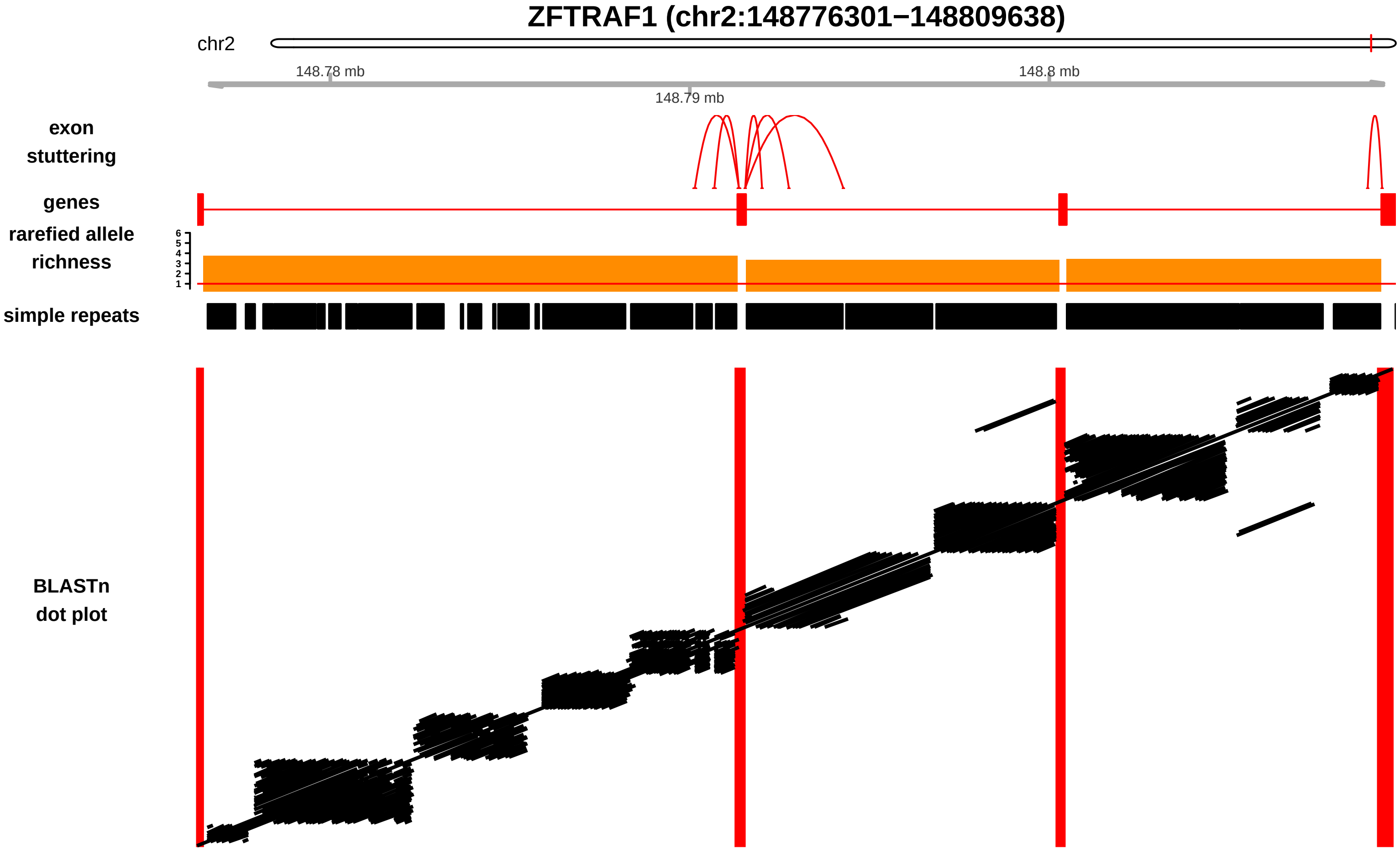

ZFTRAF1 (chr2:148776301−148809638)
chr2
148.78 mb
148.79 mb
148.8 mb
exon stuttering
genes
rarefied allele richness
1
2
3
4
5
6
simple repeats
BLASTn dot plot

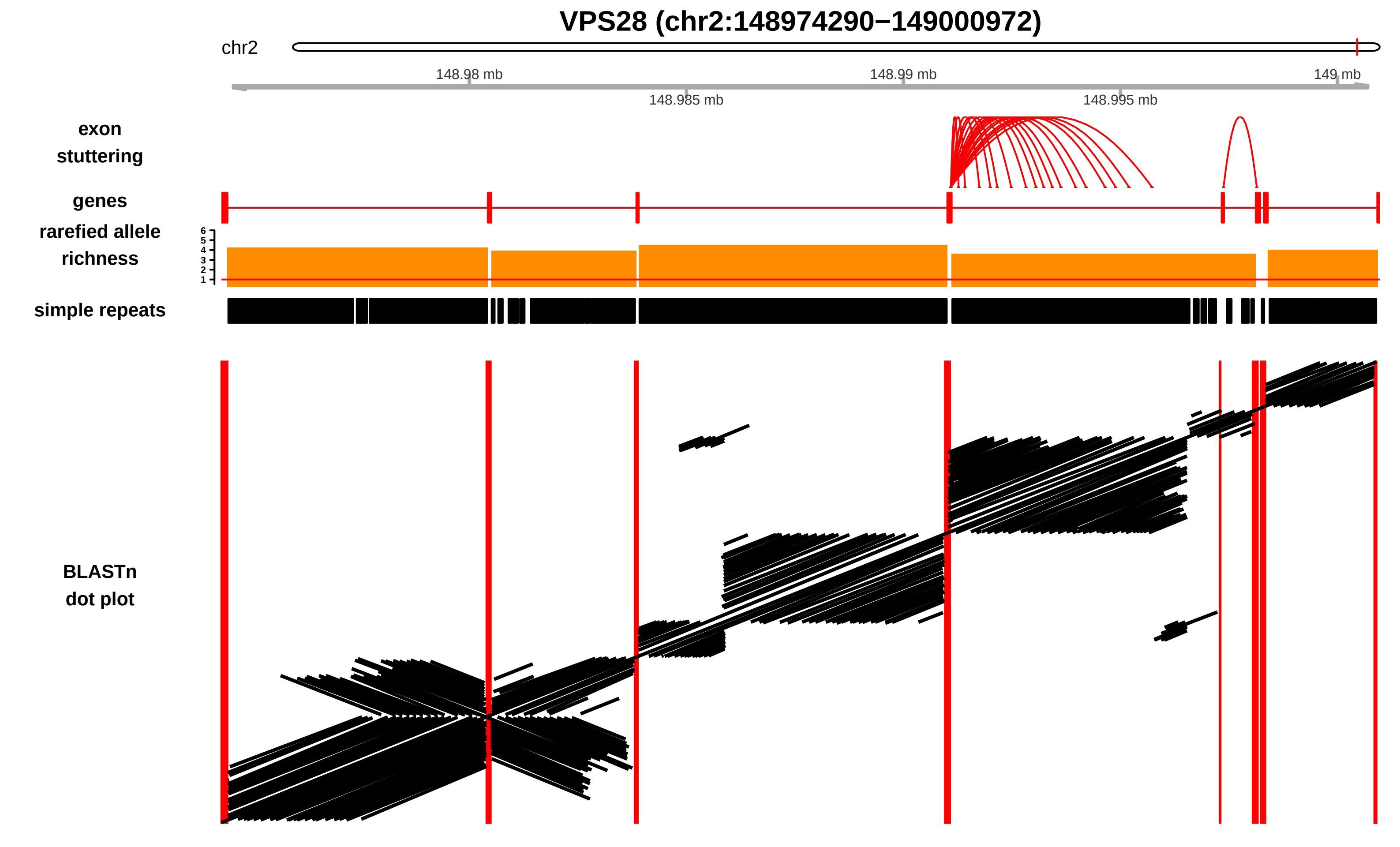

VPS28 (chr2:148974290−149000972)
chr2
148.98 mb
148.99 mb
149 mb
148.985 mb
148.995 mb
exon stuttering
genes
rarefied allele richness
6
5
4
3
2
1
simple repeats
BLASTn dot plot

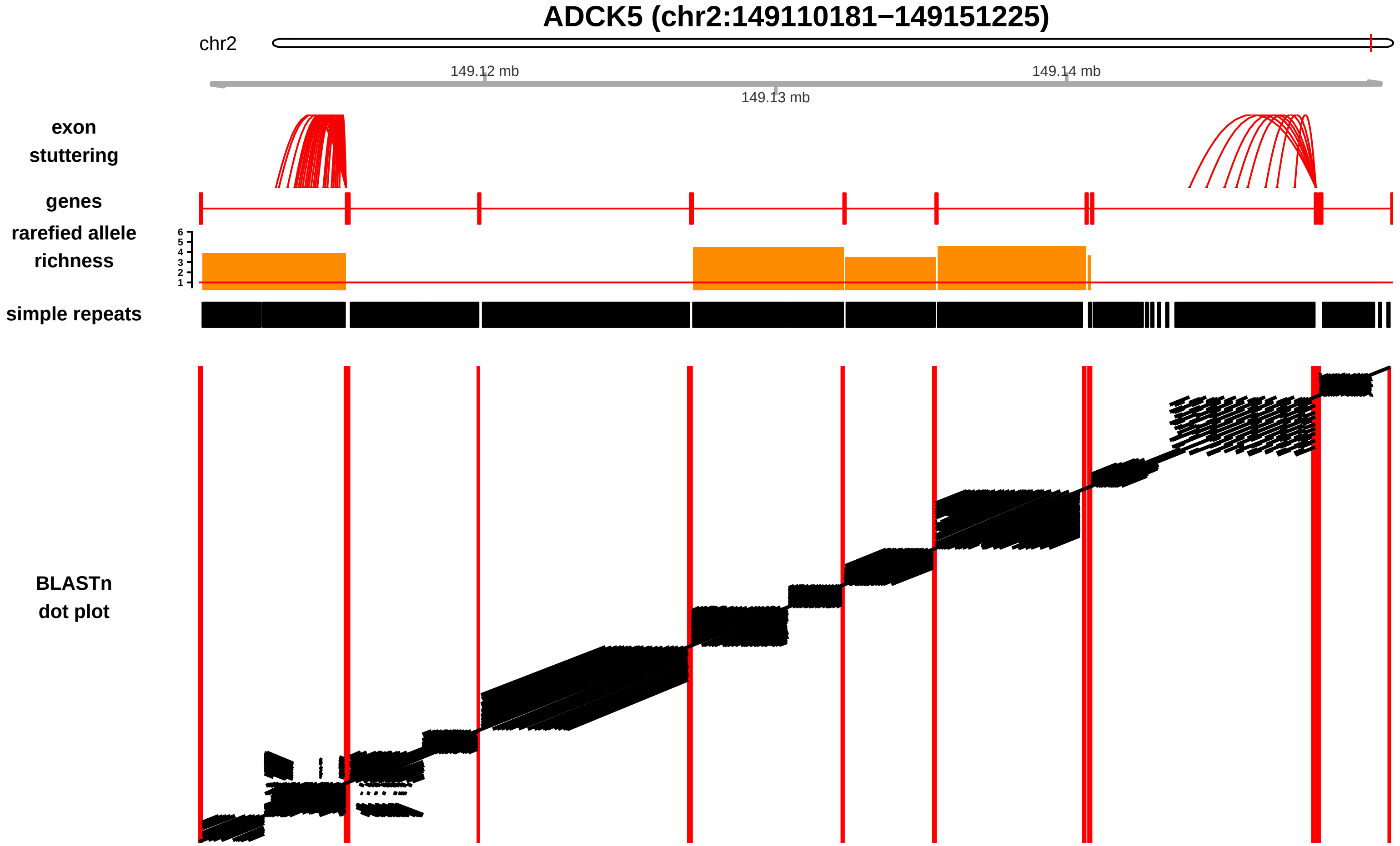

ADCK5 (chr2:149110181−149151225)
chr2
149.12 mb
149.13 mb
149.14 mb
exon stuttering
genes
rarefied allele richness
6
5
4
3
2
1
simple repeats
BLASTn dot plot

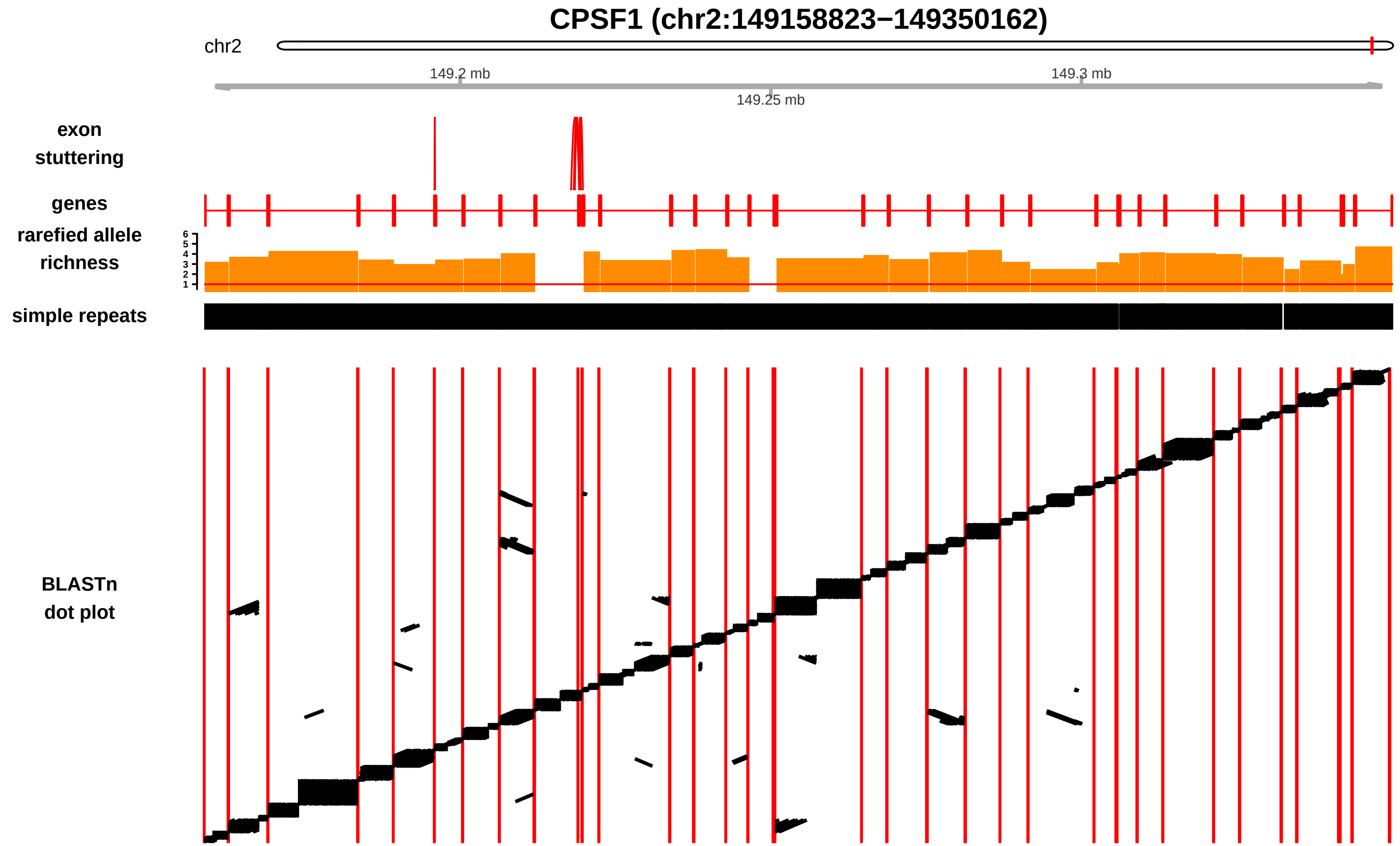

CPSF1 (chr2:149158823−149350162)
chr2
149.2 mb
149.25 mb
149.3 mb
exon
stuttering
genes
rarefied allele
richness
6
5
4
3
2
1
simple repeats
BLASTn
dot plot

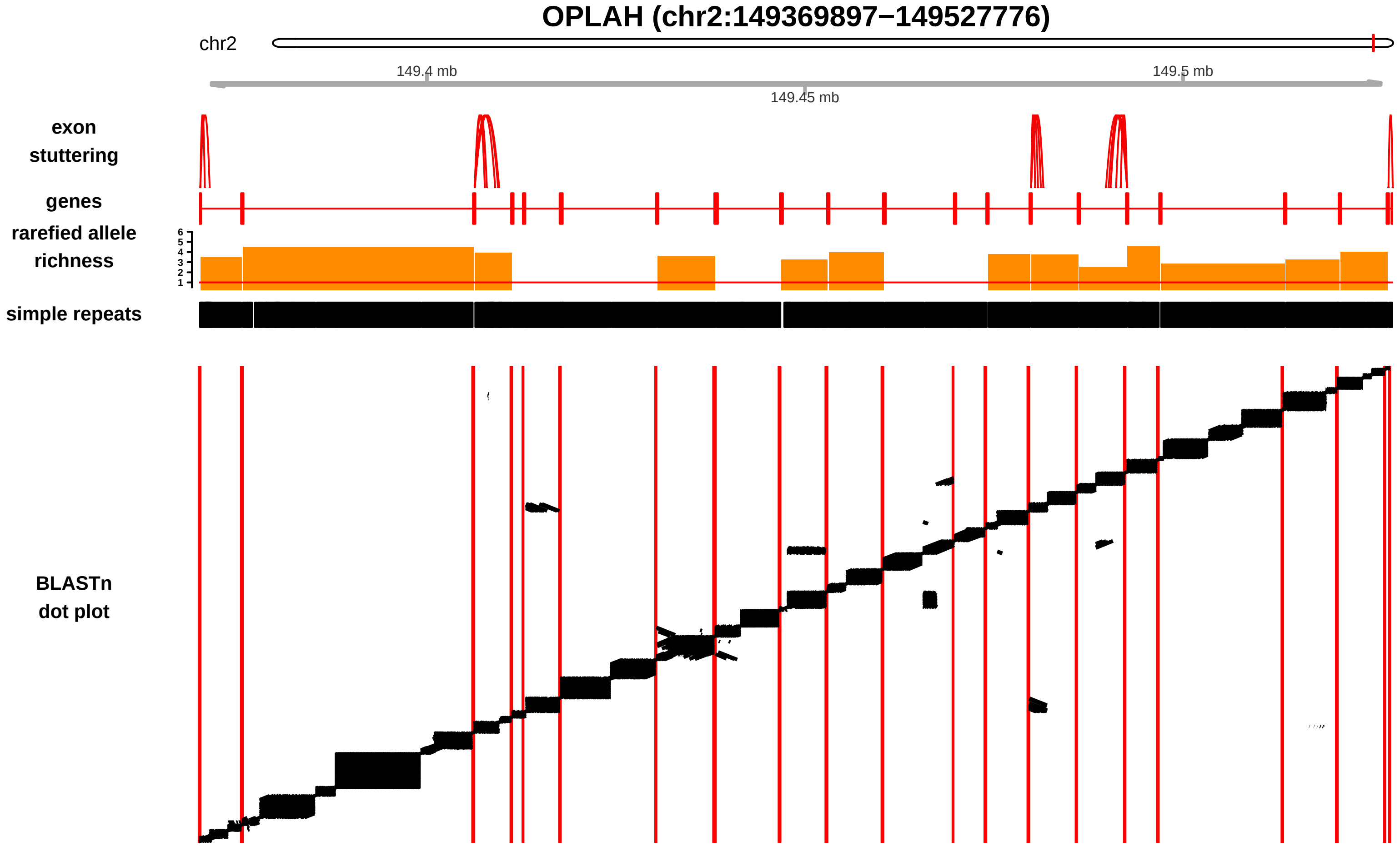
OPLAH (chr2:149369897−149527776)
chr2
149.4 mb
149.45 mb
149.5 mb
exon stuttering
genes
rarefied allele richness
6
5
4
3
2
1
simple repeats
BLASTn dot plot

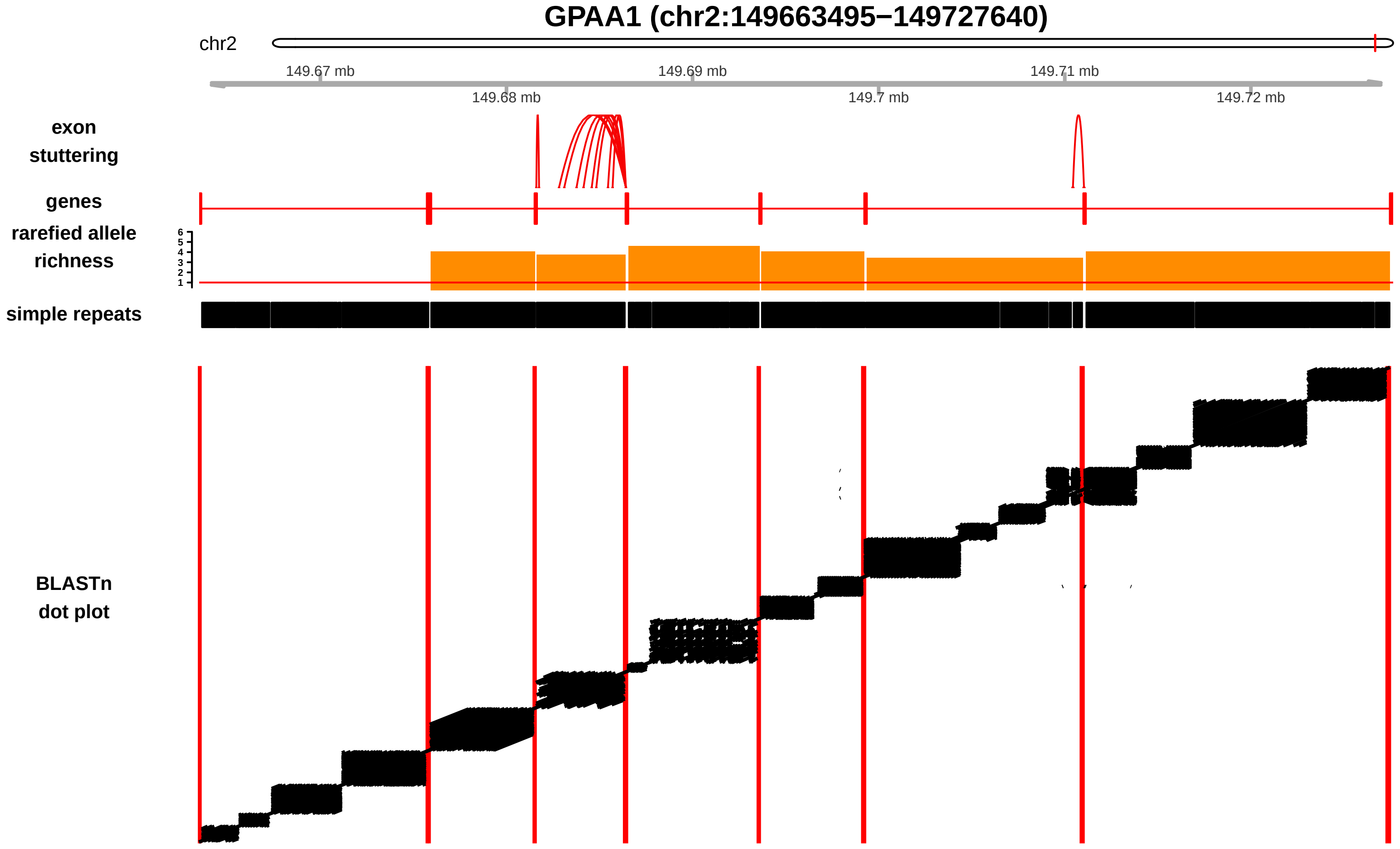
GPAA1 (chr2:149663495−149727640)
chr2
149.67 mb
149.69 mb
149.71 mb
149.68 mb
149.7 mb
149.72 mb
exon stuttering
genes
rarefied allele richness
6
5
4
3
2
1
simple repeats
BLASTn dot plot

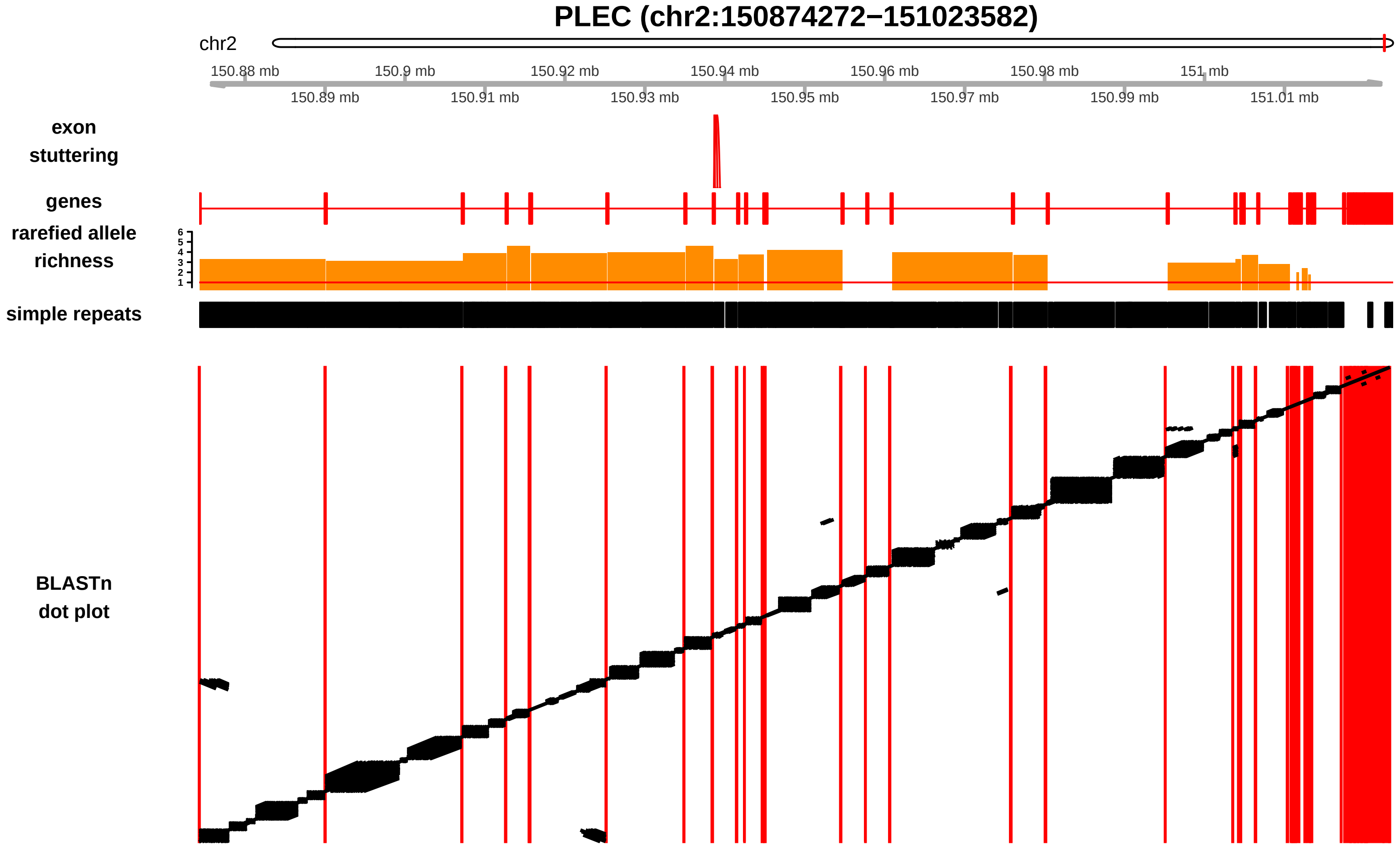
PLEC (chr2:150874272−151023582)
chr2
150.88 mb
150.9 mb
150.92 mb
150.94 mb
150.96 mb
150.98 mb
151 mb
150.89 mb
150.91 mb
150.93 mb
150.95 mb
150.97 mb
150.99 mb
151.01 mb
exon stuttering
genes
rarefied allele richness
6
5
4
3
2
1
simple repeats
BLASTn dot plot

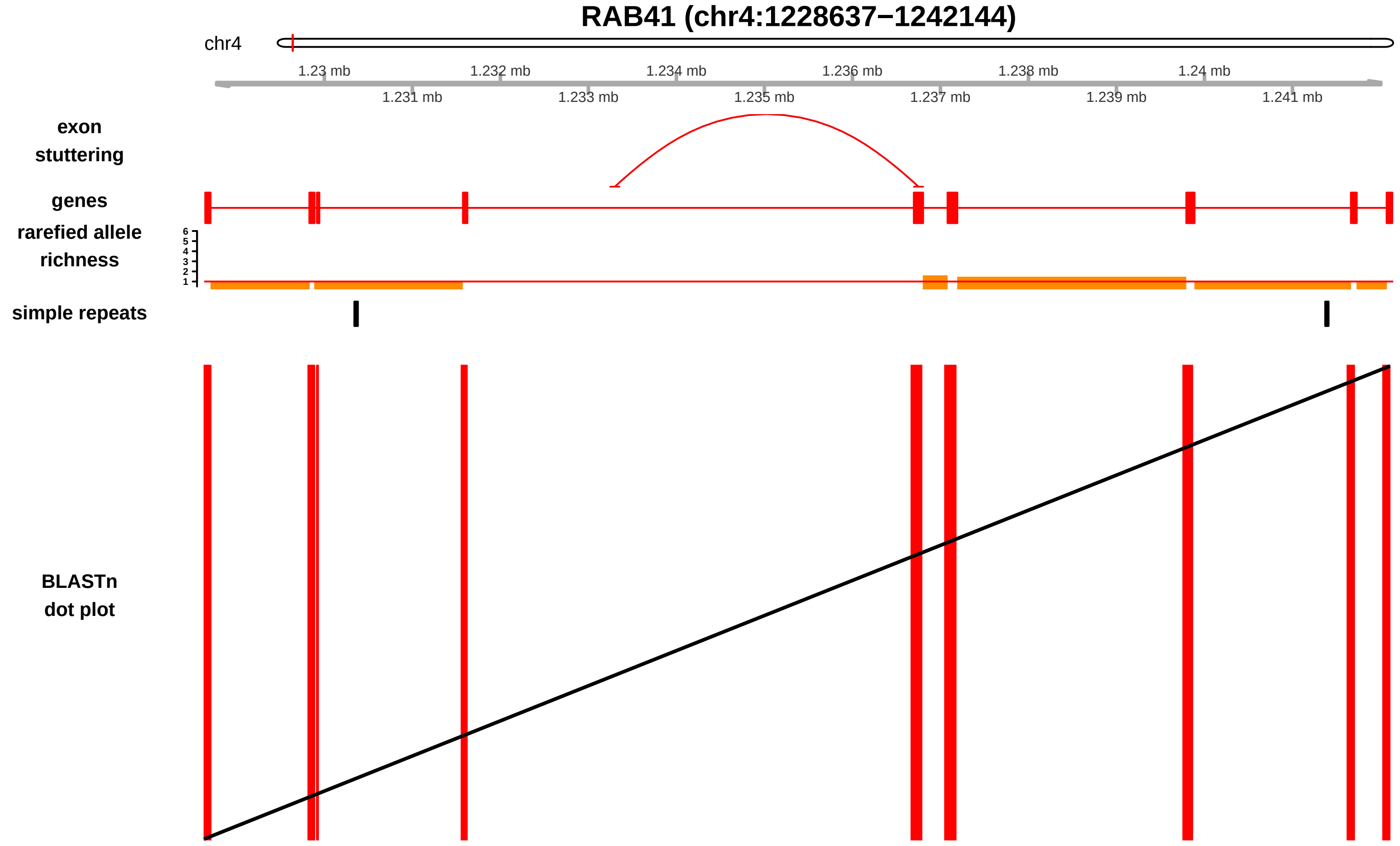

RAB41 (chr4:1228637−1242144)
chr4
1.23 mb
1.231 mb
1.232 mb
1.233 mb
1.234 mb
1.235 mb
1.236 mb
1.237 mb
1.238 mb
1.239 mb
1.24 mb
1.241 mb
exon stuttering
genes
rarefied allele richness
6
5
4
3
2
1
simple repeats
BLASTn dot plot

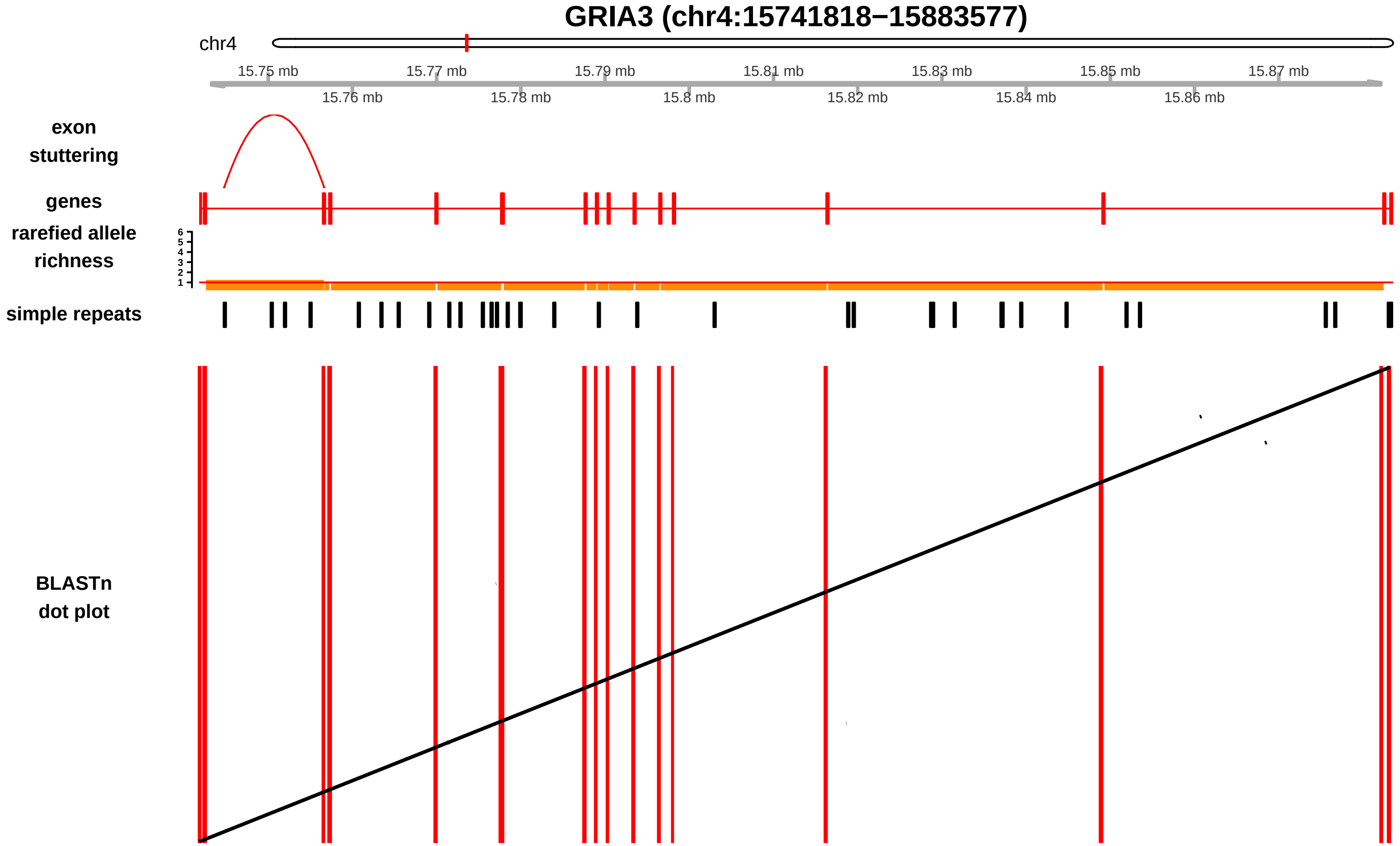

GRIA3 (chr4:15741818−15883577)
chr4
15.75 mb
15.76 mb
15.77 mb
15.78 mb
15.79 mb
15.8 mb
15.81 mb
15.82 mb
15.83 mb
15.84 mb
15.85 mb
15.86 mb
15.87 mb
exon stuttering
genes
rarefied allele richness
1
2
3
4
5
6
simple repeats
BLASTn dot plot

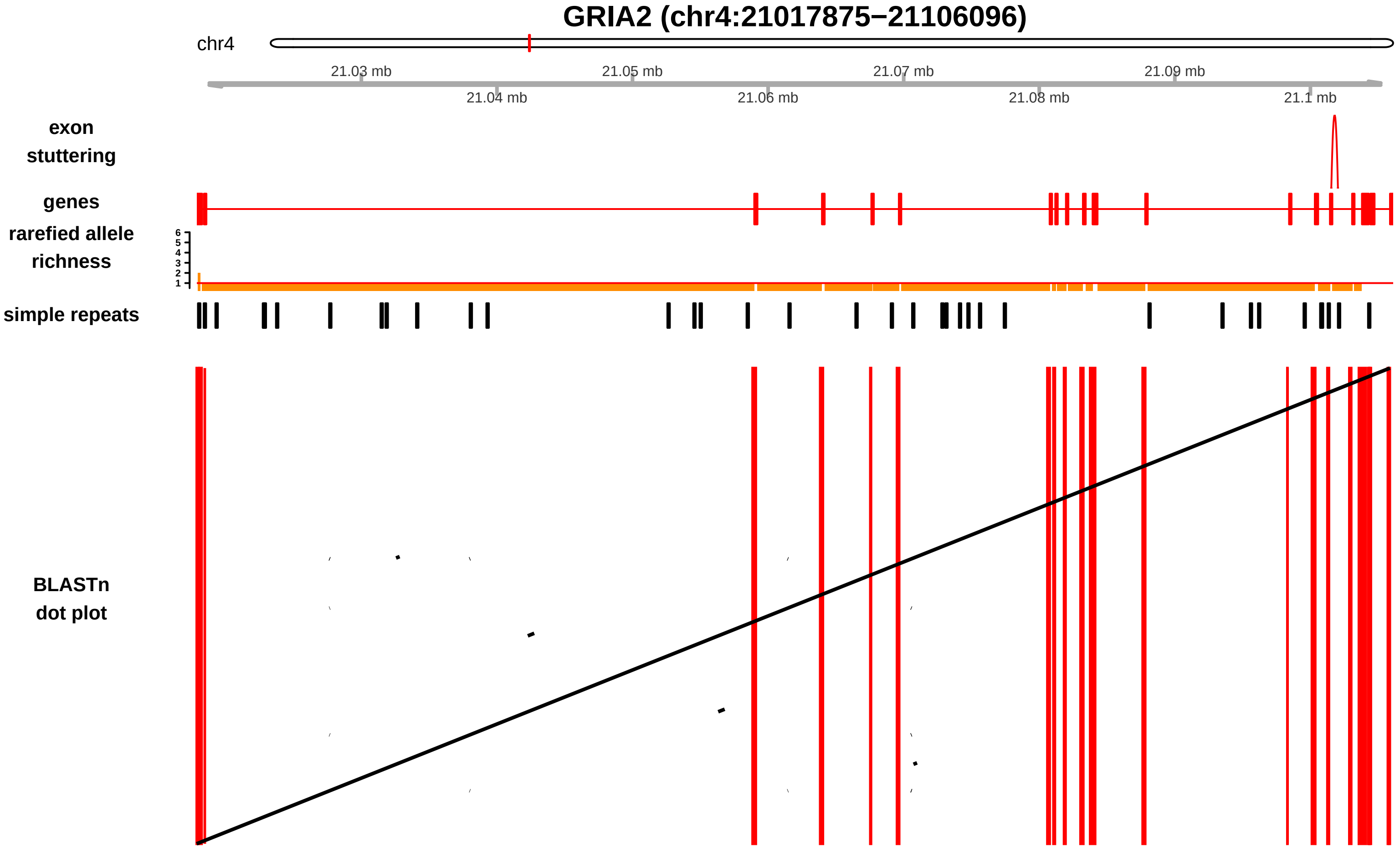
GRIA2 (chr4:21017875−21106096)
chr4
21.03 mb
21.05 mb
21.07 mb
21.09 mb
21.04 mb
21.06 mb
21.08 mb
21.1 mb
exon
stuttering
genes
rarefied allele
richness
6
5
4
3
2
1
simple repeats
BLASTn
dot plot

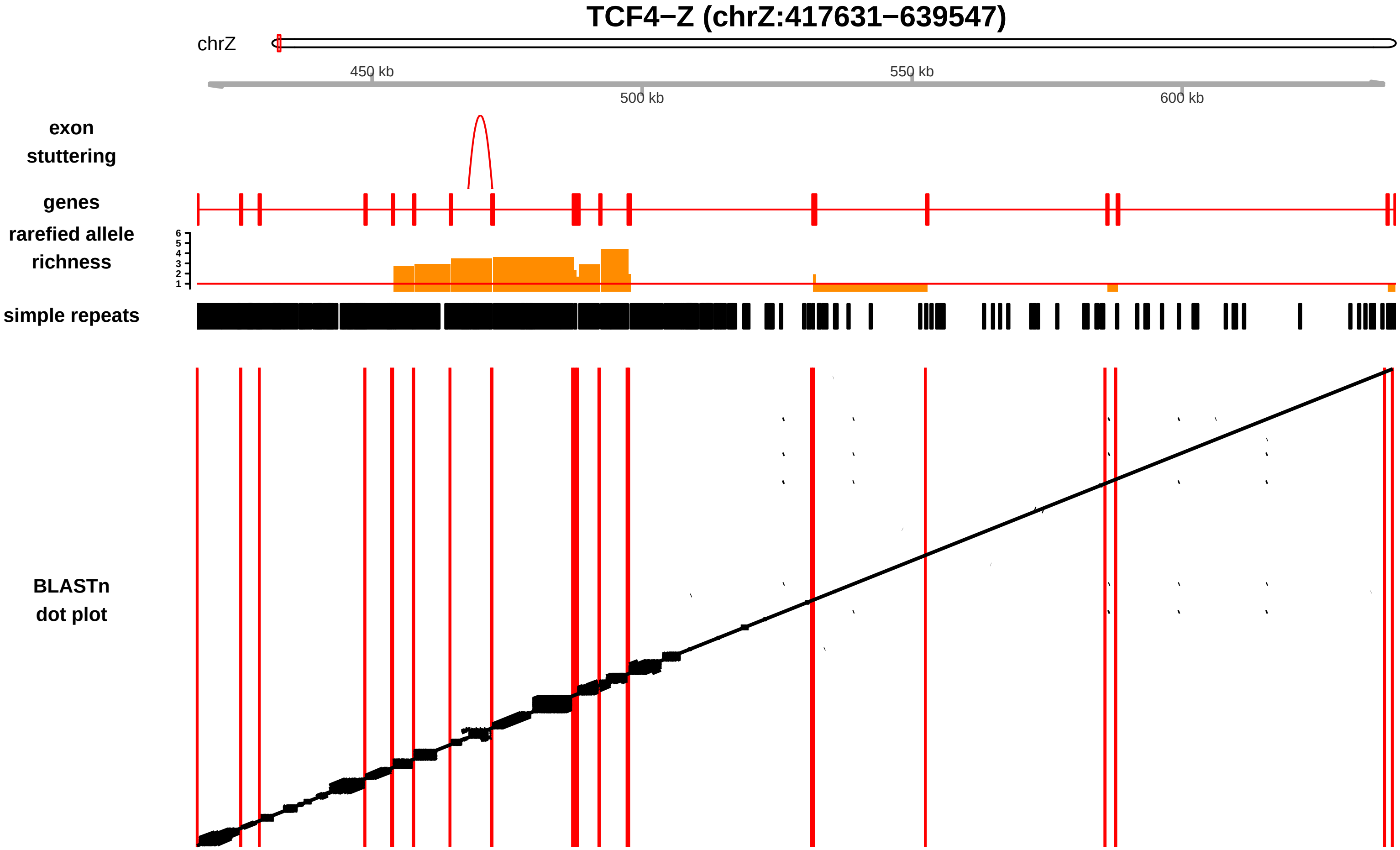

TCF4−Z (chrZ:417631−639547)
chrZ
450 kb
500 kb
550 kb
600 kb
exon stuttering
genes
rarefied allele richness
6
5
4
3
2
1
simple repeats
BLASTn dot plot

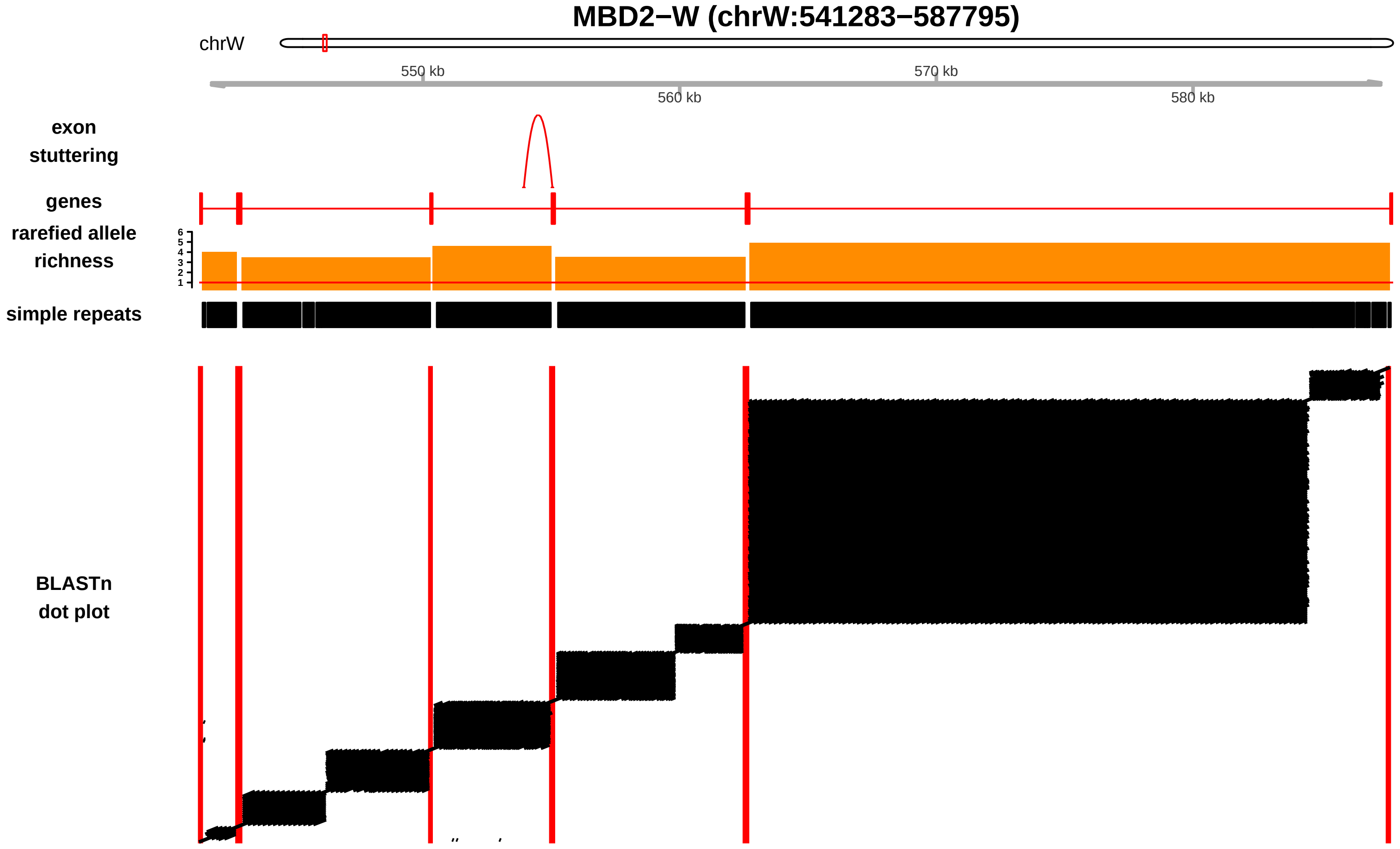
MBD2−W (chrW:541283−587795)
chrW
550 kb
560 kb
570 kb
580 kb
exon stuttering
genes
rarefied allele richness
1
2
3
4
5
6
simple repeats
BLASTn dot plot

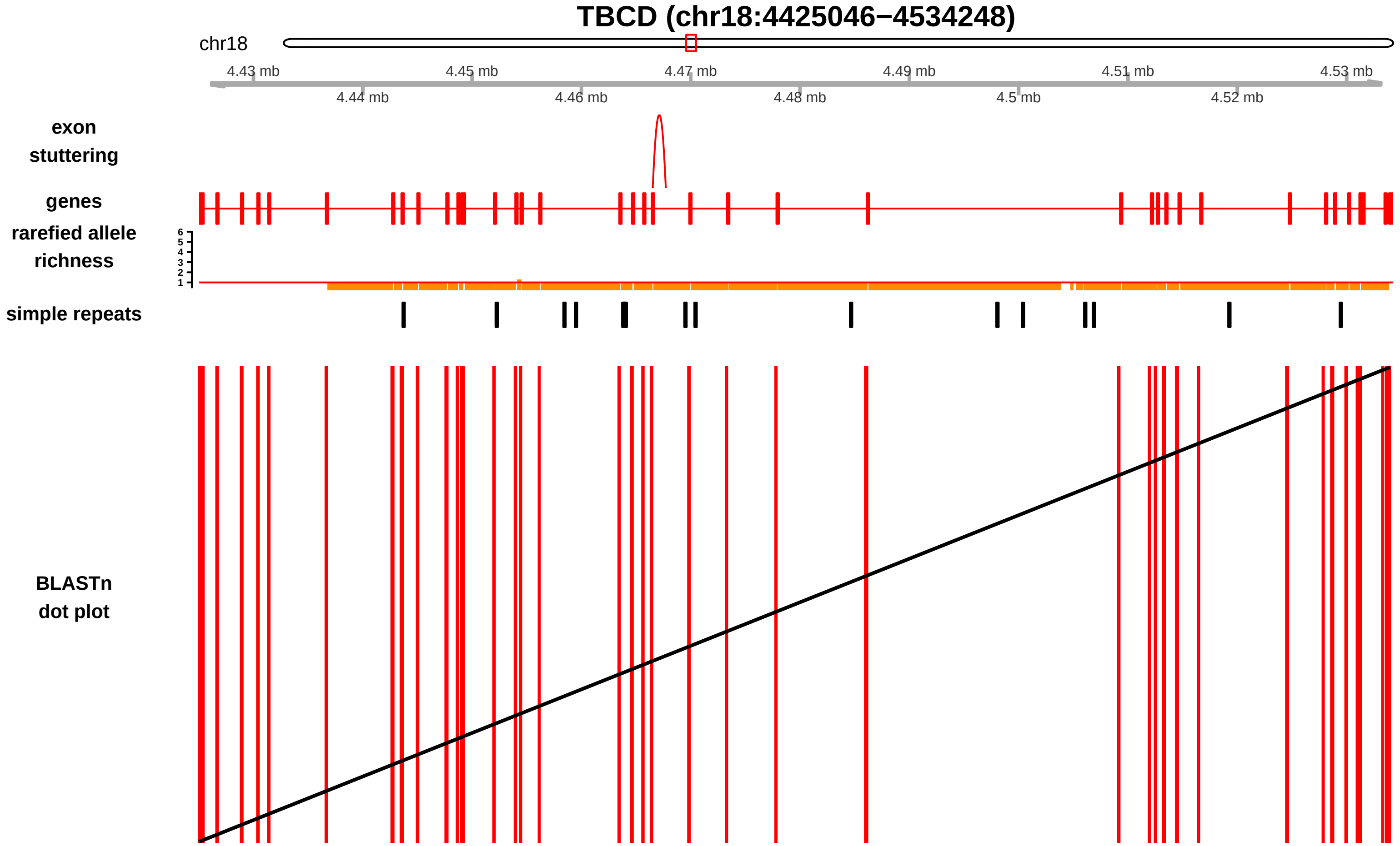

TBCD (chr18:4425046−4534248)
chr18
4.43 mb
4.44 mb
4.45 mb
4.46 mb
4.47 mb
4.48 mb
4.49 mb
4.5 mb
4.51 mb
4.52 mb
4.53 mb
exon stuttering
genes
rarefied allele richness
1
2
3
4
5
6
simple repeats
BLASTn dot plot

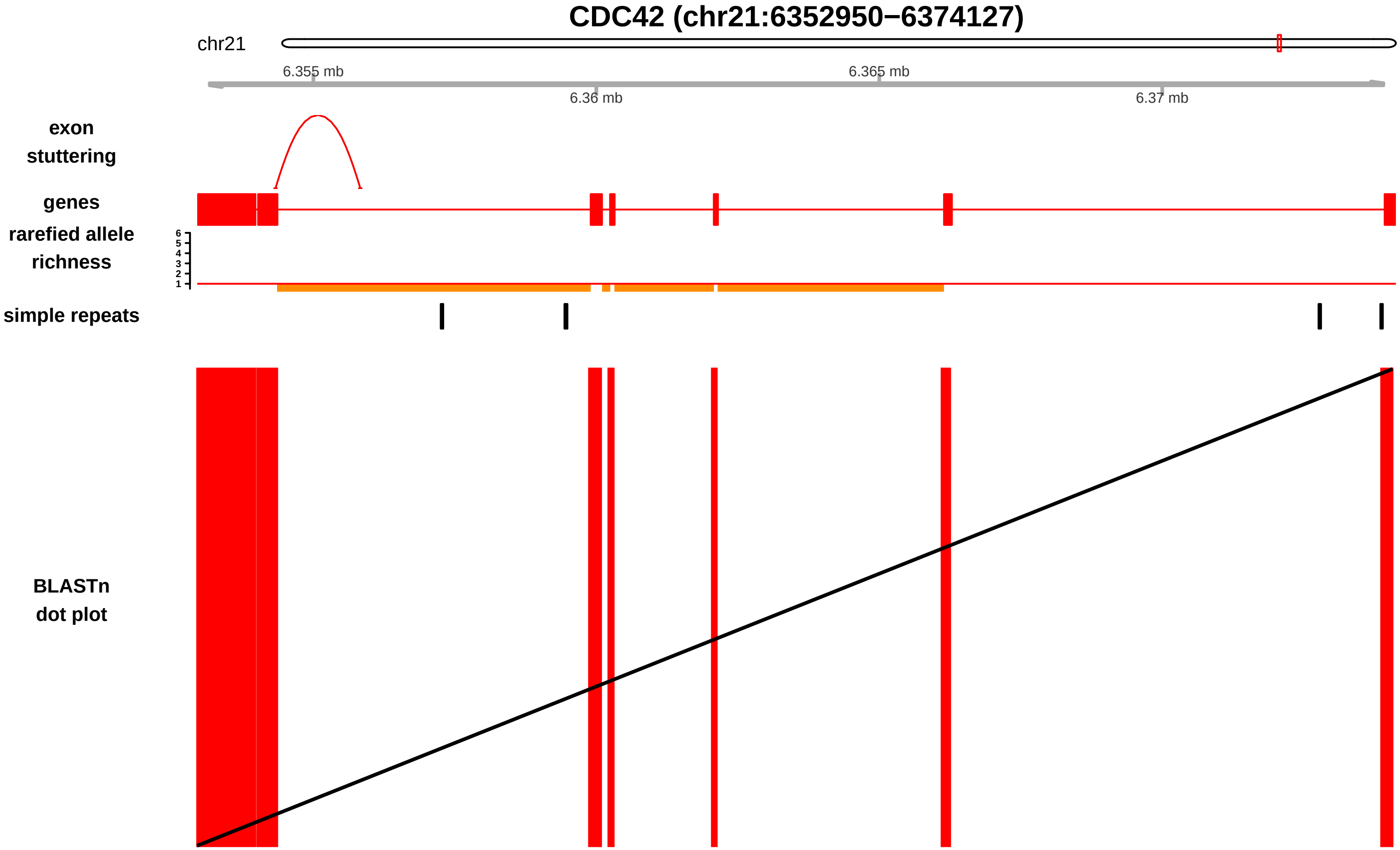
CDC42 (chr21:6352950−6374127)
chr21
6.355 mb
6.365 mb
6.36 mb
6.37 mb
exon stuttering
genes
rarefied allele richness
6
5
4
3
2
1
simple repeats
BLASTn dot plot

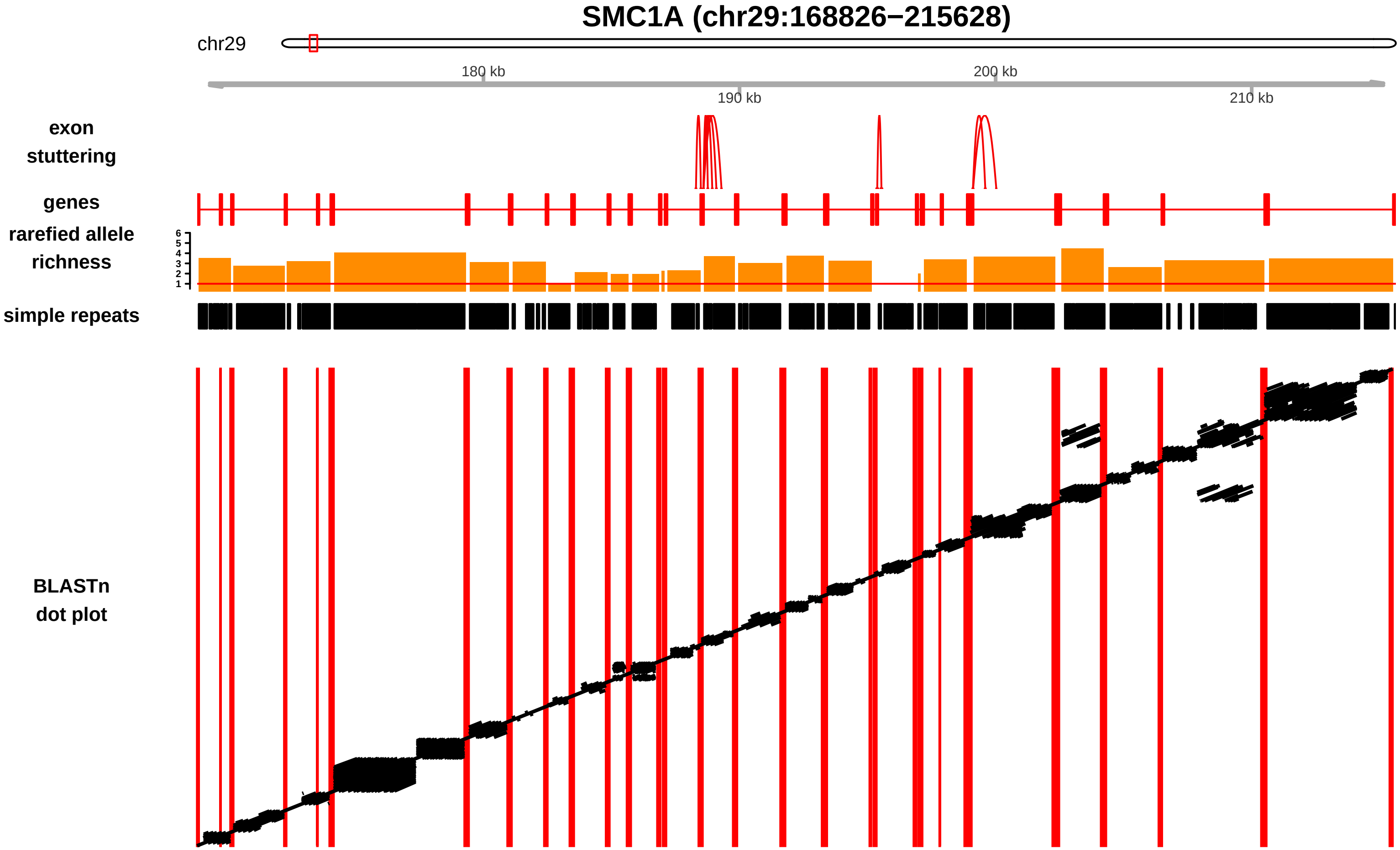

SMC1A (chr29:168826−215628)
chr29
180 kb
200 kb
190 kb
210 kb
exon stuttering
genes
rarefied allele richness
6
5
4
3
2
1
simple repeats
BLASTn dot plot

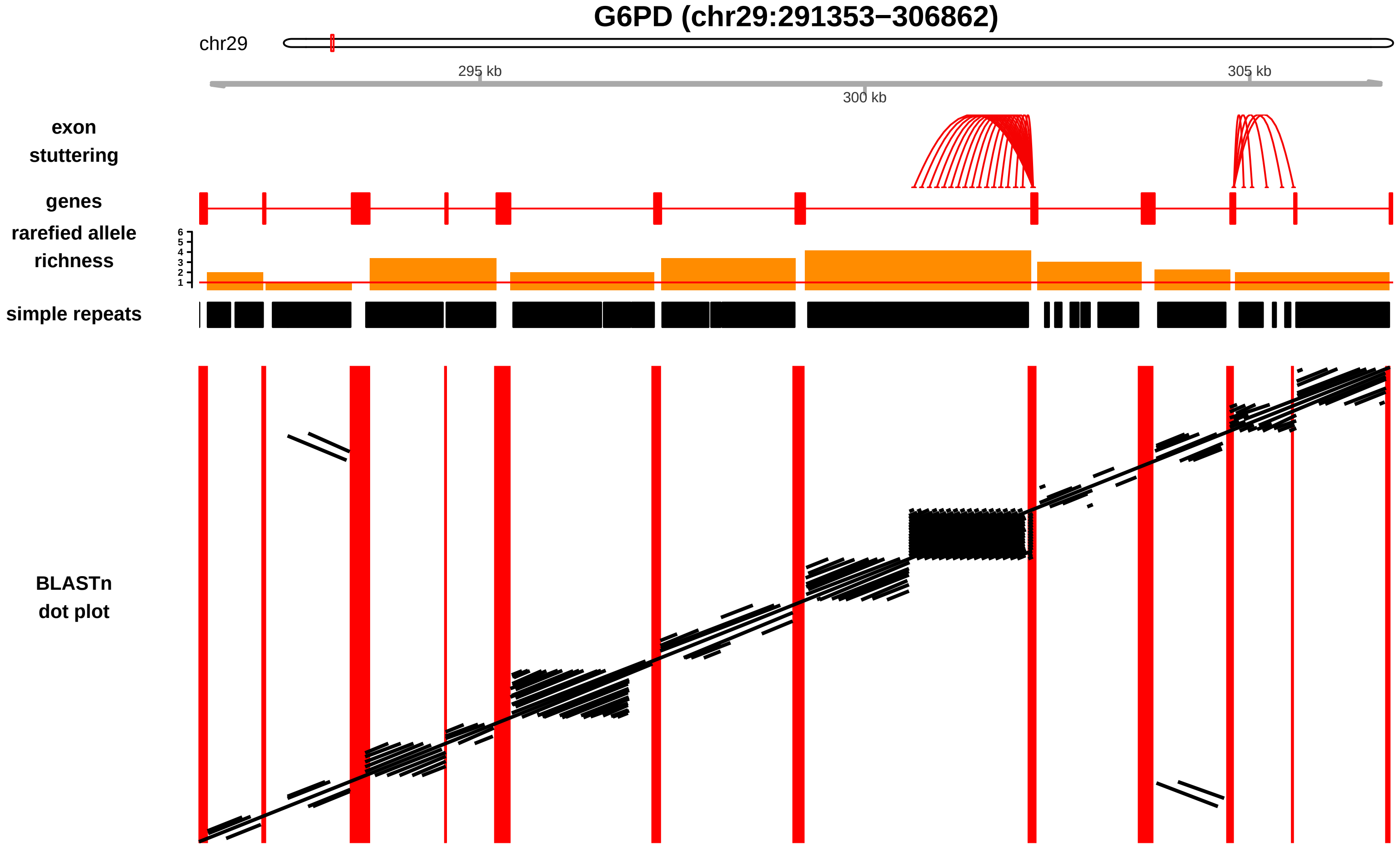
G6PD (chr29:291353−306862)
chr29
295 kb
300 kb
305 kb
exon stuttering
genes
rarefied allele richness
6
5
4
3
2
1
simple repeats
BLASTn dot plot

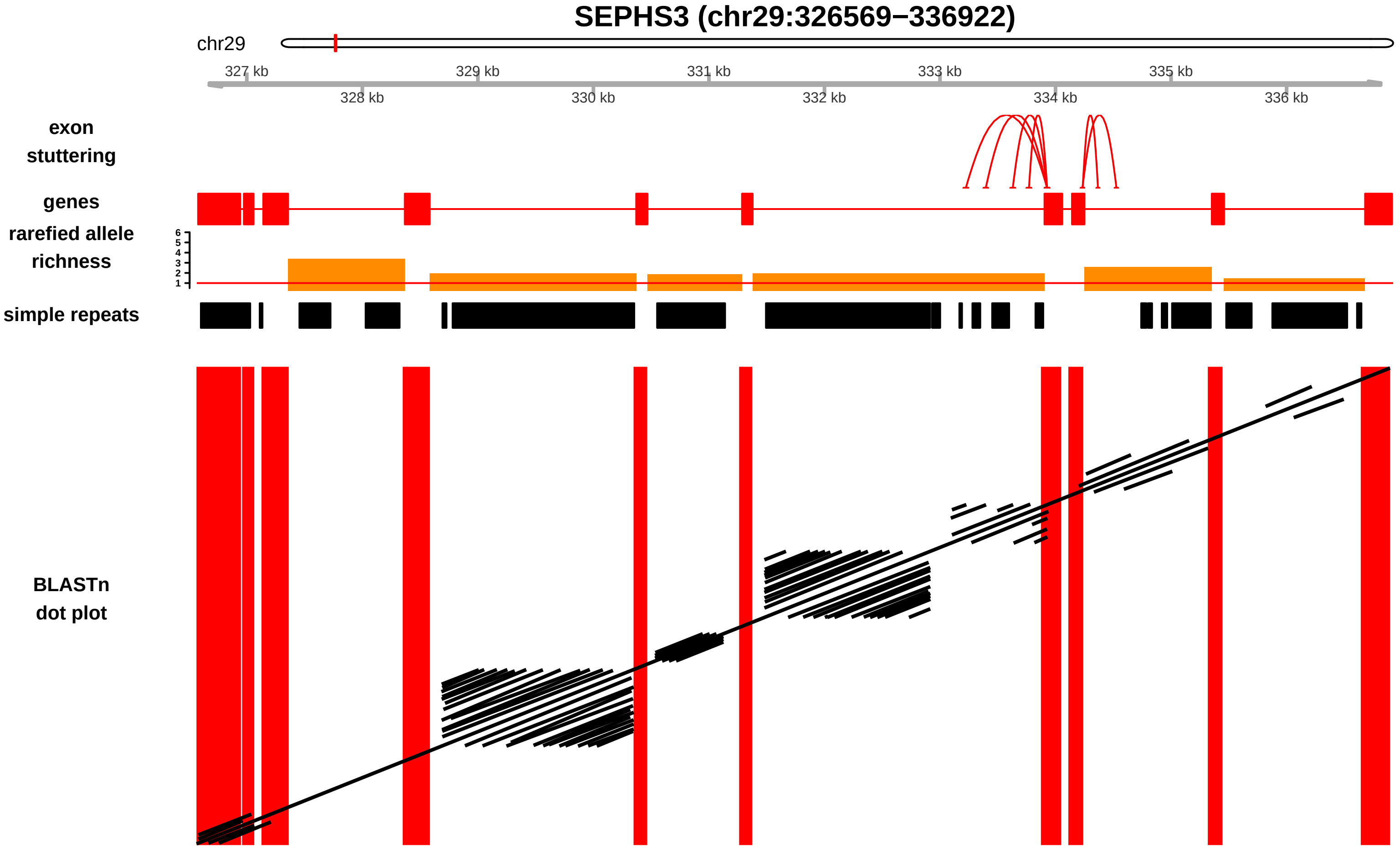

SEPHS3 (chr29:326569−336922)
chr29
327 kb
328 kb
329 kb
330 kb
331 kb
332 kb
333 kb
334 kb
335 kb
336 kb
exon stuttering
genes
rarefied allele richness
1
2
3
4
5
6
simple repeats
BLASTn dot plot

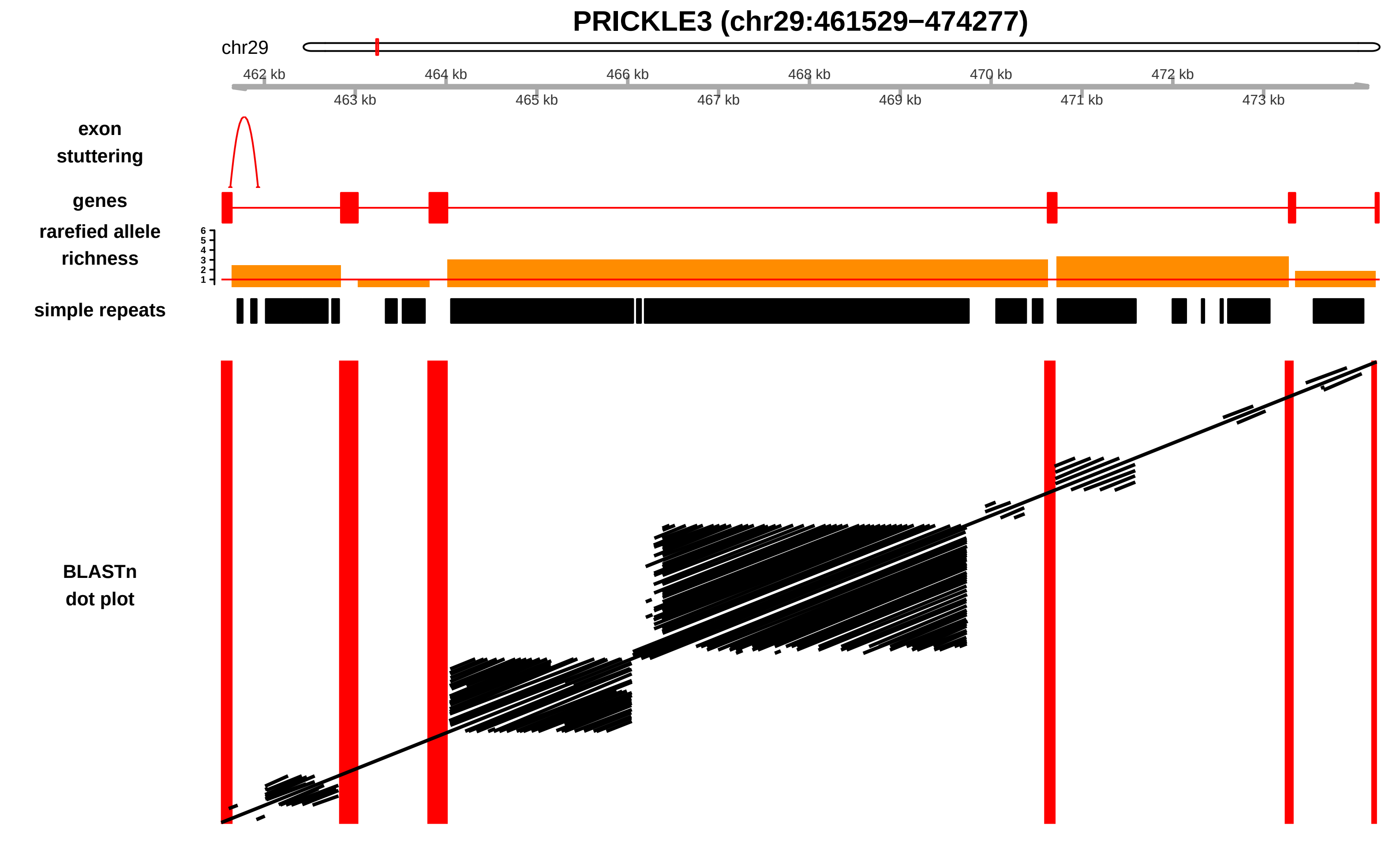
PRICKLE3 (chr29:461529−474277)
chr29
462 kb
463 kb
464 kb
465 kb
466 kb
467 kb
468 kb
469 kb
470 kb
471 kb
472 kb
473 kb
exon stuttering
genes
rarefied allele richness
1
2
3
4
5
6
simple repeats
BLASTn dot plot

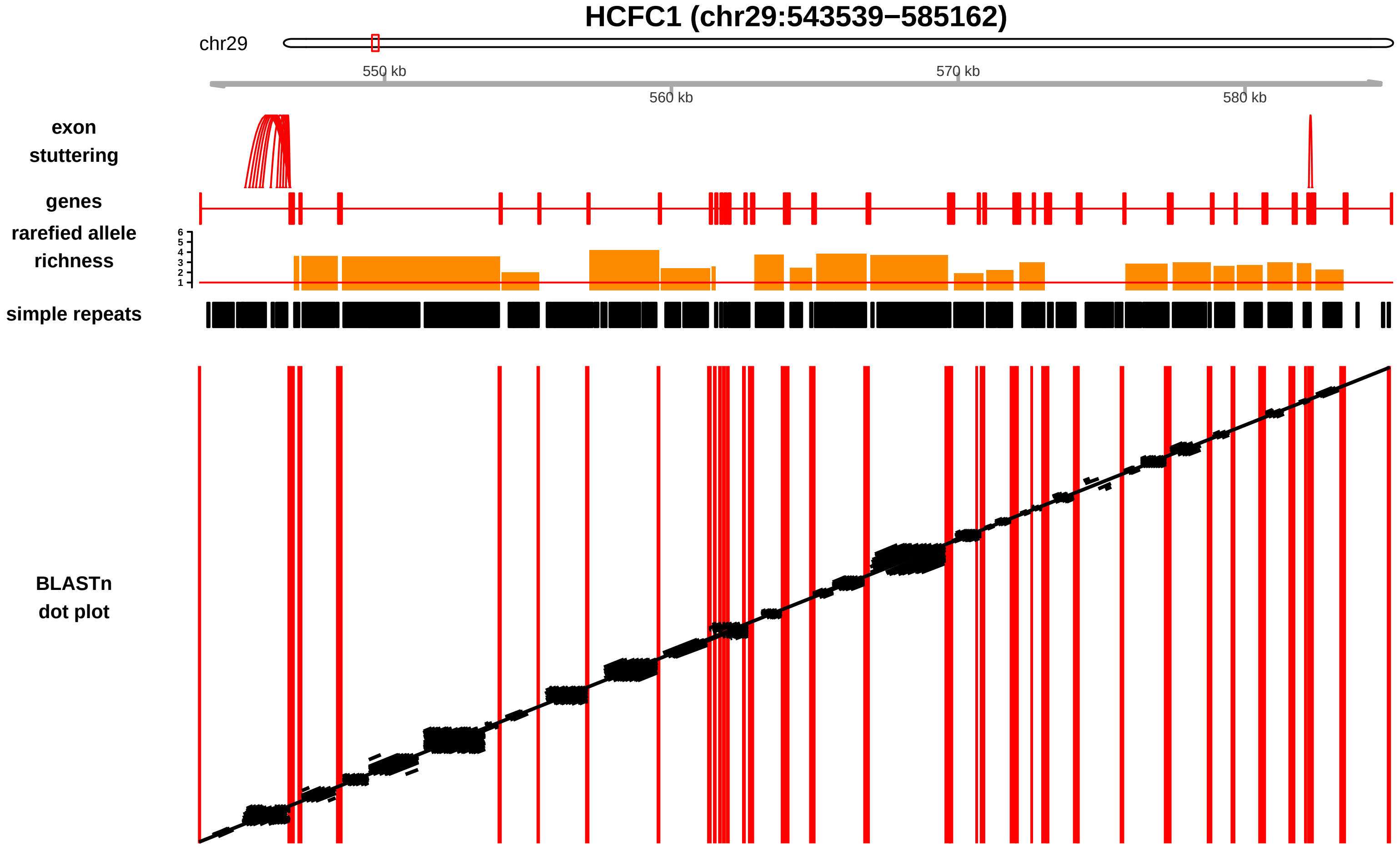

HCFC1 (chr29:543539−585162)
chr29
550 kb
570 kb
560 kb
580 kb
exon
stuttering
genes
rarefied allele
richness
6
5
4
3
2
1
simple repeats
BLASTn
dot plot

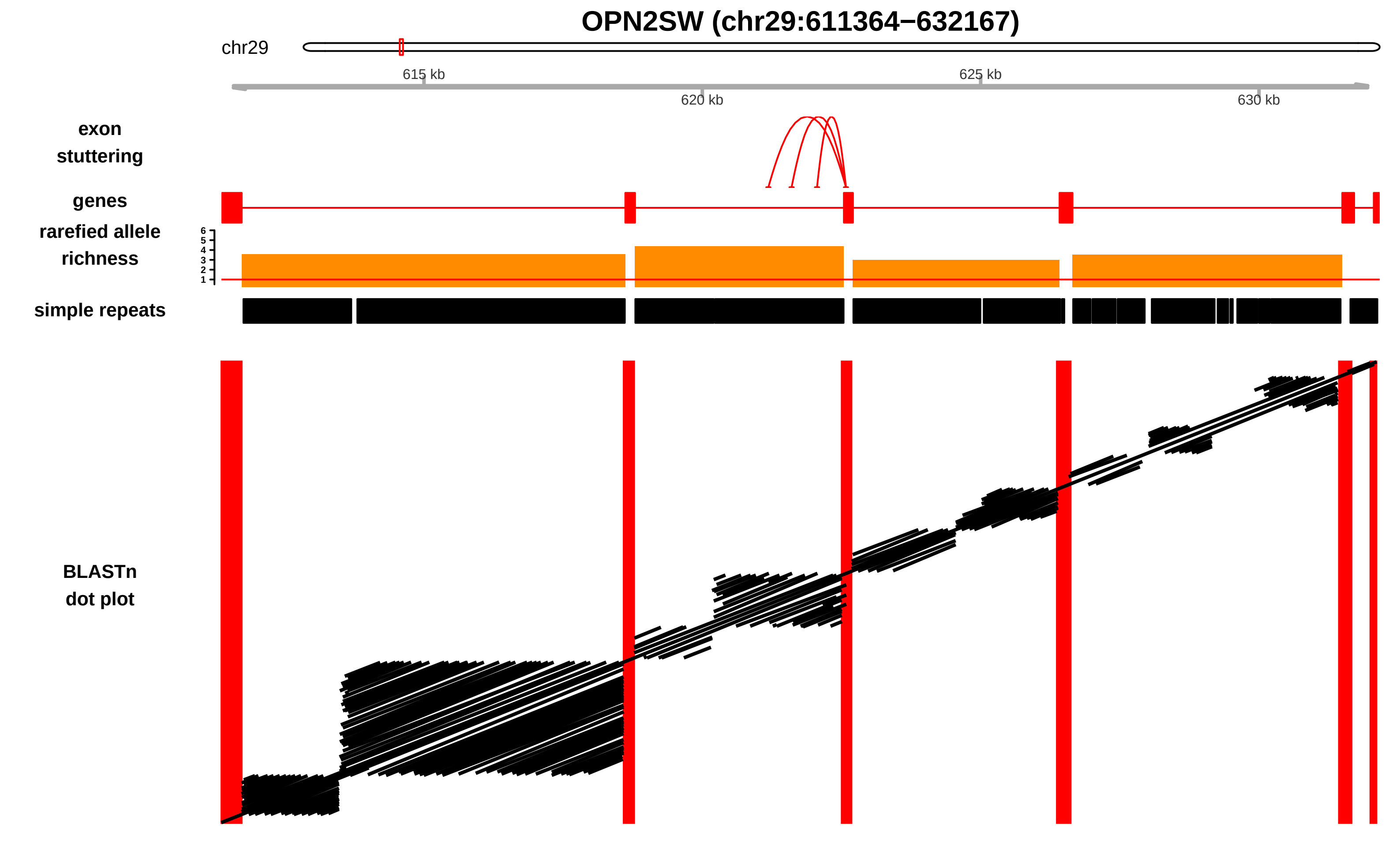

OPN2SW (chr29:611364−632167)
chr29
615 kb
620 kb
625 kb
630 kb
exon stuttering
genes
rarefied allele richness
1
2
3
4
5
6
simple repeats
BLASTn dot plot

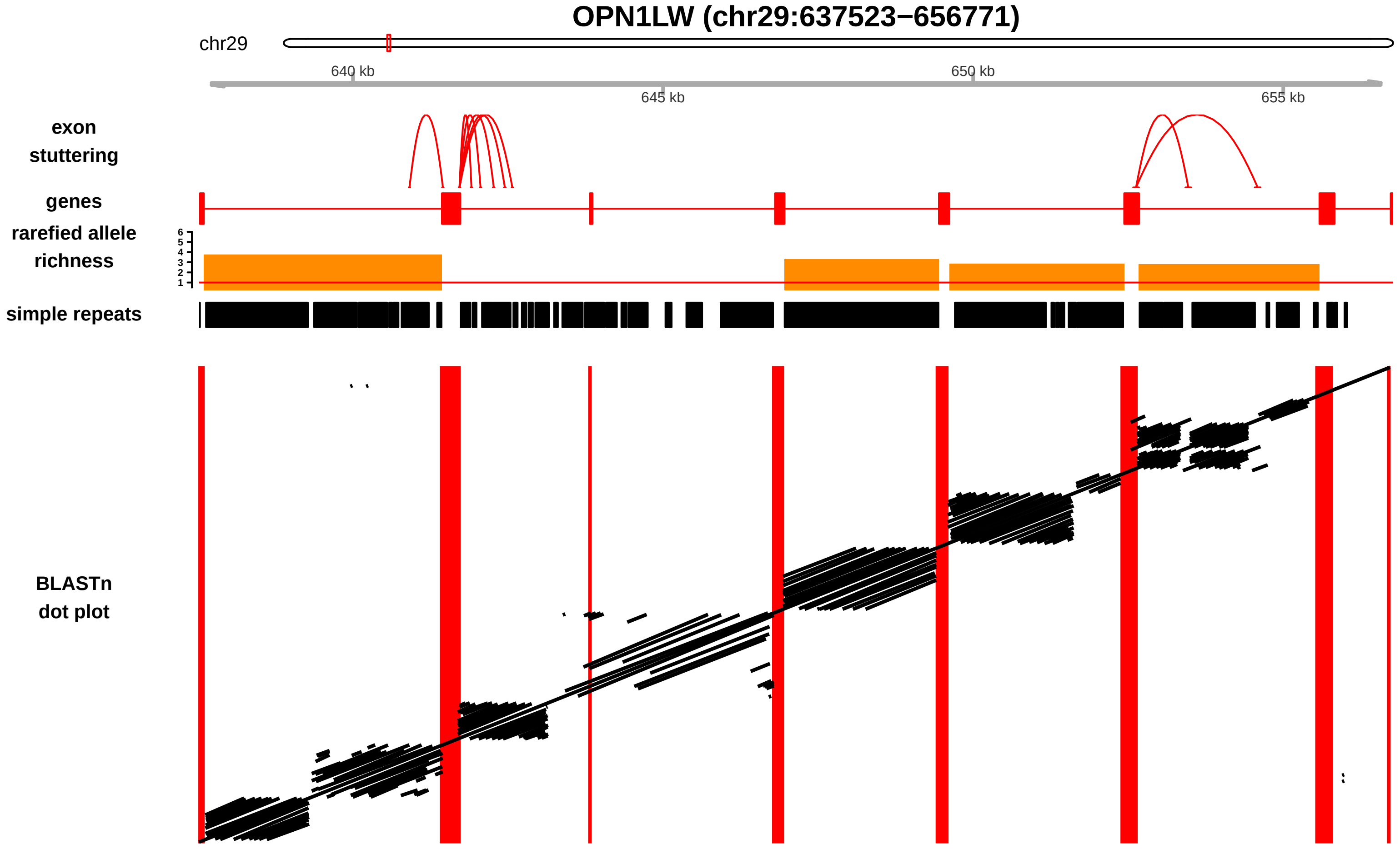
OPN1LW (chr29:637523−656771)
chr29
640 kb
645 kb
650 kb
655 kb
exon stuttering
genes
rarefied allele richness
6
5
4
3
2
1
simple repeats
BLASTn dot plot

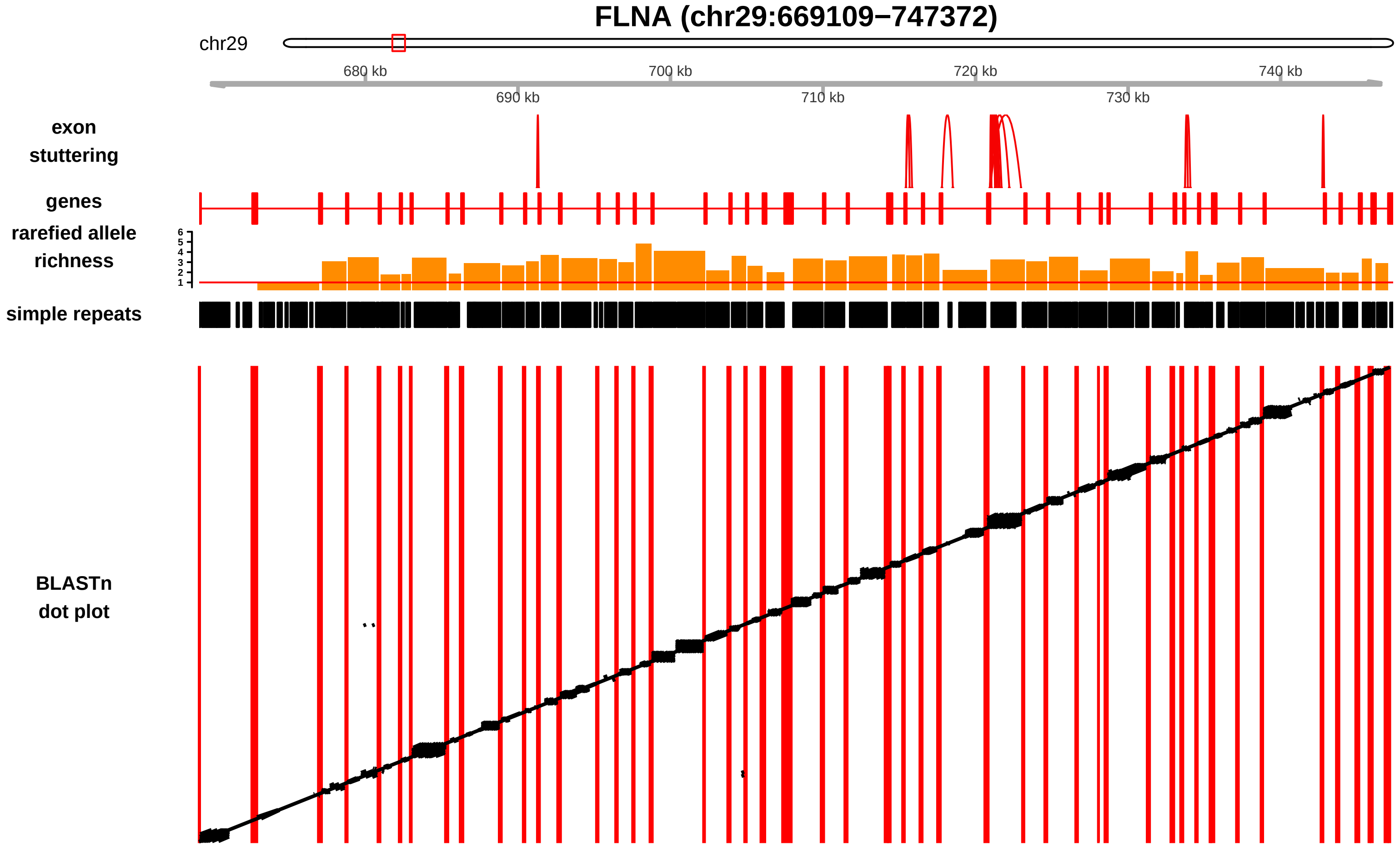
FLNA (chr29:669109−747372)
chr29
680 kb
690 kb
700 kb
710 kb
720 kb
730 kb
740 kb
exon stuttering
genes
rarefied allele richness
1
2
3
4
5
6
simple repeats
BLASTn dot plot

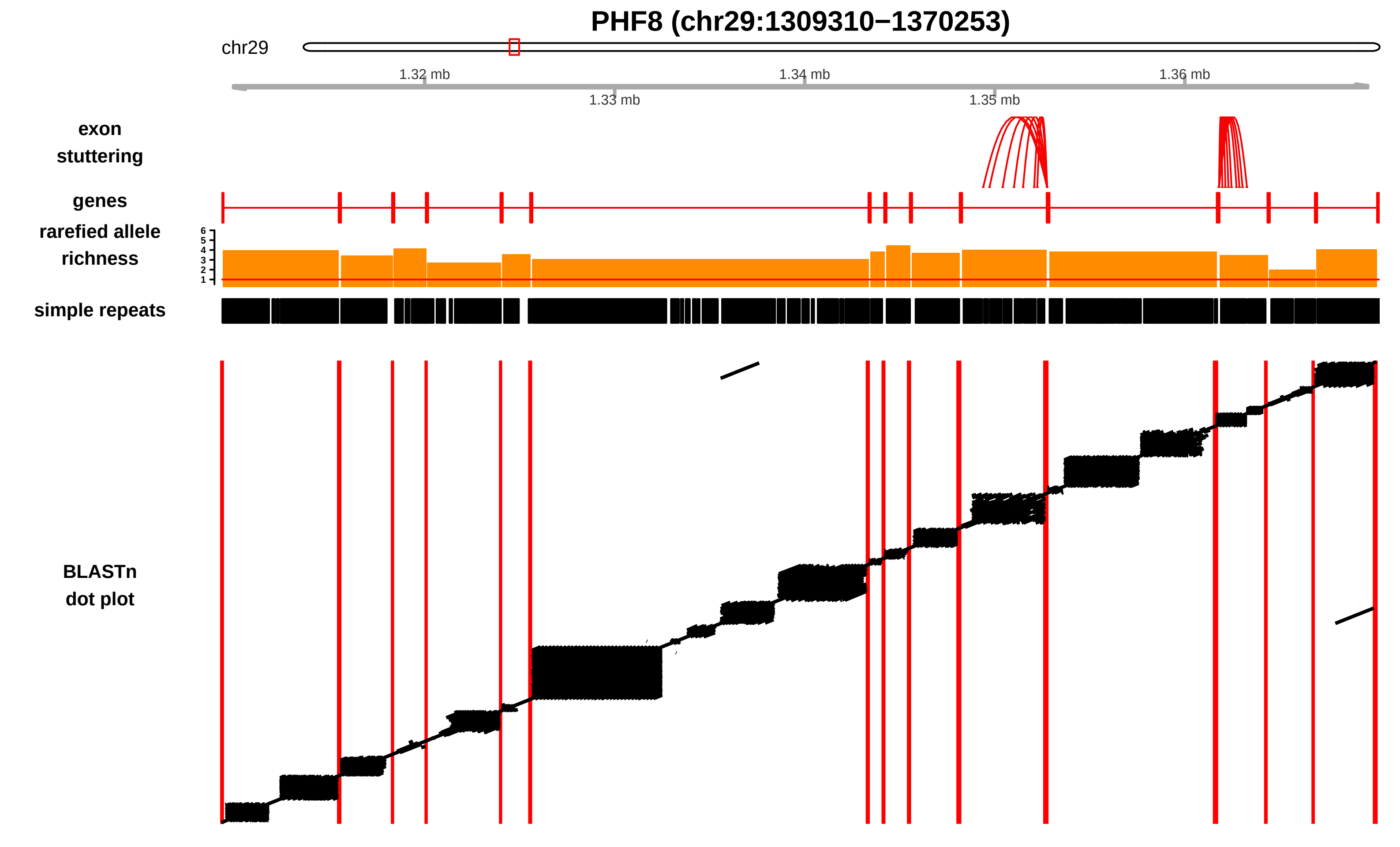

PHF8 (chr29:1309310−1370253)
chr29
1.32 mb
1.33 mb
1.34 mb
1.35 mb
1.36 mb
exon stuttering
genes
rarefied allele richness
1
2
3
4
5
6
simple repeats
BLASTn dot plot

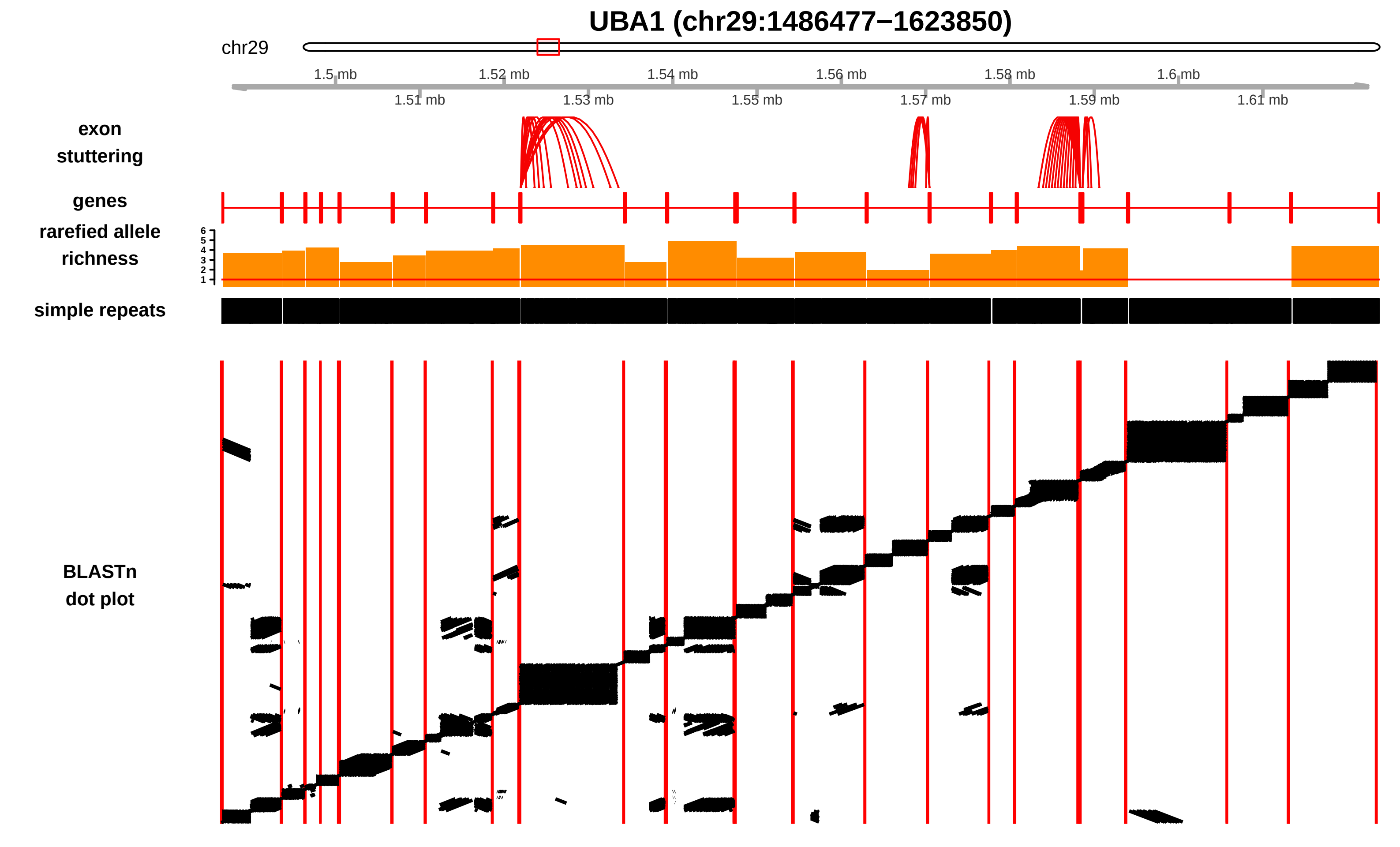

UBA1 (chr29:1486477−1623850)
chr29
1.5 mb
1.52 mb
1.54 mb
1.56 mb
1.58 mb
1.6 mb
1.51 mb
1.53 mb
1.55 mb
1.57 mb
1.59 mb
1.61 mb
exon stuttering
genes
rarefied allele richness
6
5
4
3
2
1
simple repeats
BLASTn dot plot

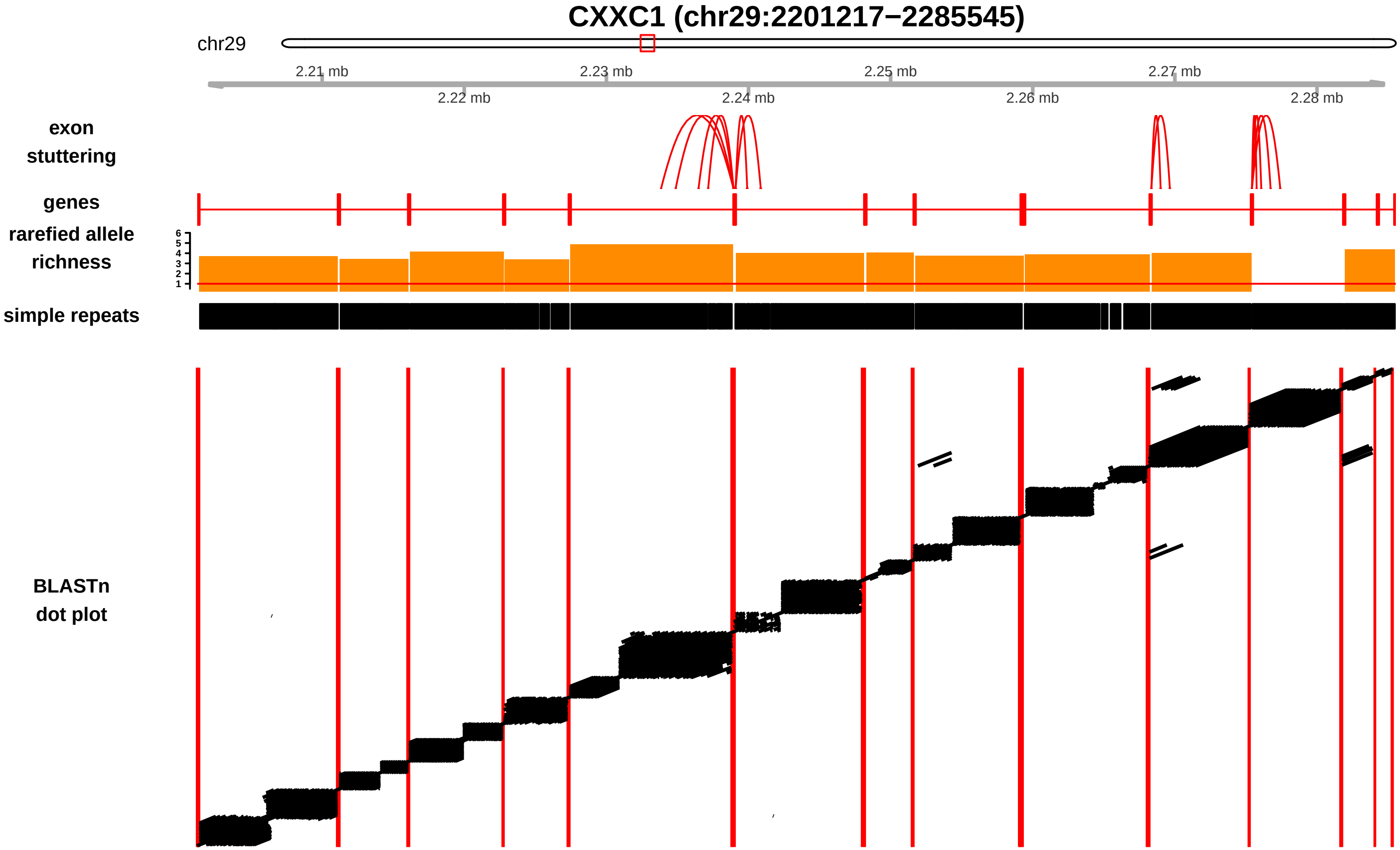
CXXC1 (chr29:2201217−2285545)
chr29
2.21 mb
2.22 mb
2.23 mb
2.24 mb
2.25 mb
2.26 mb
2.27 mb
2.28 mb
exon stuttering
genes
rarefied allele richness
1
2
3
4
5
6
simple repeats
BLASTn dot plot

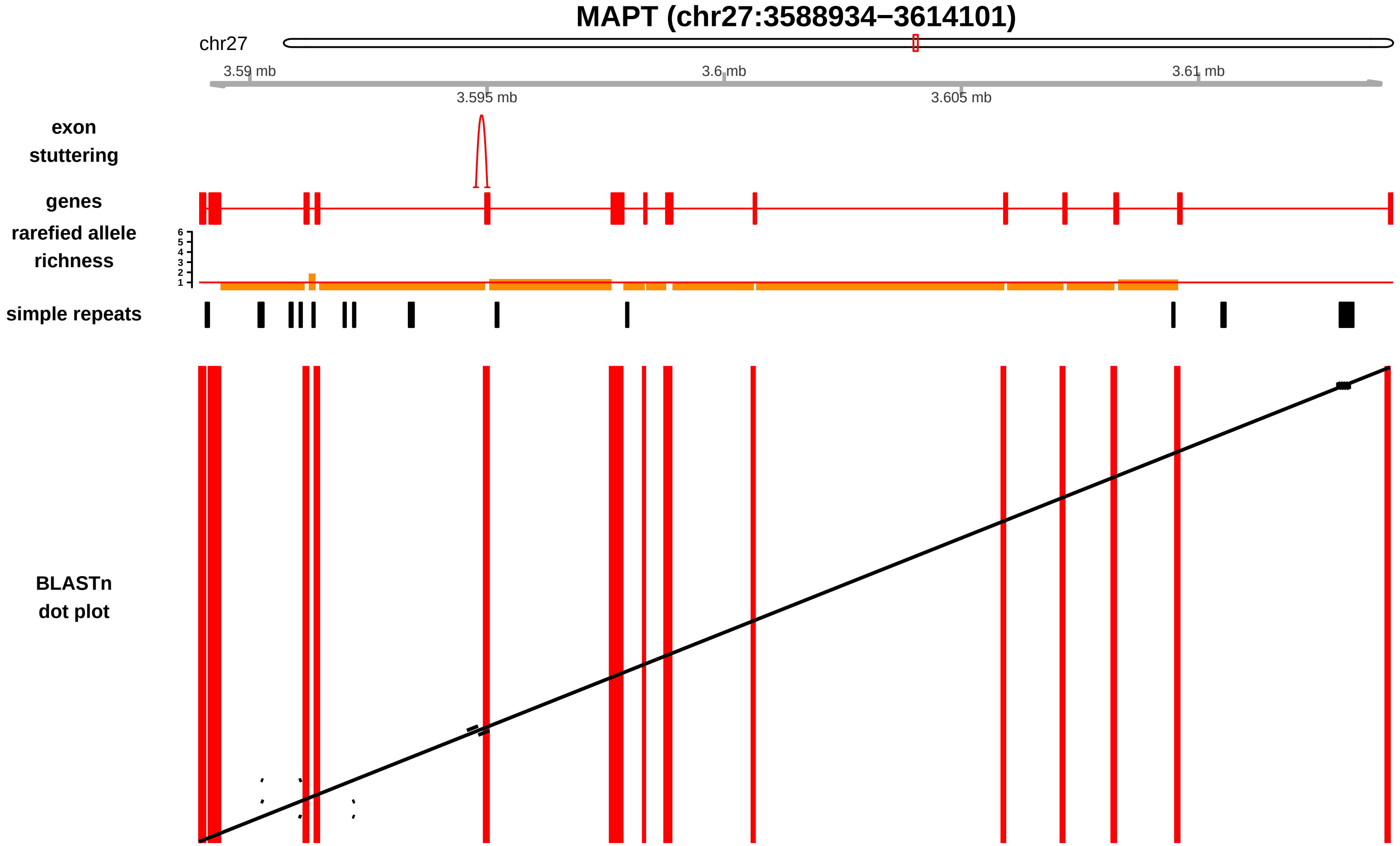
MAPT (chr27:3588934–3614101)
chr27
3.59 mb
3.595 mb
3.6 mb
3.605 mb
3.61 mb
exon stuttering
genes
rarefied allele richness
6
5
4
3
2
1
simple repeats
BLASTn dot plot

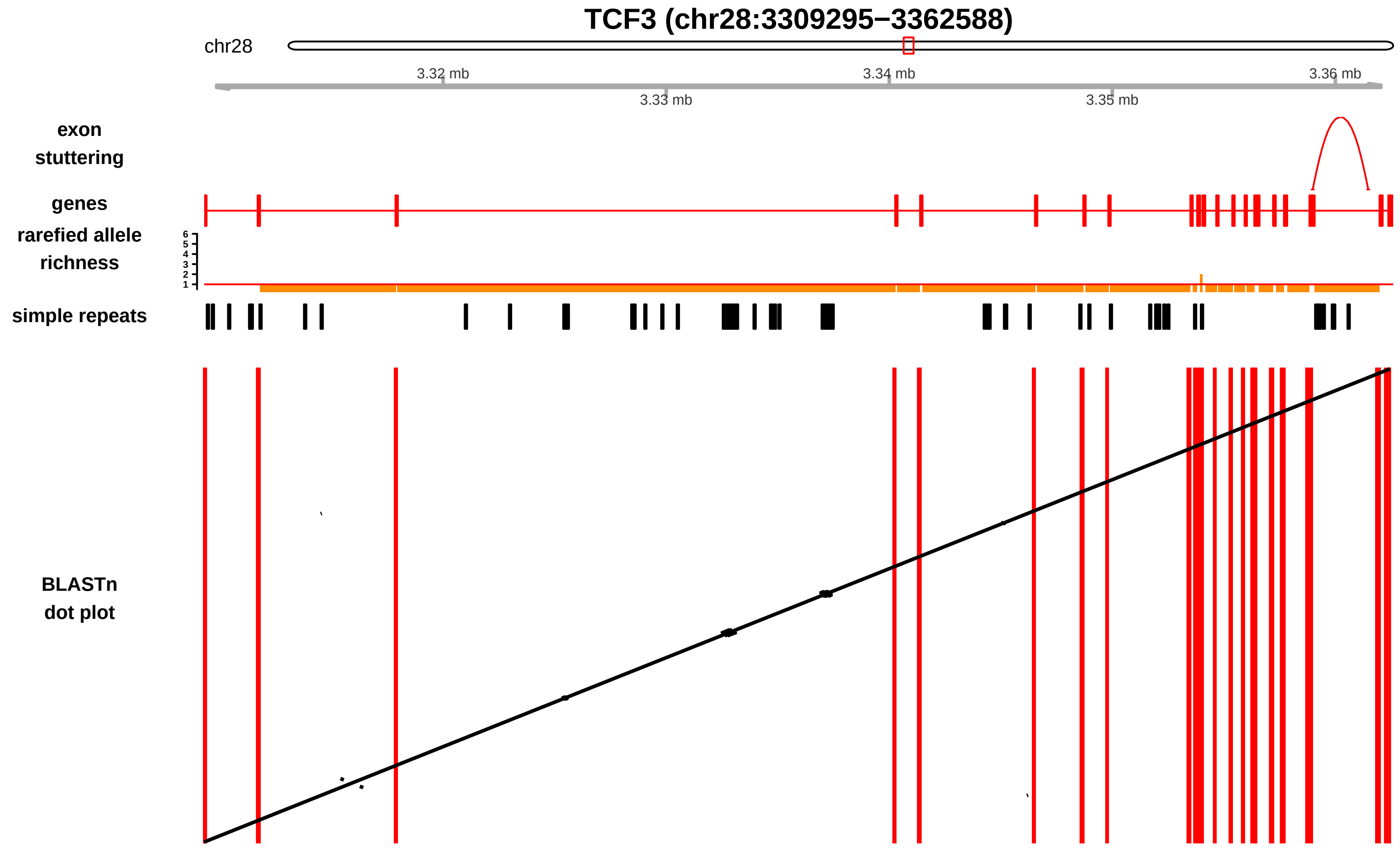

TCF3 (chr28:3309295−3362588)
chr28
3.32 mb
3.33 mb
3.34 mb
3.35 mb
3.36 mb
exon stuttering
genes
rarefied allele richness
6
5
4
3
2
1
simple repeats
BLASTn dot plot

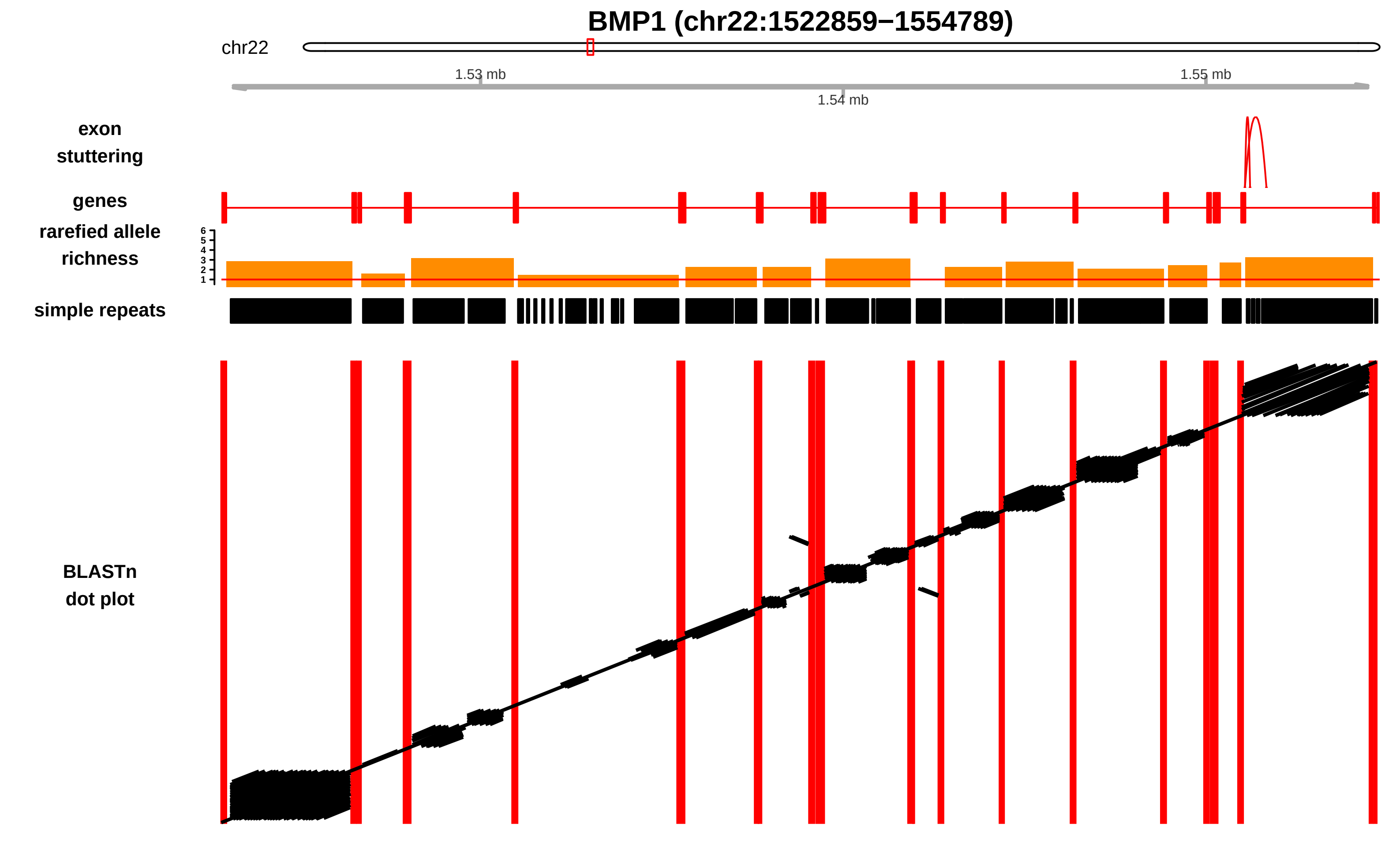

BMP1 (chr22:1522859−1554789)
chr22
1.53 mb
1.54 mb
1.55 mb
exon stuttering
genes
rarefied allele richness
6
5
4
3
2
1
simple repeats
BLASTn dot plot

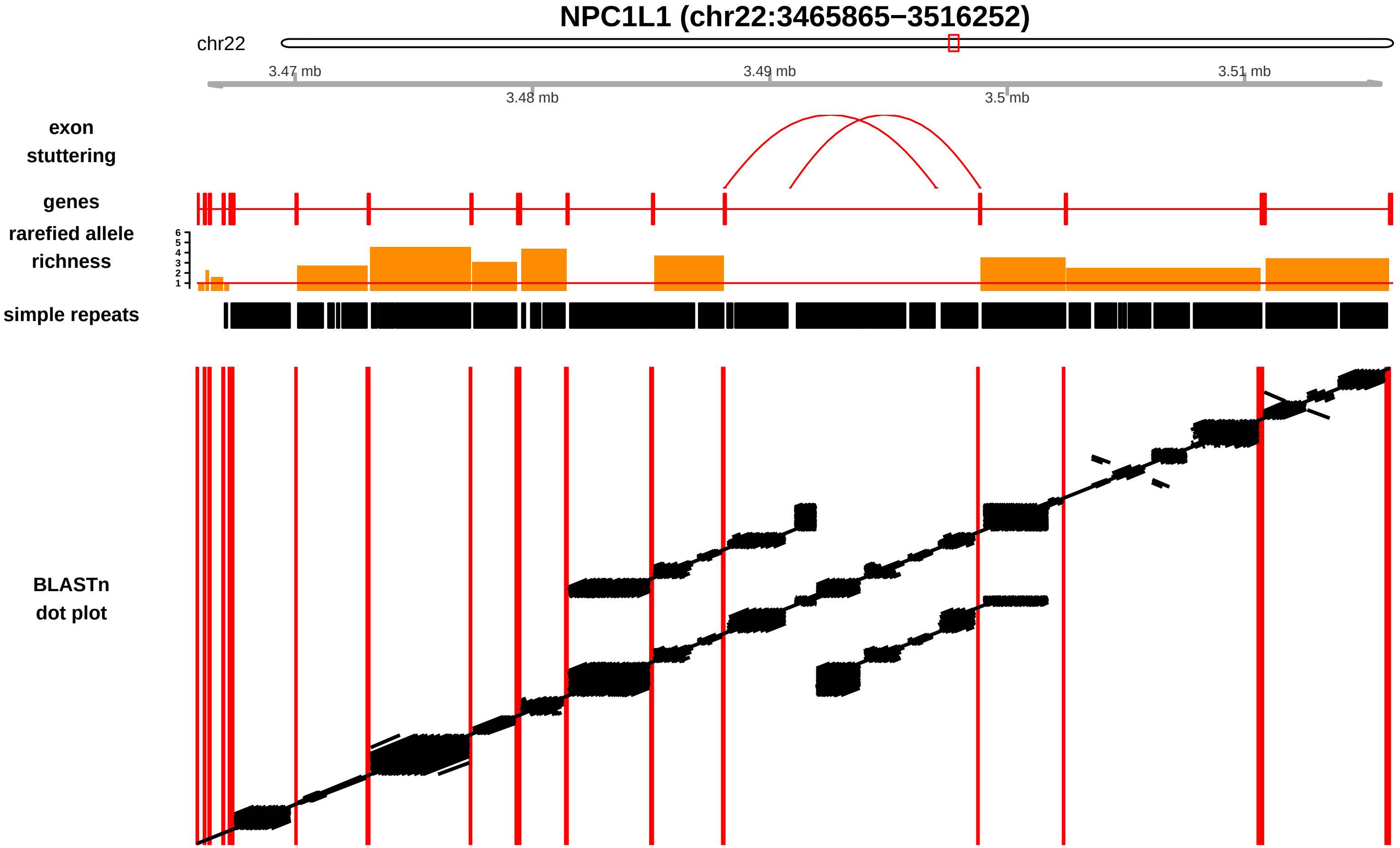

NPC1L1 (chr22:3465865−3516252)
chr22
3.47 mb
3.48 mb
3.49 mb
3.5 mb
3.51 mb
exon stuttering
genes
rarefied allele richness
1
2
3
4
5
6
simple repeats
BLASTn dot plot

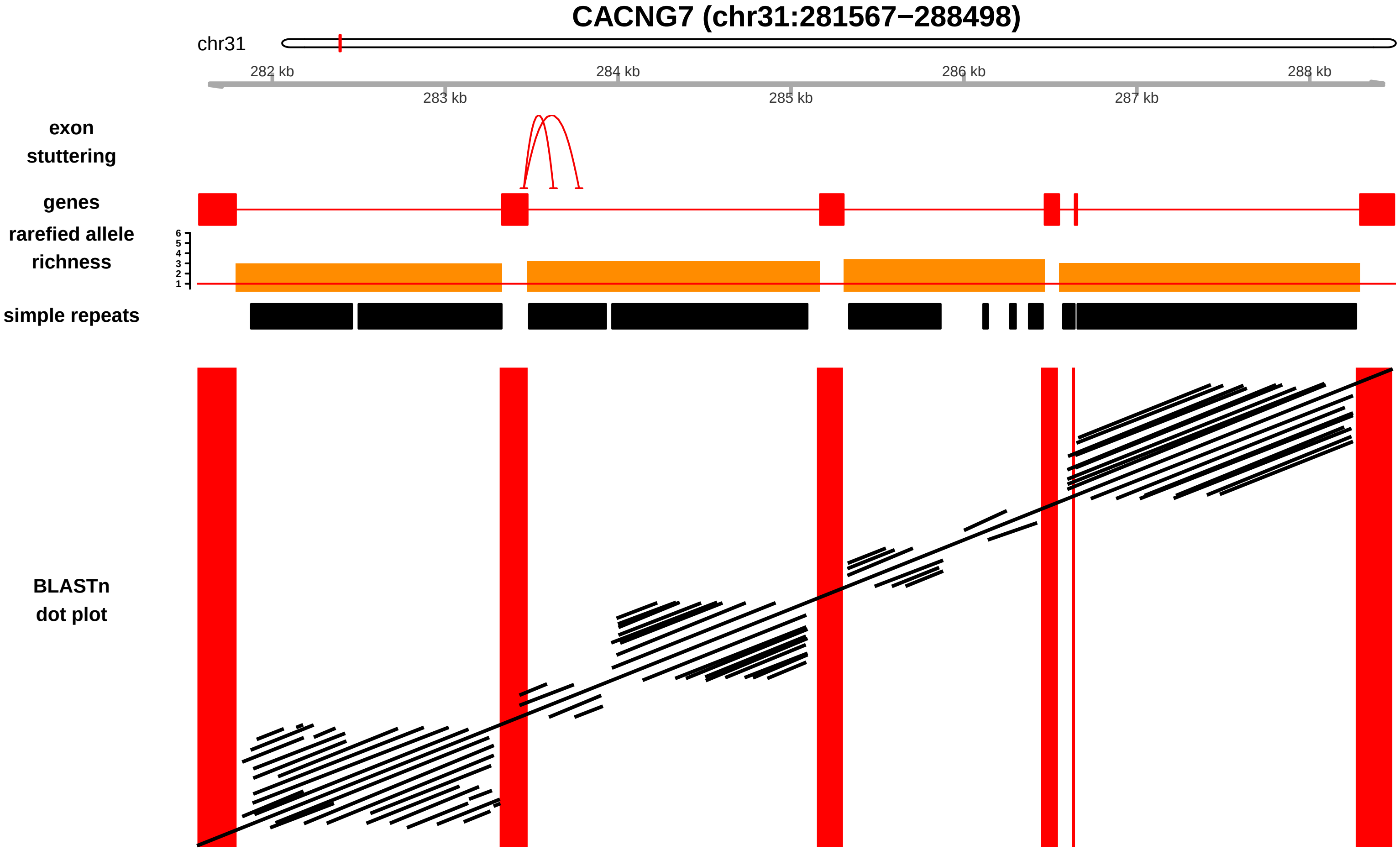

CACNG7 (chr31:281567−288498)
chr31
282 kb
283 kb
284 kb
285 kb
286 kb
287 kb
288 kb
exon stuttering
genes
rarefied allele richness
6
5
4
3
2
1
simple repeats
BLASTn dot plot

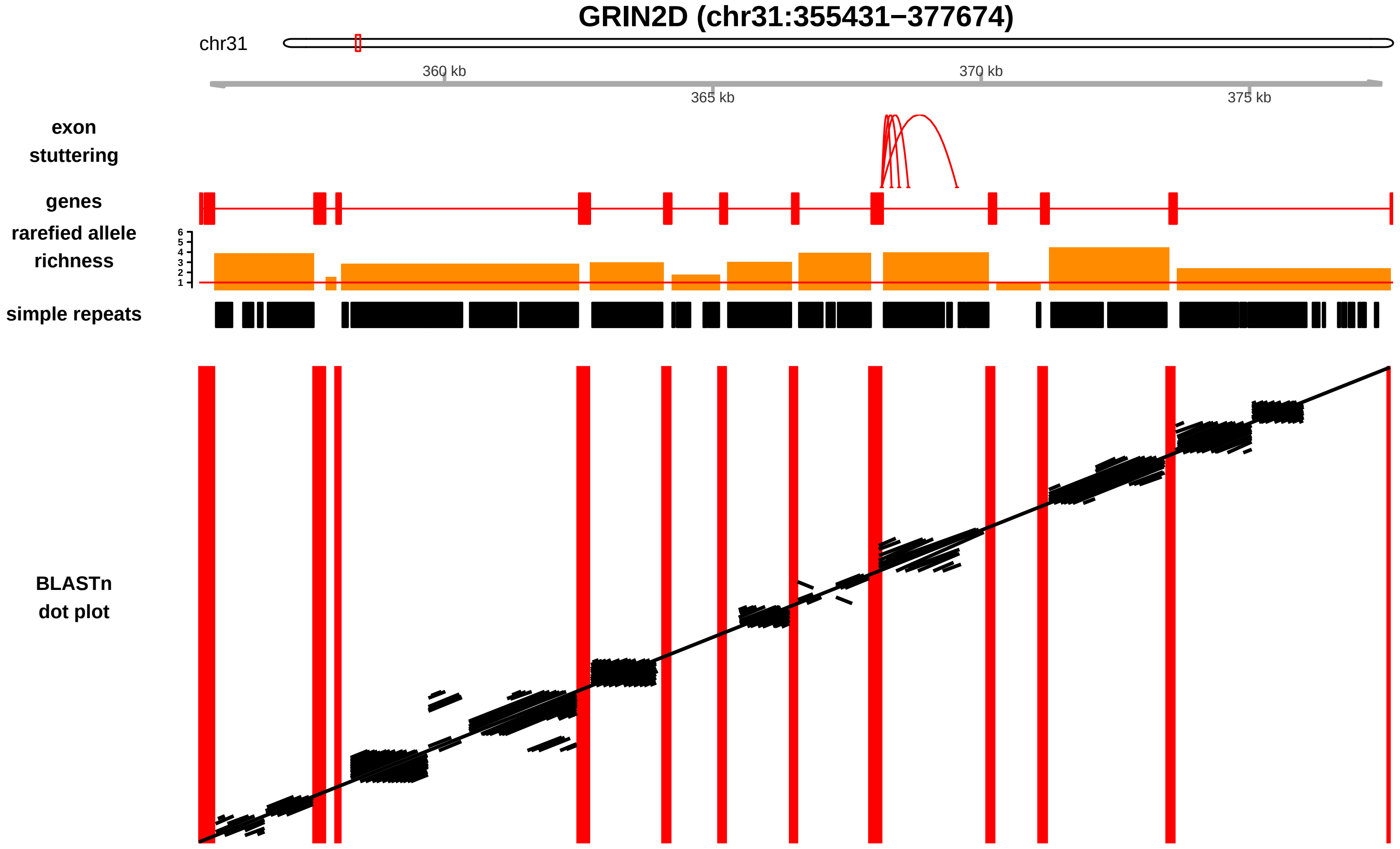
GRIN2D (chr31:355431−377674)
chr31
360 kb
365 kb
370 kb
375 kb
exon stuttering
genes
rarefied allele richness
6
5
4
3
2
1
simple repeats
BLASTn dot plot

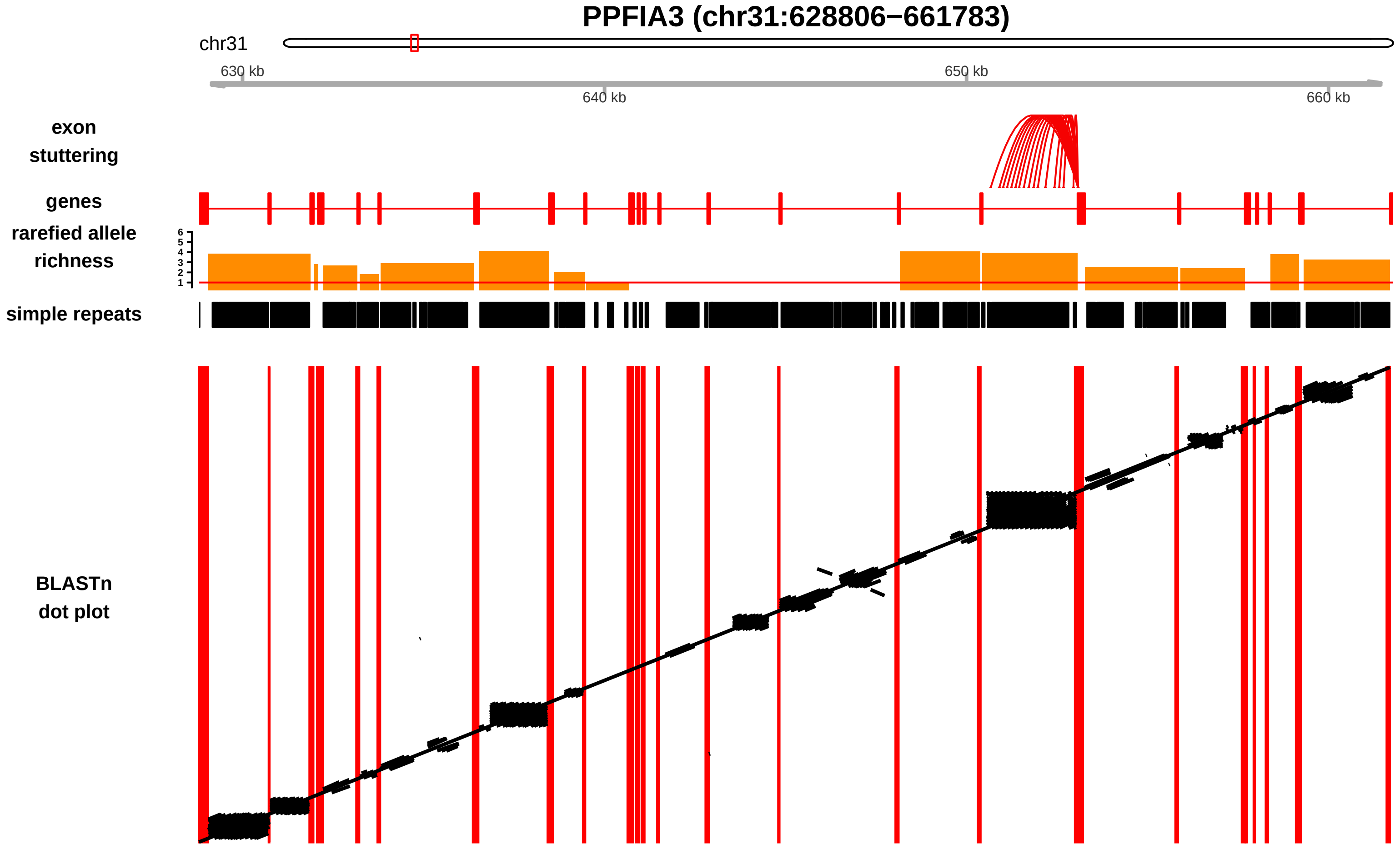
PPFIA3 (chr31:628806−661783)
chr31
630 kb
640 kb
650 kb
660 kb
exon stuttering
genes
rarefied allele richness
1
2
3
4
5
6
simple repeats
BLASTn dot plot

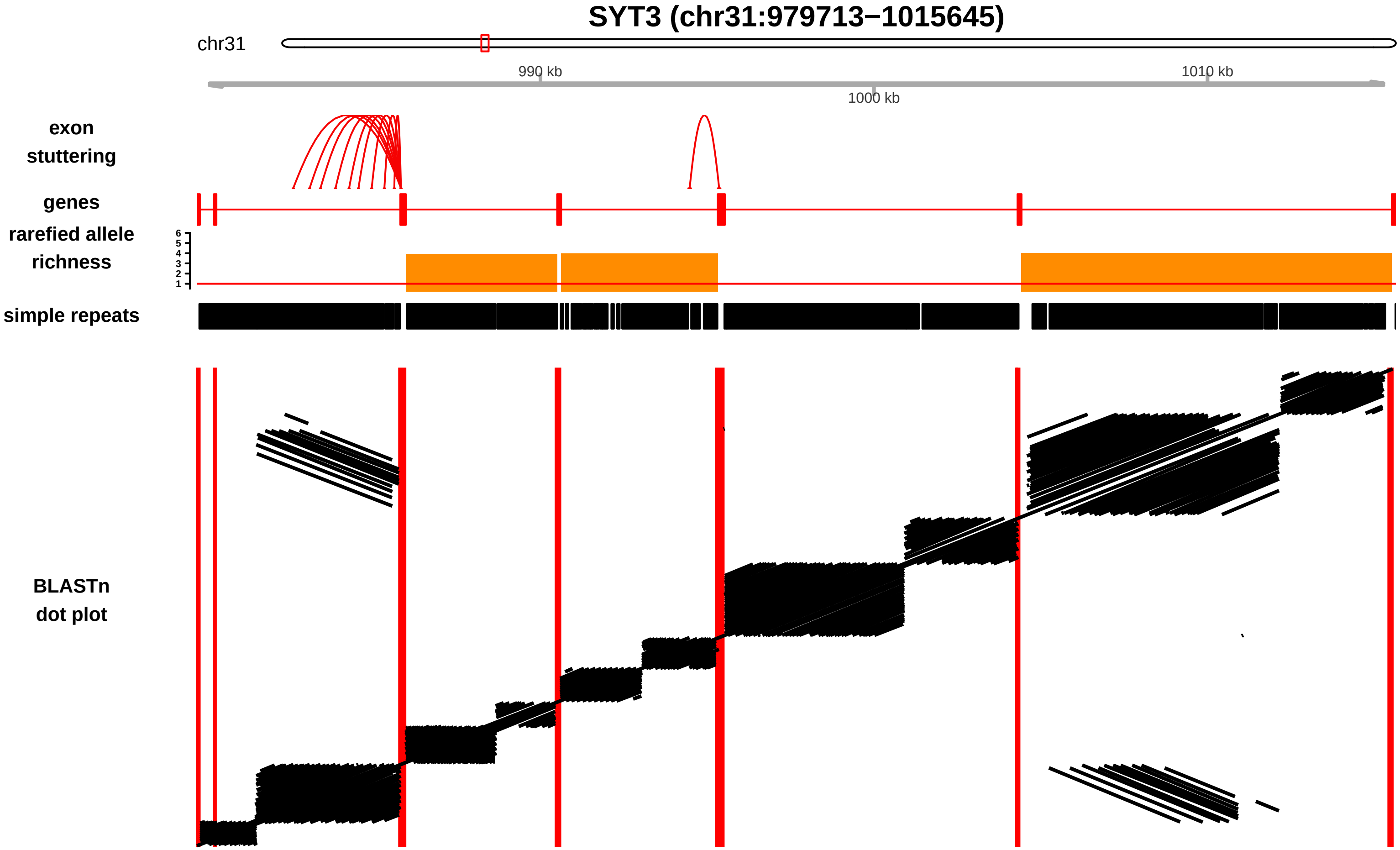

SYT3 (chr31:979713−1015645)
chr31
990 kb
1000 kb
1010 kb
exon stuttering
genes
rarefied allele richness
6
5
4
3
2
1
simple repeats
BLASTn dot plot

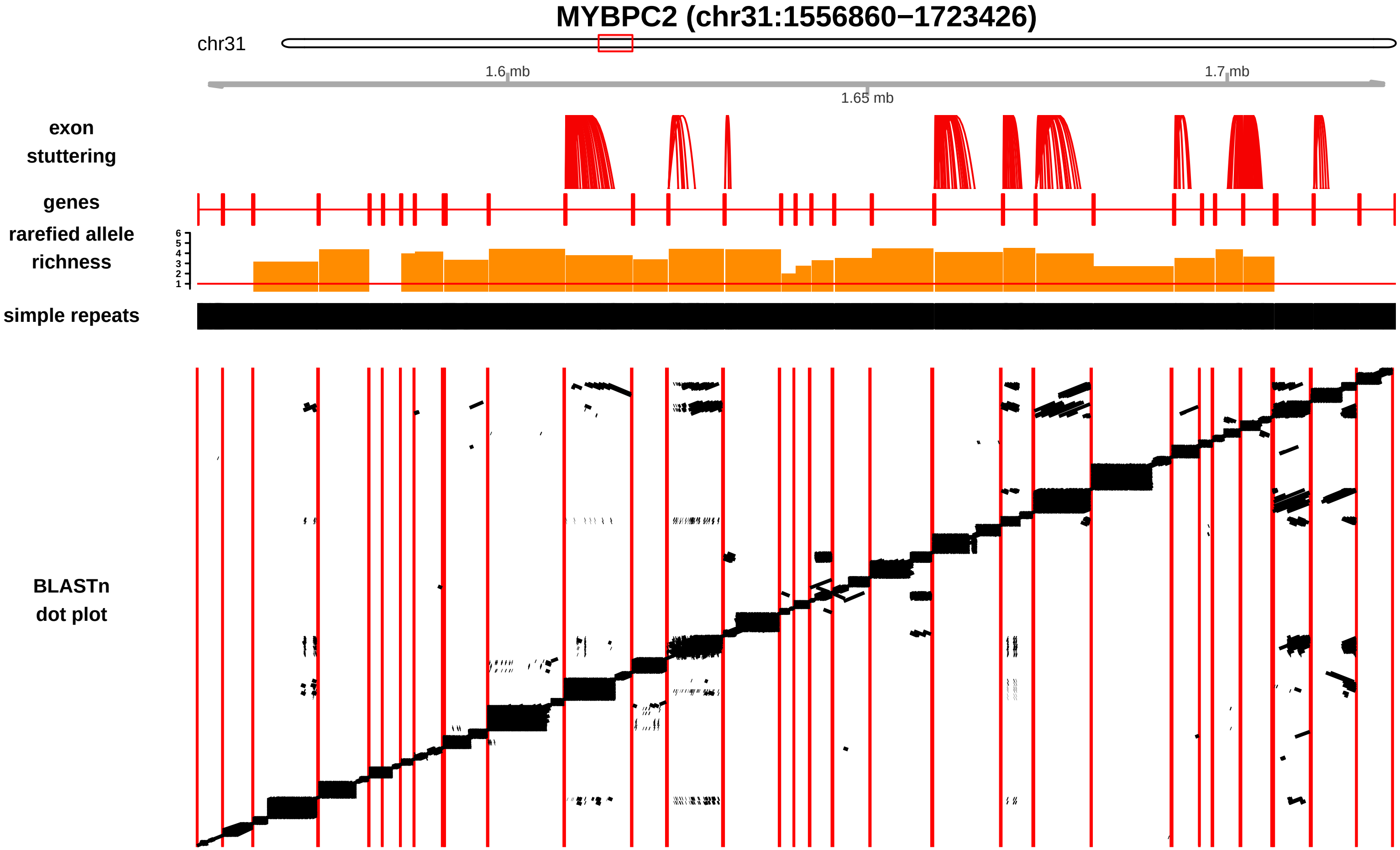

MYBPC2 (chr31:1556860−1723426)
chr31
1.6 mb
1.65 mb
1.7 mb
exon stuttering
genes
rarefied allele richness
6
5
4
3
2
1
simple repeats
BLASTn dot plot

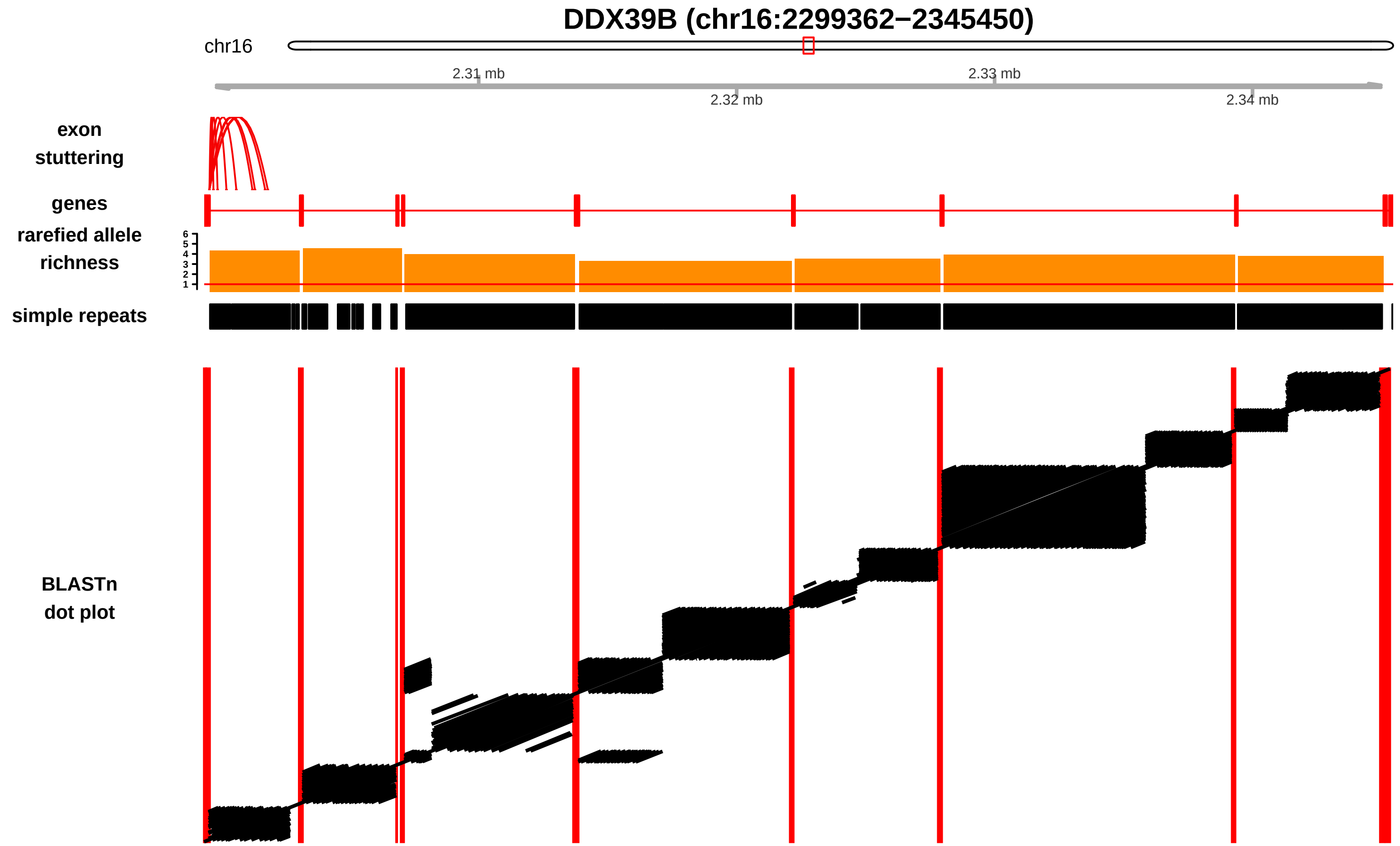

DDX39B (chr16:2299362−2345450)
chr16
2.31 mb
2.32 mb
2.33 mb
2.34 mb
exon stuttering
genes
rarefied allele richness
1
2
3
4
5
6
simple repeats
BLASTn dot plot

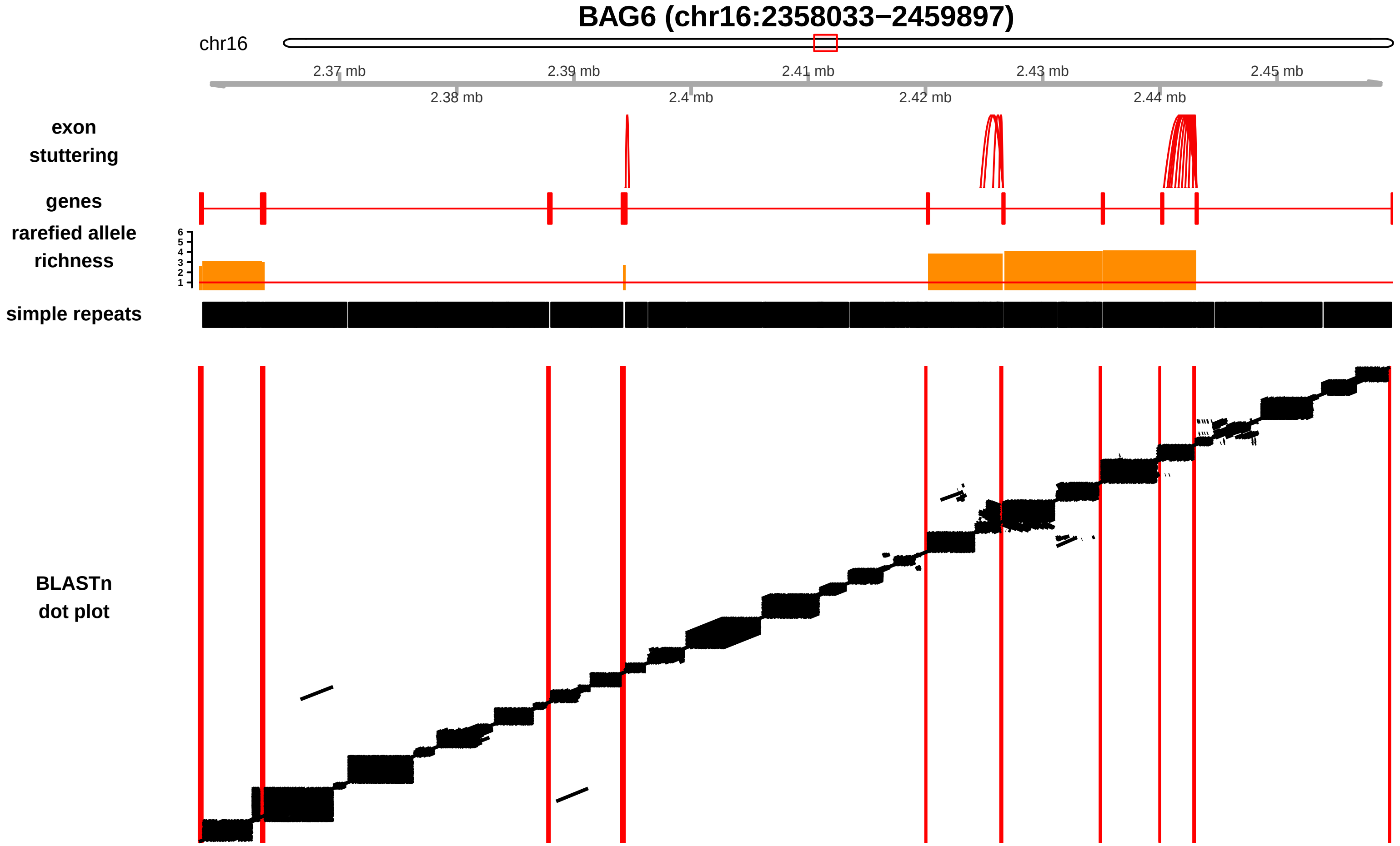

BAG6 (chr16:2358033−2459897)
chr16
2.37 mb
2.38 mb
2.39 mb
2.4 mb
2.41 mb
2.42 mb
2.43 mb
2.44 mb
2.45 mb
exon stuttering
genes
rarefied allele richness
1
2
3
4
5
6
simple repeats
BLASTn dot plot

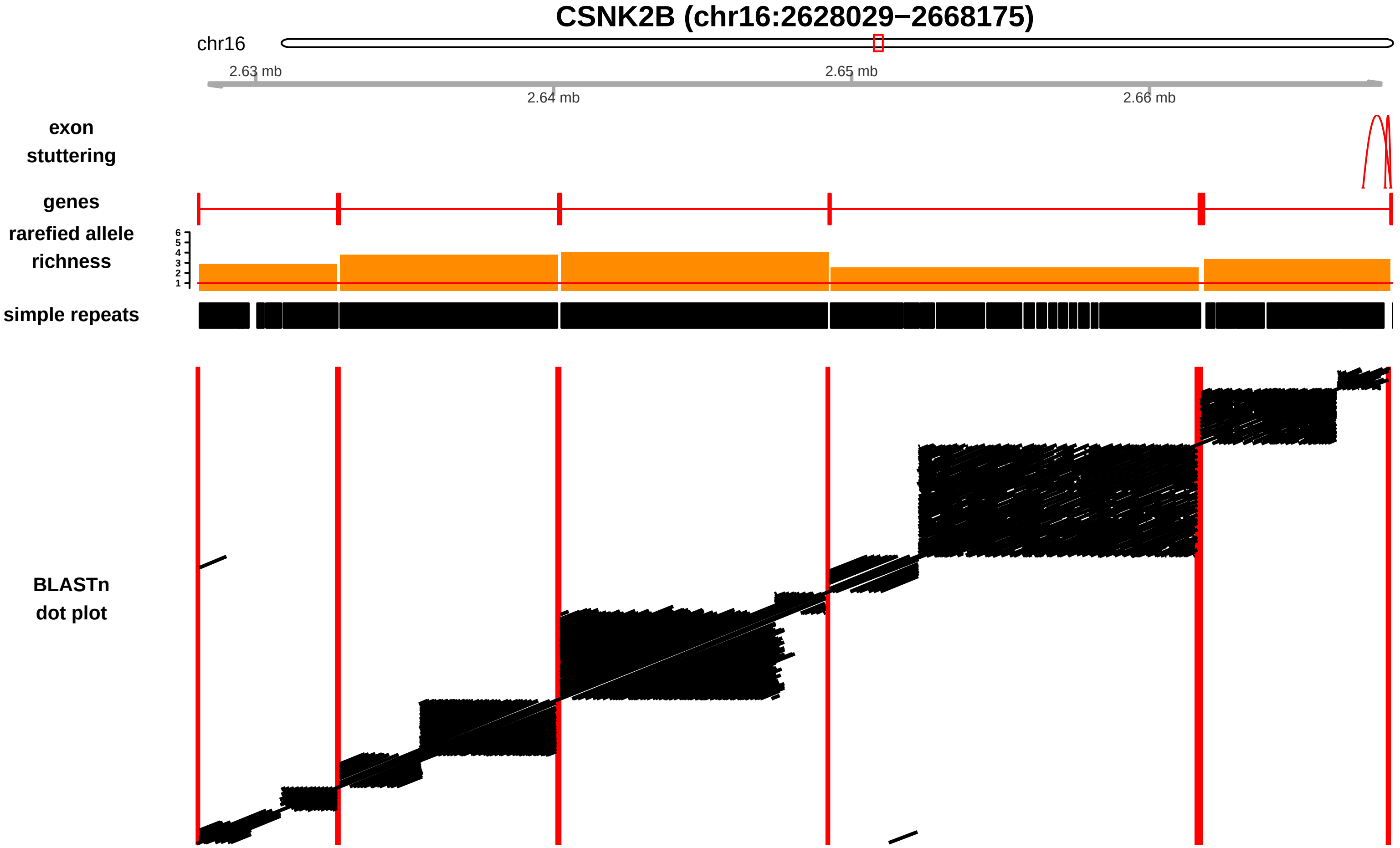
CSNK2B (chr16:2628029−2668175)
chr16
2.63 mb
2.64 mb
2.65 mb
2.66 mb
exon stuttering
genes
rarefied allele richness
6
5
4
3
2
1
simple repeats
BLASTn dot plot

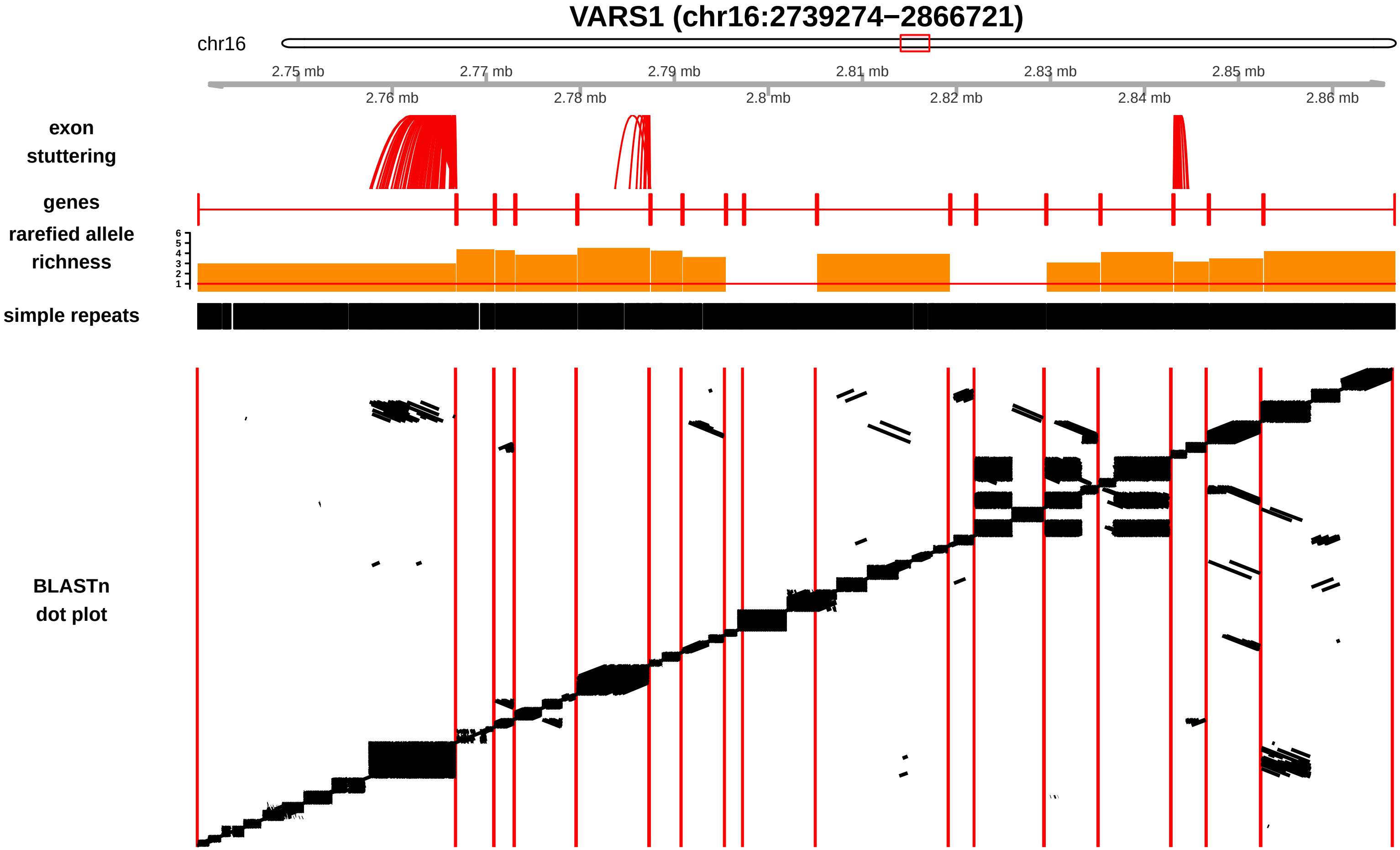

VARS1 (chr16:2739274–2866721)
chr16
2.75 mb
2.76 mb
2.77 mb
2.78 mb
2.79 mb
2.8 mb
2.81 mb
2.82 mb
2.83 mb
2.84 mb
2.85 mb
2.86 mb
exon stuttering
genes
rarefied allele richness
6
5
4
3
2
1
simple repeats
BLASTn dot plot

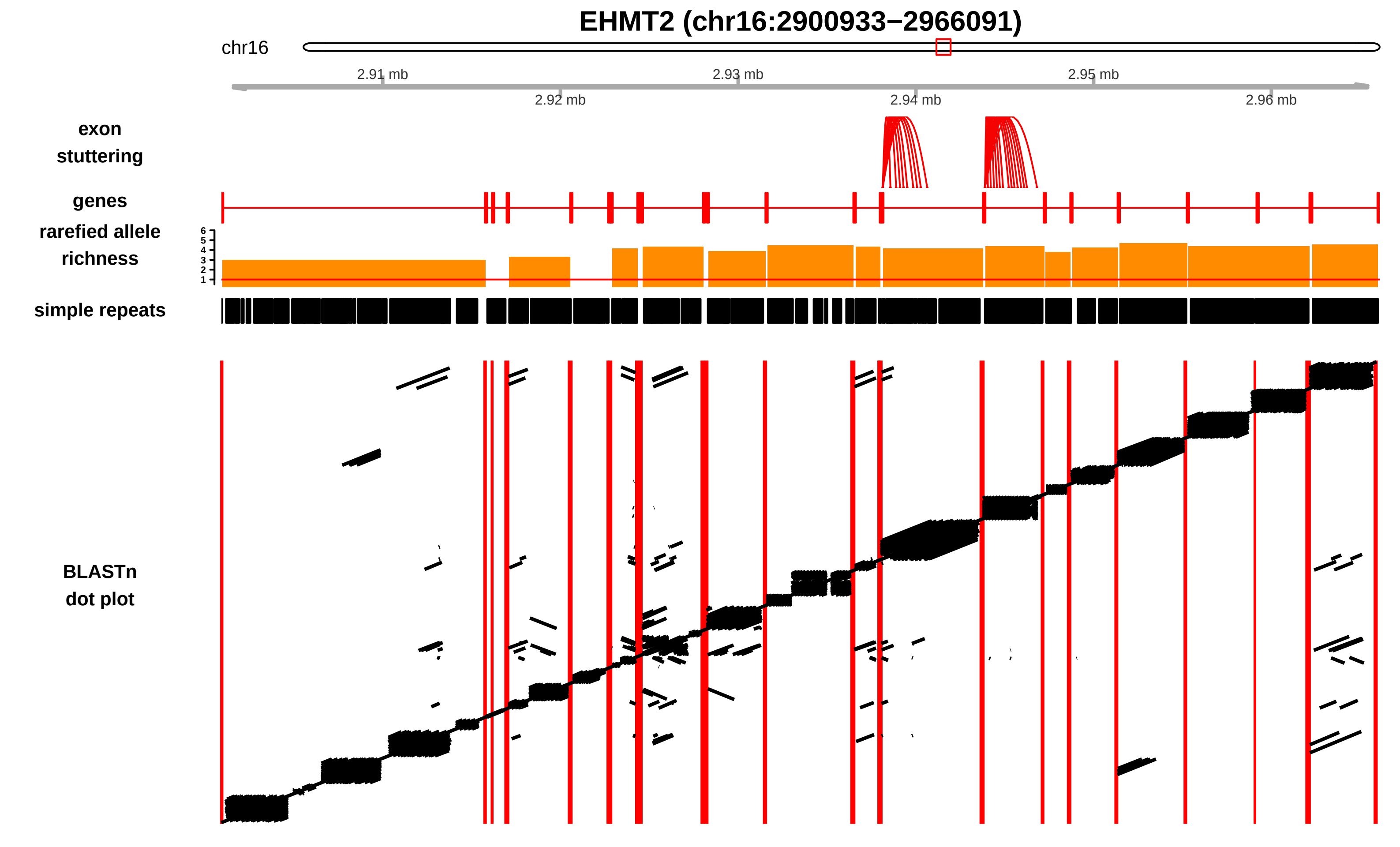
EHMT2 (chr16:2900933−2966091)
chr16
2.91 mb
2.92 mb
2.93 mb
2.94 mb
2.95 mb
2.96 mb
exon stuttering
genes
rarefied allele richness
6
5
4
3
2
1
simple repeats
BLASTn dot plot

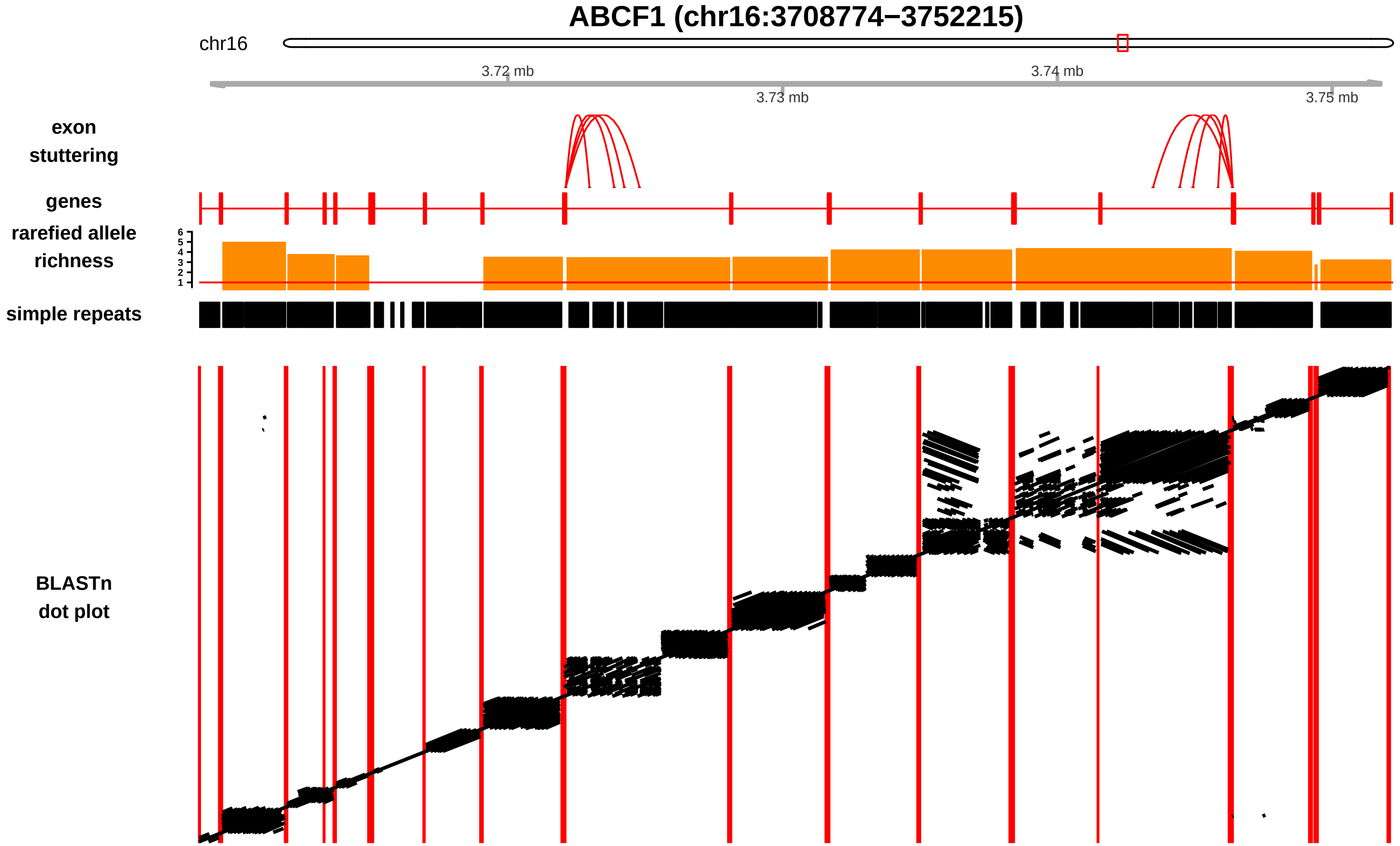

ABCF1 (chr16:3708774–3752215)
chr16
3.72 mb
3.73 mb
3.74 mb
3.75 mb
exon stuttering
genes
rarefied allele richness
1
2
3
4
5
6
simple repeats
BLASTn dot plot

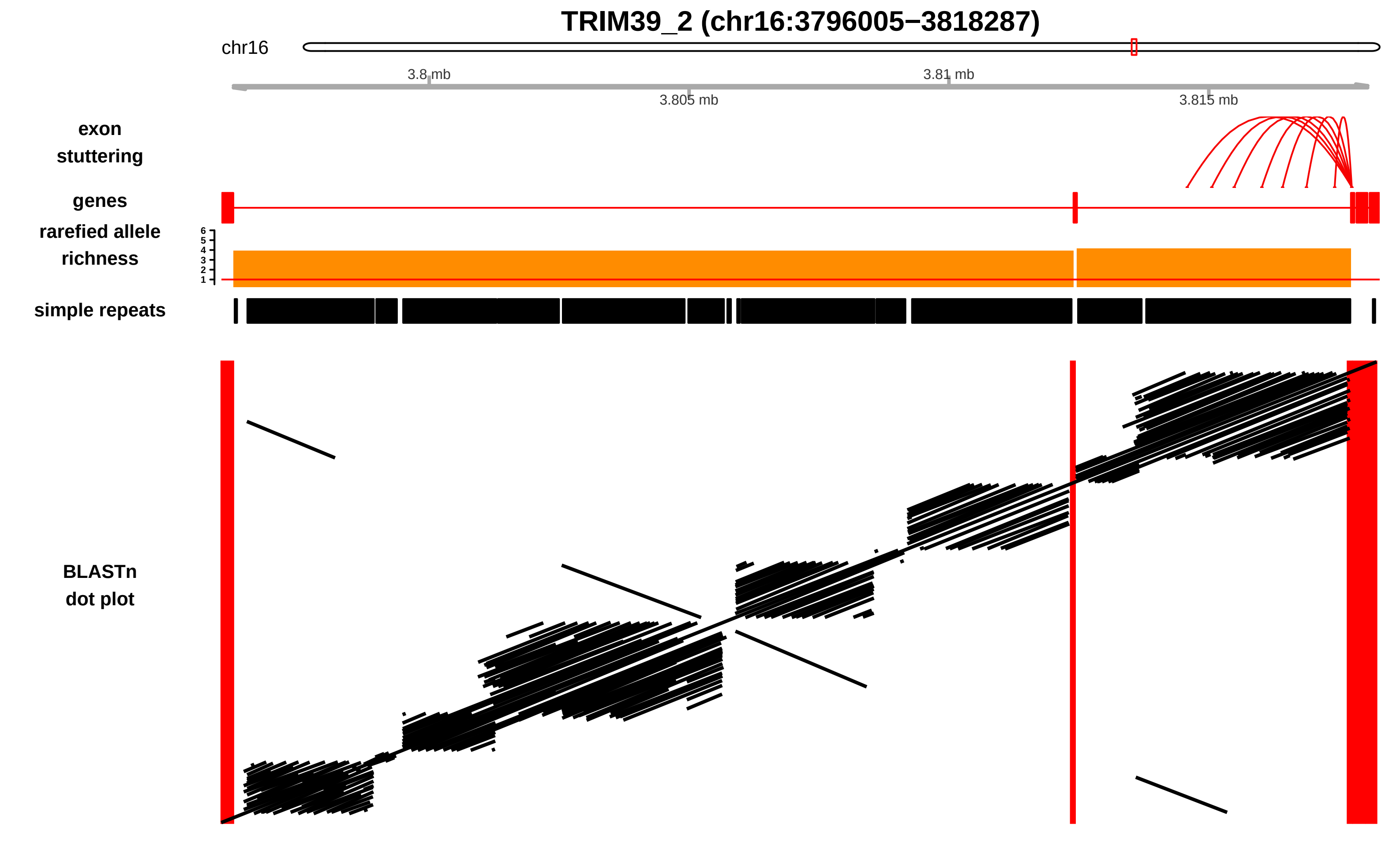

TRIM39_2 (chr16:3796005−3818287)
chr16
3.8 mb
3.81 mb
3.805 mb
3.815 mb
exon stuttering
genes
rarefied allele richness
6
5
4
3
2
1
simple repeats
BLASTn dot plot

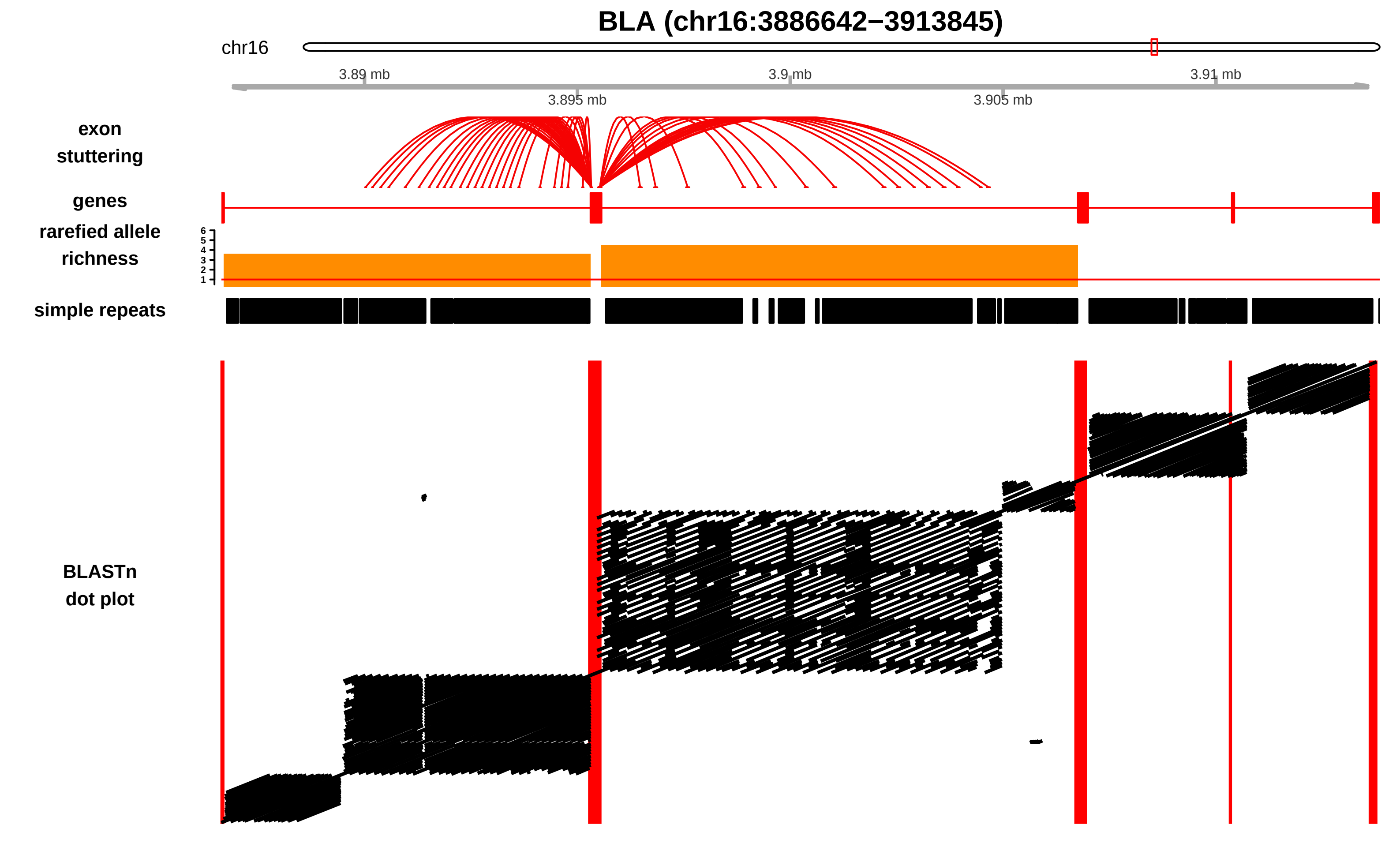
BLA (chr16:3886642−3913845)
chr16
3.89 mb
3.9 mb
3.91 mb
3.895 mb
3.905 mb
exon stuttering
genes
rarefied allele richness
6
5
4
3
2
1
simple repeats
BLASTn dot plot

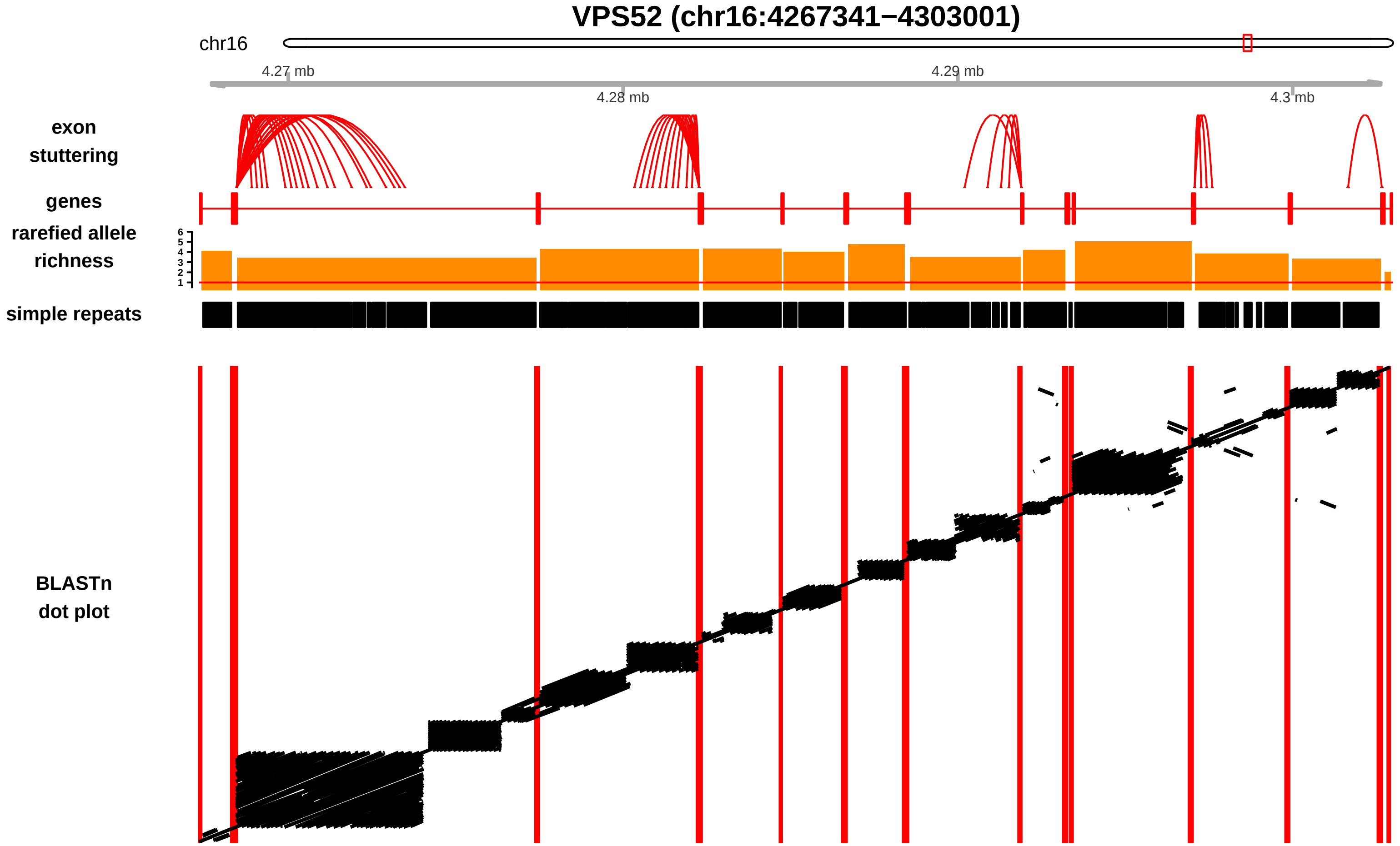
VPS52 (chr16:4267341−4303001)
chr16
4.27 mb
4.28 mb
4.29 mb
4.3 mb
exon
stuttering
genes
rarefied allele
richness
6
5
4
3
2
1
simple repeats
BLASTn
dot plot

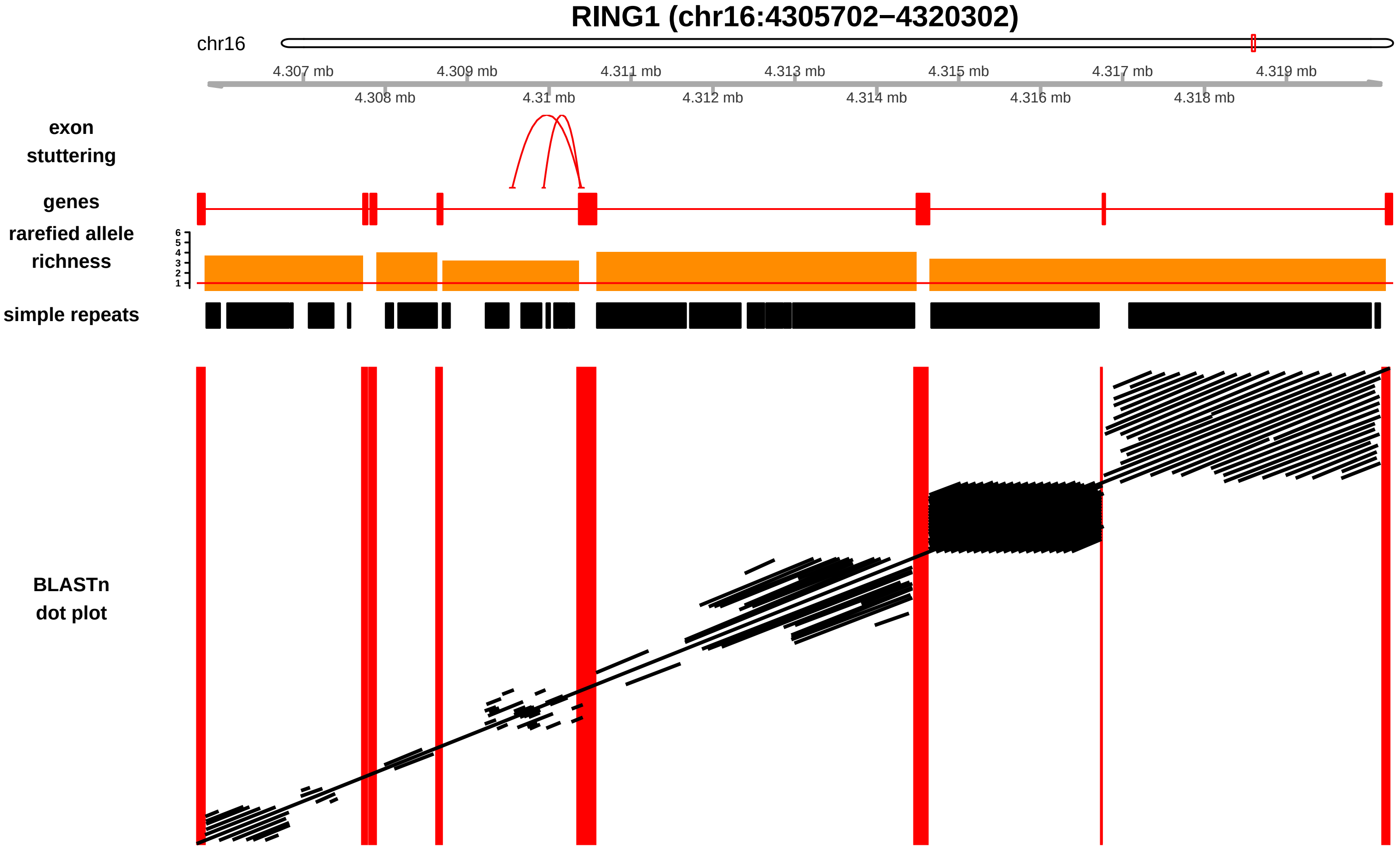
RING1 (chr16:4305702−4320302)
chr16
4.307 mb
4.309 mb
4.311 mb
4.313 mb
4.315 mb
4.317 mb
4.319 mb
4.308 mb
4.31 mb
4.312 mb
4.314 mb
4.316 mb
4.318 mb
exon stuttering
genes
rarefied allele richness
6
5
4
3
2
1
simple repeats
BLASTn dot plot

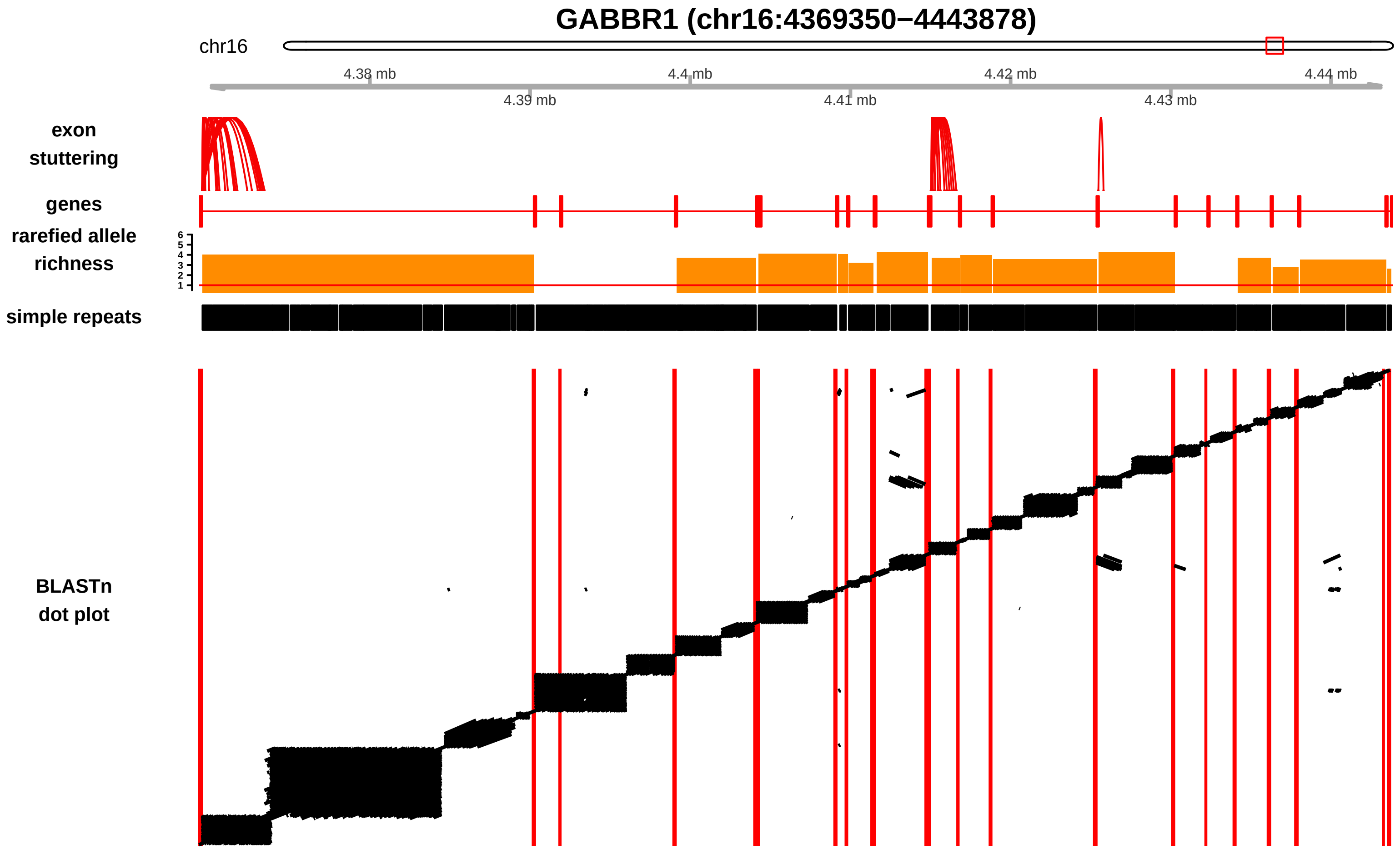

GABBR1 (chr16:4369350−4443878)
chr16
4.38 mb
4.39 mb
4.4 mb
4.41 mb
4.42 mb
4.43 mb
4.44 mb
exon stuttering
genes
rarefied allele richness
6
5
4
3
2
1
simple repeats
BLASTn dot plot

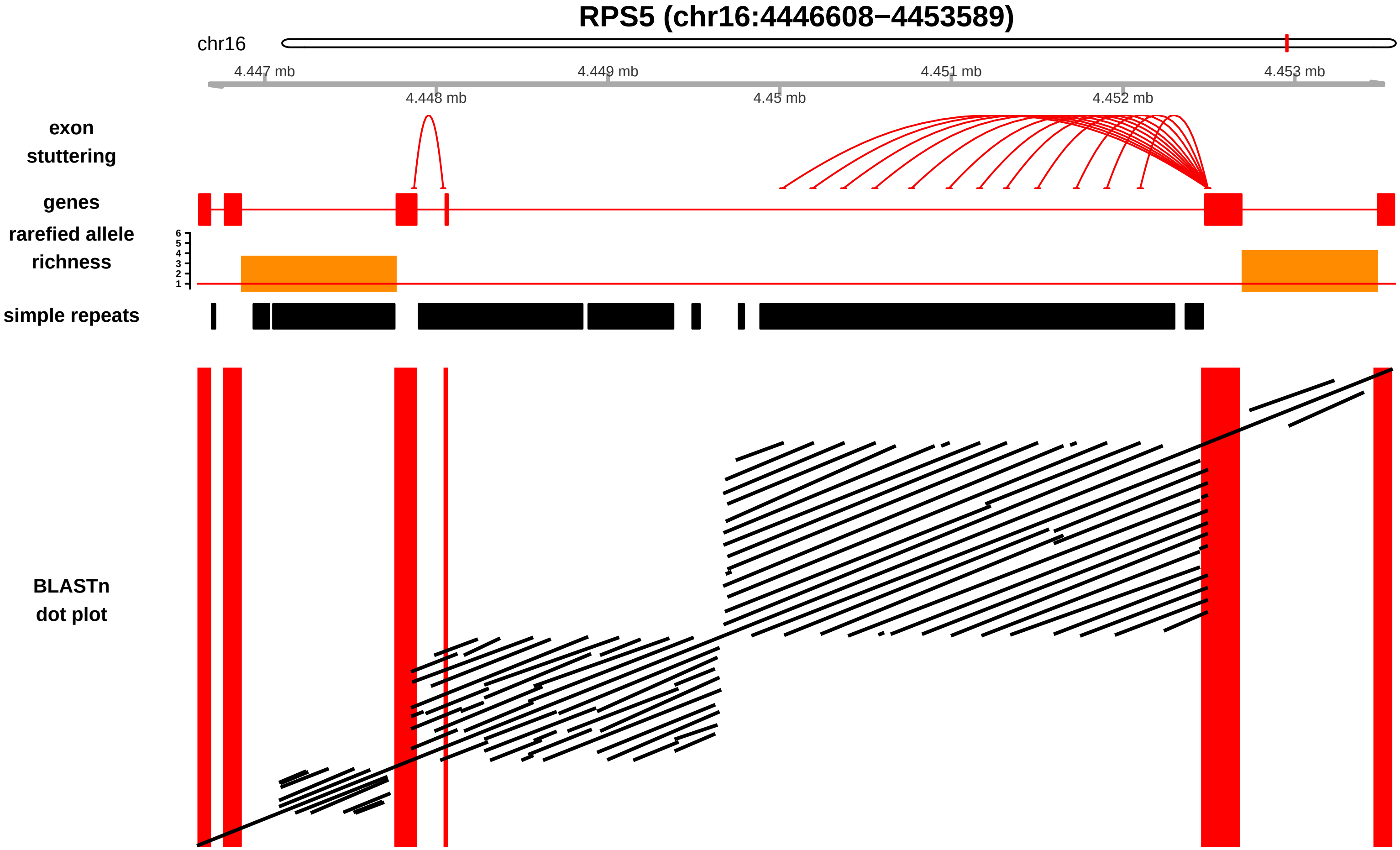
RPS5 (chr16:4446608−4453589)
chr16
4.447 mb
4.448 mb
4.449 mb
4.45 mb
4.451 mb
4.452 mb
4.453 mb
exon stuttering
genes
rarefied allele richness
6
5
4
3
2
1
simple repeats
BLASTn dot plot

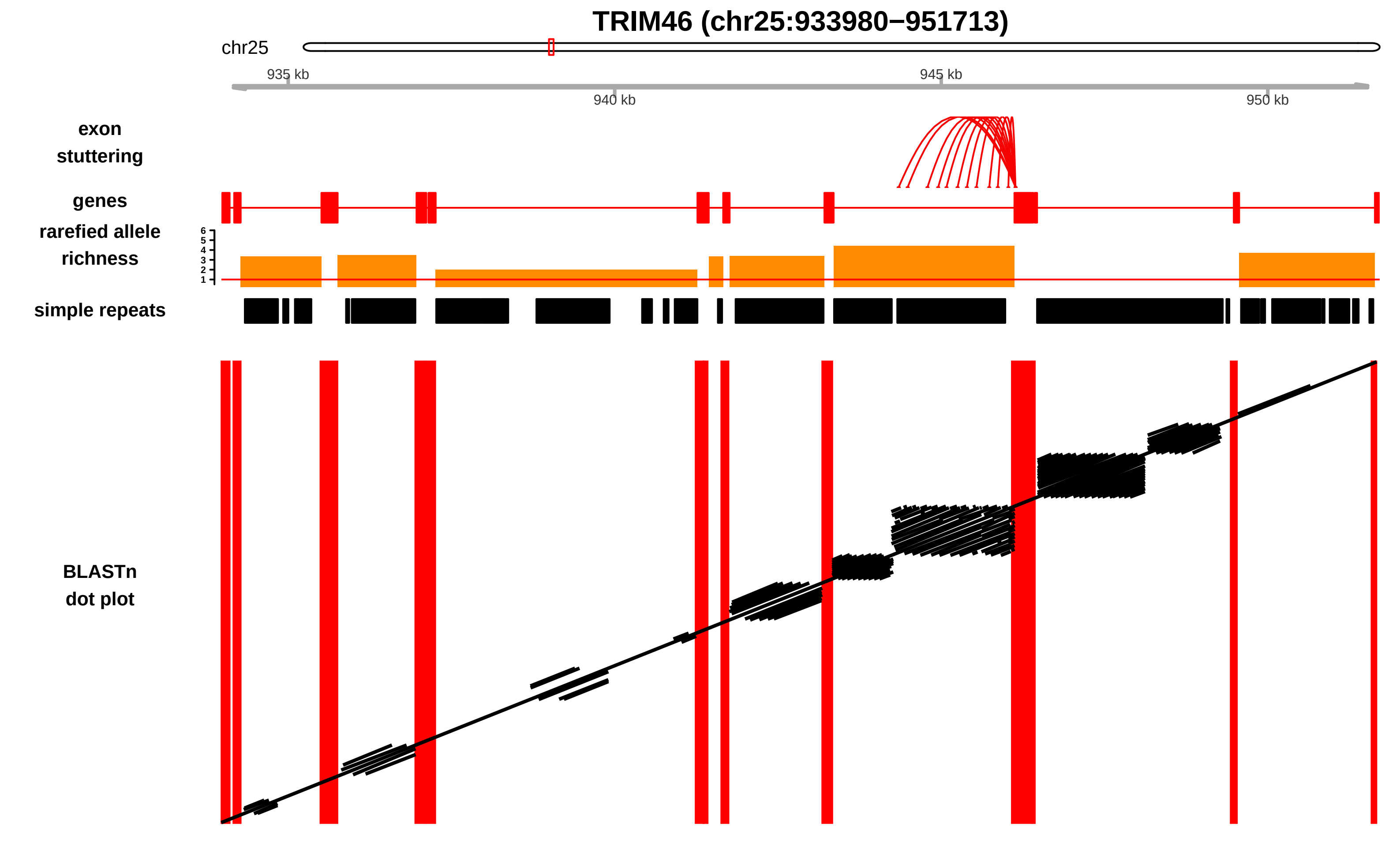
TRIM46 (chr25:933980−951713)
chr25
935 kb
940 kb
945 kb
950 kb
exon stuttering
genes
rarefied allele richness
1
2
3
4
5
6
simple repeats
BLASTn dot plot

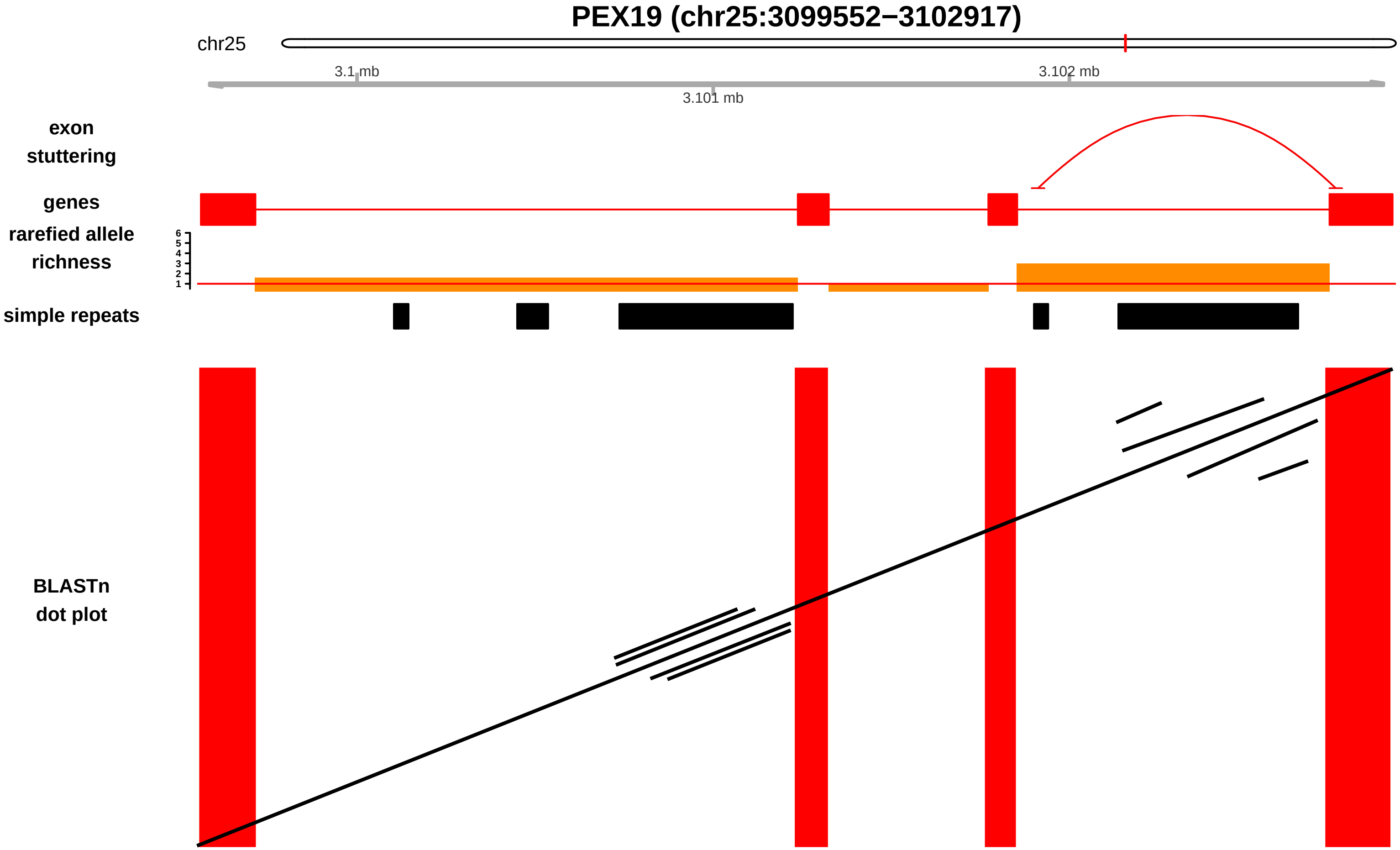

PEX19 (chr25:3099552−3102917)
chr25
3.1 mb
3.101 mb
3.102 mb
exon
stuttering
genes
rarefied allele
richness
6
5
4
3
2
1
simple repeats
BLASTn
dot plot

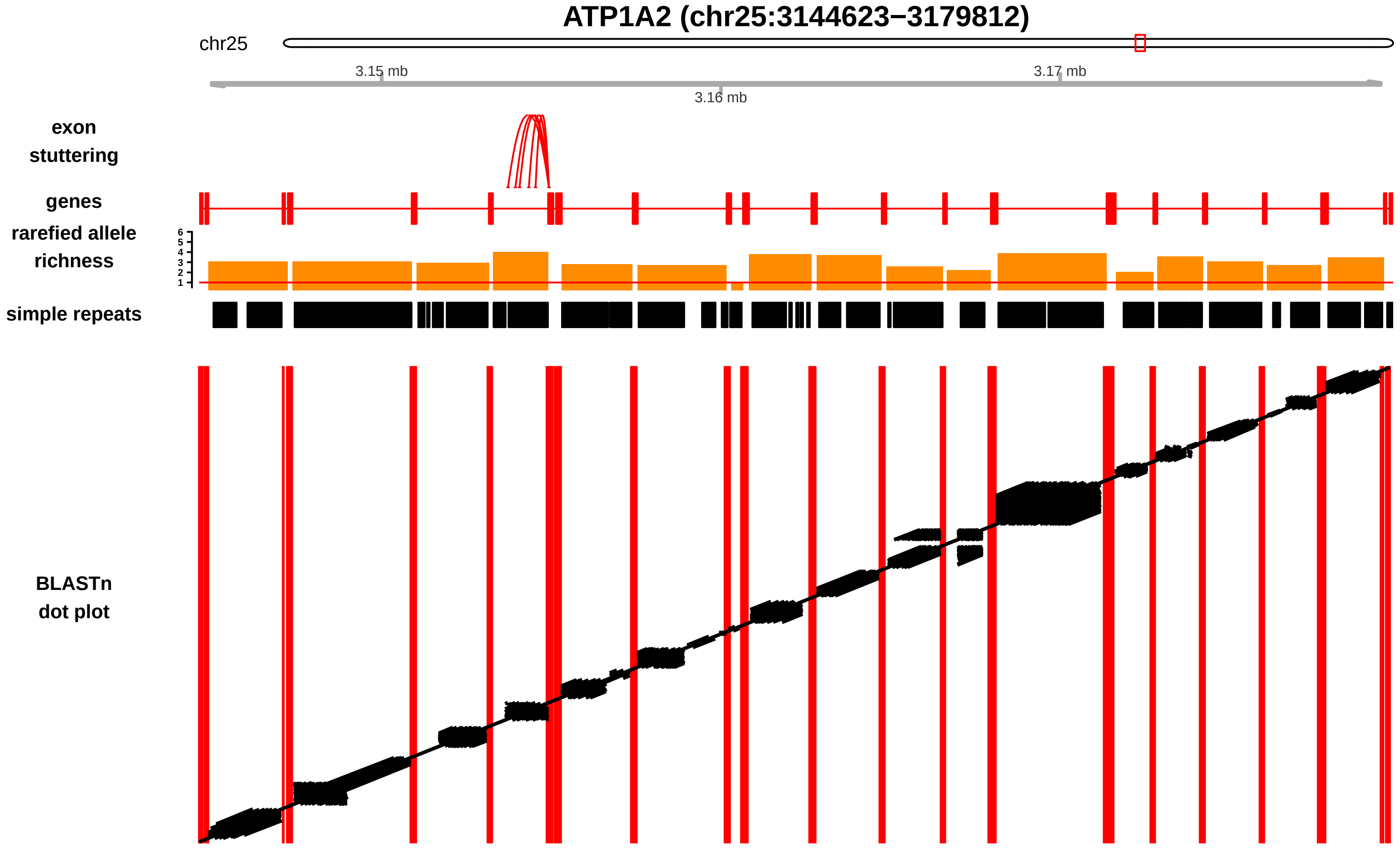
ATP1A2 (chr25:3144623−3179812)
chr25
3.15 mb
3.16 mb
3.17 mb
exon stuttering
genes
rarefied allele richness
6
5
4
3
2
1
simple repeats
BLASTn dot plot

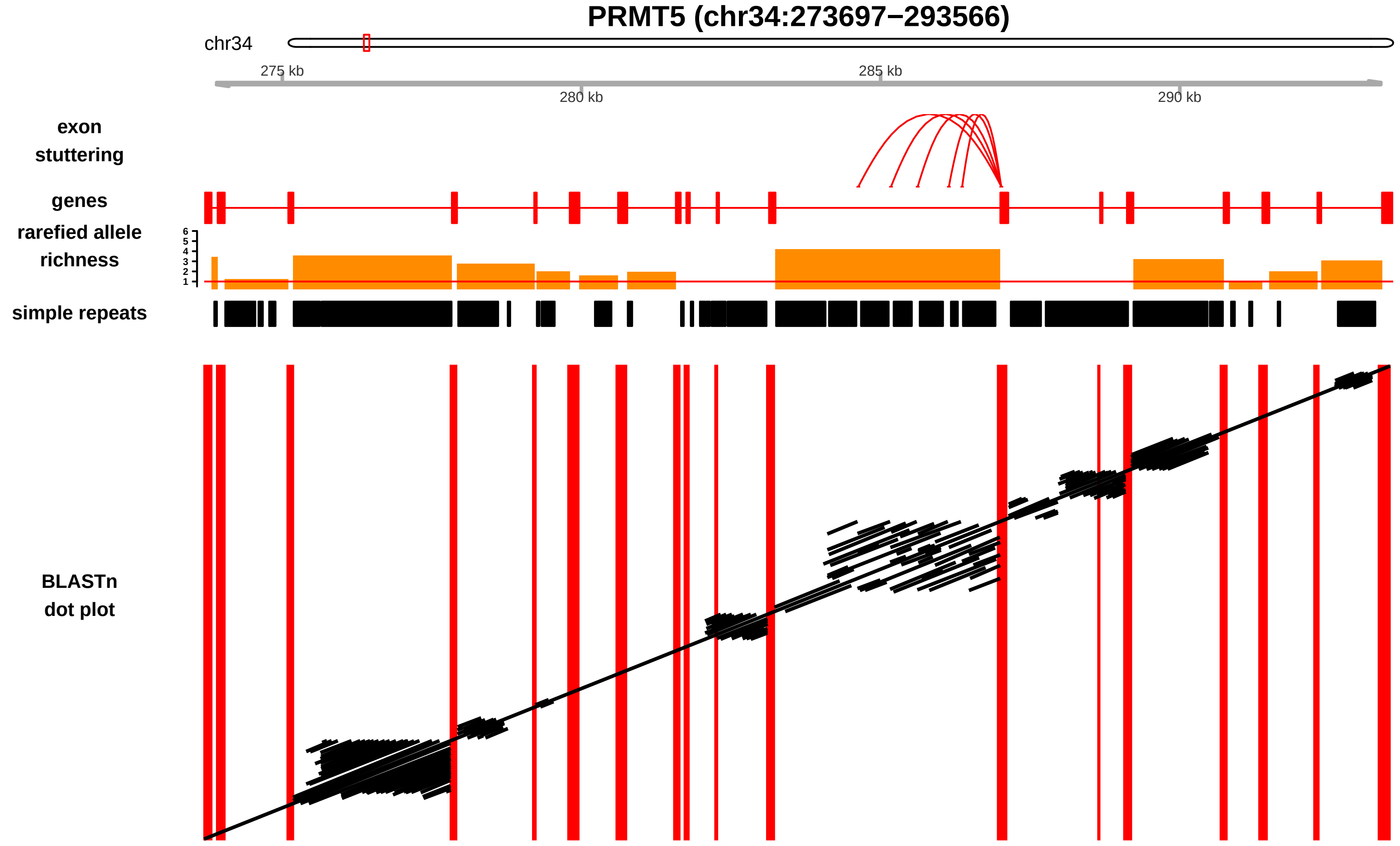
PRMT5 (chr34:273697−293566)
chr34
275 kb
280 kb
285 kb
290 kb
exon stuttering
genes
rarefied allele richness
1
2
3
4
5
6
simple repeats
BLASTn dot plot

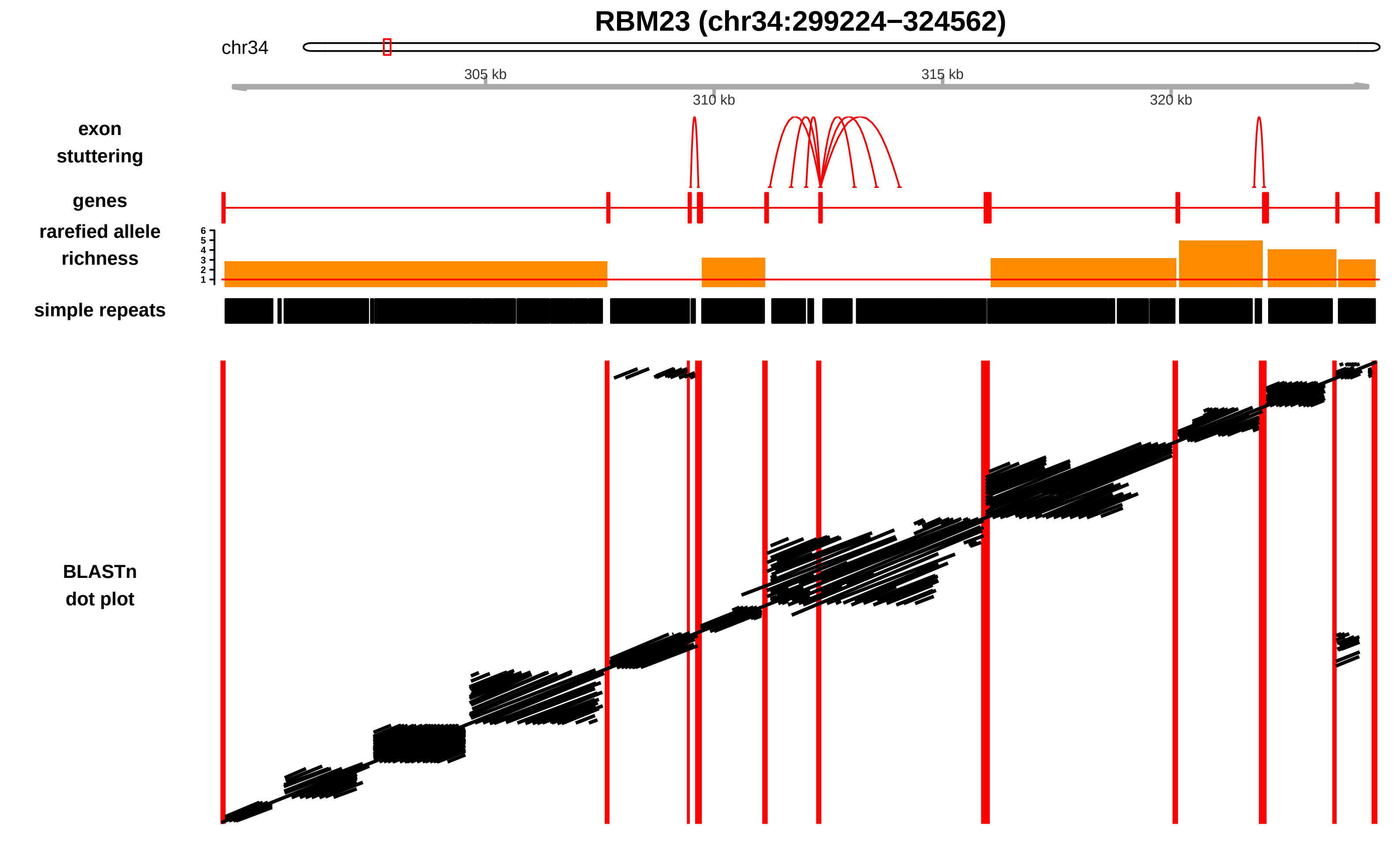

RBM23 (chr34:299224−324562)
chr34
305 kb
310 kb
315 kb
320 kb
exon stuttering
genes
rarefied allele richness
1
2
3
4
5
6
simple repeats
BLASTn dot plot

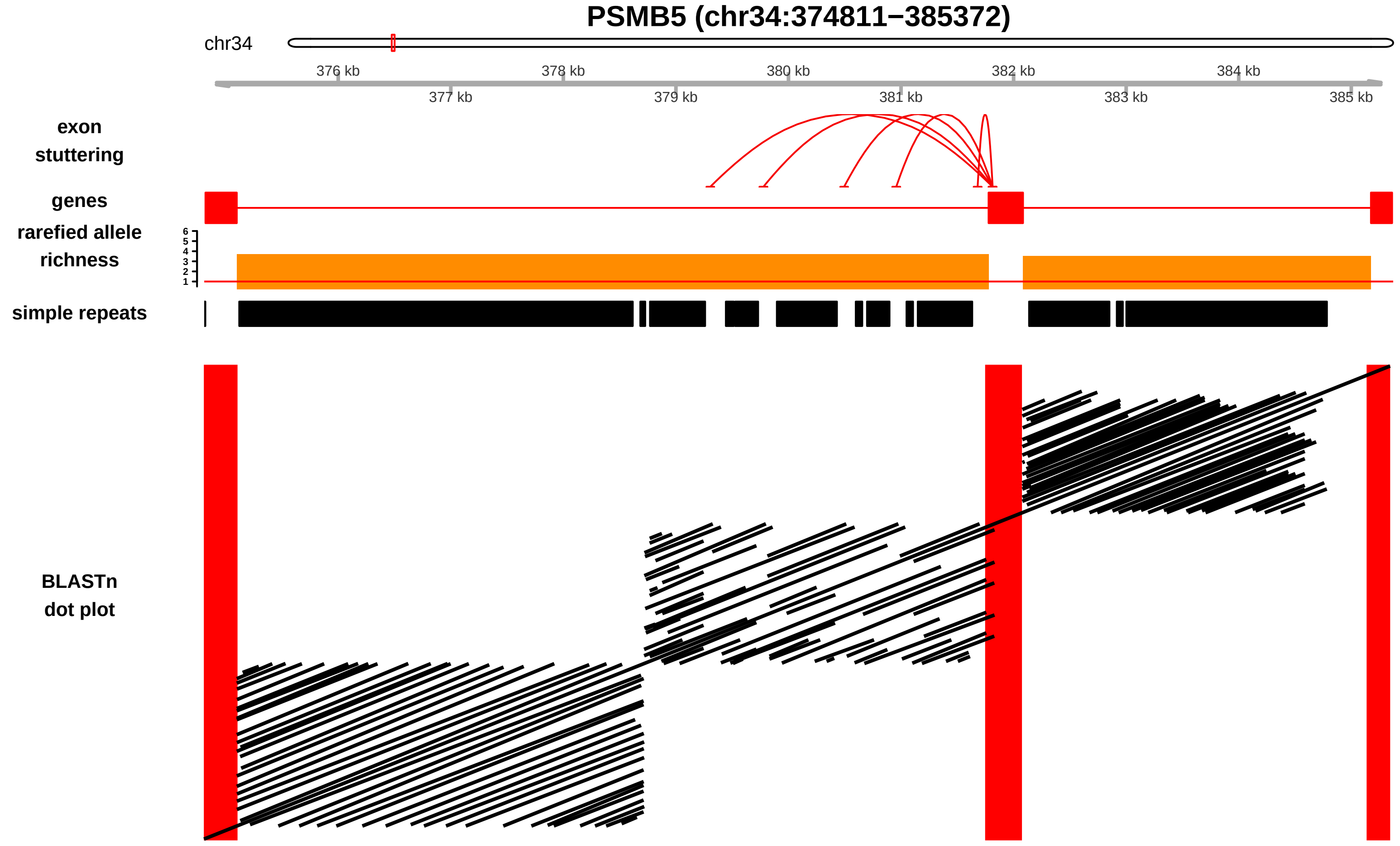

PSMB5 (chr34:374811−385372)
chr34
376 kb
378 kb
380 kb
382 kb
384 kb
377 kb
379 kb
381 kb
383 kb
385 kb
exon stuttering
genes
rarefied allele richness
6
5
4
3
2
1
simple repeats
BLASTn dot plot

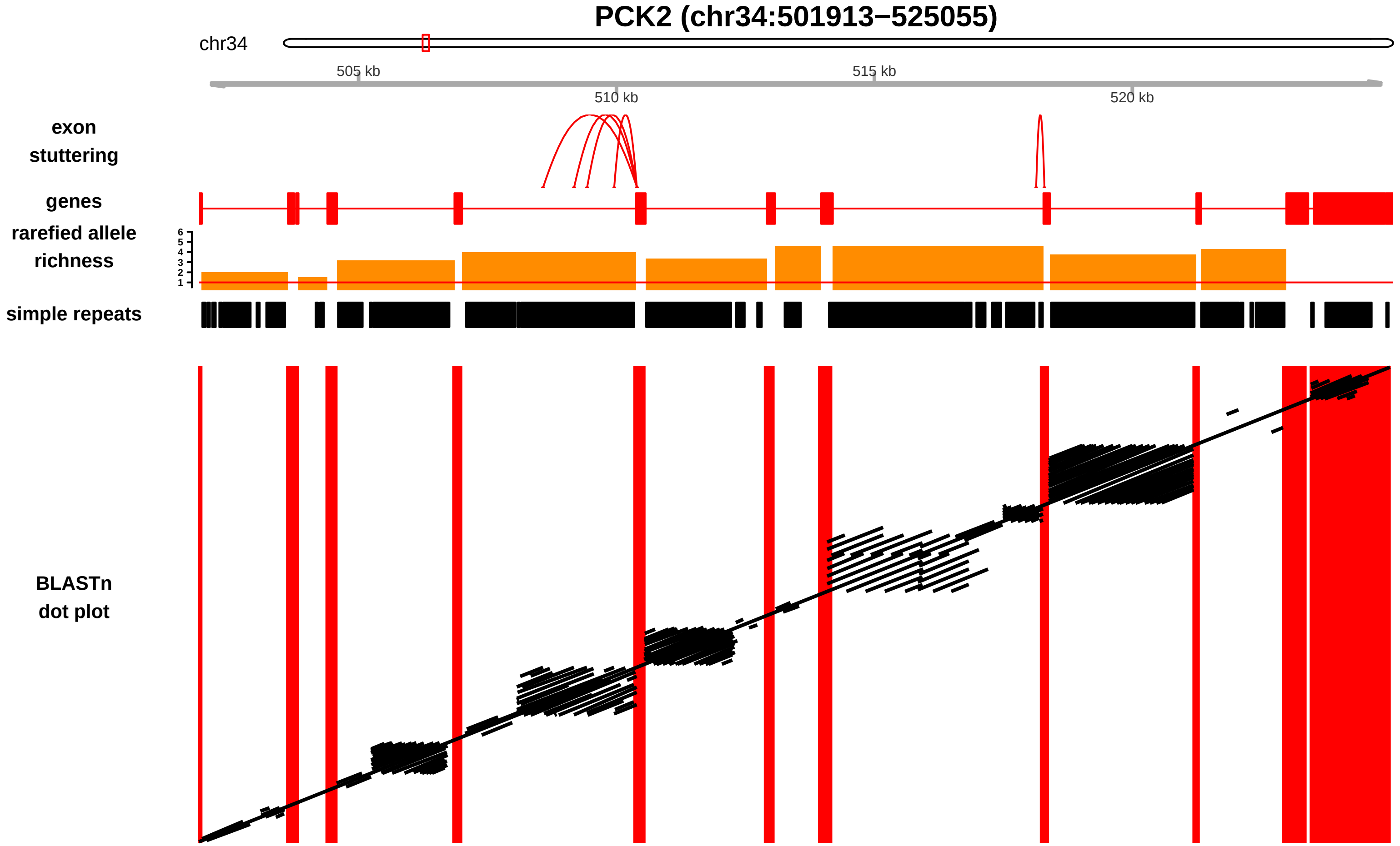

PCK2 (chr34:501913–525055)
chr34
505 kb
510 kb
515 kb
520 kb
exon stuttering
genes
rarefied allele richness
6
5
4
3
2
1
simple repeats
BLASTn dot plot

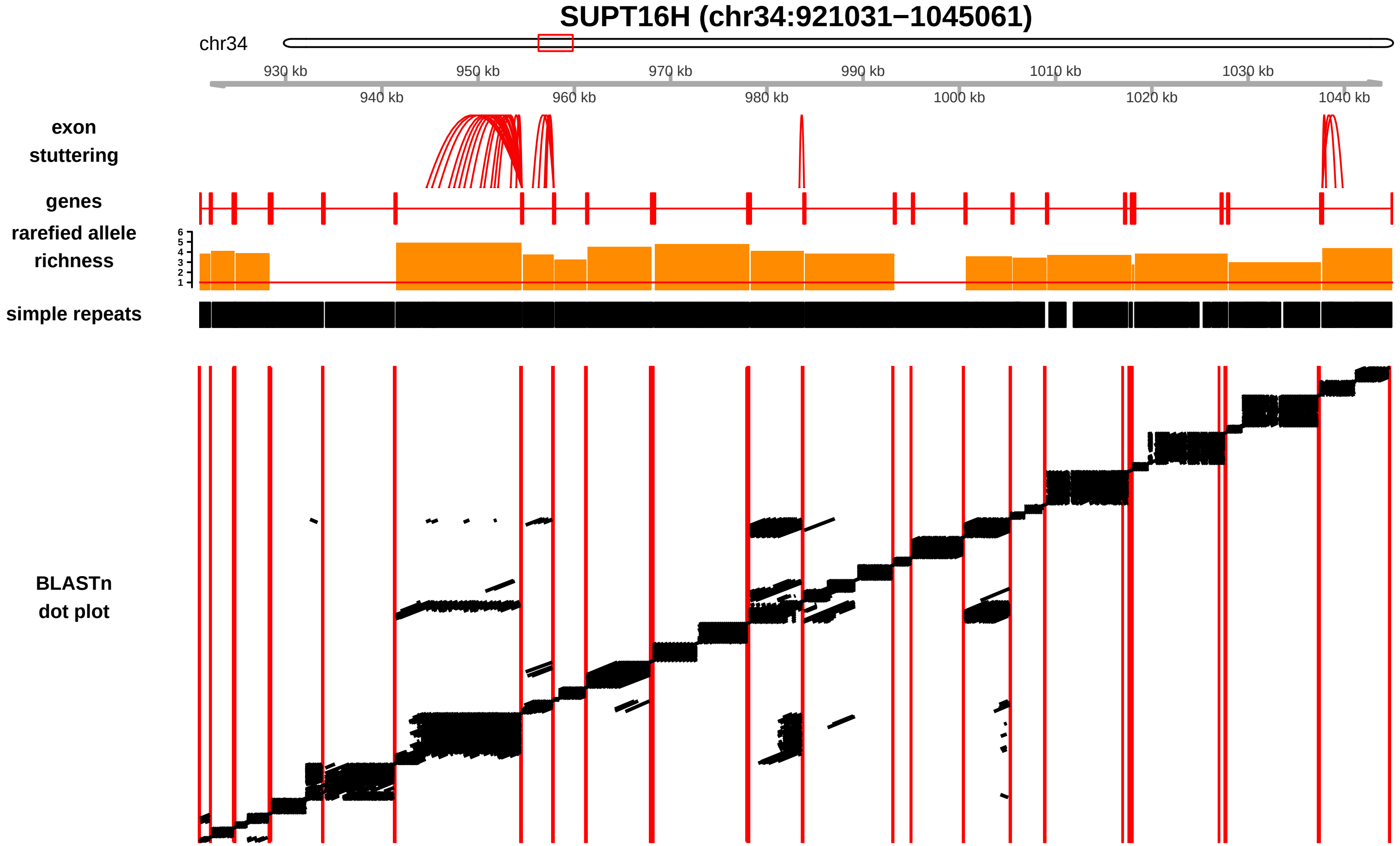

SUPT16H (chr34:921031−1045061)
chr34
930 kb
940 kb
950 kb
960 kb
970 kb
980 kb
990 kb
1000 kb
1010 kb
1020 kb
1030 kb
1040 kb
exon stuttering
genes
rarefied allele richness
1
2
3
4
5
6
simple repeats
BLASTn dot plot

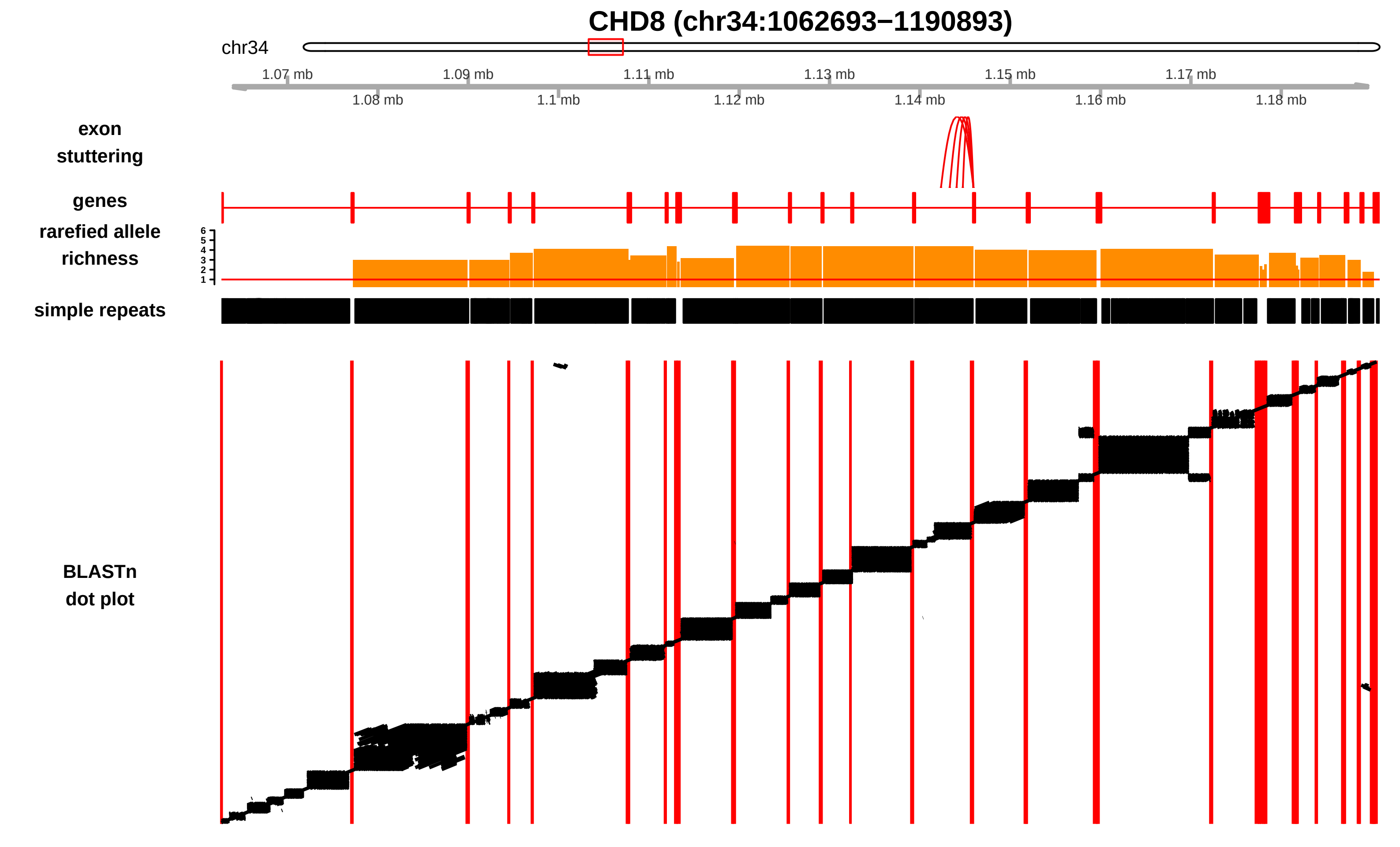

CHD8 (chr34:1062693−1190893)
chr34
1.07 mb
1.08 mb
1.09 mb
1.1 mb
1.11 mb
1.12 mb
1.13 mb
1.14 mb
1.15 mb
1.16 mb
1.17 mb
1.18 mb
exon stuttering
genes
rarefied allele richness
6
5
4
3
2
1
simple repeats
BLASTn dot plot

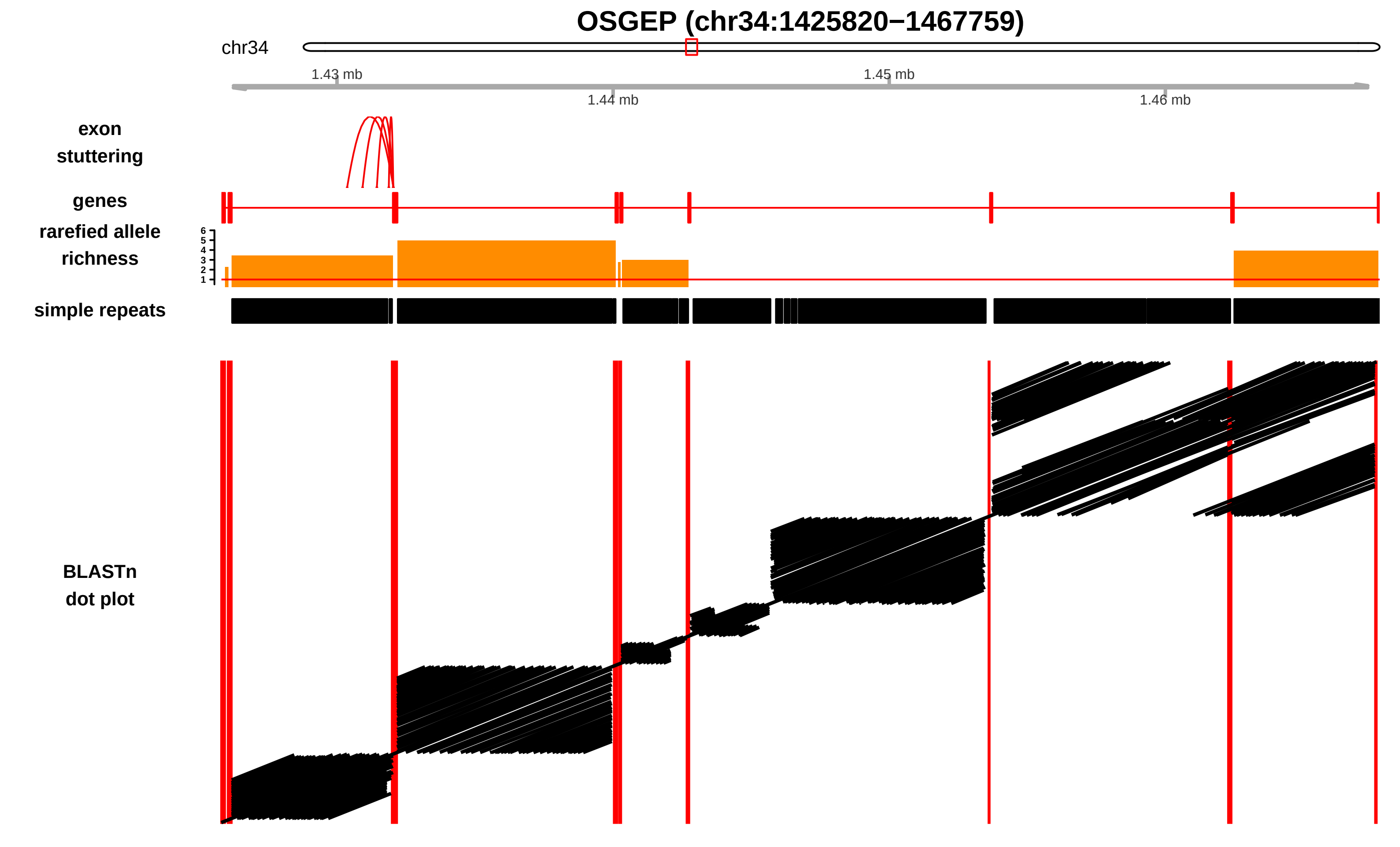

OSGEP (chr34:1425820−1467759)
chr34
1.43 mb
1.44 mb
1.45 mb
1.46 mb
exon stuttering
genes
rarefied allele richness
6
5
4
3
2
1
simple repeats
BLASTn dot plot

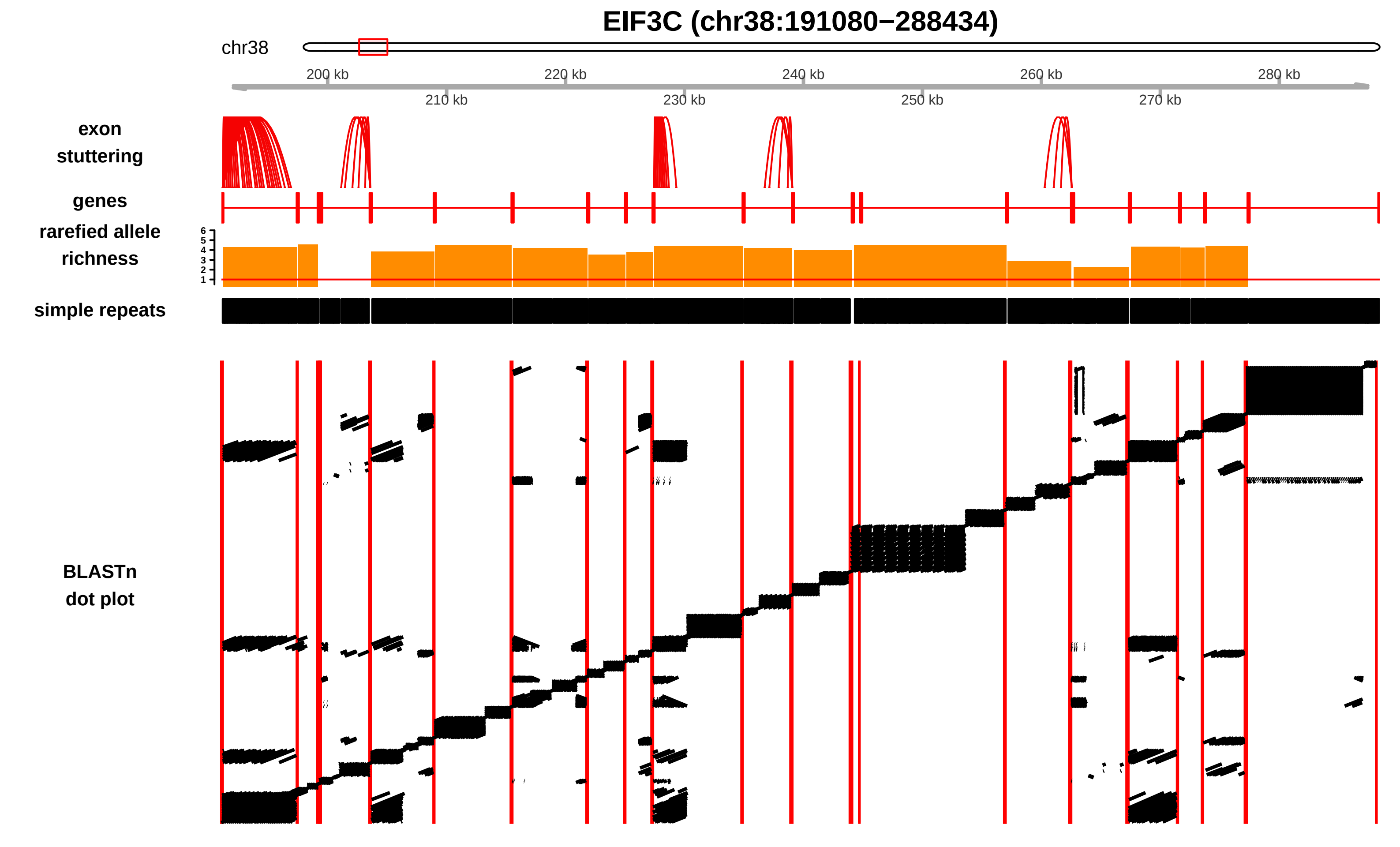
EIF3C (chr38:191080−288434)
chr38
200 kb
210 kb
220 kb
230 kb
240 kb
250 kb
260 kb
270 kb
280 kb
exon stuttering
genes
rarefied allele richness
6
5
4
3
2
1
simple repeats
BLASTn dot plot

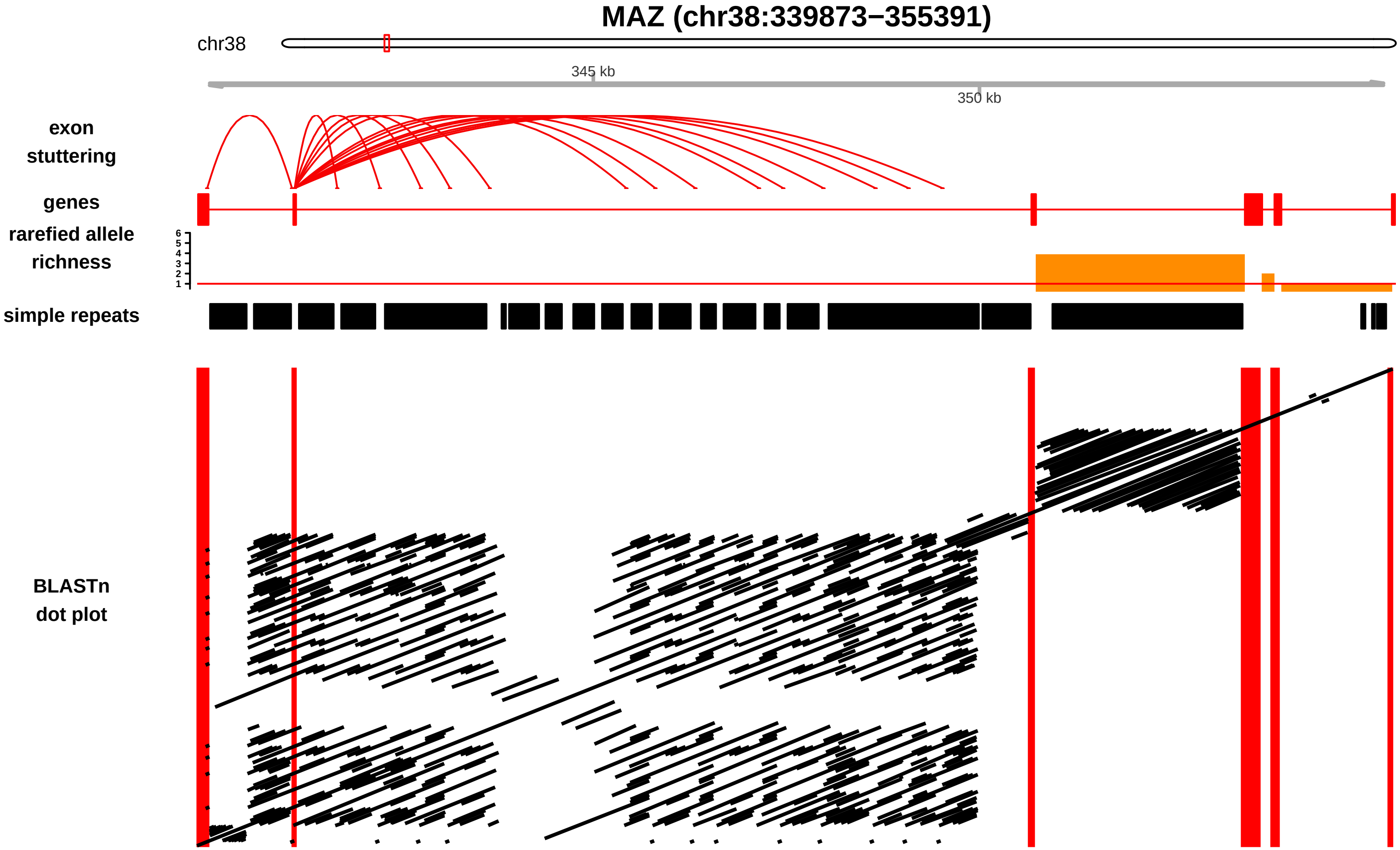
MAZ (chr38:339873−355391)
chr38
345 kb
350 kb
exon stuttering
genes
rarefied allele richness
6
5
4
3
2
1
simple repeats
BLASTn dot plot

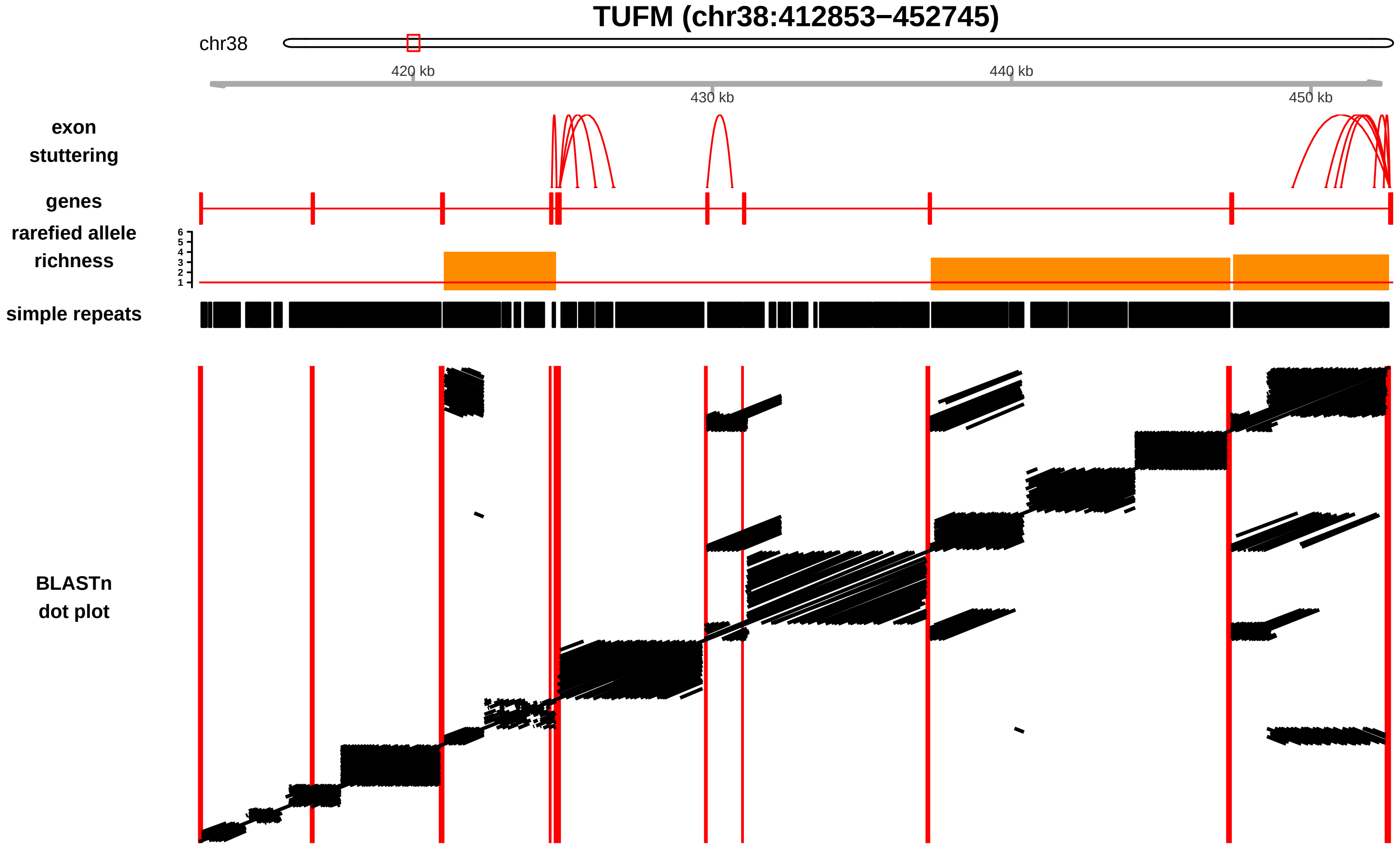
TUFM (chr38:412853−452745)
chr38
420 kb
430 kb
440 kb
450 kb
exon stuttering
genes
rarefied allele richness
1
2
3
4
5
6
simple repeats
BLASTn dot plot

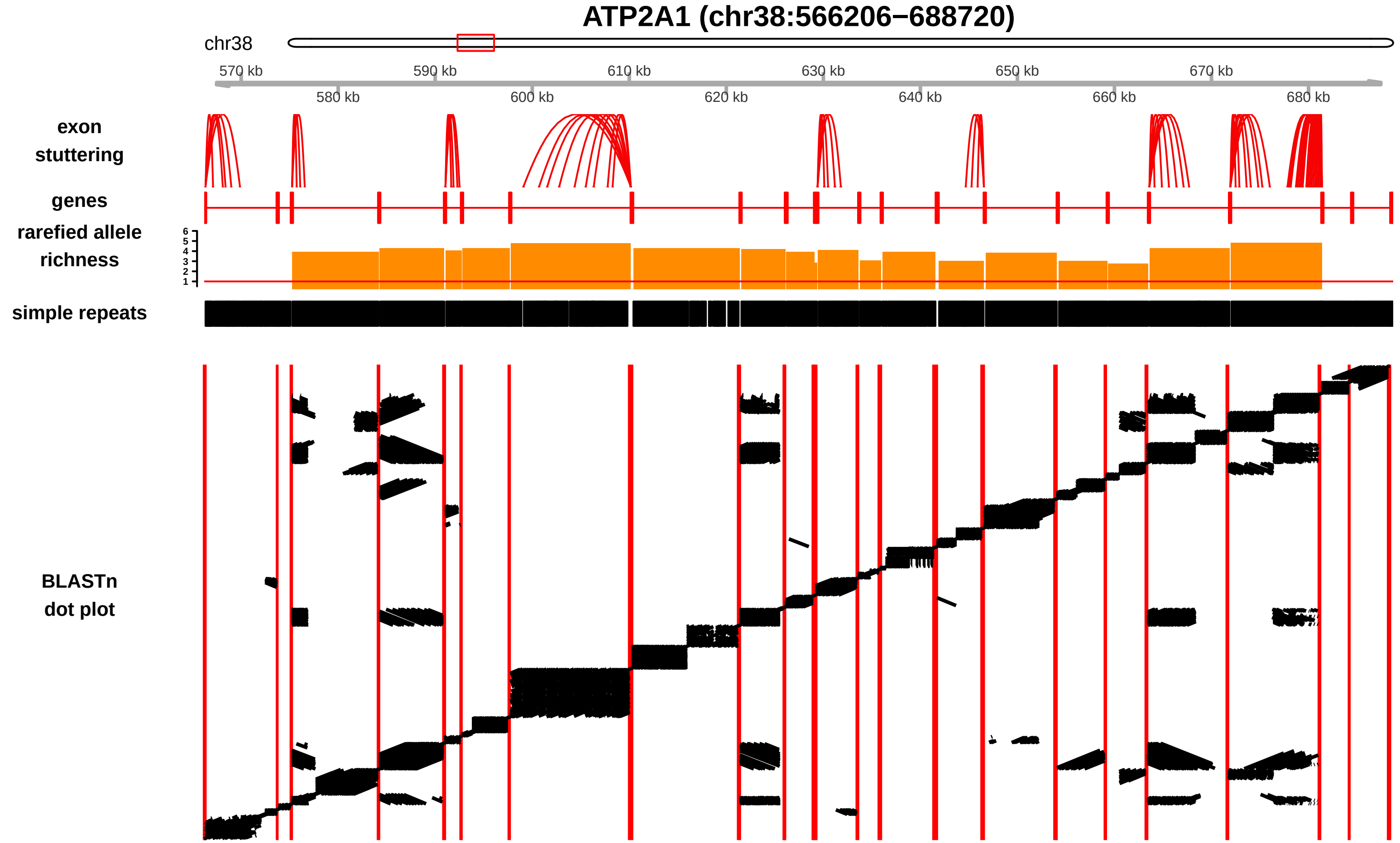

ATP2A1 (chr38:566206−688720)
chr38
570 kb
580 kb
590 kb
600 kb
610 kb
620 kb
630 kb
640 kb
650 kb
660 kb
670 kb
680 kb
exon stuttering
genes
rarefied allele richness
1
2
3
4
5
6
simple repeats
BLASTn dot plot

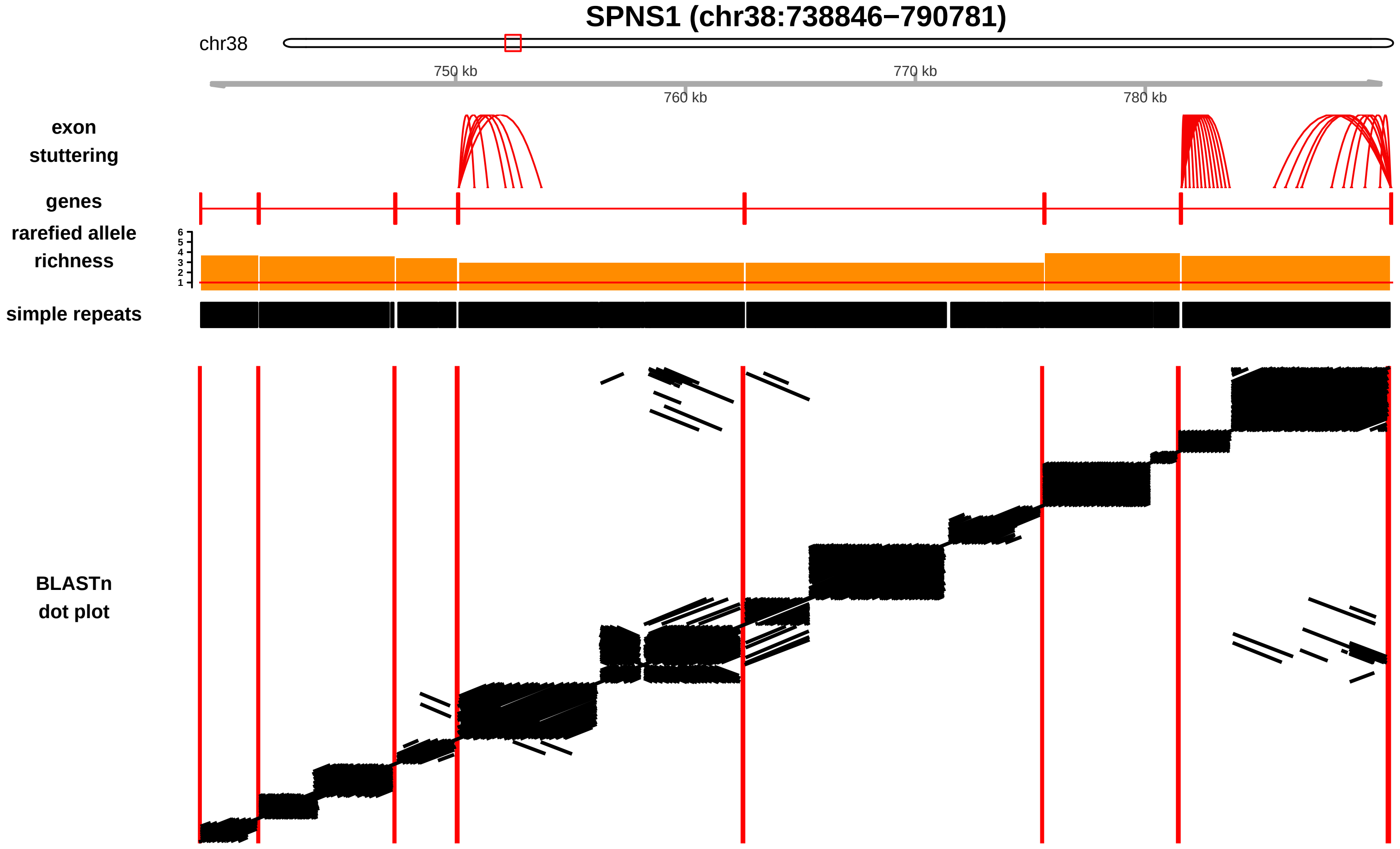
SPNS1 (chr38:738846−790781)
chr38
750 kb
770 kb
760 kb
780 kb
exon stuttering
genes
rarefied allele richness
6
5
4
3
2
1
simple repeats
BLASTn dot plot

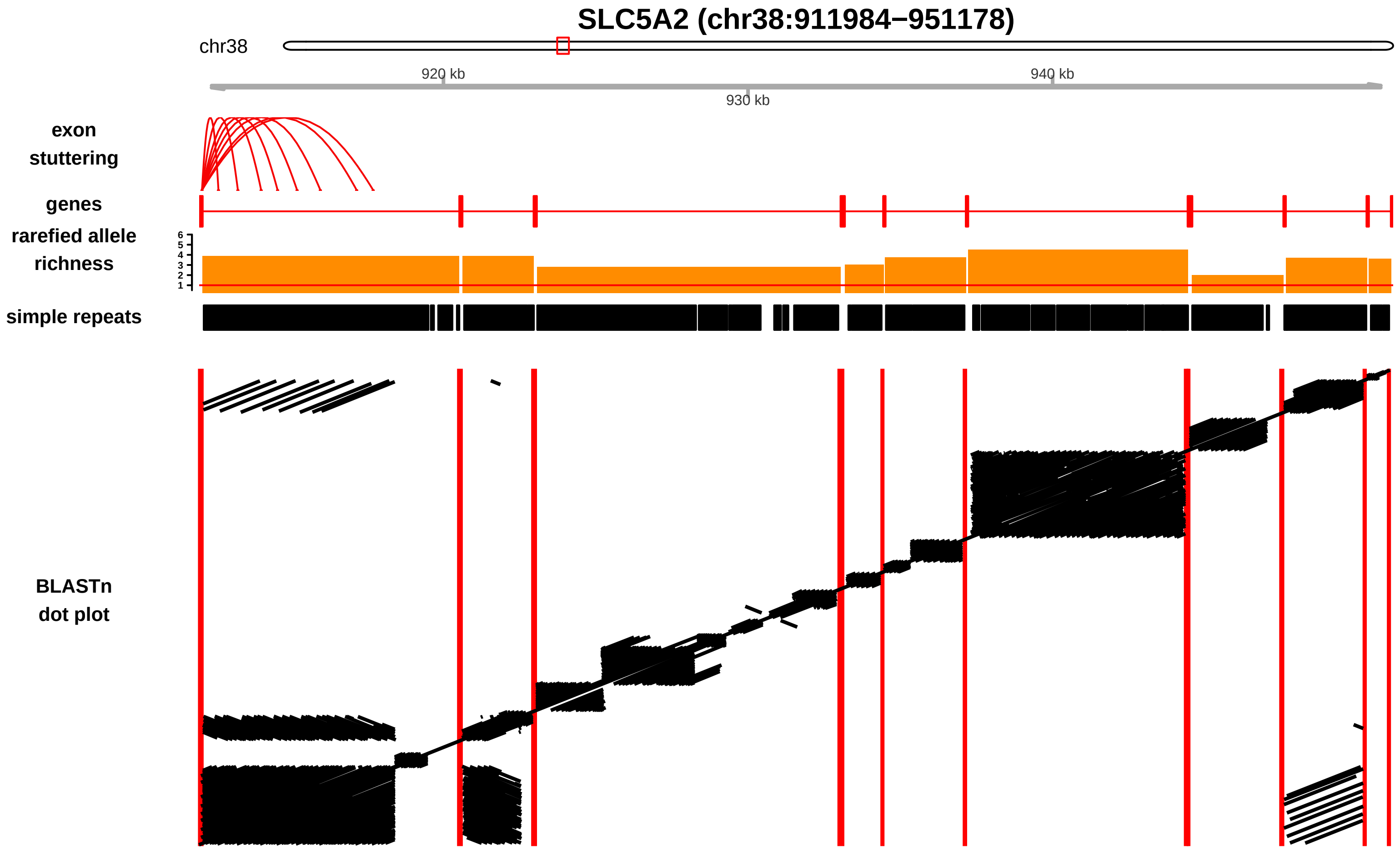
SLC5A2 (chr38:911984−951178)
chr38
920 kb
930 kb
940 kb
exon
stuttering
genes
rarefied allele
richness
6
5
4
3
2
1
simple repeats
BLASTn
dot plot

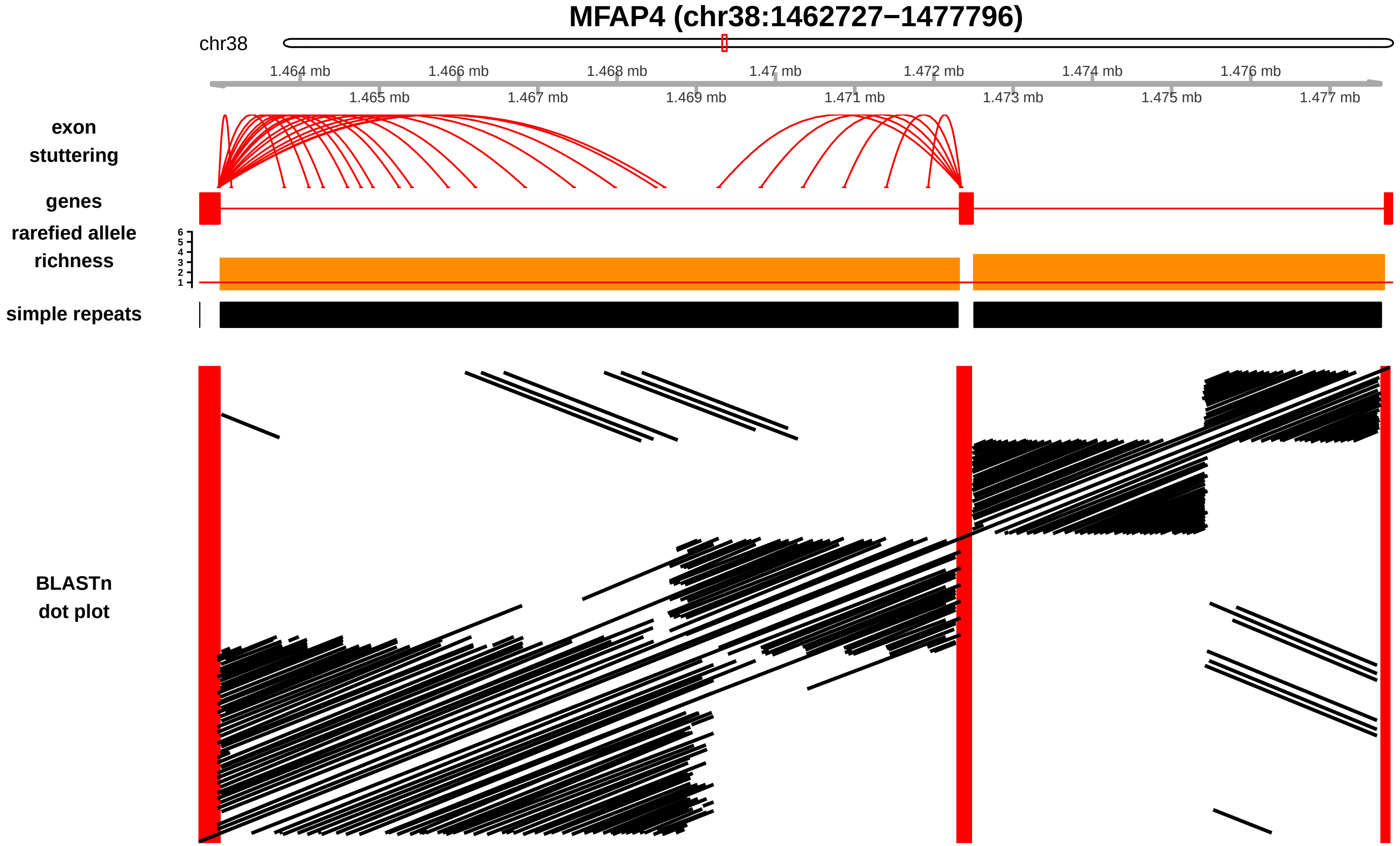

MFAP4 (chr38:1462727−1477796)
chr38
1.464 mb
1.466 mb
1.468 mb
1.47 mb
1.472 mb
1.474 mb
1.476 mb
1.465 mb
1.467 mb
1.469 mb
1.471 mb
1.473 mb
1.475 mb
1.477 mb
exon stuttering
genes
rarefied allele richness
6
5
4
3
2
1
simple repeats
BLASTn dot plot

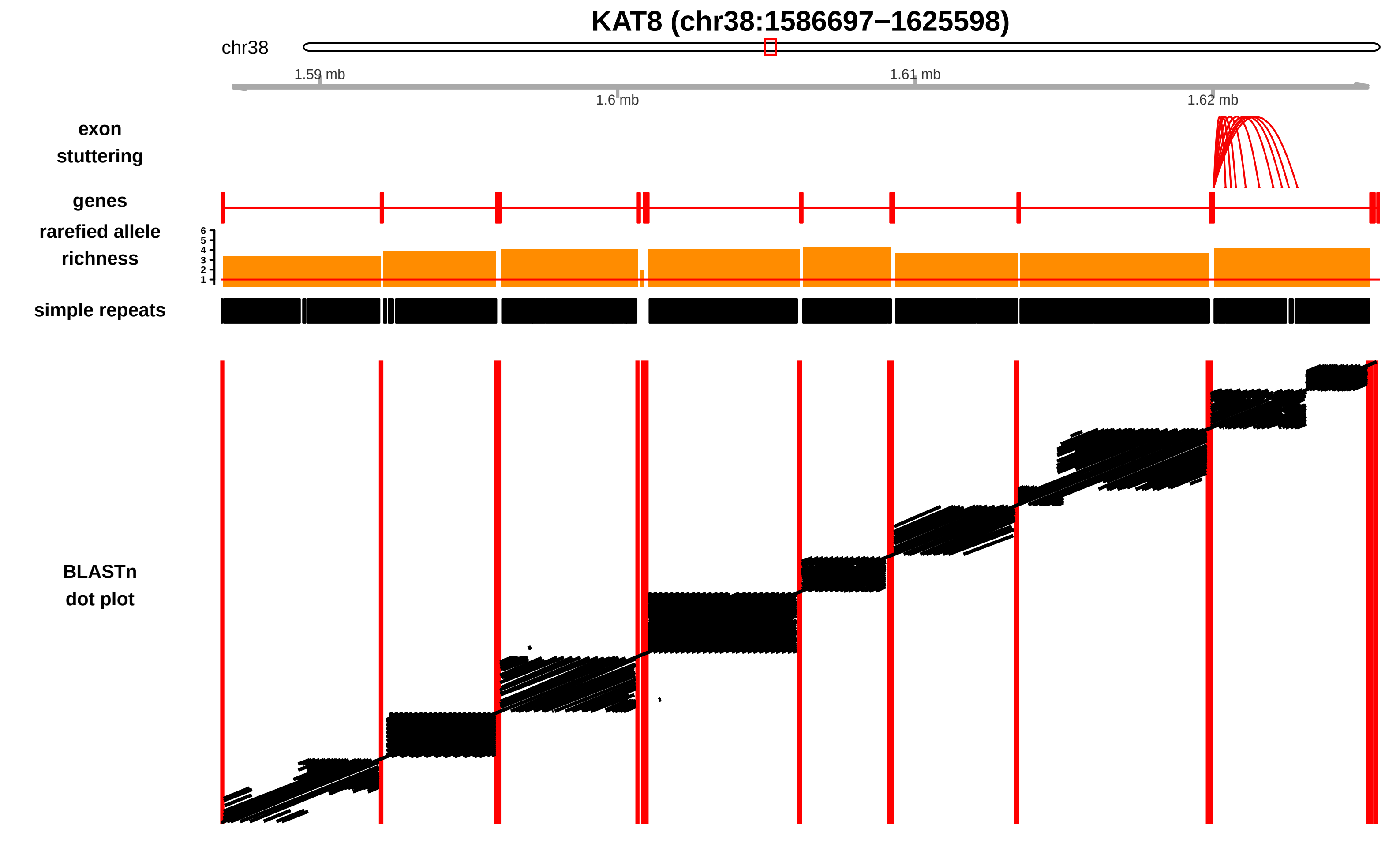

KAT8 (chr38:1586697−1625598)
chr38
1.59 mb
1.6 mb
1.61 mb
1.62 mb
exon stuttering
genes
rarefied allele richness
1
2
3
4
5
6
simple repeats
BLASTn dot plot

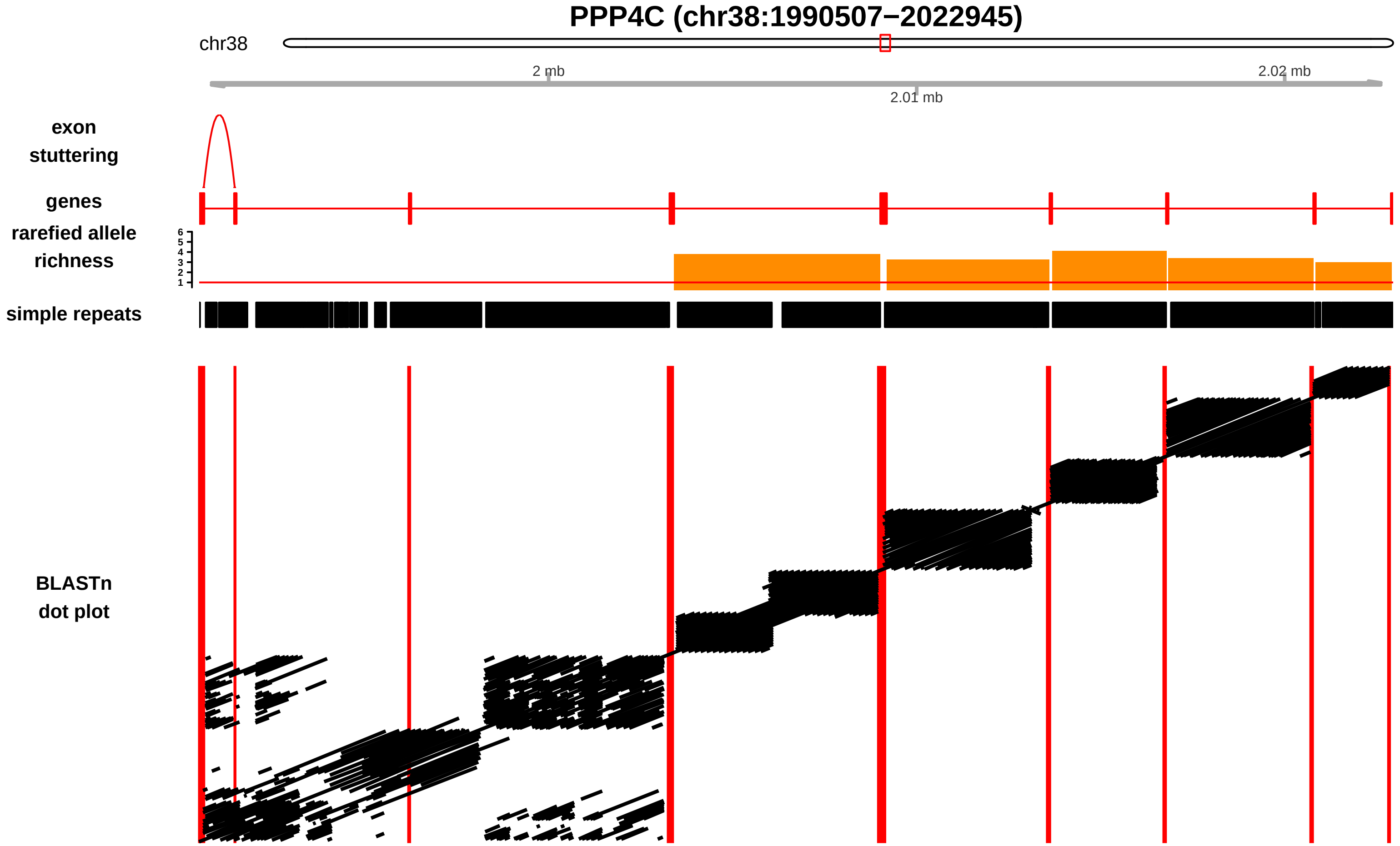

PPP4C (chr38:1990507−2022945)
chr38
2 mb
2.01 mb
2.02 mb
exon
stuttering
genes
rarefied allele
richness
6
5
4
3
2
1
simple repeats
BLASTn
dot plot

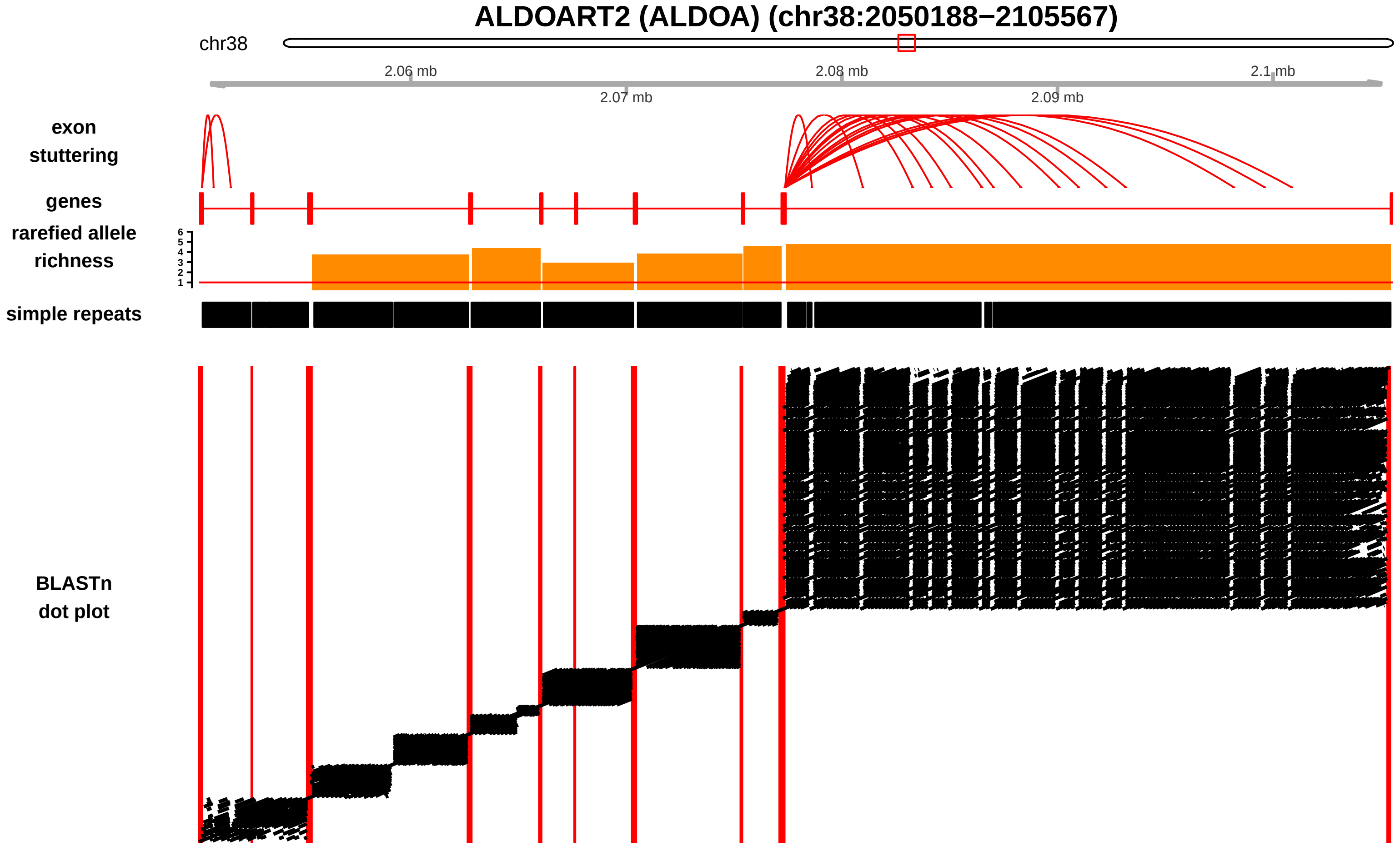
ALDOART2 (ALDOA) (chr38:2050188−2105567)
chr38
2.06 mb
2.07 mb
2.08 mb
2.09 mb
2.1 mb
exon stuttering
genes
rarefied allele richness
1
2
3
4
5
6
simple repeats
BLASTn dot plot

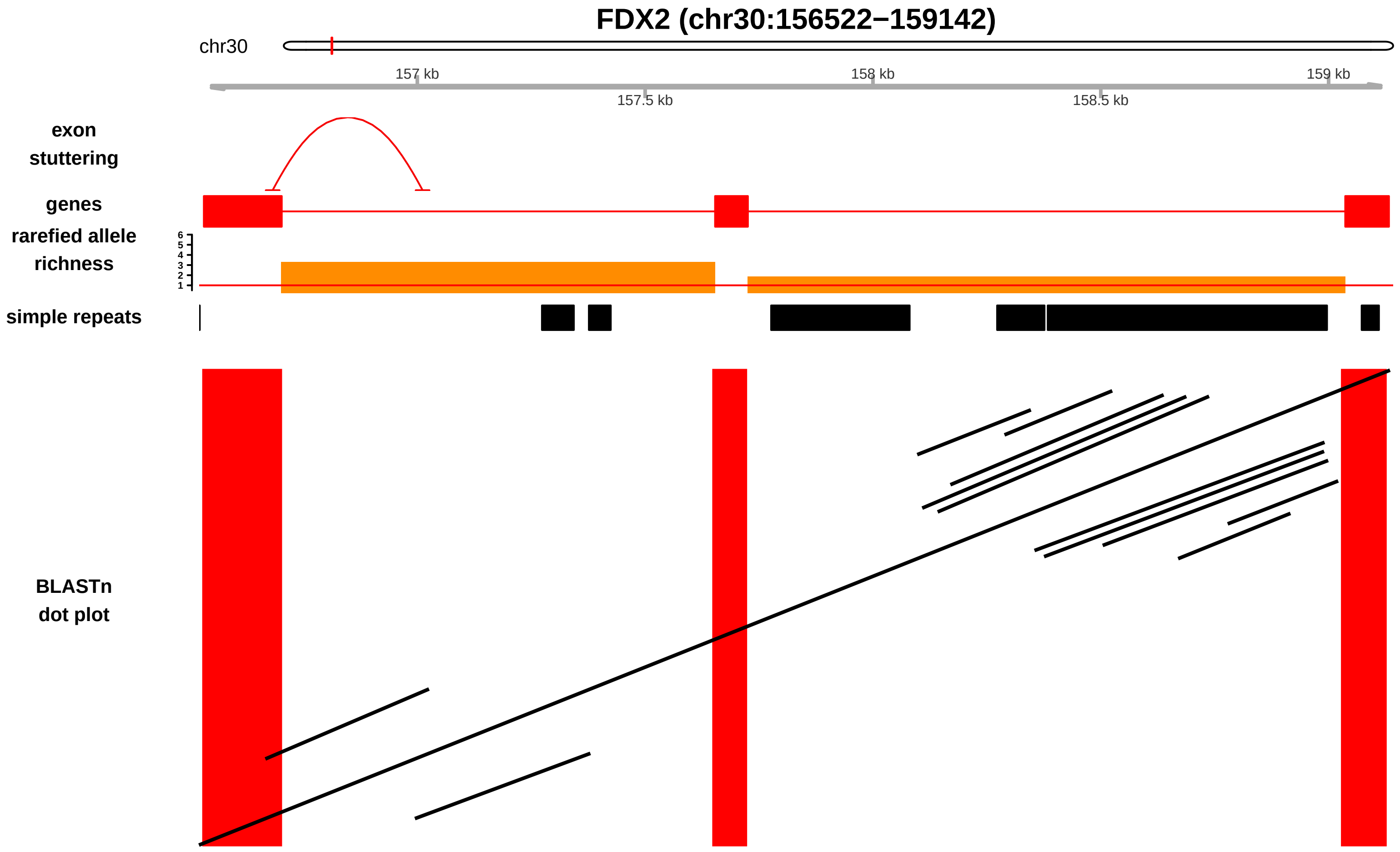

FDX2 (chr30:156522−159142)
chr30
157 kb
157.5 kb
158 kb
158.5 kb
159 kb
exon stuttering
genes
rarefied allele richness
6
5
4
3
2
1
simple repeats
BLASTn dot plot

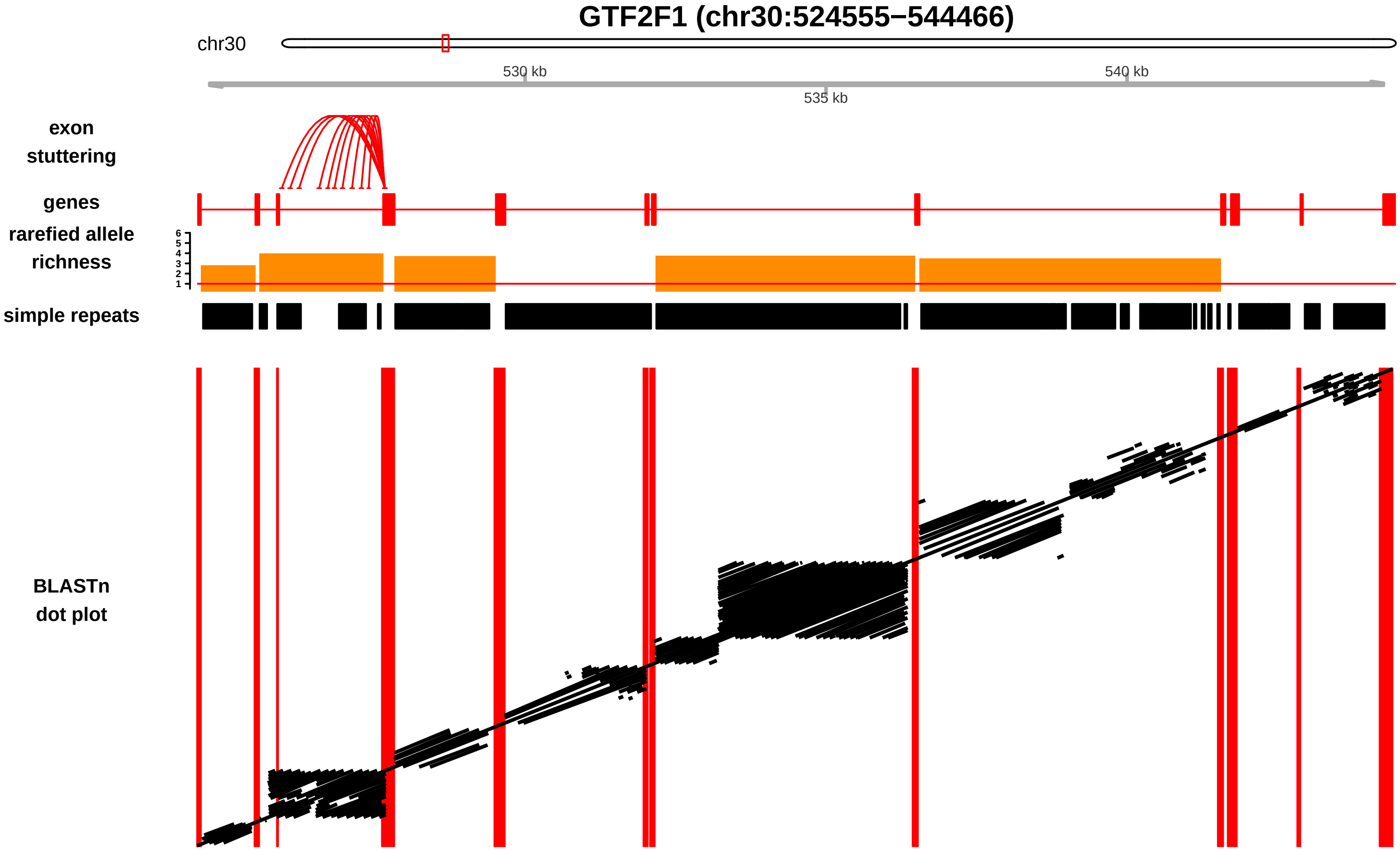

GTF2F1 (chr30:524555−544466)
chr30
530 kb
535 kb
540 kb
exon stuttering
genes
rarefied allele richness
6
5
4
3
2
1
simple repeats
BLASTn dot plot

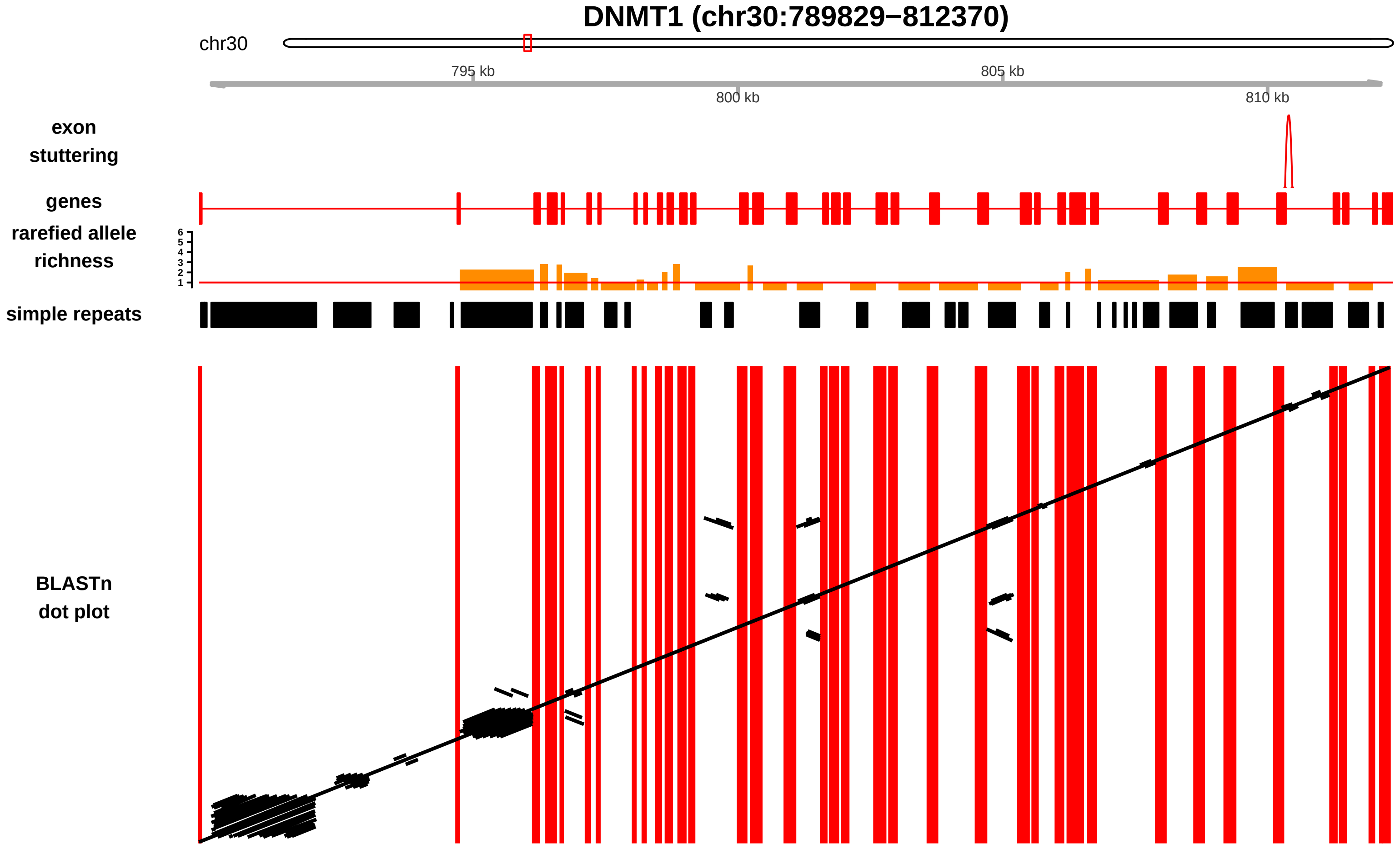

DNMT1 (chr30:789829−812370)
chr30
795 kb
800 kb
805 kb
810 kb
exon stuttering
genes
rarefied allele richness
1
2
3
4
5
6
simple repeats
BLASTn dot plot

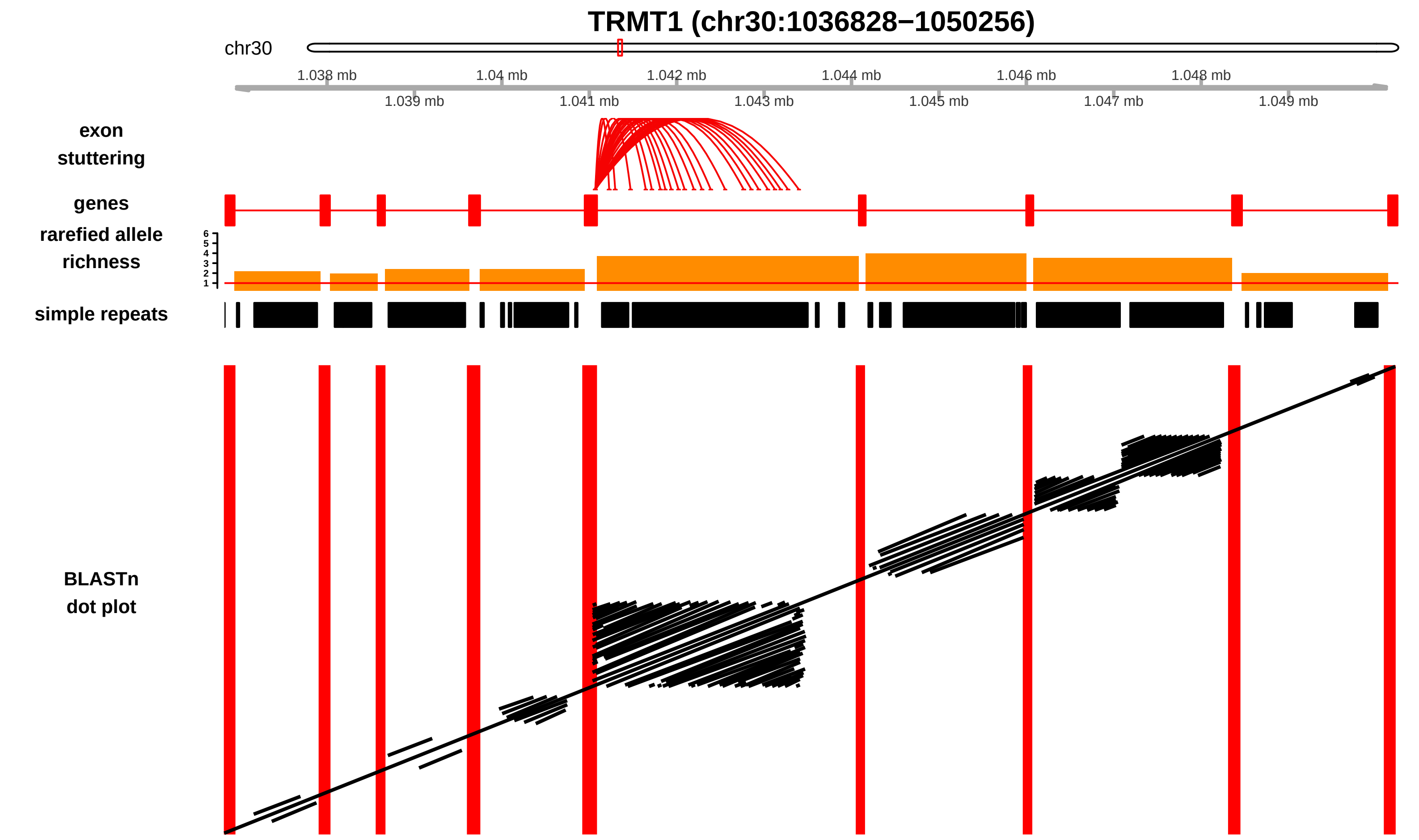
TRMT1 (chr30:1036828−1050256)
chr30
1.038 mb
1.04 mb
1.042 mb
1.044 mb
1.046 mb
1.048 mb
1.039 mb
1.041 mb
1.043 mb
1.045 mb
1.047 mb
1.049 mb
exon stuttering
genes
rarefied allele richness
6
5
4
3
2
1
simple repeats
BLASTn dot plot

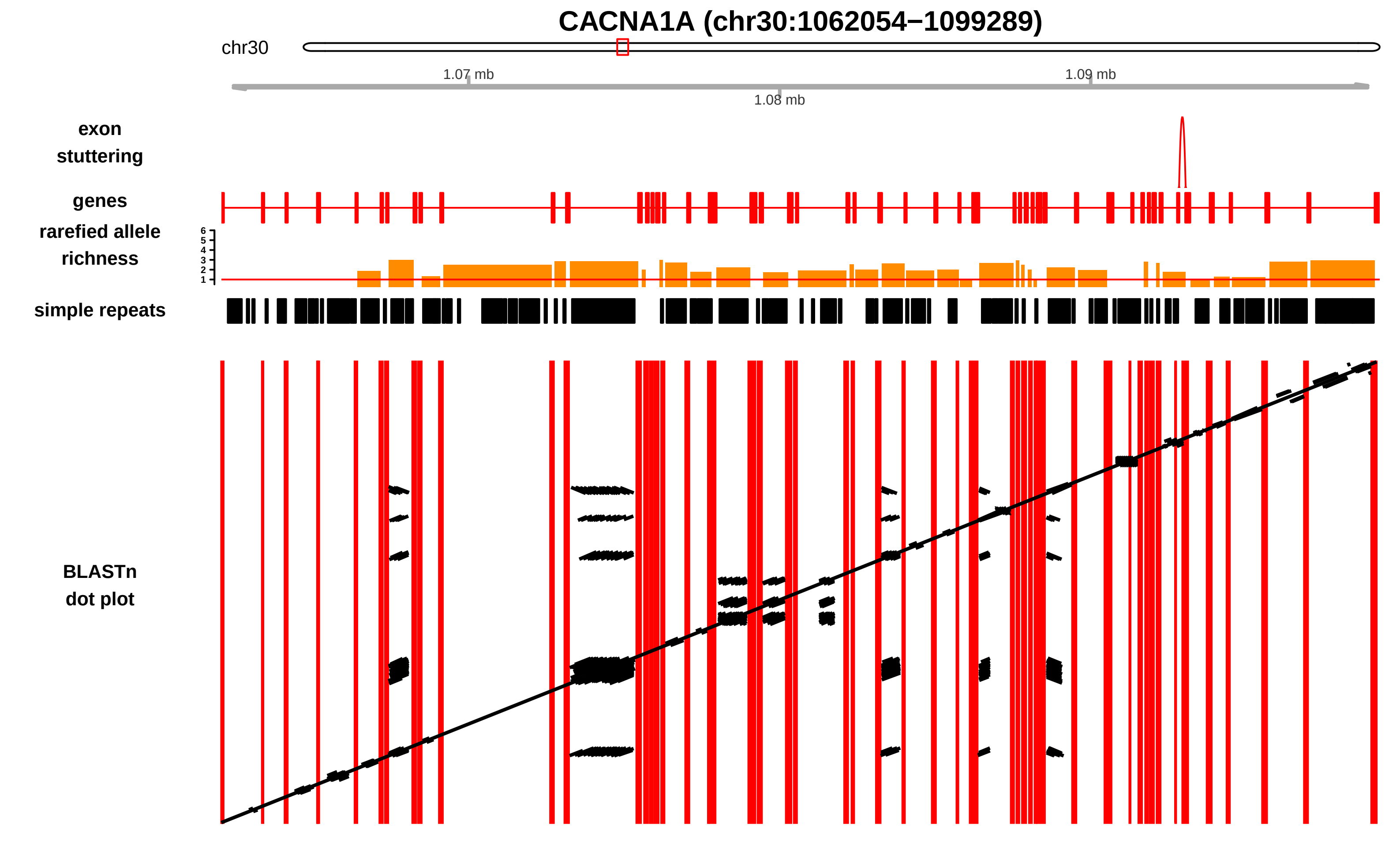
CACNA1A (chr30:1062054−1099289)
chr30
1.07 mb
1.08 mb
1.09 mb
exon stuttering
genes
rarefied allele richness
6
5
4
3
2
1
simple repeats
BLASTn dot plot

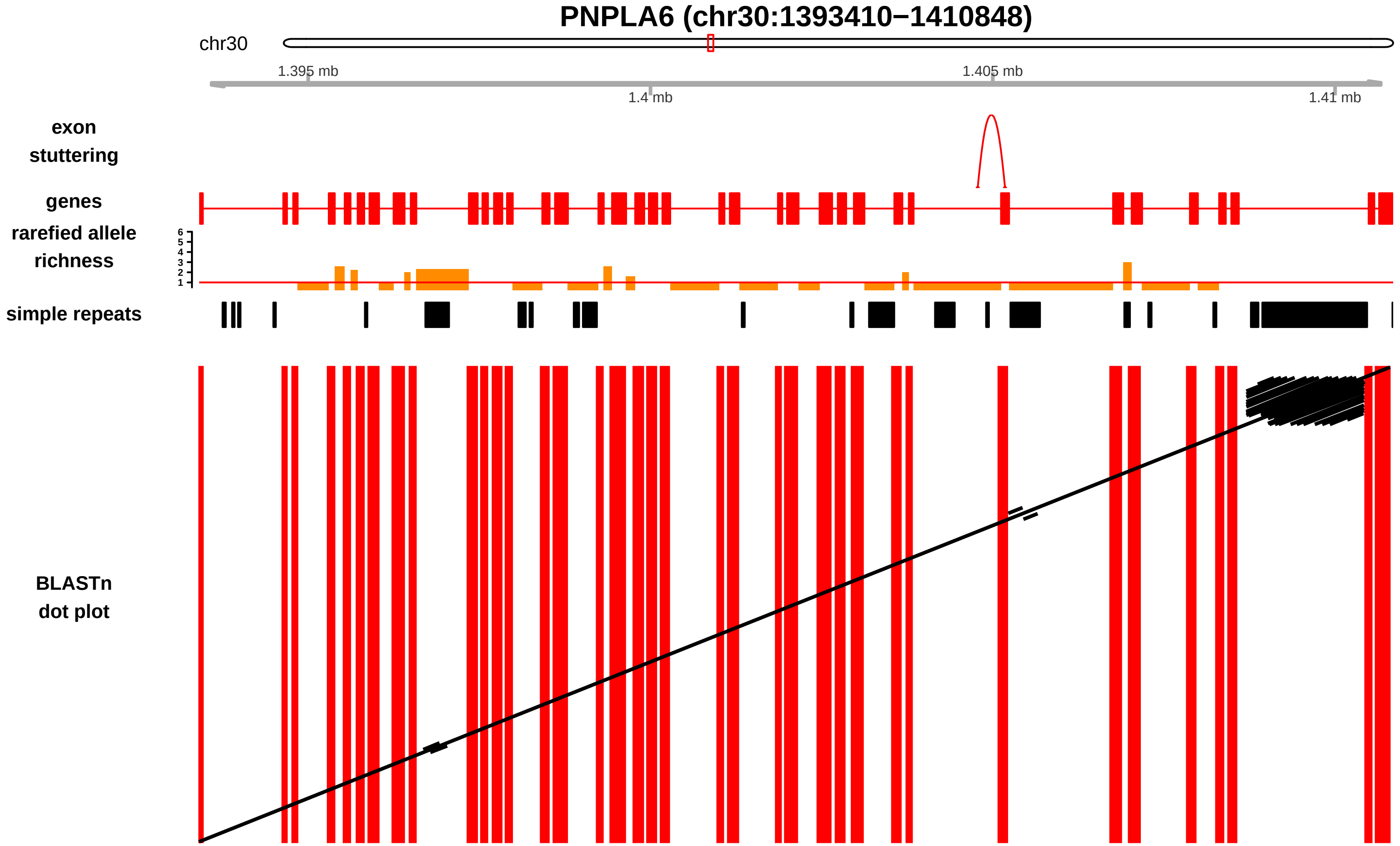
PNPLA6 (chr30:1393410−1410848)
chr30
1.395 mb
1.405 mb
1.4 mb
1.41 mb
exon stuttering
genes
rarefied allele richness
6
5
4
3
2
1
simple repeats
BLASTn dot plot

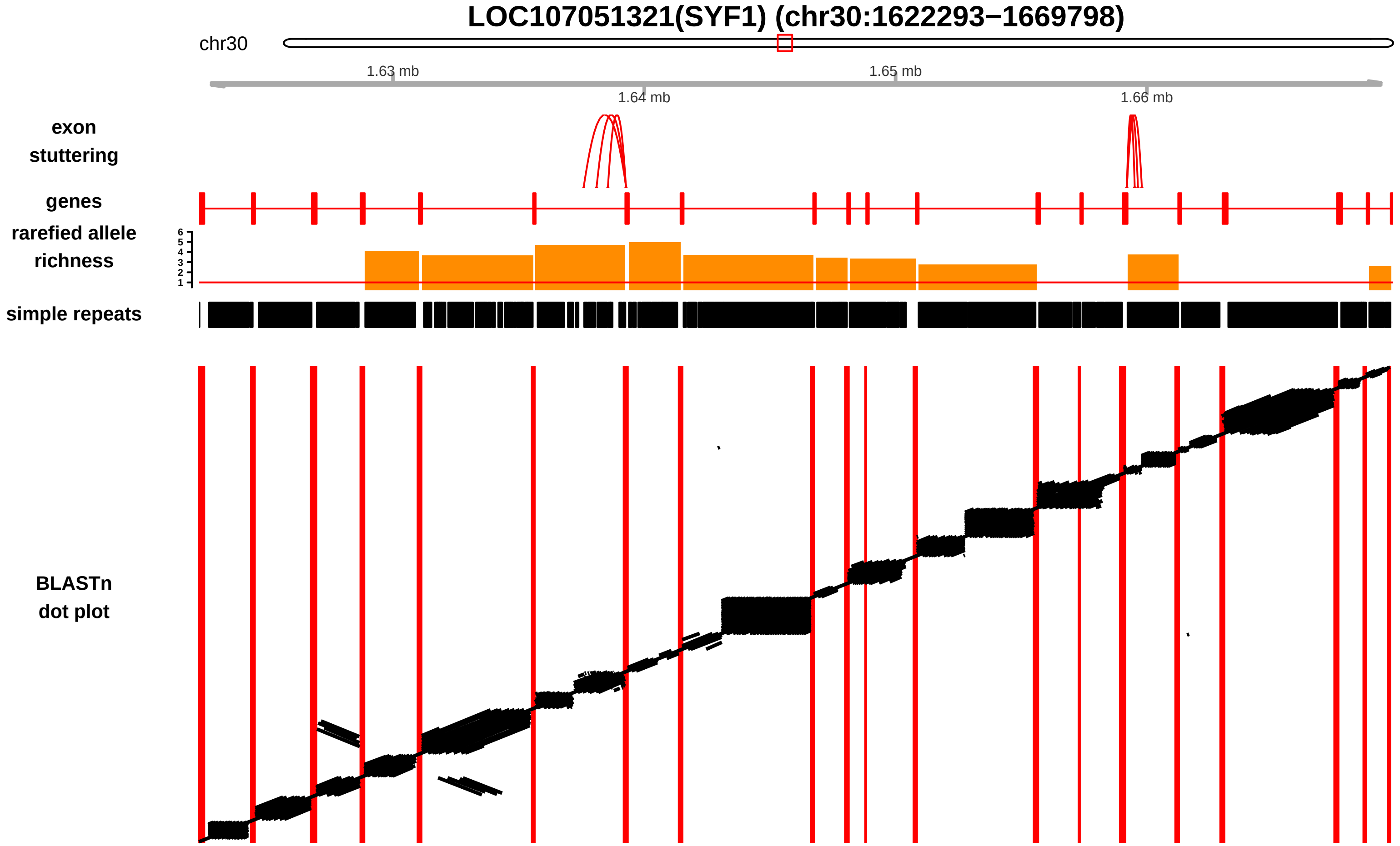
LOC107051321(SYF1) (chr30:1622293−1669798)
chr30
1.63 mb
1.64 mb
1.65 mb
1.66 mb
exon stuttering
genes
rarefied allele richness
6
5
4
3
2
1
simple repeats
BLASTn dot plot

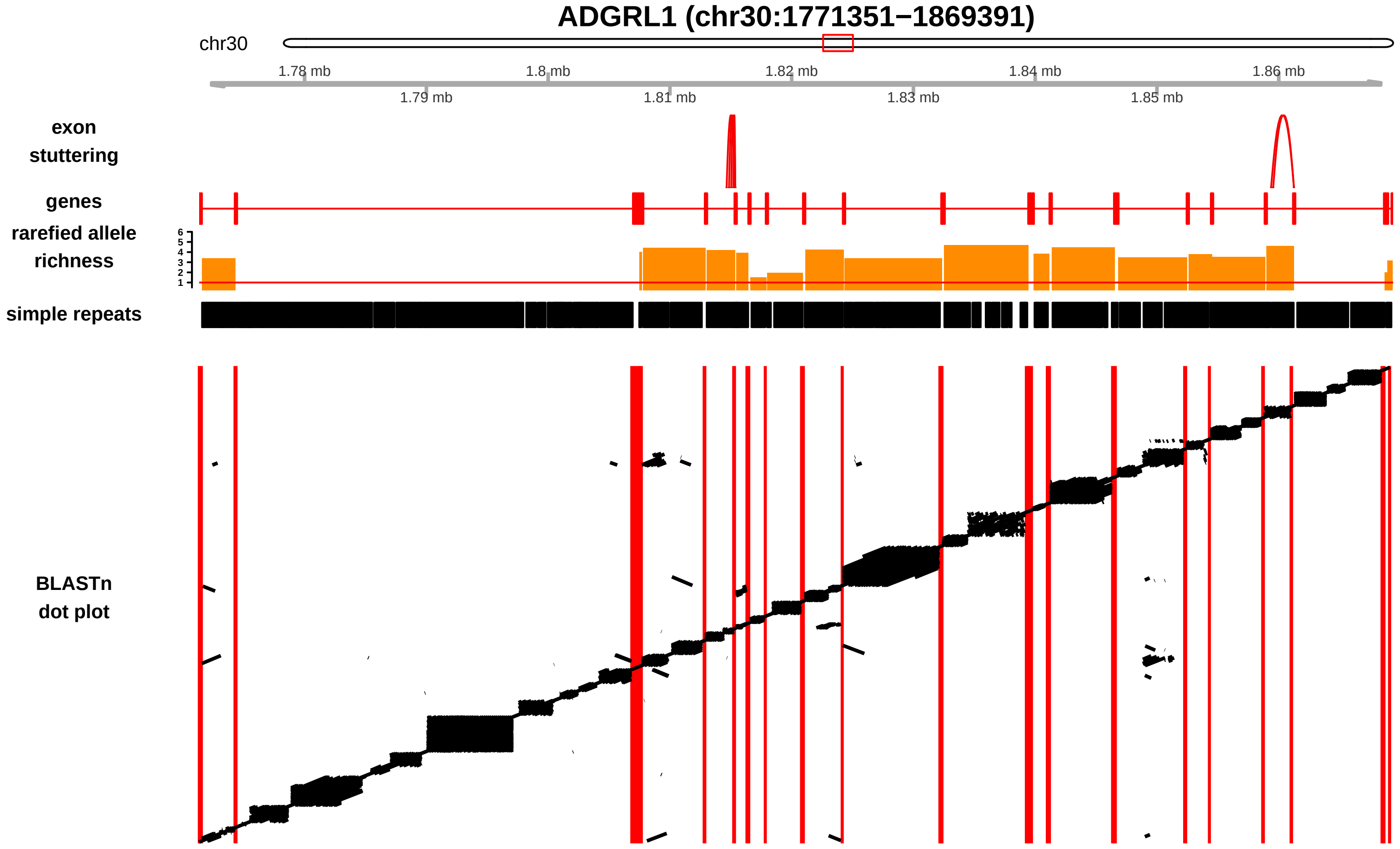

ADGRL1 (chr30:1771351−1869391)
chr30
1.78 mb
1.8 mb
1.82 mb
1.84 mb
1.86 mb
1.79 mb
1.81 mb
1.83 mb
1.85 mb
exon stuttering
genes
rarefied allele richness
1
2
3
4
5
6
simple repeats
BLASTn dot plot

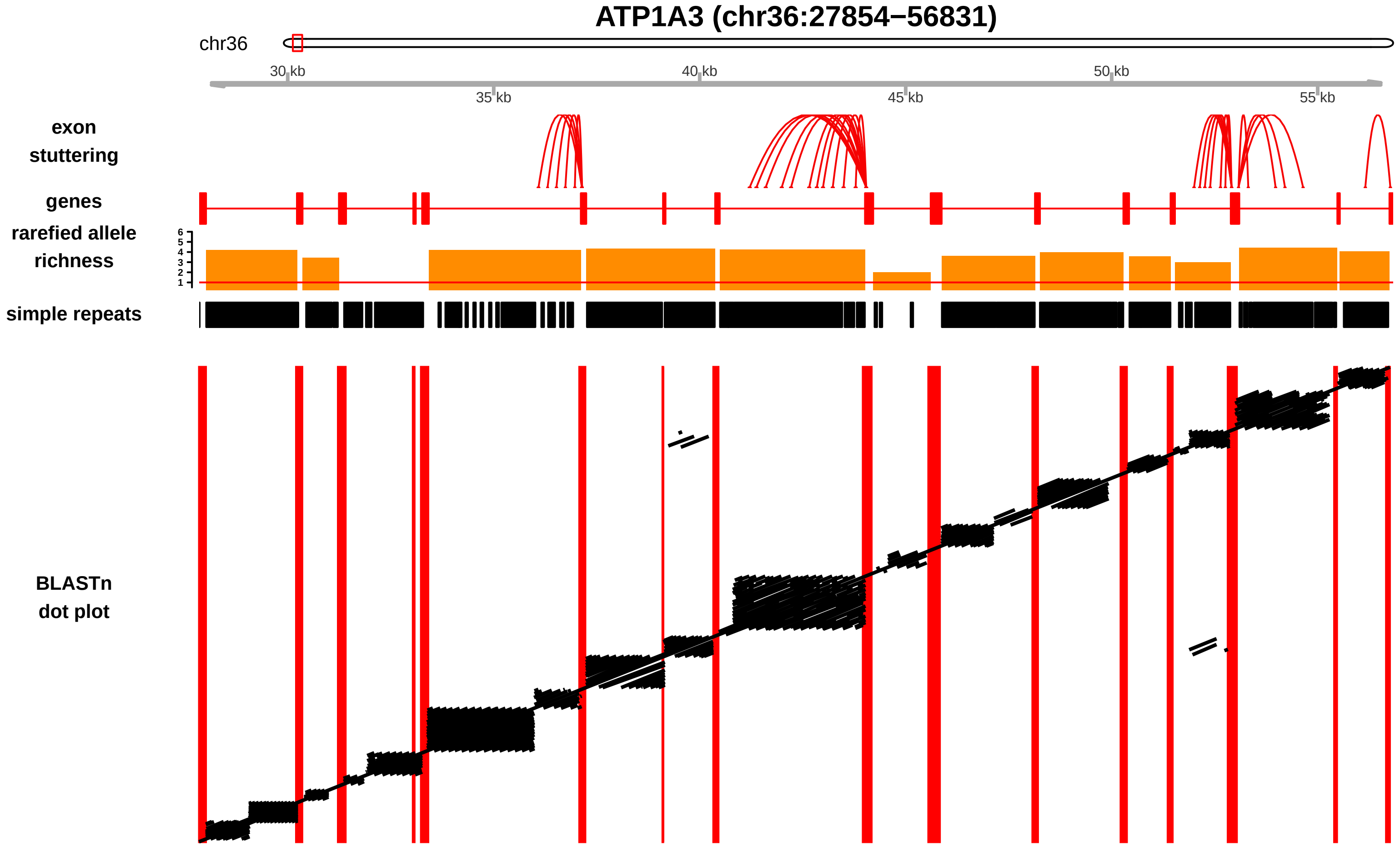

ATP1A3 (chr36:27854–56831)
chr36
30 kb
35 kb
40 kb
45 kb
50 kb
55 kb
exon stuttering
genes
rarefied allele richness
1
2
3
4
5
6
simple repeats
BLASTn dot plot

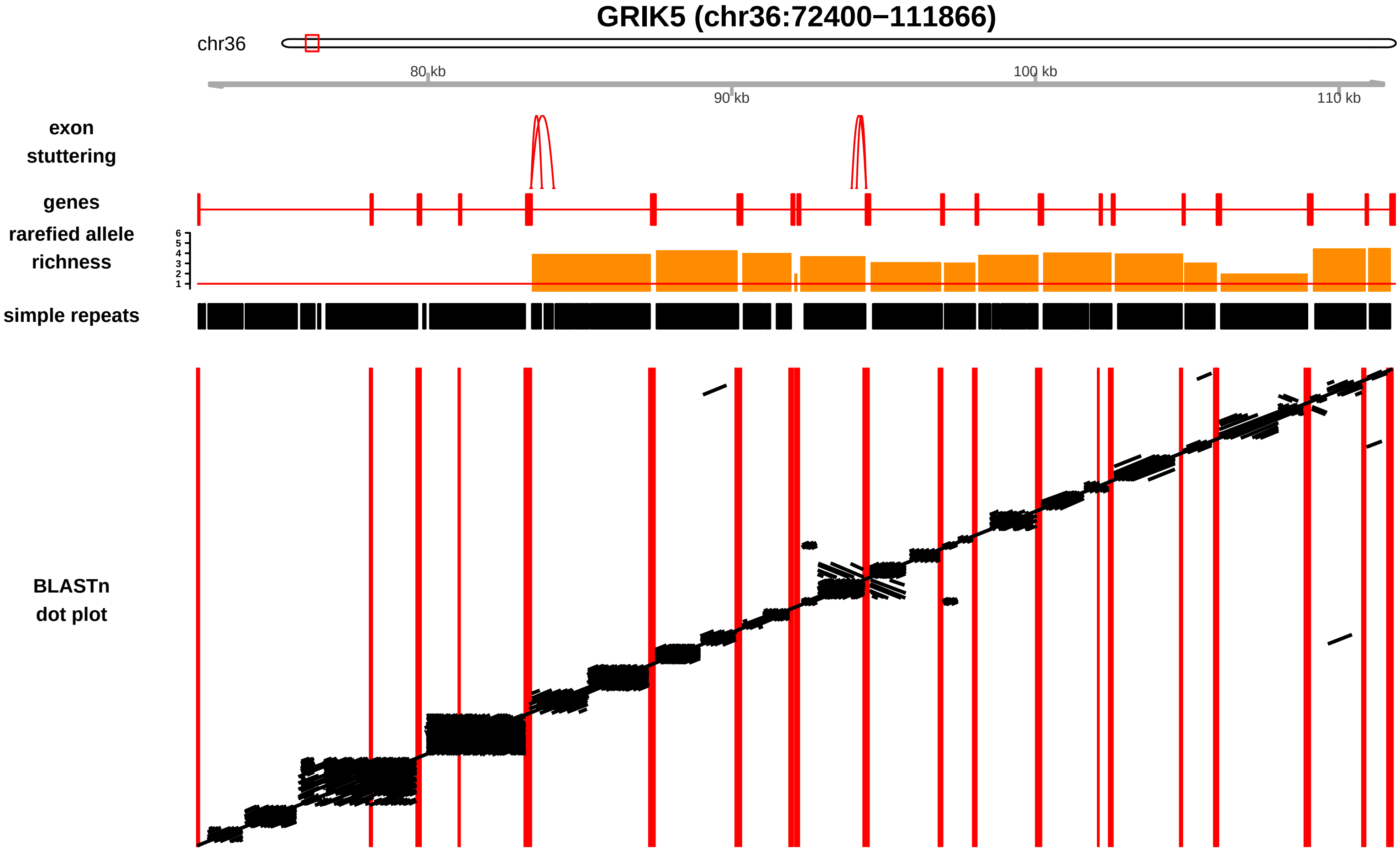
GRIK5 (chr36:72400−111866)
chr36
80 kb
100 kb
90 kb
110 kb
exon
stuttering
genes
rarefied allele
richness
6
5
4
3
2
1
simple repeats
BLASTn
dot plot

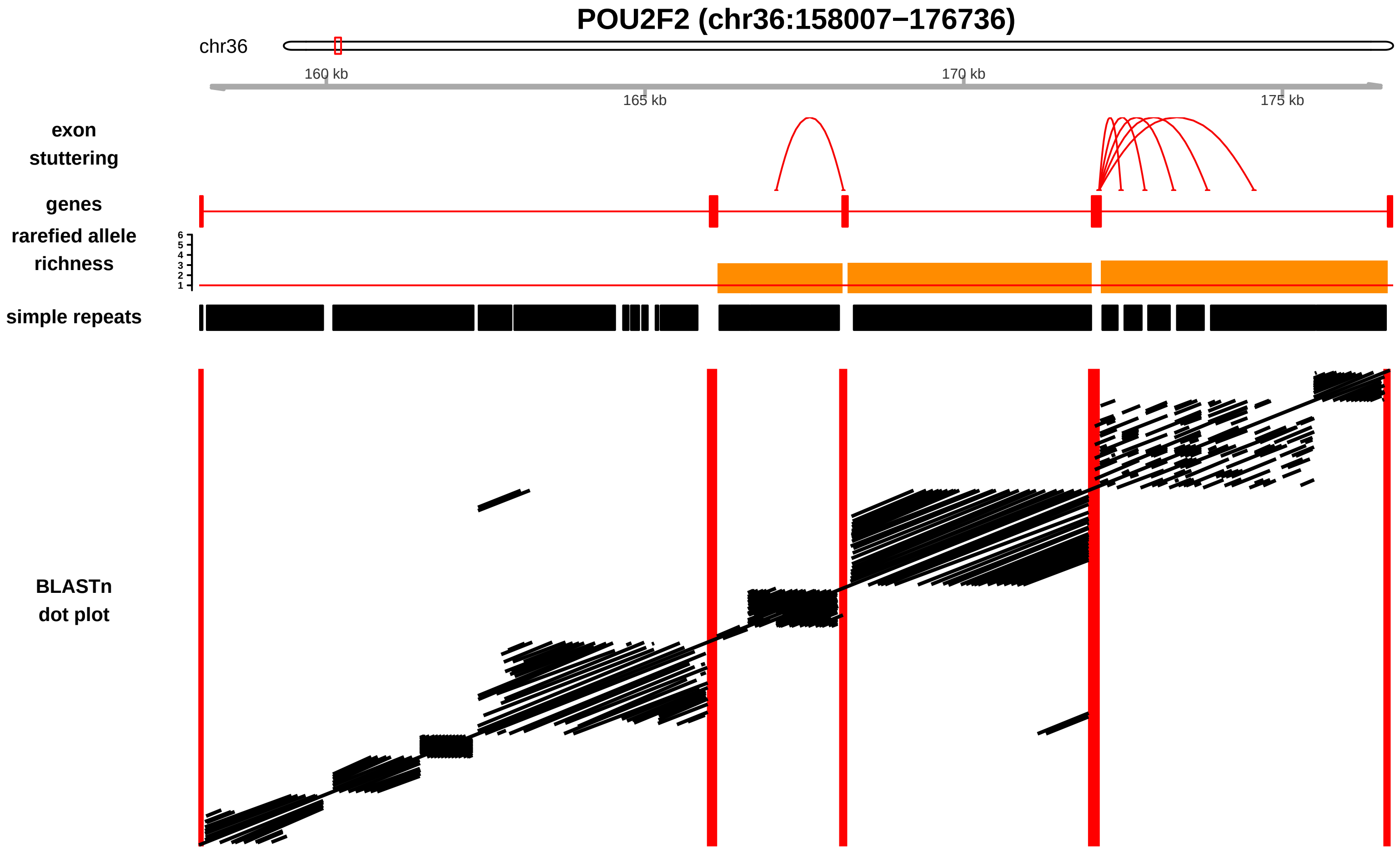

POU2F2 (chr36:158007−176736)
chr36
160 kb
165 kb
170 kb
175 kb
exon stuttering
genes
rarefied allele richness
6
5
4
3
2
1
simple repeats
BLASTn dot plot

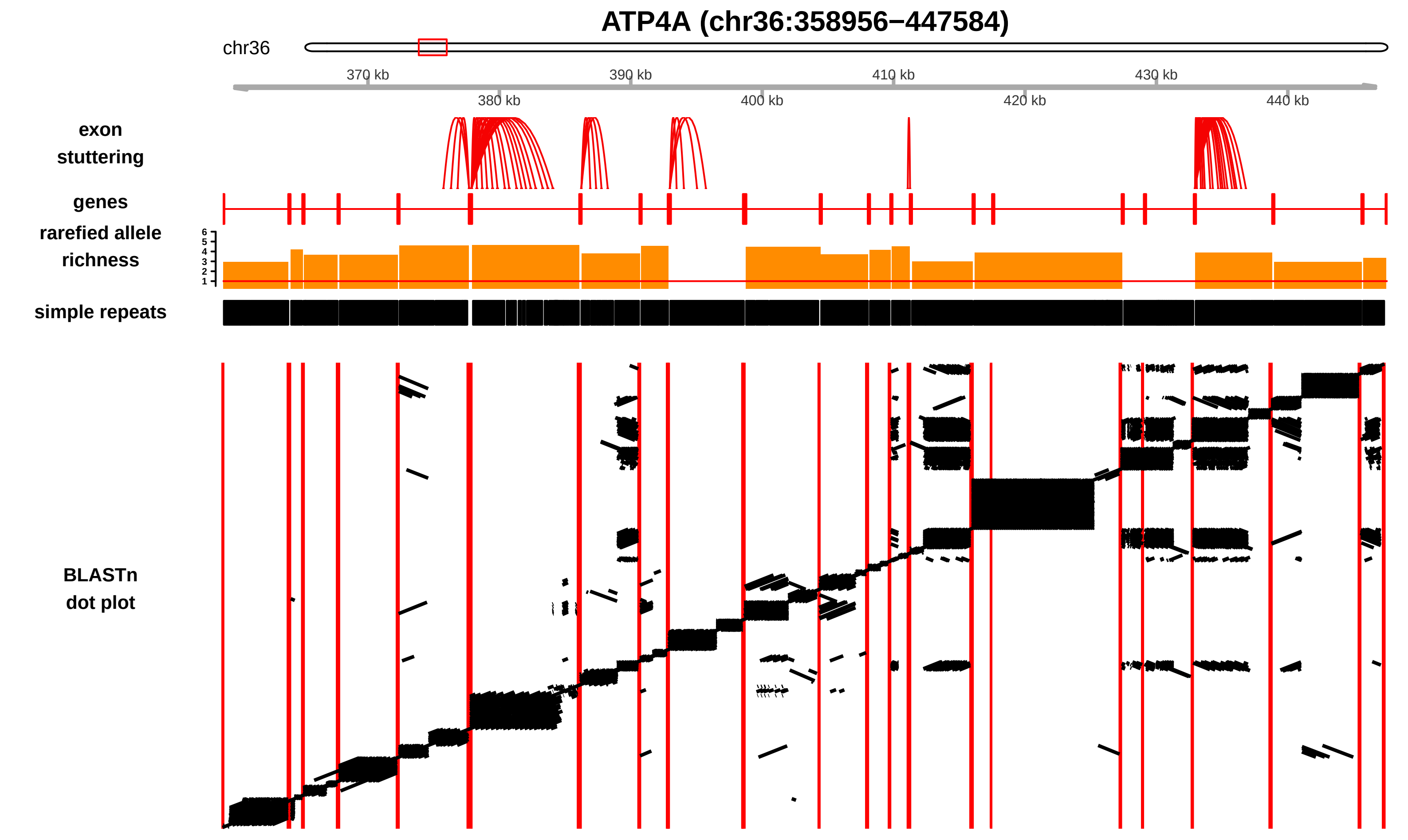

ATP4A (chr36:358956−447584)
chr36
370 kb
390 kb
410 kb
430 kb
380 kb
400 kb
420 kb
440 kb
exon stuttering
genes
rarefied allele richness
6
5
4
3
2
1
simple repeats
BLASTn dot plot

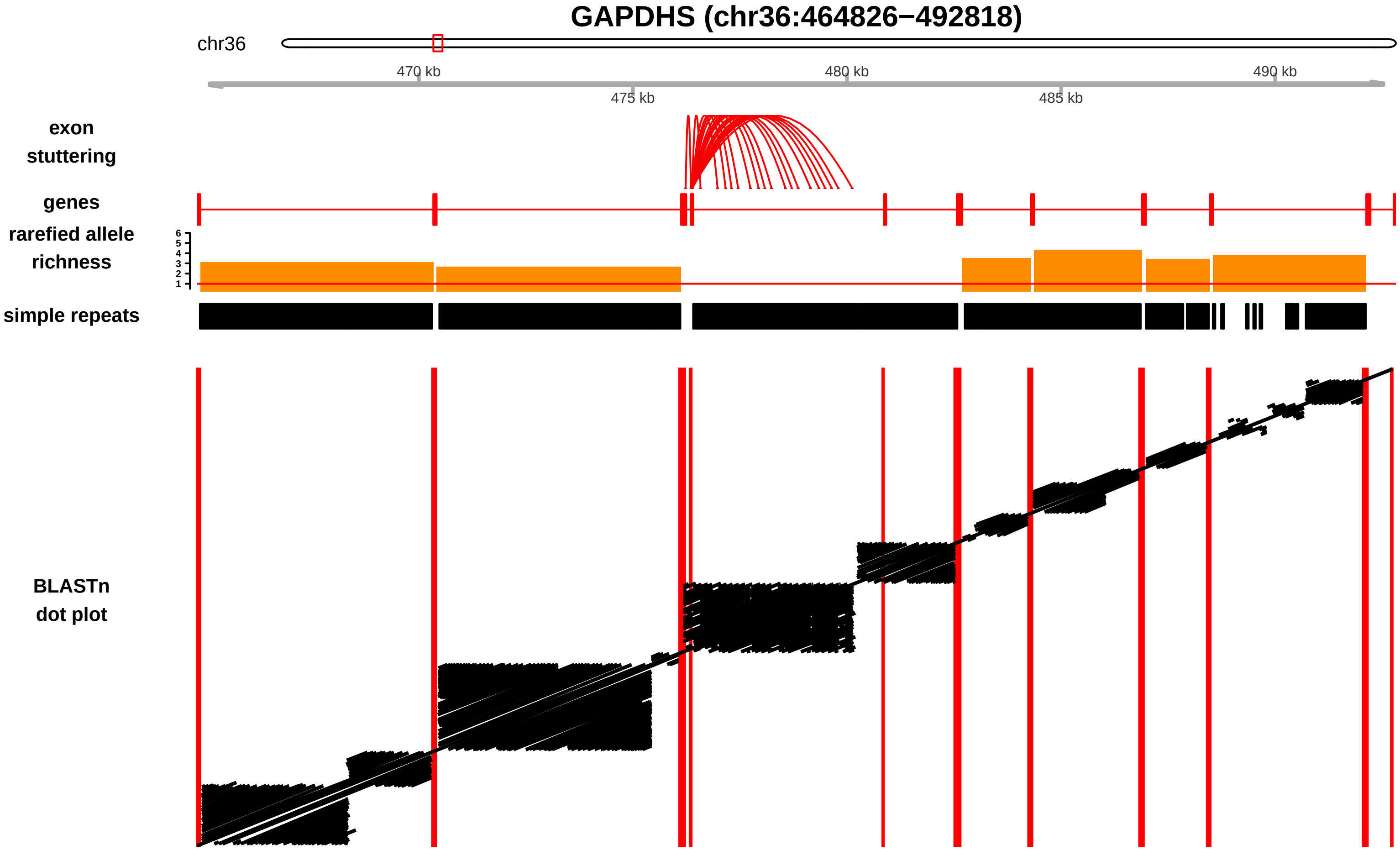
GAPDHS (chr36:464826−492818)
chr36
470 kb
480 kb
490 kb
475 kb
485 kb
exon
stuttering
genes
rarefied allele
richness
6
5
4
3
2
1
simple repeats
BLASTn
dot plot

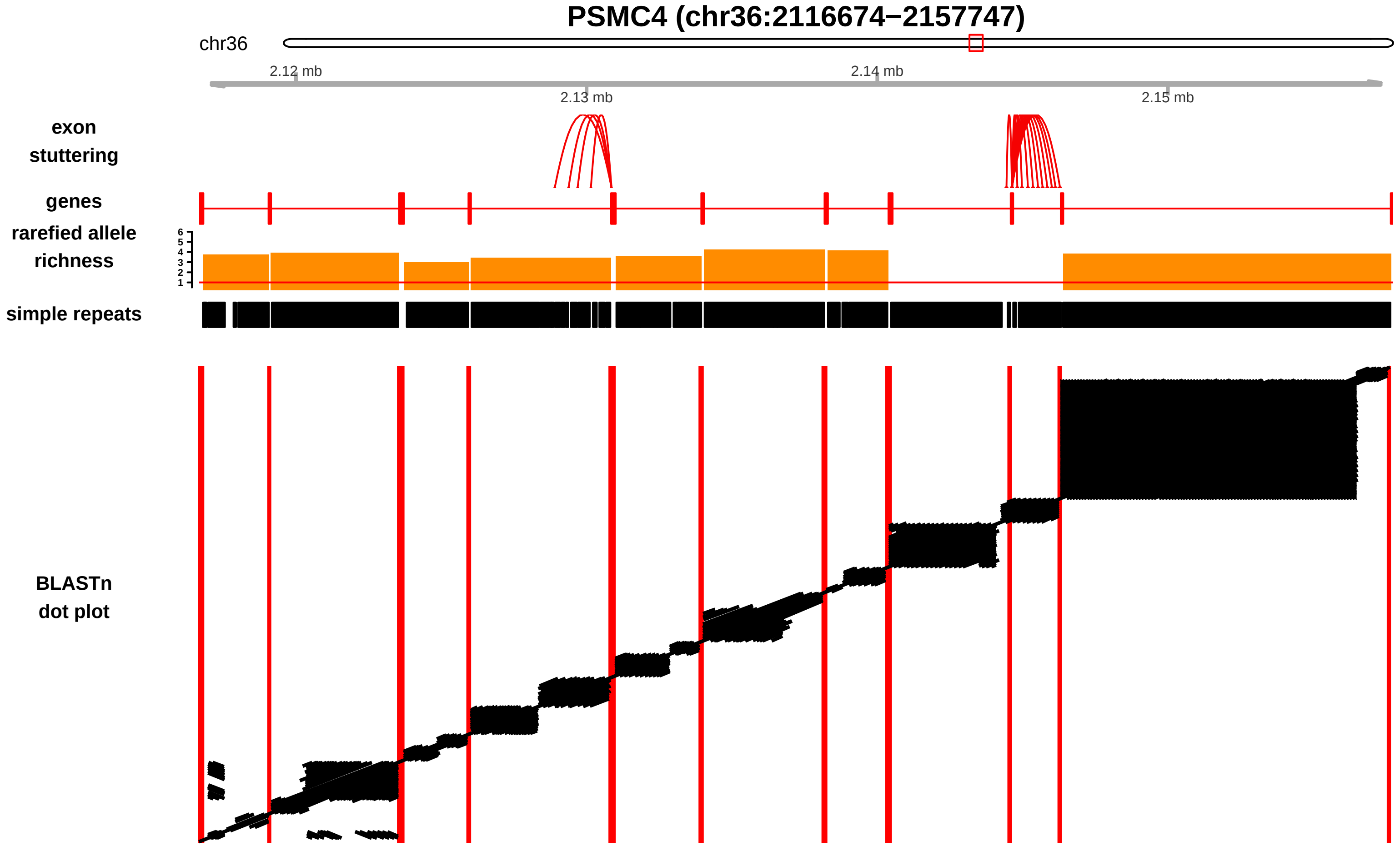

PSMC4 (chr36:2116674–2157747)
chr36
2.12 mb
2.13 mb
2.14 mb
2.15 mb
exon stuttering
genes
rarefied allele richness
1
2
3
4
5
6
simple repeats
BLASTn dot plot

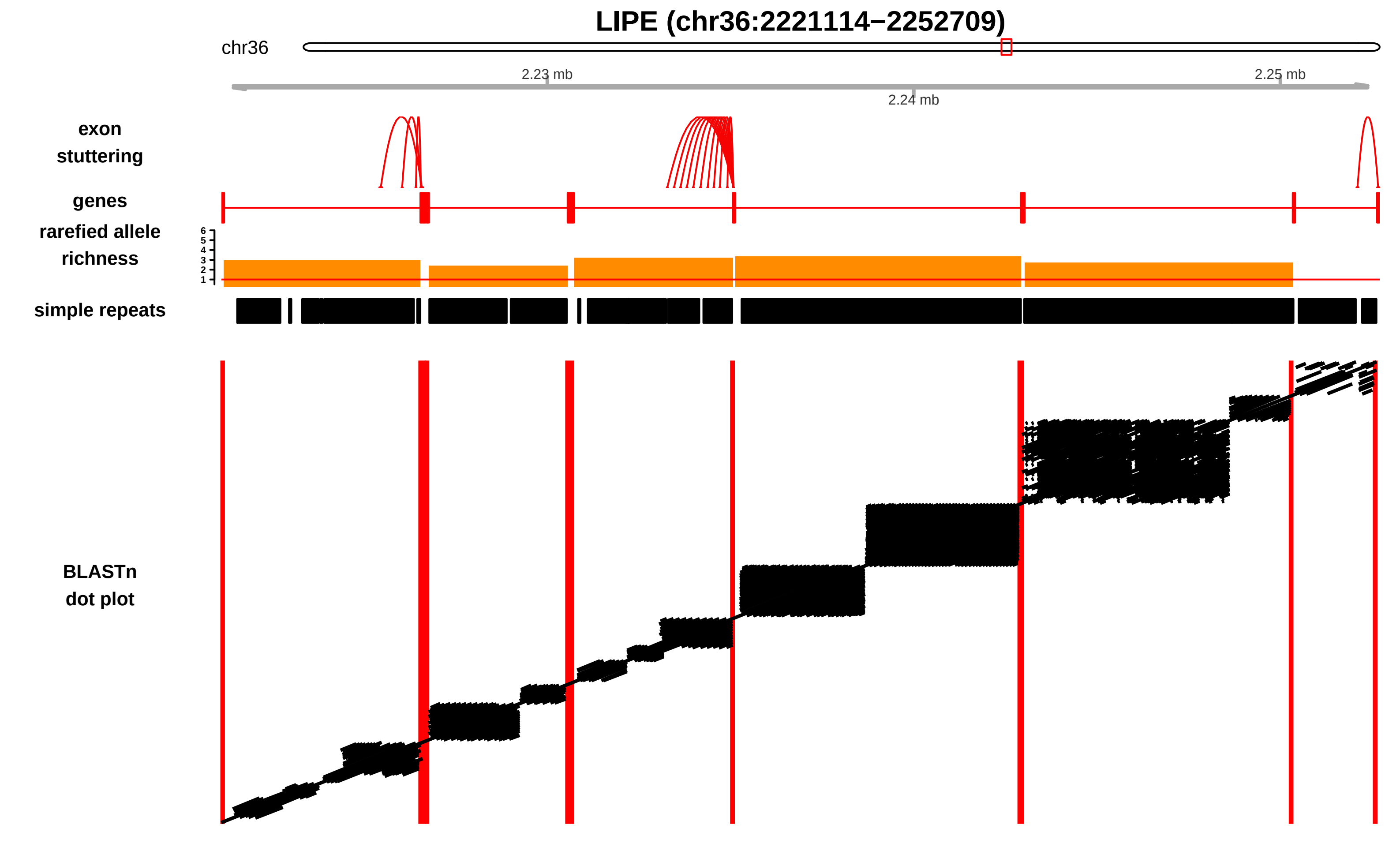

LIPE (chr36:2221114−2252709)
chr36
2.23 mb
2.24 mb
2.25 mb
exon
stuttering
genes
rarefied allele
richness
6
5
4
3
2
1
simple repeats
BLASTn
dot plot

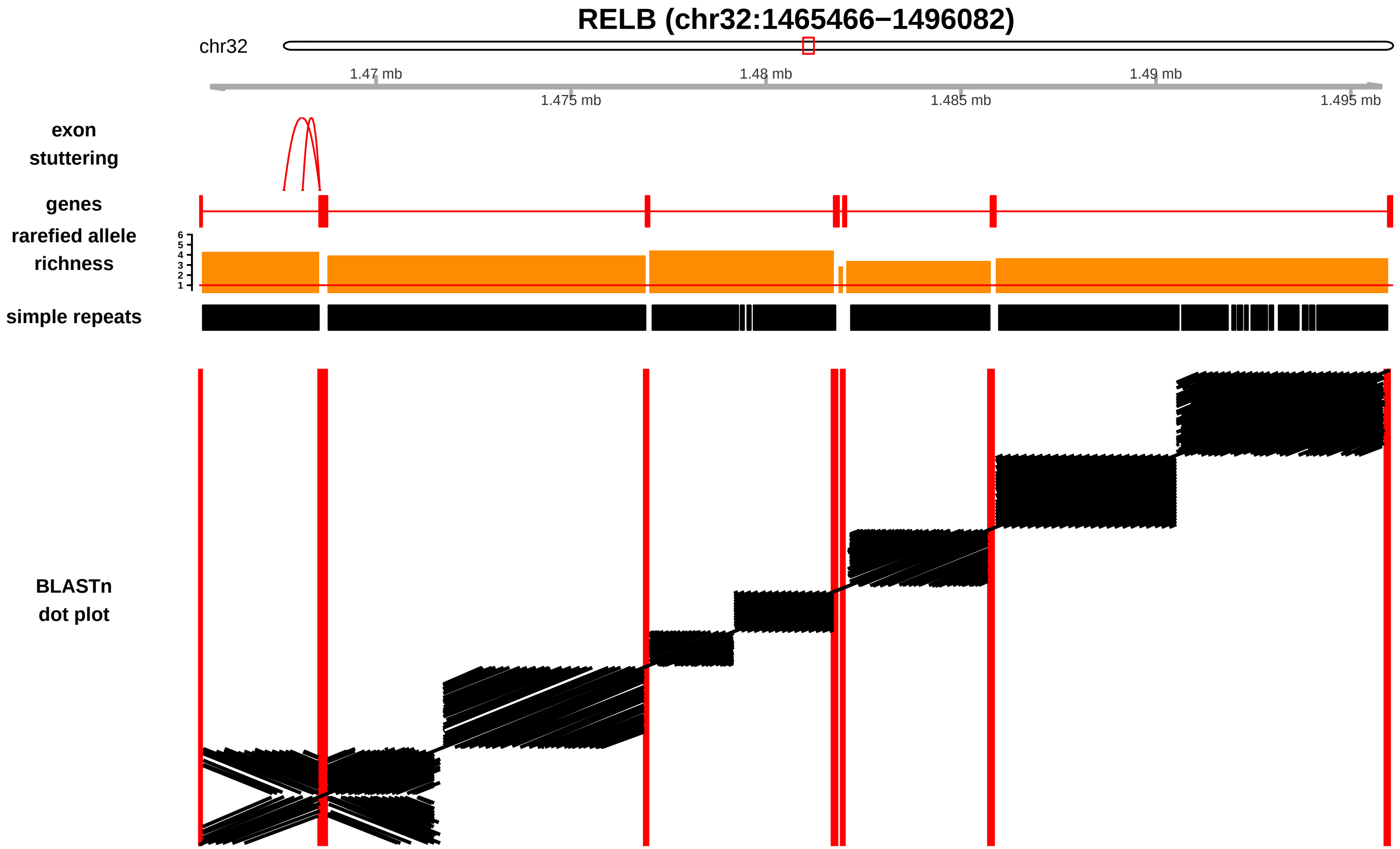
RELB (chr32:1465466−1496082)
chr32
1.47 mb
1.48 mb
1.49 mb
1.475 mb
1.485 mb
1.495 mb
exon
stuttering
genes
rarefied allele
richness
6
5
4
3
2
1
simple repeats
BLASTn
dot plot

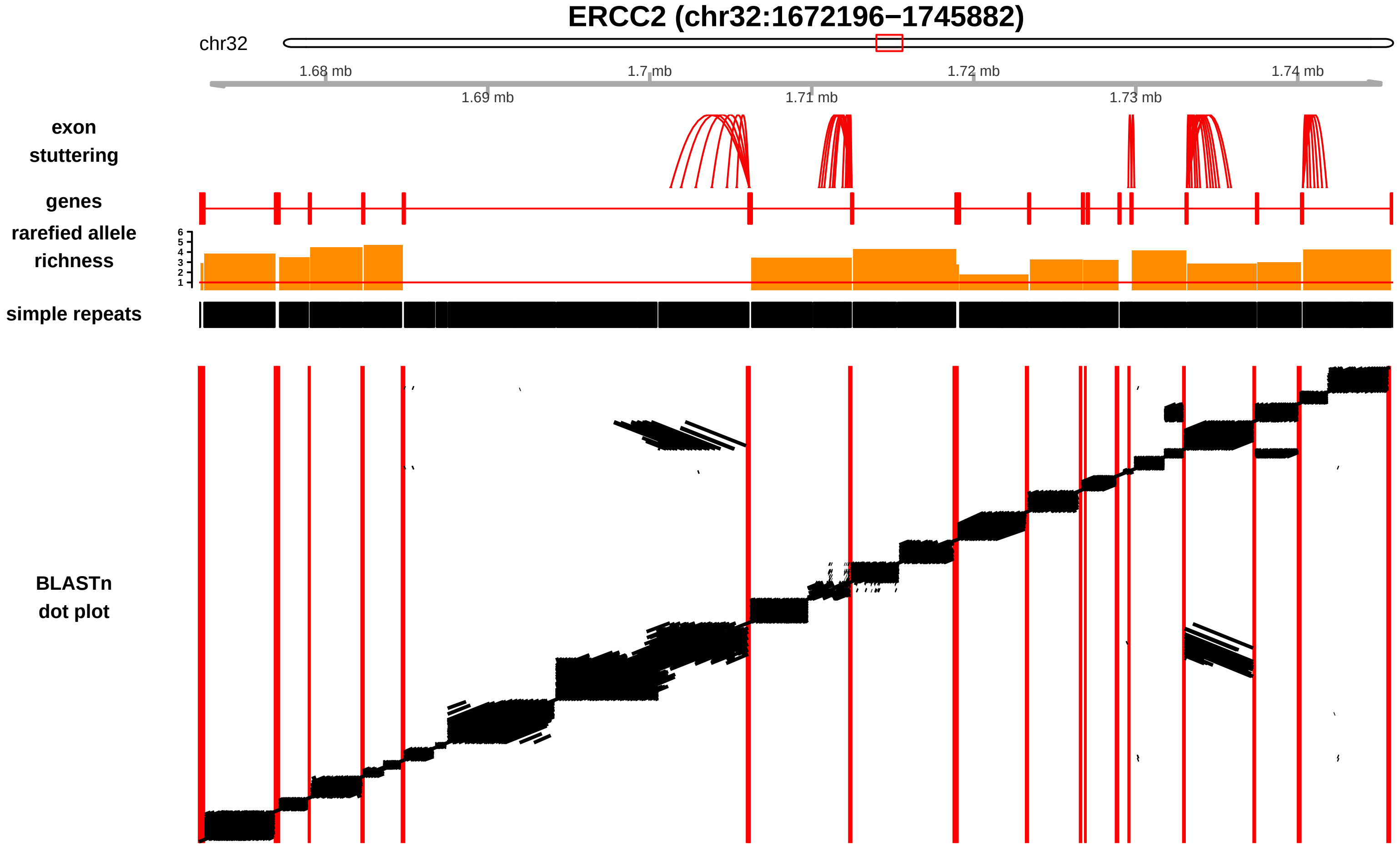
ERCC2 (chr32:1672196−1745882)
chr32
1.68 mb
1.69 mb
1.7 mb
1.71 mb
1.72 mb
1.73 mb
1.74 mb
exon stuttering
genes
rarefied allele richness
6
5
4
3
2
1
simple repeats
BLASTn dot plot

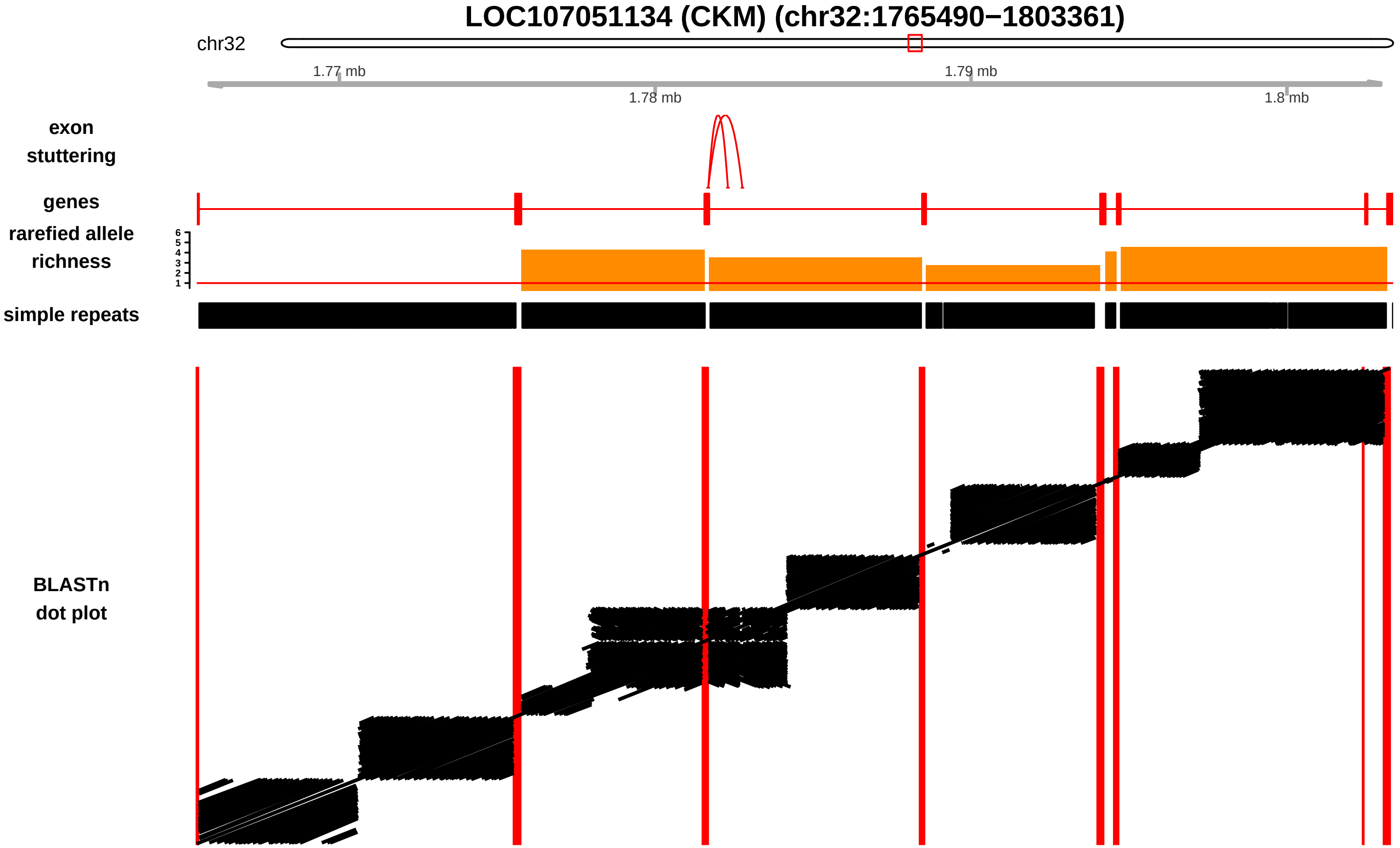

LOC107051134 (CKM) (chr32:1765490−1803361)
chr32
1.77 mb
1.78 mb
1.79 mb
1.8 mb
exon stuttering
genes
rarefied allele richness
6
5
4
3
2
1
simple repeats
BLASTn dot plot

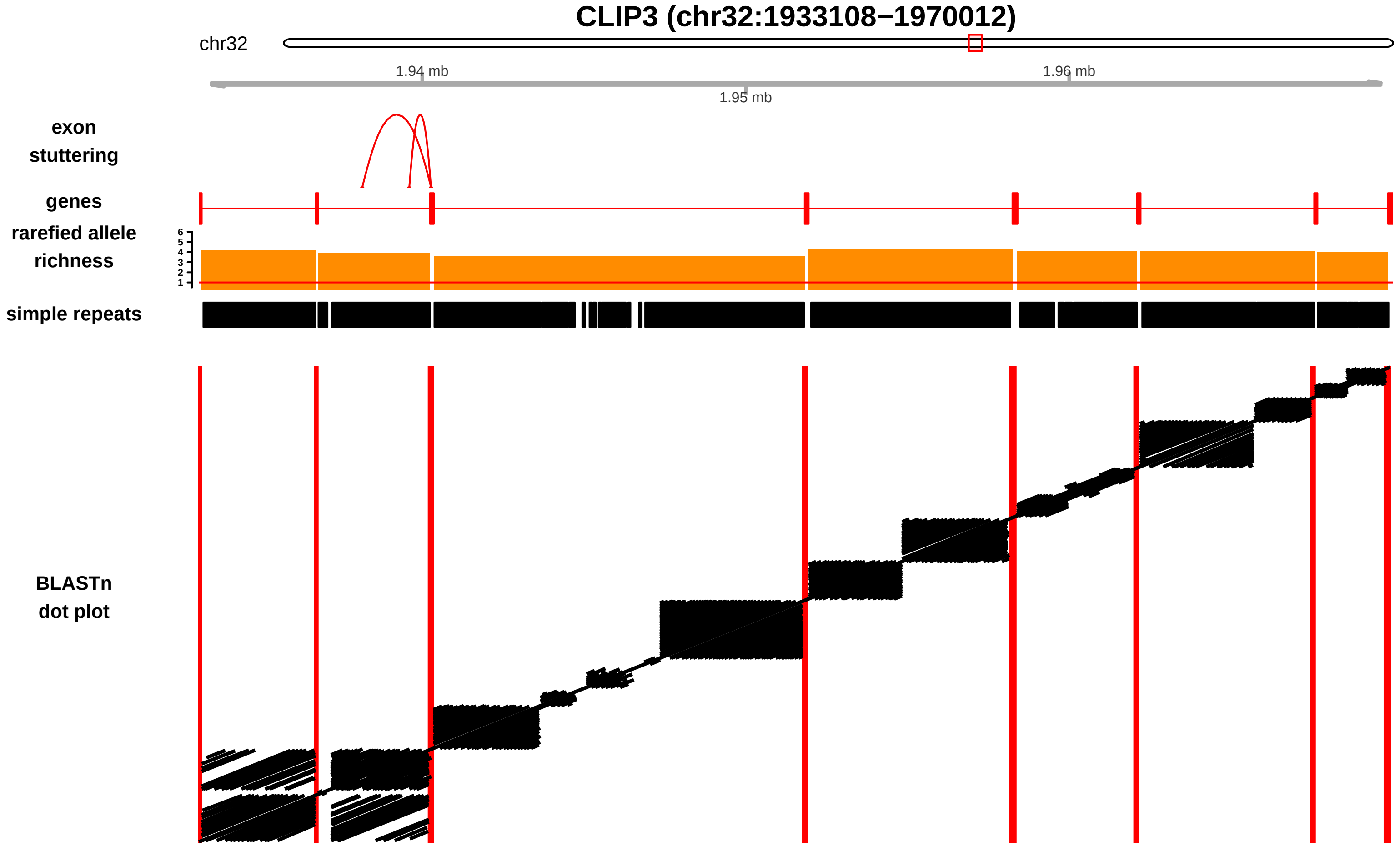
CLIP3 (chr32:1933108−1970012)
chr32
1.94 mb
1.95 mb
1.96 mb
exon stuttering
genes
rarefied allele richness
6
5
4
3
2
1
simple repeats
BLASTn dot plot

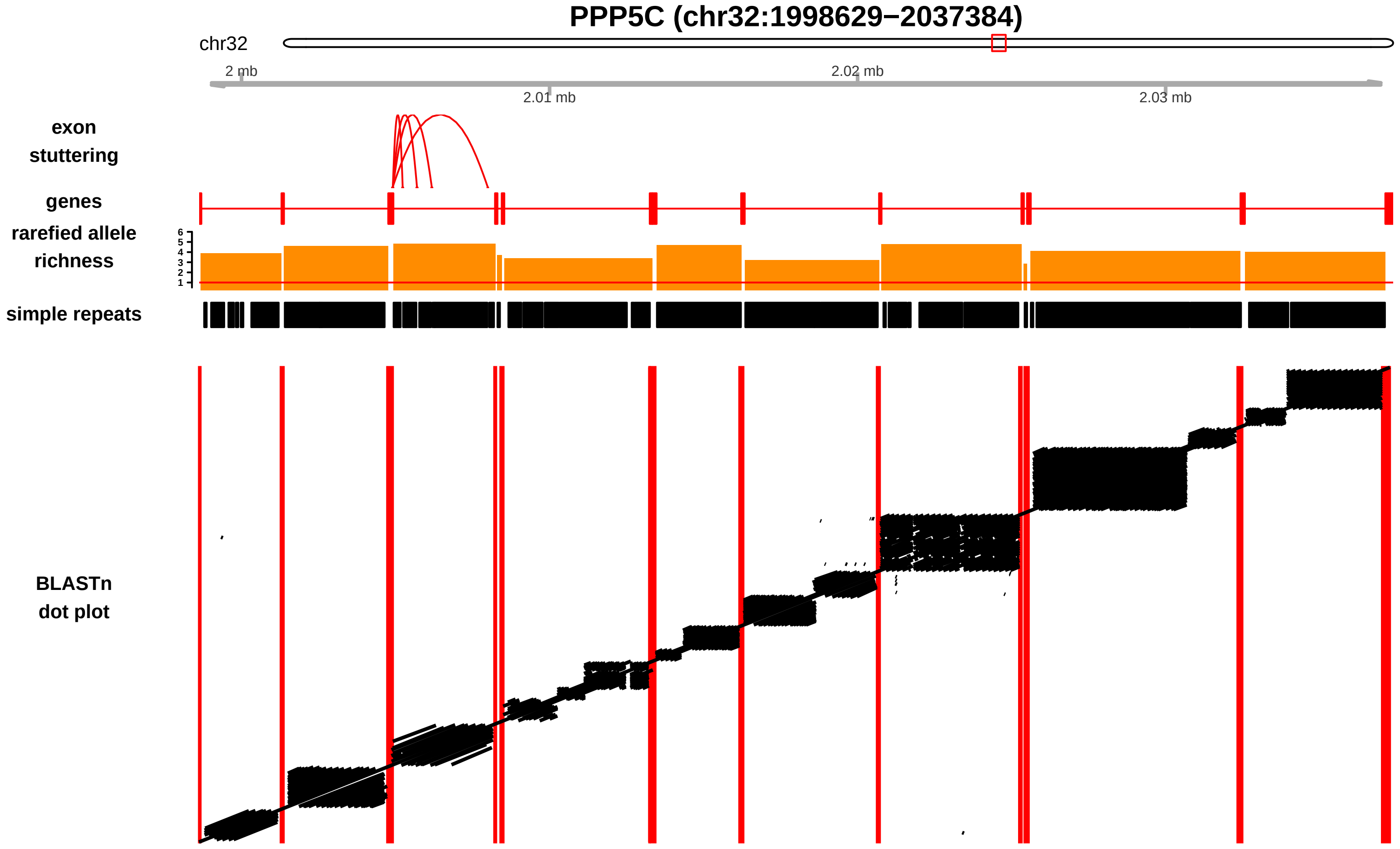

PPP5C (chr32:1998629−2037384)
chr32
2 mb
2.01 mb
2.02 mb
2.03 mb
exon stuttering
genes
rarefied allele richness
6
5
4
3
2
1
simple repeats
BLASTn dot plot

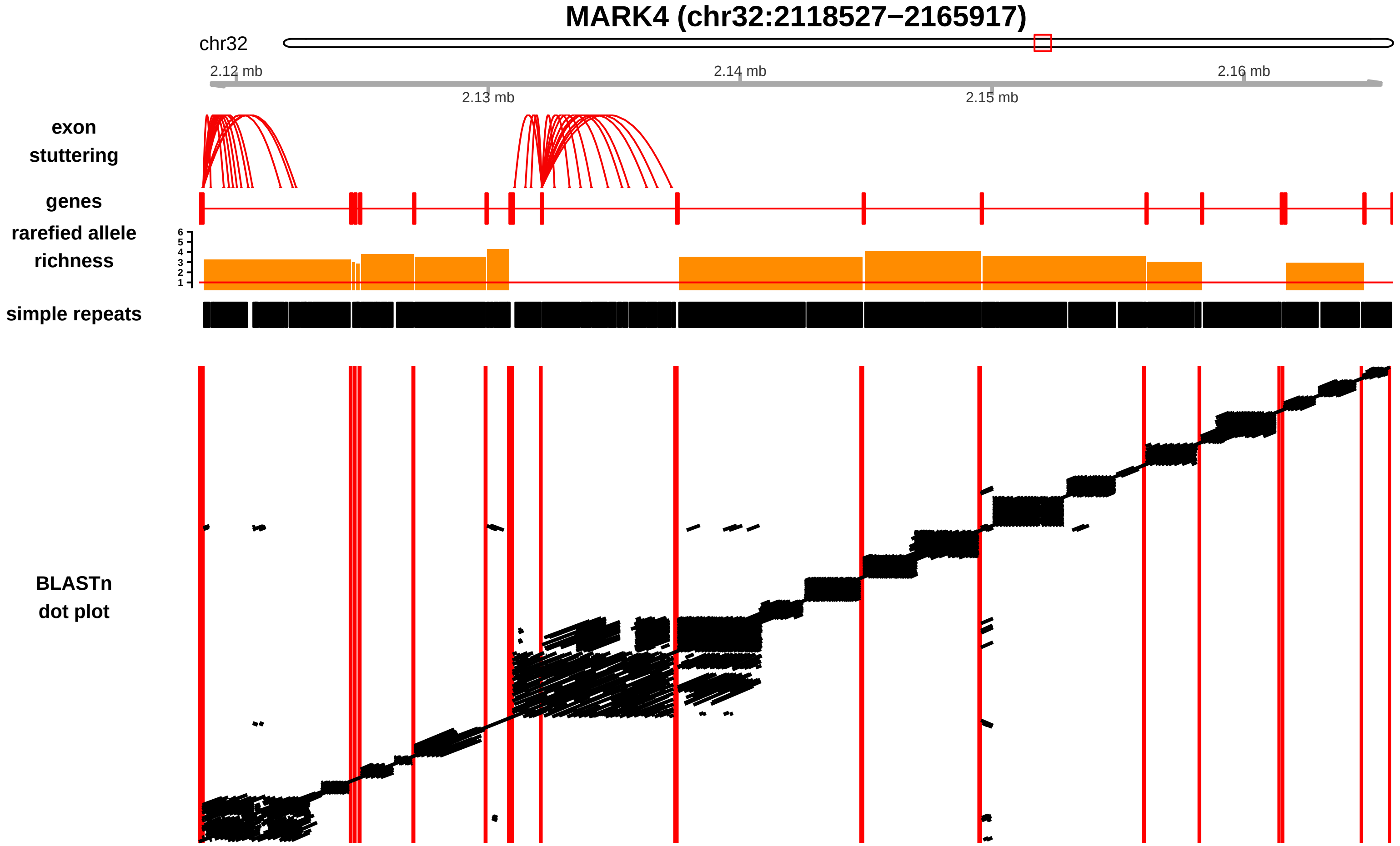
MARK4 (chr32:2118527−2165917)
chr32
2.12 mb
2.13 mb
2.14 mb
2.15 mb
2.16 mb
exon stuttering
genes
rarefied allele richness
1
2
3
4
5
6
simple repeats
BLASTn dot plot

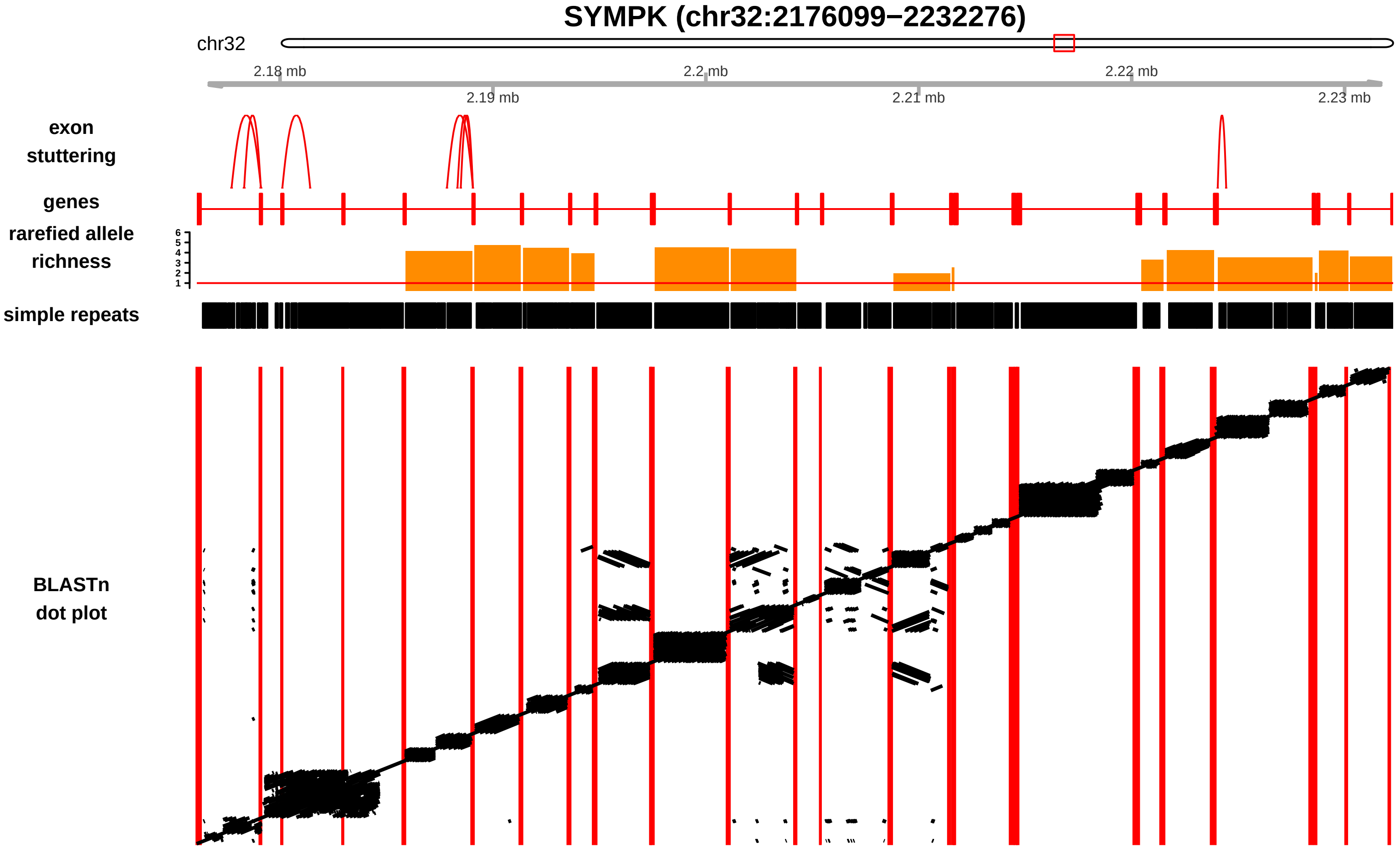
SYMPK (chr32:2176099−2232276)
chr32
2.18 mb
2.19 mb
2.2 mb
2.21 mb
2.22 mb
2.23 mb
exon stuttering
genes
rarefied allele richness
1
2
3
4
5
6
simple repeats
BLASTn dot plot

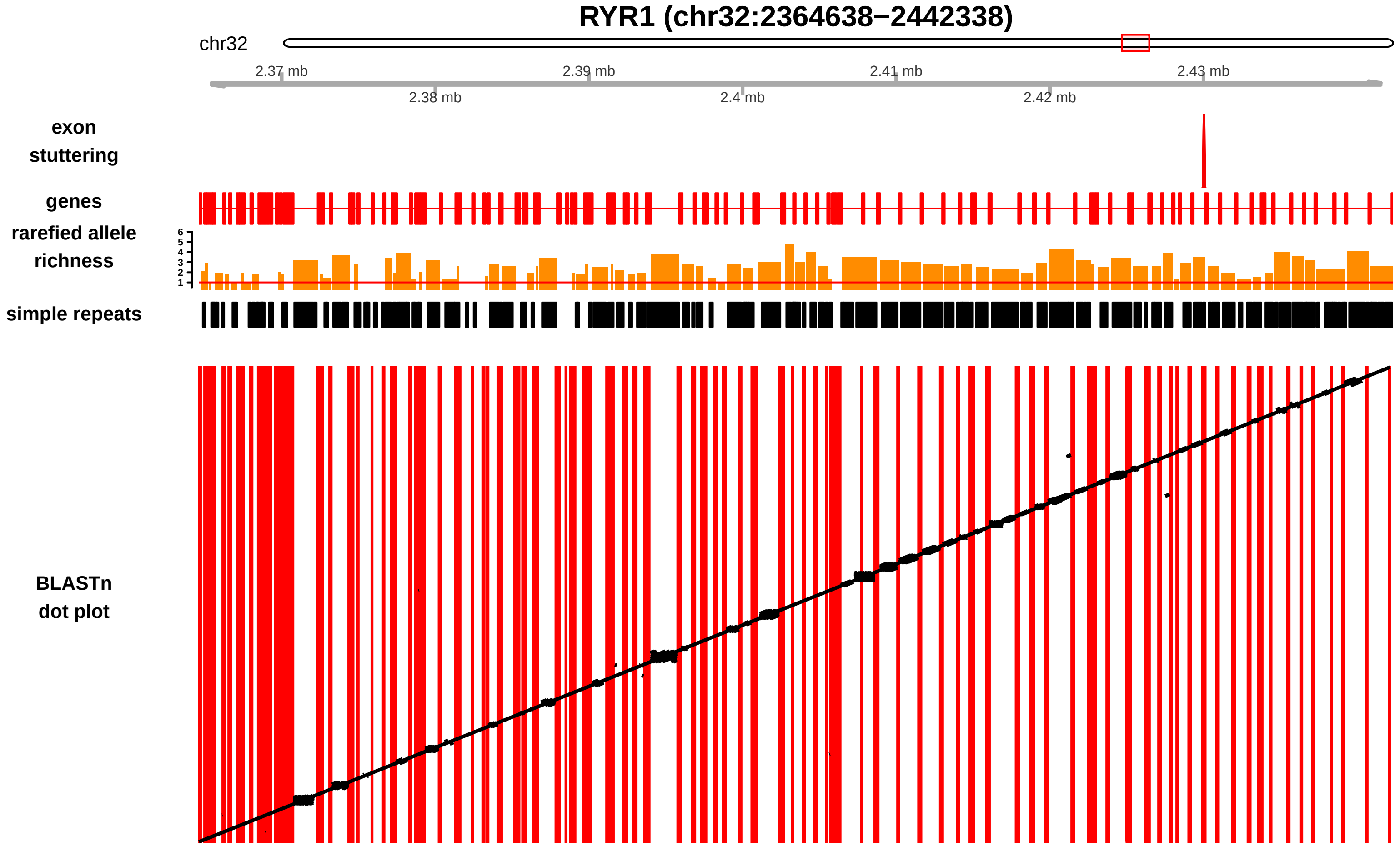
RYR1 (chr32:2364638−2442338)
chr32
2.37 mb
2.38 mb
2.39 mb
2.4 mb
2.41 mb
2.42 mb
2.43 mb
exon stuttering
genes
rarefied allele richness
1
2
3
4
5
6
simple repeats
BLASTn dot plot

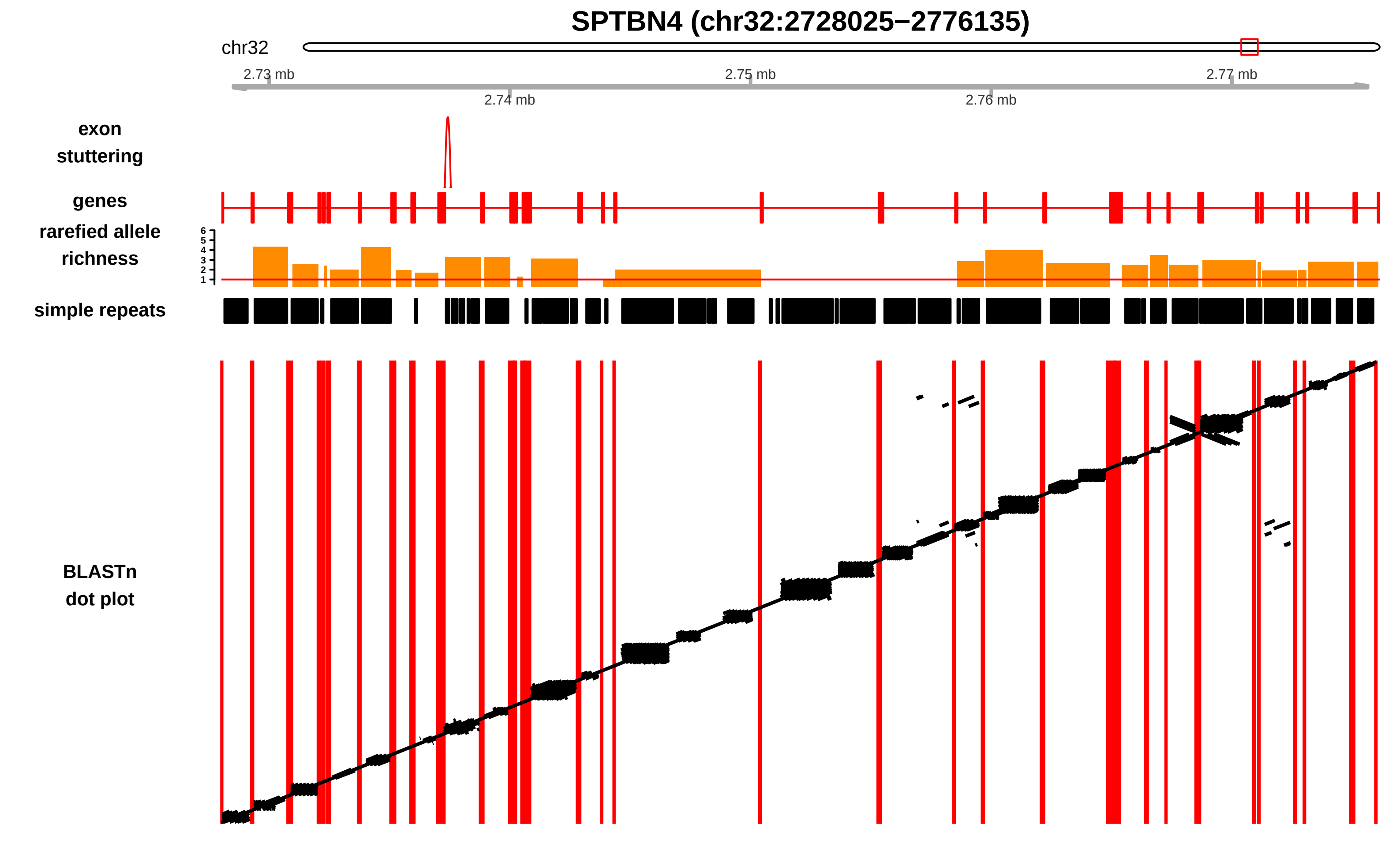
SPTBN4 (chr32:2728025−2776135)
chr32
2.73 mb
2.74 mb
2.75 mb
2.76 mb
2.77 mb
exon stuttering
genes
rarefied allele richness
1
2
3
4
5
6
simple repeats
BLASTn dot plot

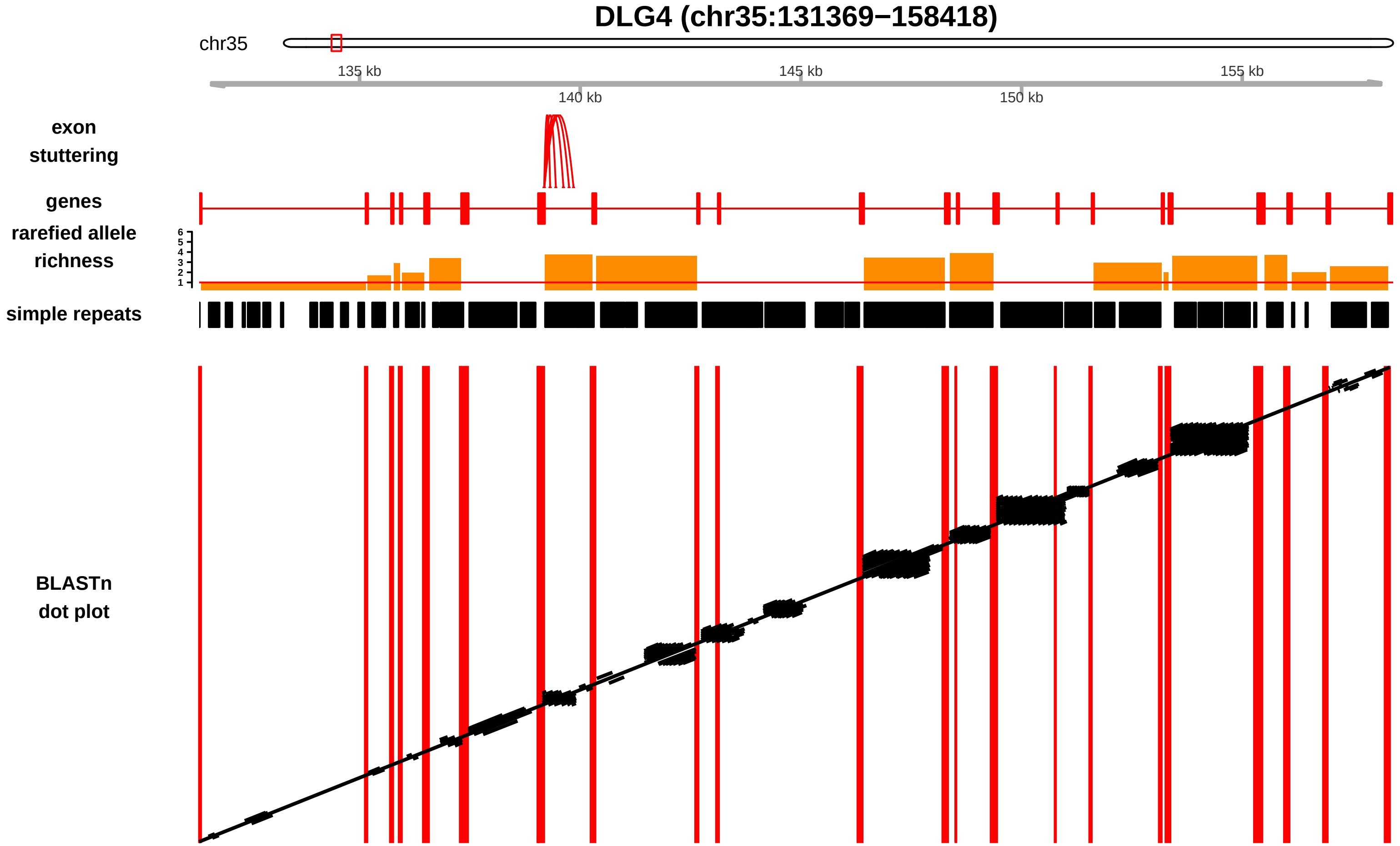
DLG4 (chr35:131369−158418)
chr35
135 kb
140 kb
145 kb
150 kb
155 kb
exon stuttering
genes
rarefied allele richness
6
5
4
3
2
1
simple repeats
BLASTn dot plot

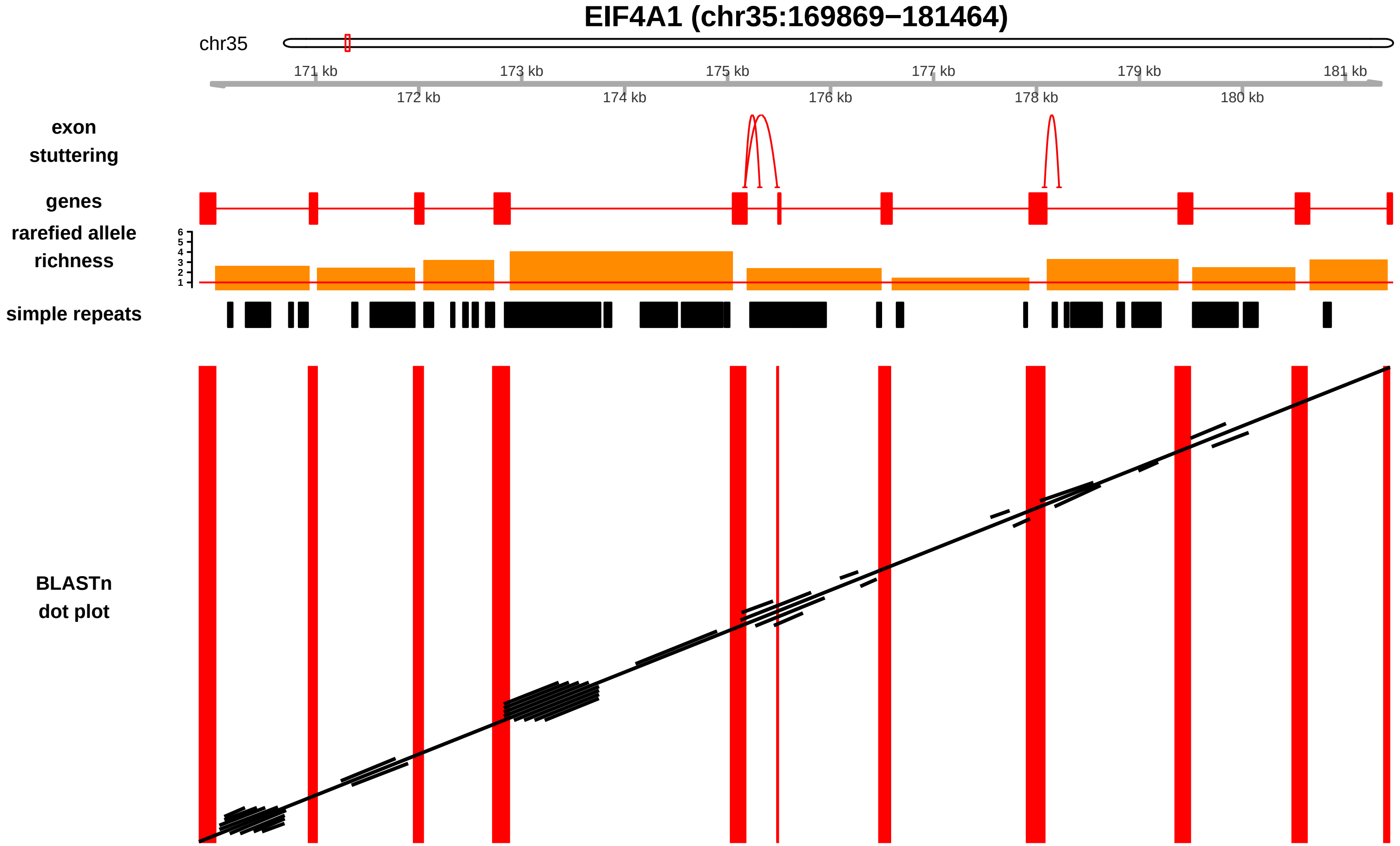
EIF4A1 (chr35:169869−181464)
chr35
171 kb
172 kb
173 kb
174 kb
175 kb
176 kb
177 kb
178 kb
179 kb
180 kb
181 kb
exon stuttering
genes
rarefied allele richness
1
2
3
4
5
6
simple repeats
BLASTn dot plot

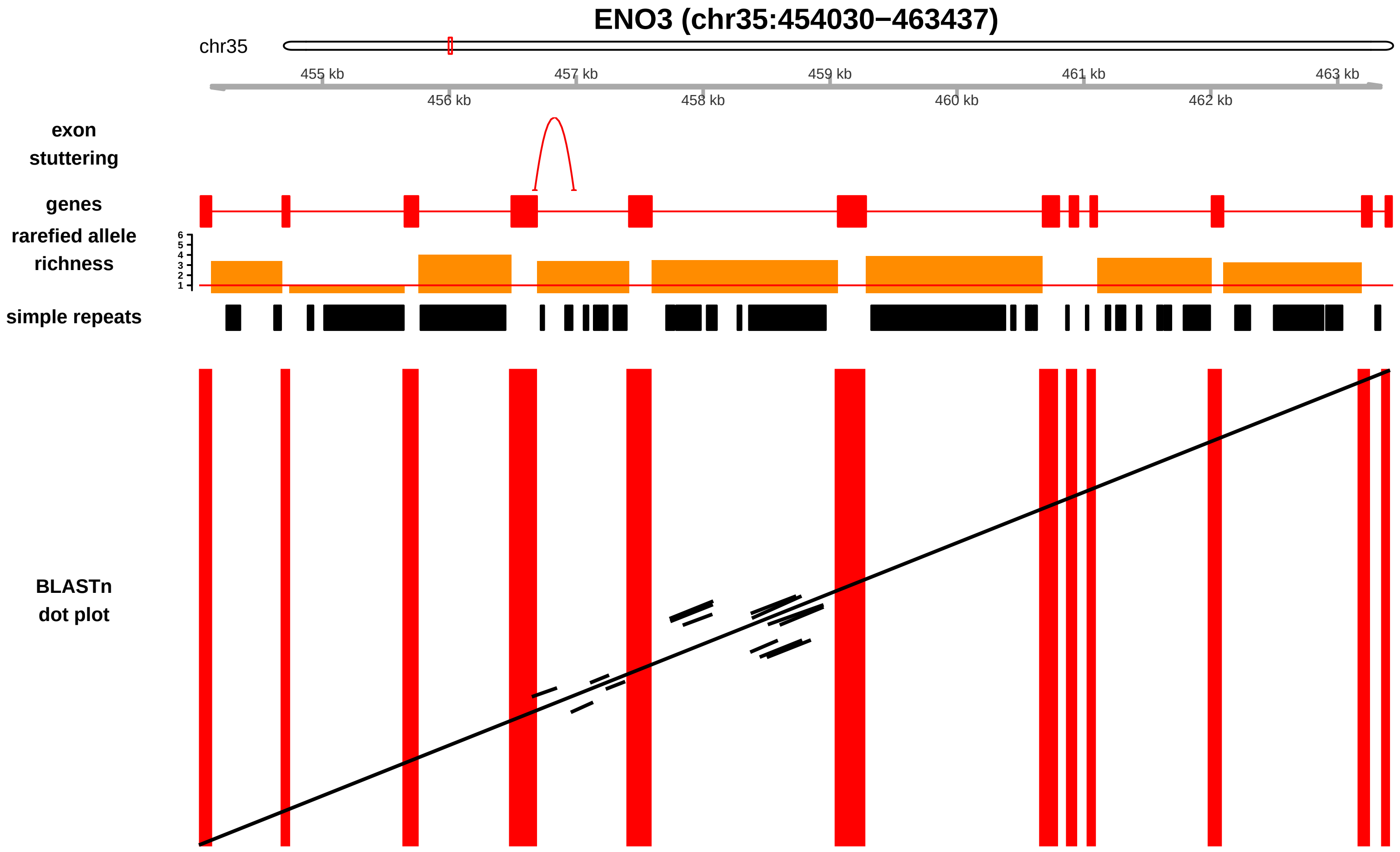

ENO3 (chr35:454030−463437)
chr35
455 kb
456 kb
457 kb
458 kb
459 kb
460 kb
461 kb
462 kb
463 kb
exon stuttering
genes
rarefied allele richness
1
2
3
4
5
6
simple repeats
BLASTn dot plot

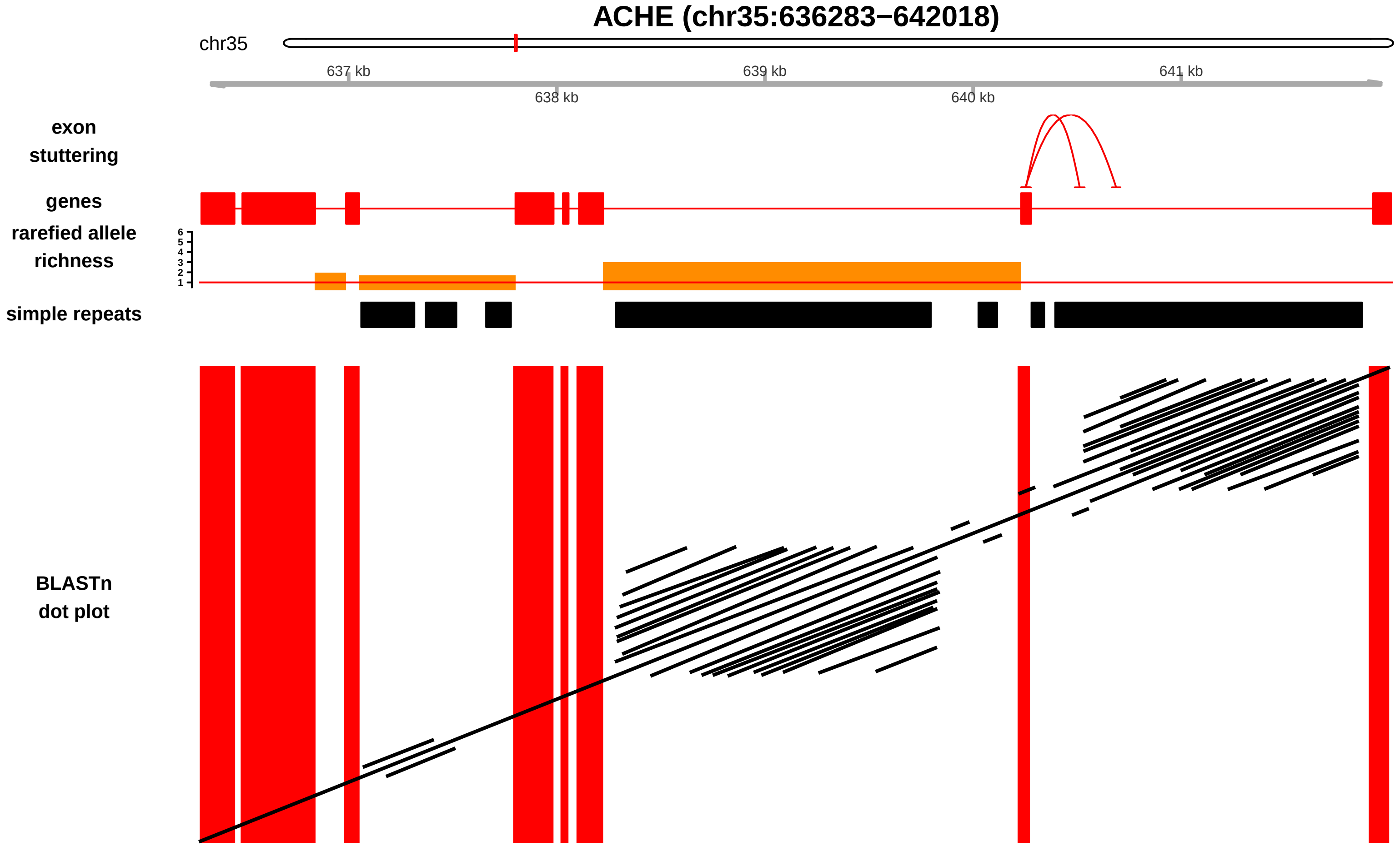
ACHE (chr35:636283−642018)
chr35
637 kb
638 kb
639 kb
640 kb
641 kb
exon stuttering
genes
rarefied allele richness
1
2
3
4
5
6
simple repeats
BLASTn dot plot

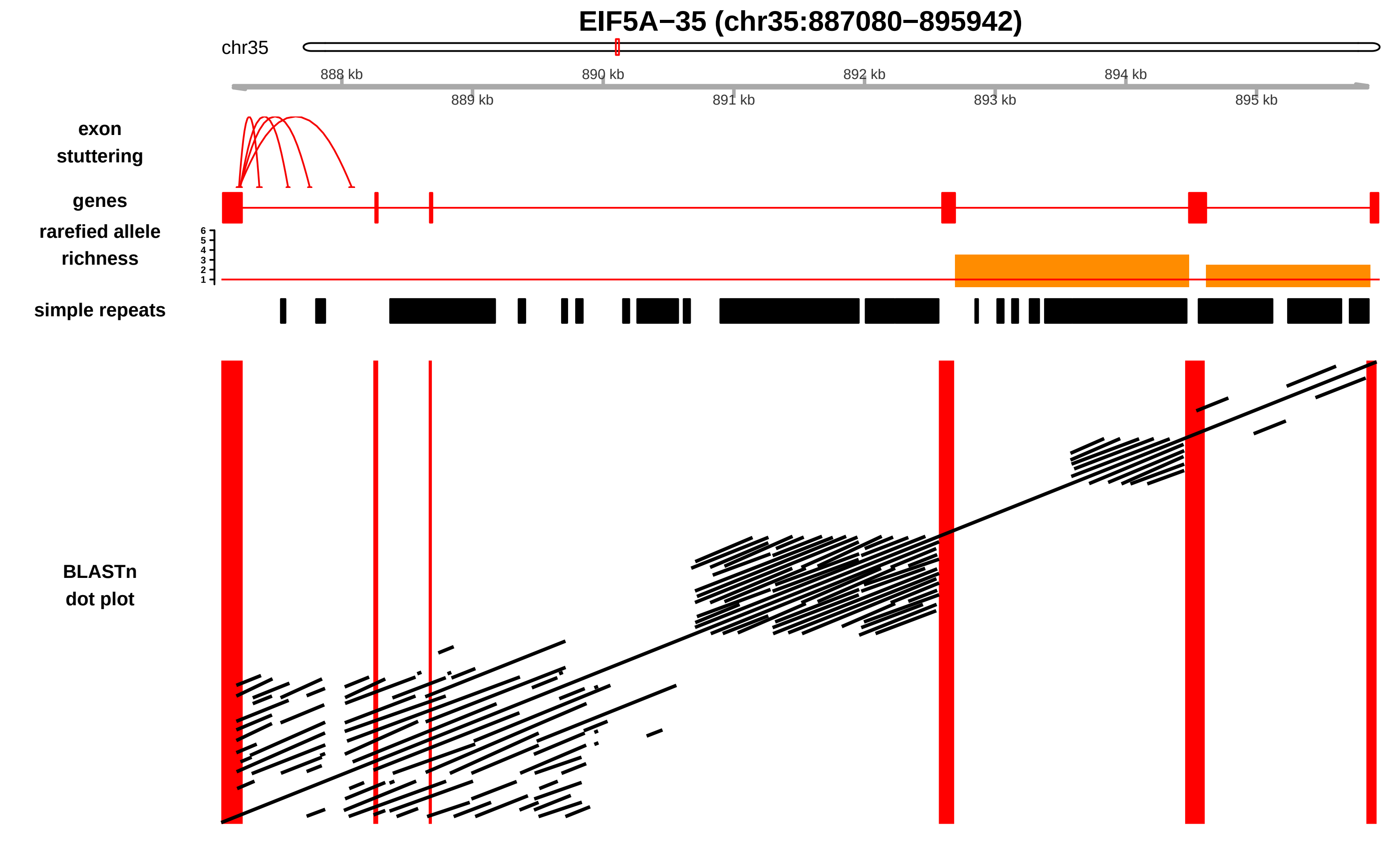
EIF5A−35 (chr35:887080−895942)
chr35
888 kb
889 kb
890 kb
891 kb
892 kb
893 kb
894 kb
895 kb
exon
stuttering
genes
rarefied allele
richness
6
5
4
3
2
1
simple repeats
BLASTn
dot plot

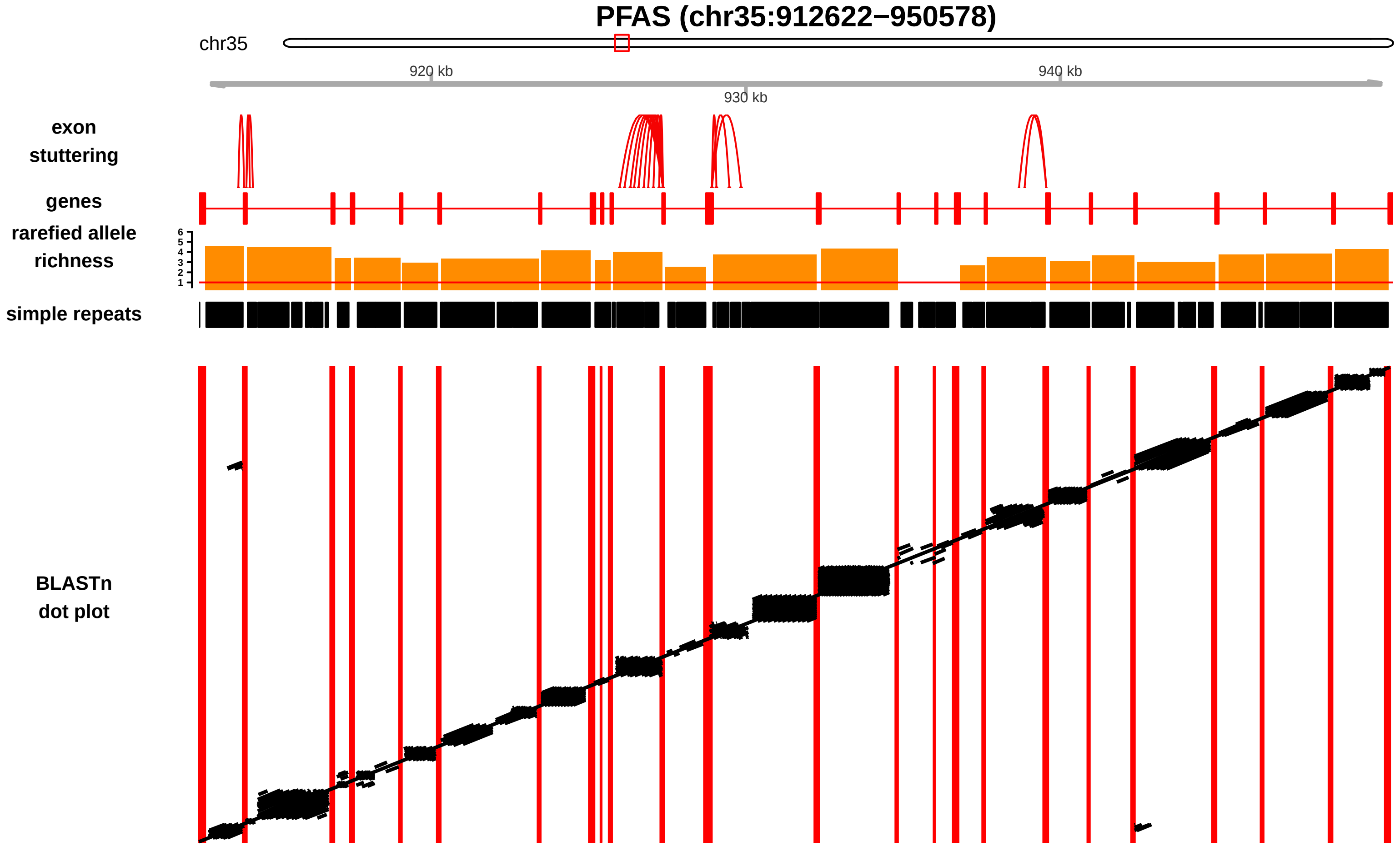

PFAS (chr35:912622−950578)
chr35
920 kb
930 kb
940 kb
exon stuttering
genes
rarefied allele richness
6
5
4
3
2
1
simple repeats
BLASTn dot plot

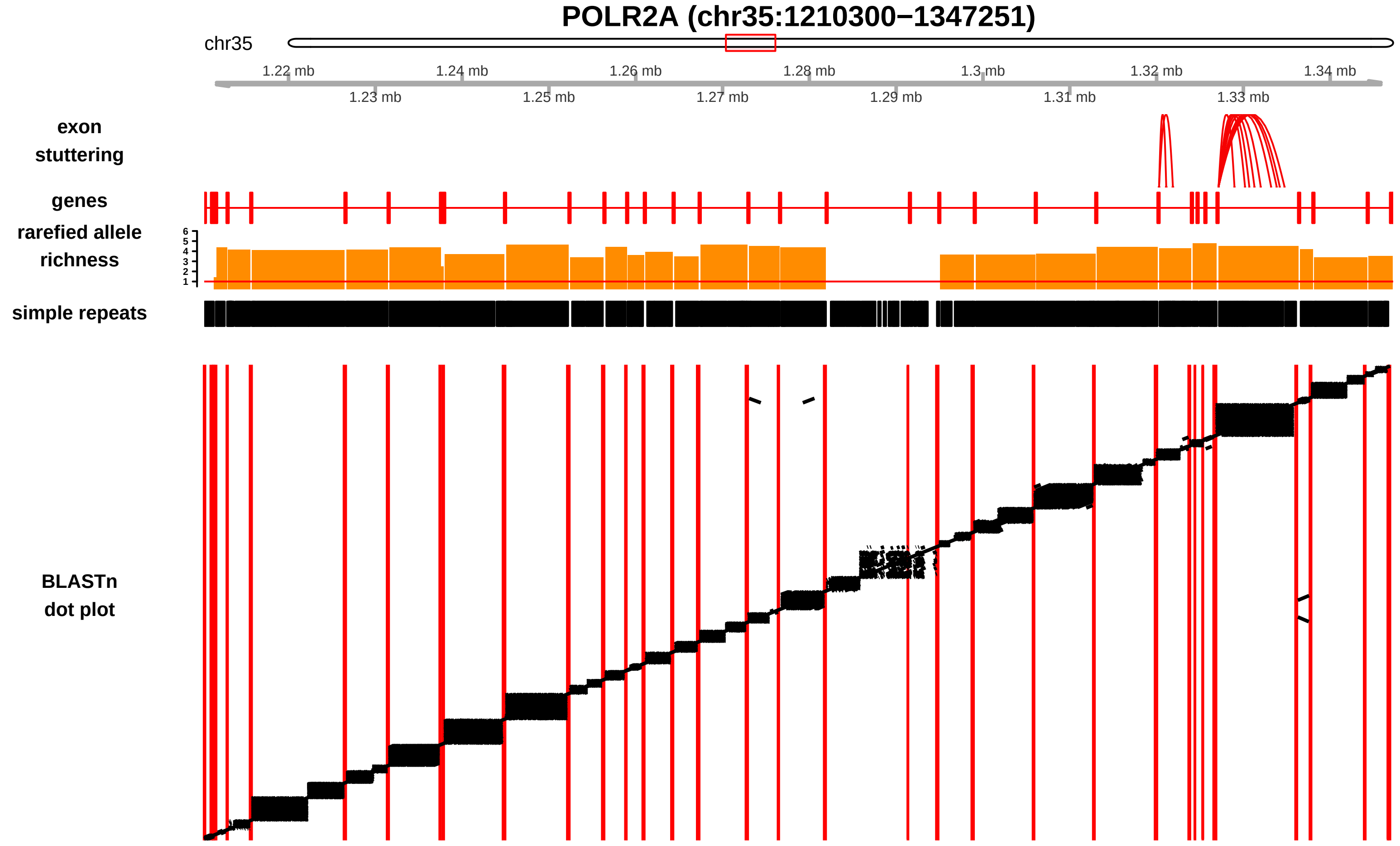
POLR2A (chr35:1210300−1347251)
chr35
1.22 mb
1.23 mb
1.24 mb
1.25 mb
1.26 mb
1.27 mb
1.28 mb
1.29 mb
1.3 mb
1.31 mb
1.32 mb
1.33 mb
1.34 mb
exon stuttering
genes
rarefied allele richness
1
2
3
4
5
6
simple repeats
BLASTn dot plot

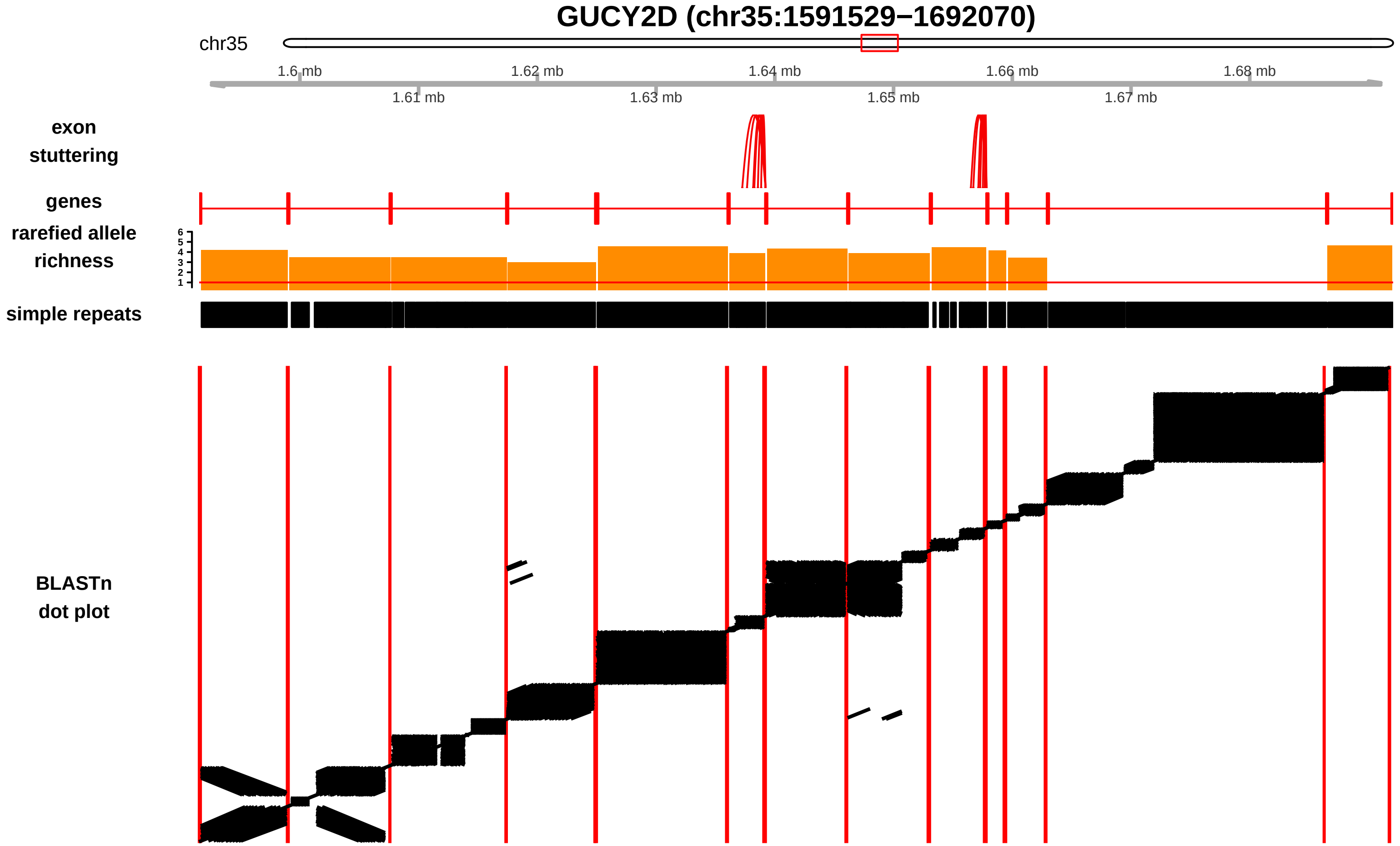
GUCY2D (chr35:1591529−1692070)
chr35
1.6 mb
1.61 mb
1.62 mb
1.63 mb
1.64 mb
1.65 mb
1.66 mb
1.67 mb
1.68 mb
exon stuttering
genes
rarefied allele richness
6
5
4
3
2
1
simple repeats
BLASTn dot plot

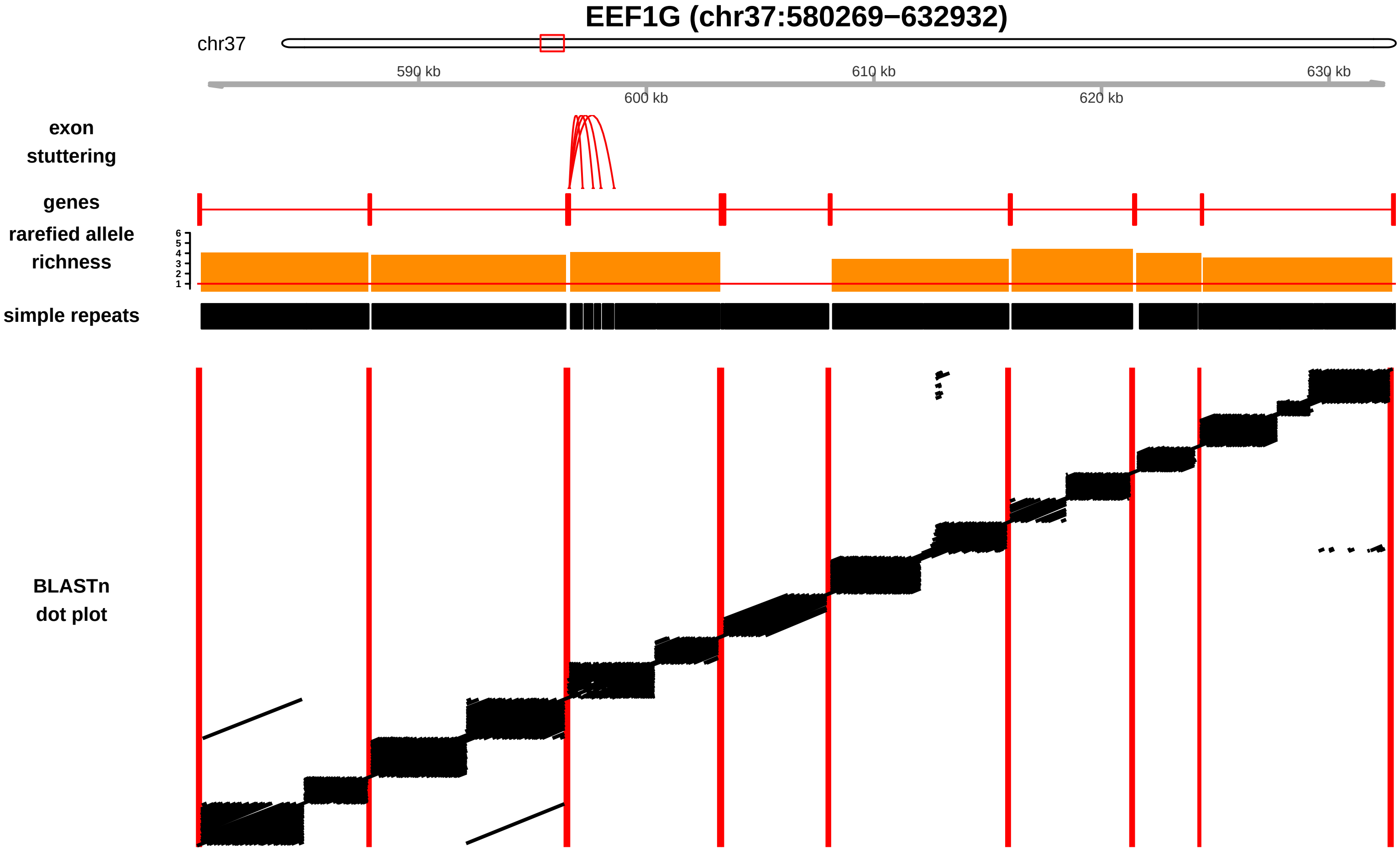
EEF1G (chr37:580269−632932)
chr37
590 kb
600 kb
610 kb
620 kb
630 kb
exon stuttering
genes
rarefied allele richness
6
5
4
3
2
1
simple repeats
BLASTn dot plot

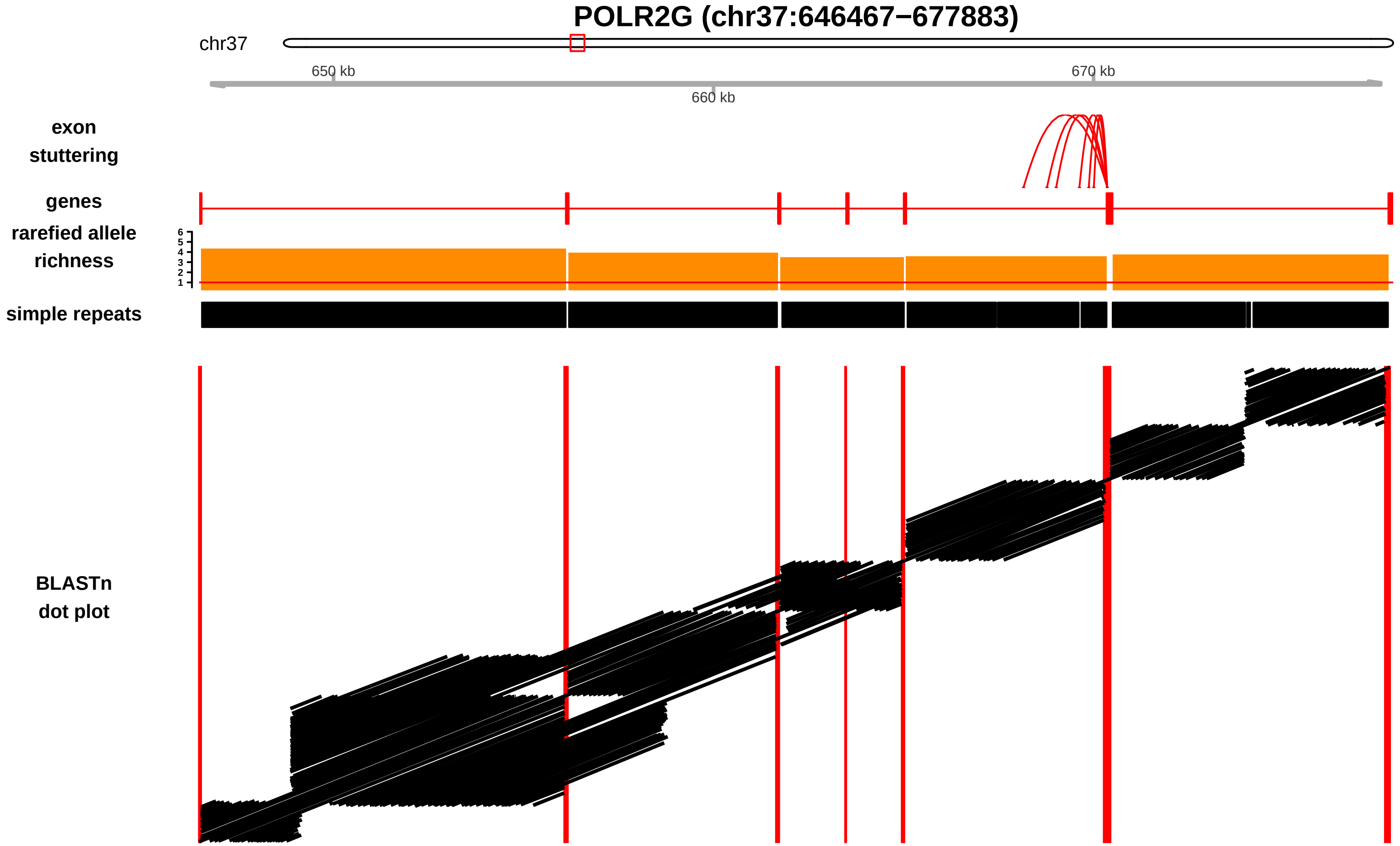
POLR2G (chr37:646467−677883)
chr37
650 kb
660 kb
670 kb
exon stuttering
genes
rarefied allele richness
1
2
3
4
5
6
simple repeats
BLASTn dot plot

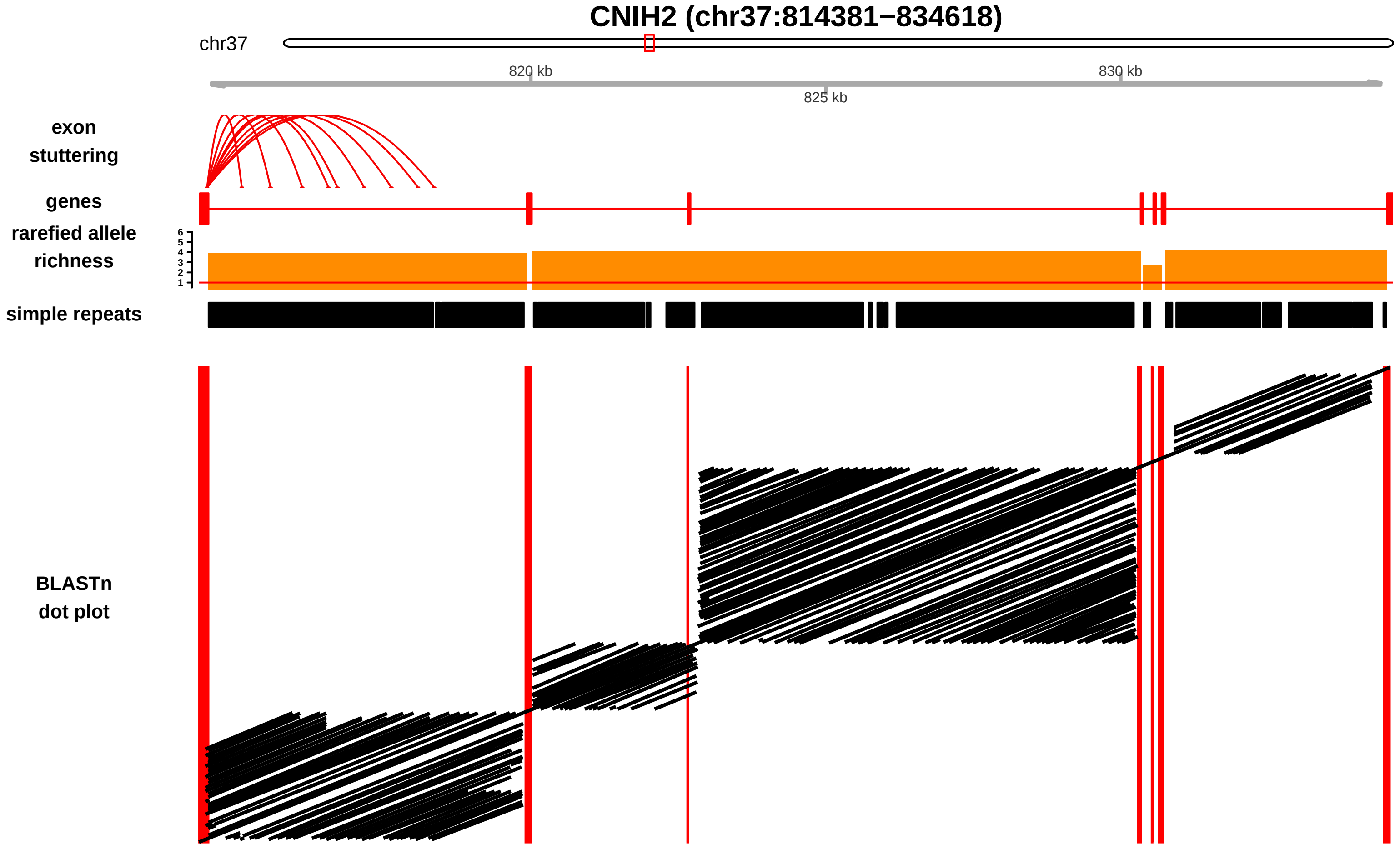

CNIH2 (chr37:814381−834618)
chr37
820 kb
825 kb
830 kb
exon stuttering
genes
rarefied allele richness
6
5
4
3
2
1
simple repeats
BLASTn dot plot

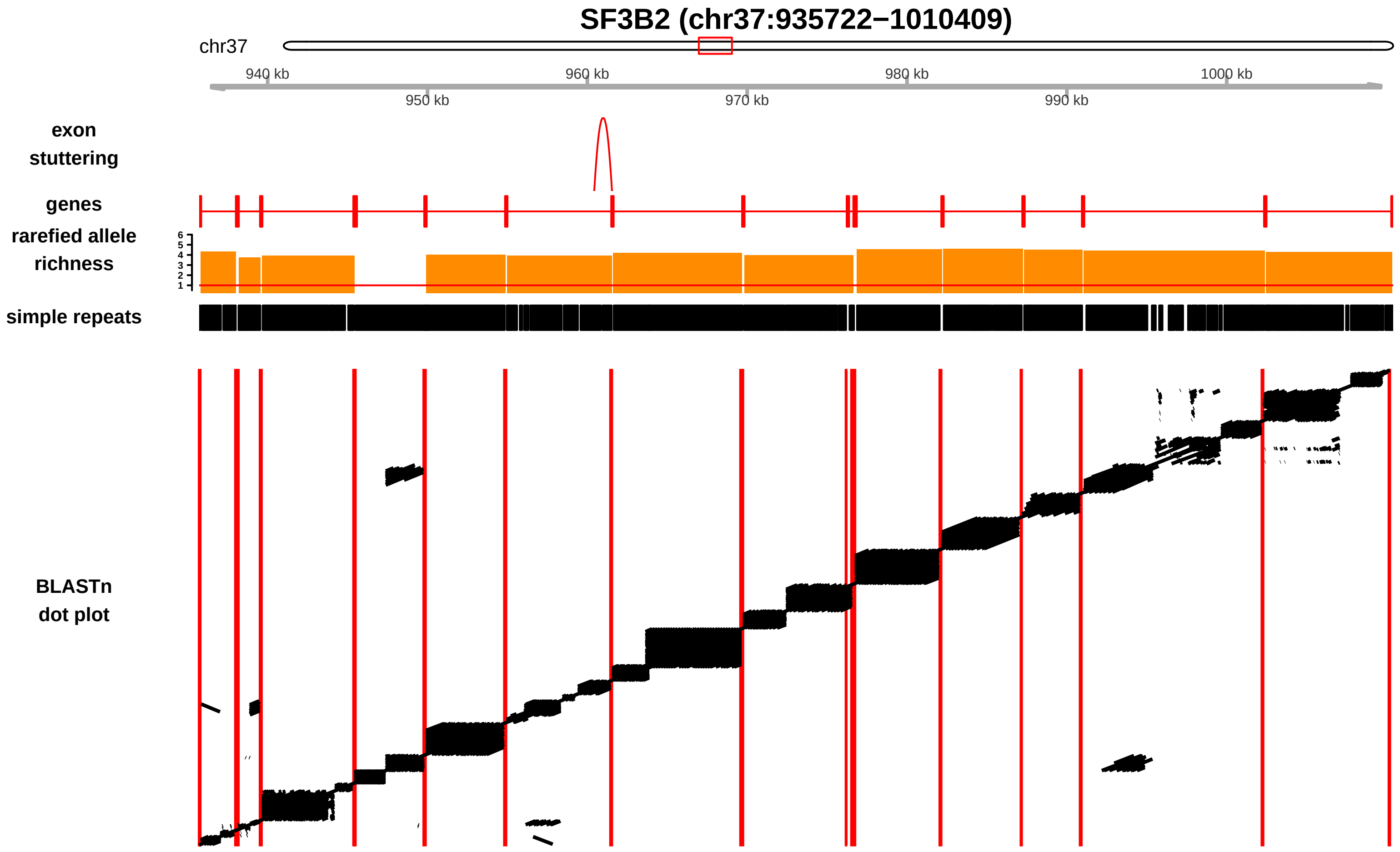
SF3B2 (chr37:935722−1010409)
chr37
940 kb
960 kb
980 kb
1000 kb
950 kb
970 kb
990 kb
exon stuttering
genes
rarefied allele richness
6
5
4
3
2
1
simple repeats
BLASTn dot plot

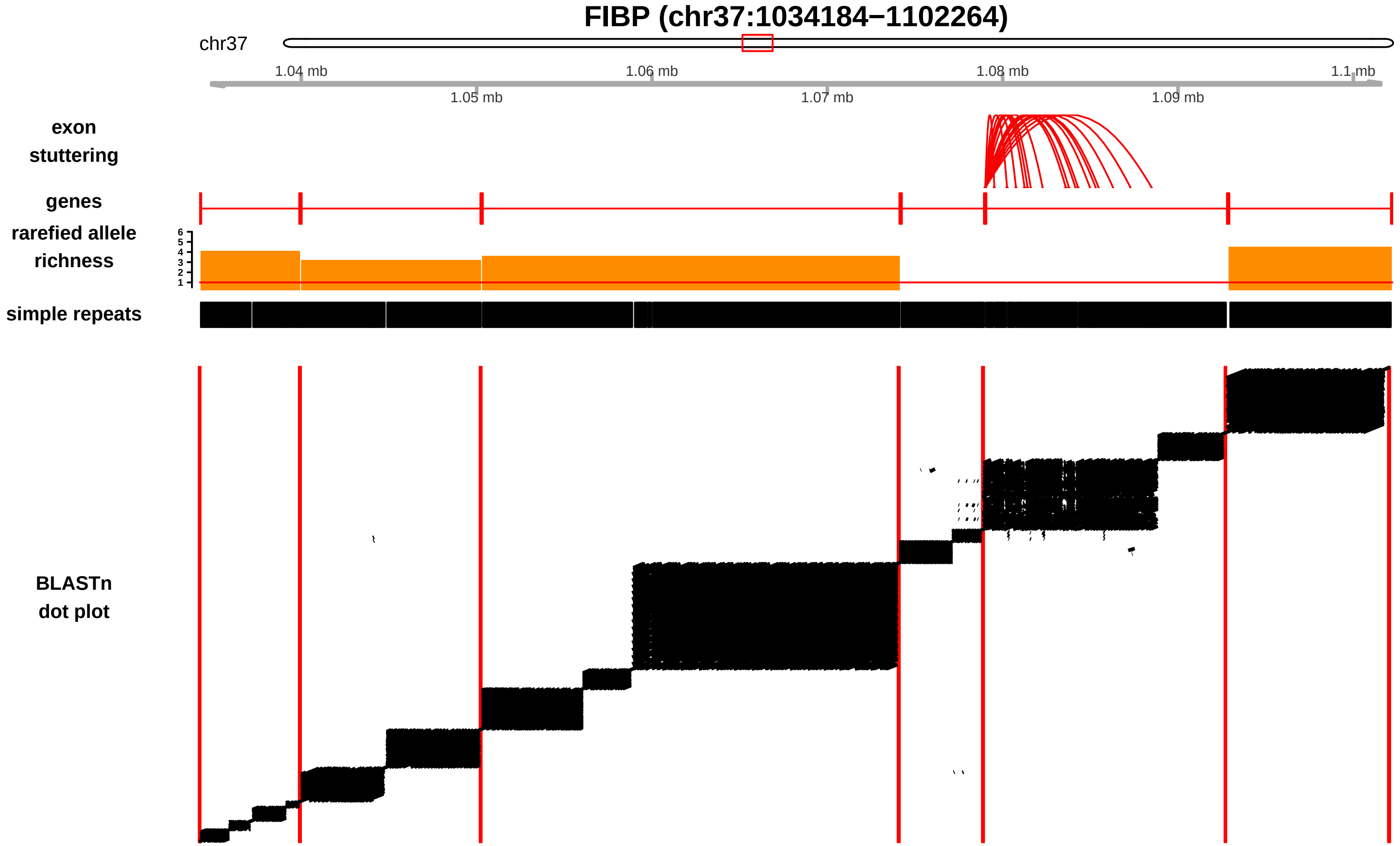
FIBP (chr37:1034184–1102264)
chr37
1.04 mb
1.05 mb
1.06 mb
1.07 mb
1.08 mb
1.09 mb
1.1 mb
exon stuttering
genes
rarefied allele richness
1
2
3
4
5
6
simple repeats
BLASTn dot plot

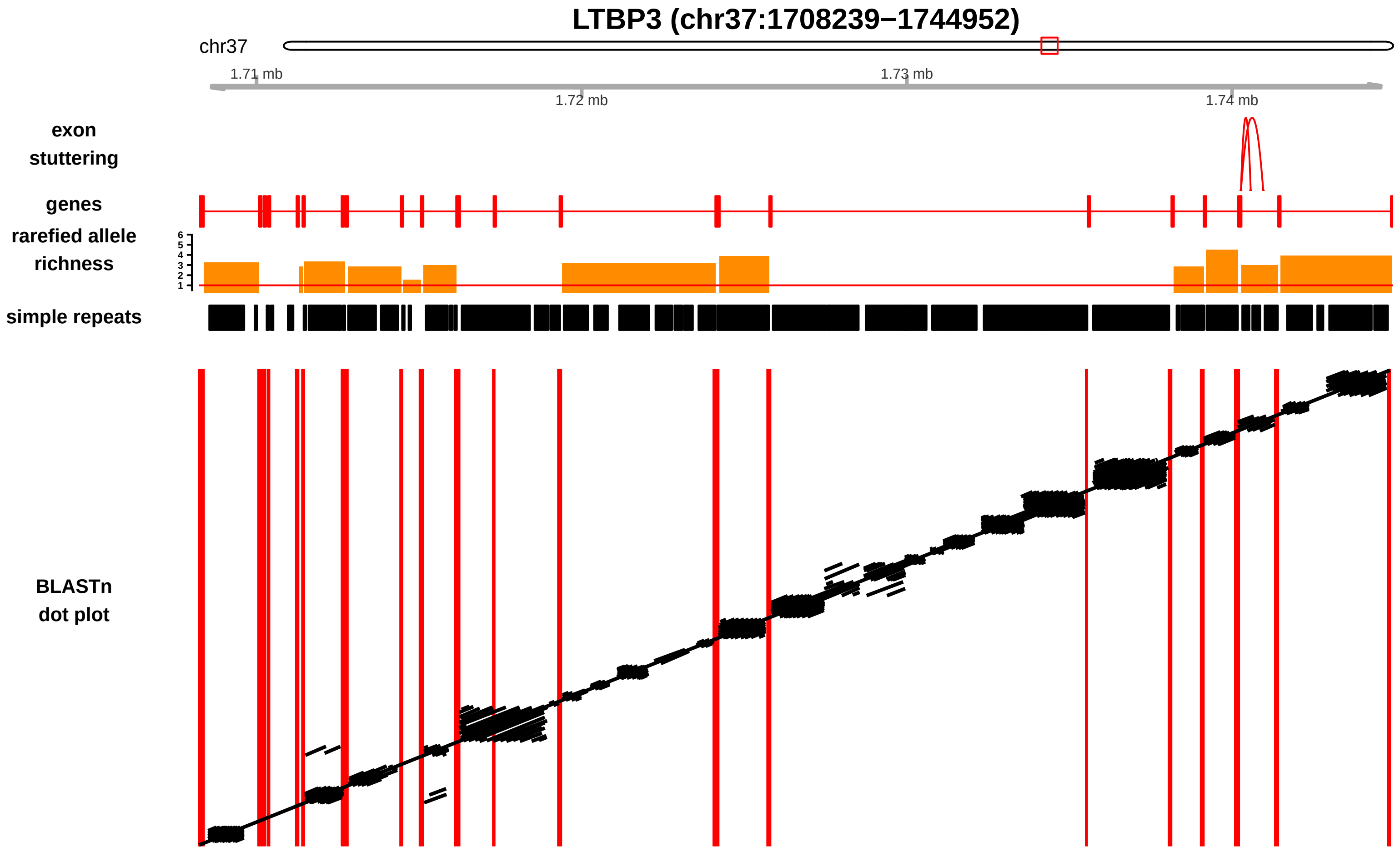
LTBP3 (chr37:1708239−1744952)
chr37
1.71 mb
1.72 mb
1.73 mb
1.74 mb
exon stuttering
genes
rarefied allele richness
6
5
4
3
2
1
simple repeats
BLASTn dot plot

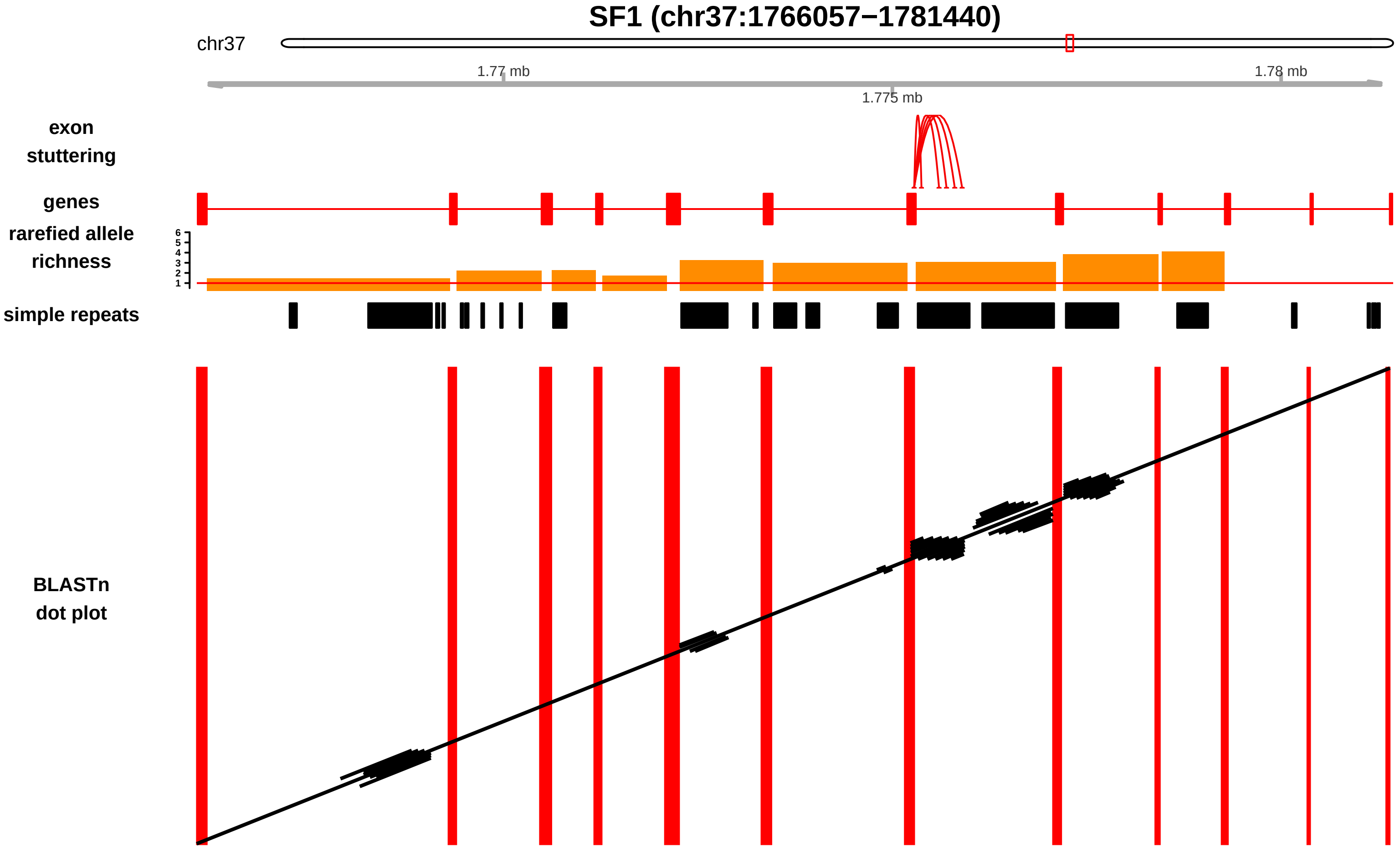

SF1 (chr37:1766057−1781440)
chr37
1.77 mb
1.775 mb
1.78 mb
exon stuttering
genes
rarefied allele richness
1
2
3
4
5
6
simple repeats
BLASTn dot plot

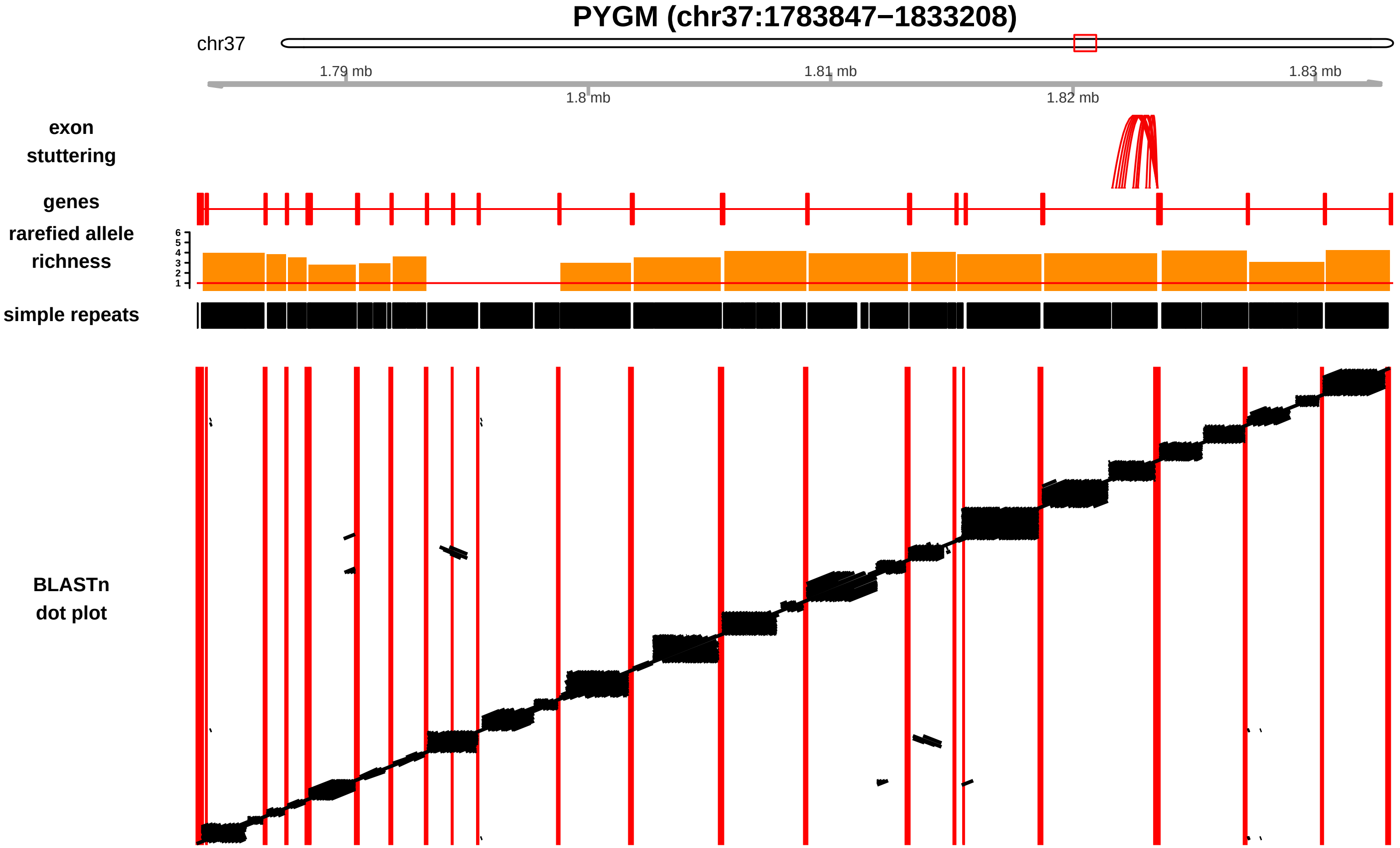
PYGM (chr37:1783847−1833208)
chr37
1.79 mb
1.8 mb
1.81 mb
1.82 mb
1.83 mb
exon stuttering
genes
rarefied allele richness
6
5
4
3
2
1
simple repeats
BLASTn dot plot

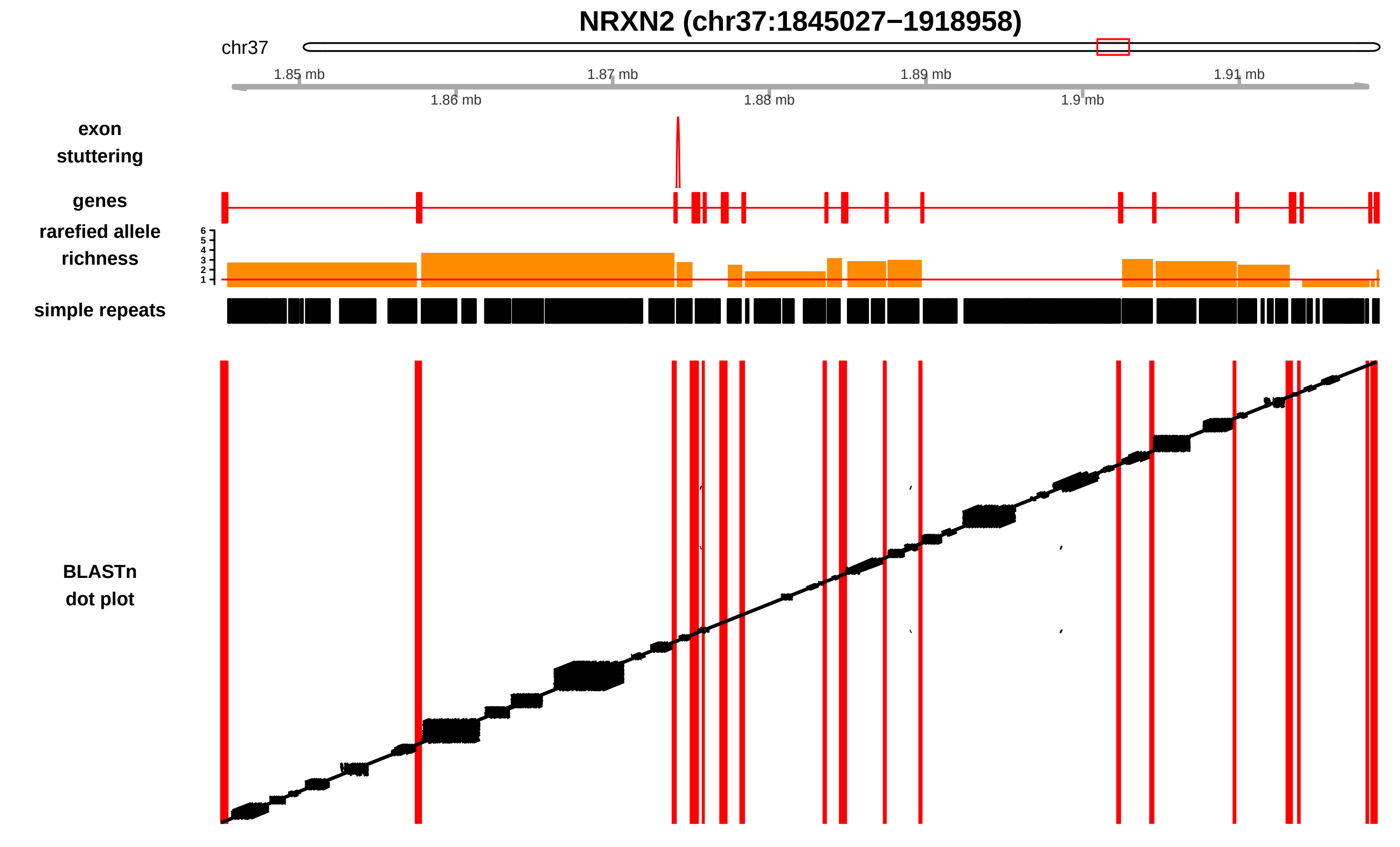

NRXN2 (chr37:1845027−1918958)
chr37
1.85 mb
1.87 mb
1.89 mb
1.91 mb
1.86 mb
1.88 mb
1.9 mb
exon stuttering
genes
rarefied allele richness
6
5
4
3
2
1
simple repeats
BLASTn dot plot

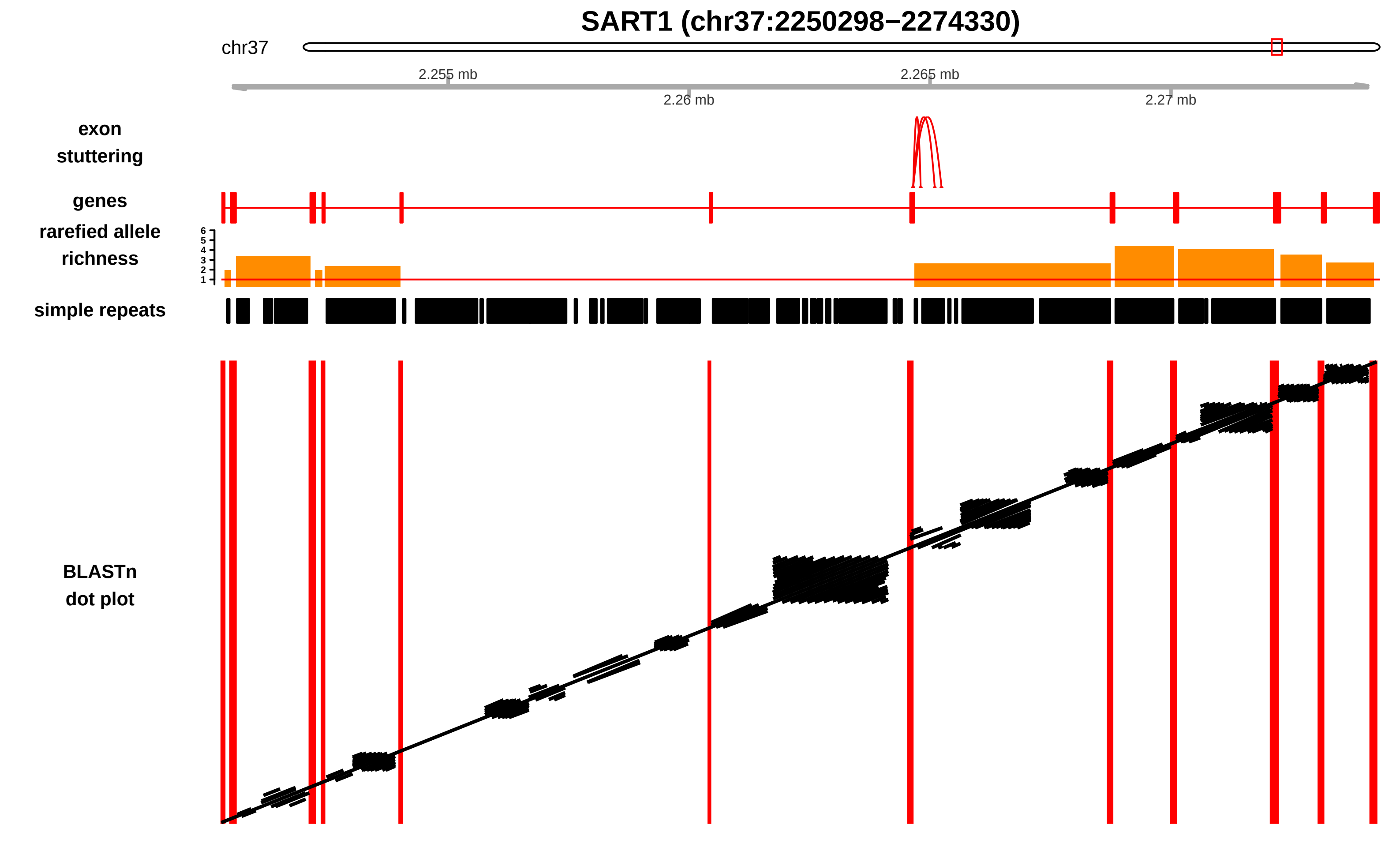
SART1 (chr37:2250298−2274330)
chr37
2.255 mb
2.265 mb
2.26 mb
2.27 mb
exon
stuttering
genes
rarefied allele
richness
6
5
4
3
2
1
simple repeats
BLASTn
dot plot

Supplementary Figure 5. Visualization of 115 chicken genes exhibiting exon stuttering. Each gene is visualized on a separate page. Gene symbol, chromosome coordinates, and position in the Ggswu genome are shown at the top of the page. The following data are visualized as genomic tracks aligned by chromosome position: exon stuttering − positions of significant hits of exon sequences in proximal introns, where each hit is connected to its source exon sequence by arcs; genes − gene annotation from the UCSC RefSeq mRNAs Track; rarefied allele richness − number of distinct length alleles detected in a cohort of chicken samples rarefied to 3 diploid individuals (n=6). The absence of bars in a particular intron indicates that data was insufficient to compute the metric; simple repeats − coverage by simple repeats; BLASTn dot plot − BLASTn alignments of genomic sequence versus itself where dark regions in the plots represent BLASTn hits, black rectangular regions therefore indicate repetitive clusters.

A

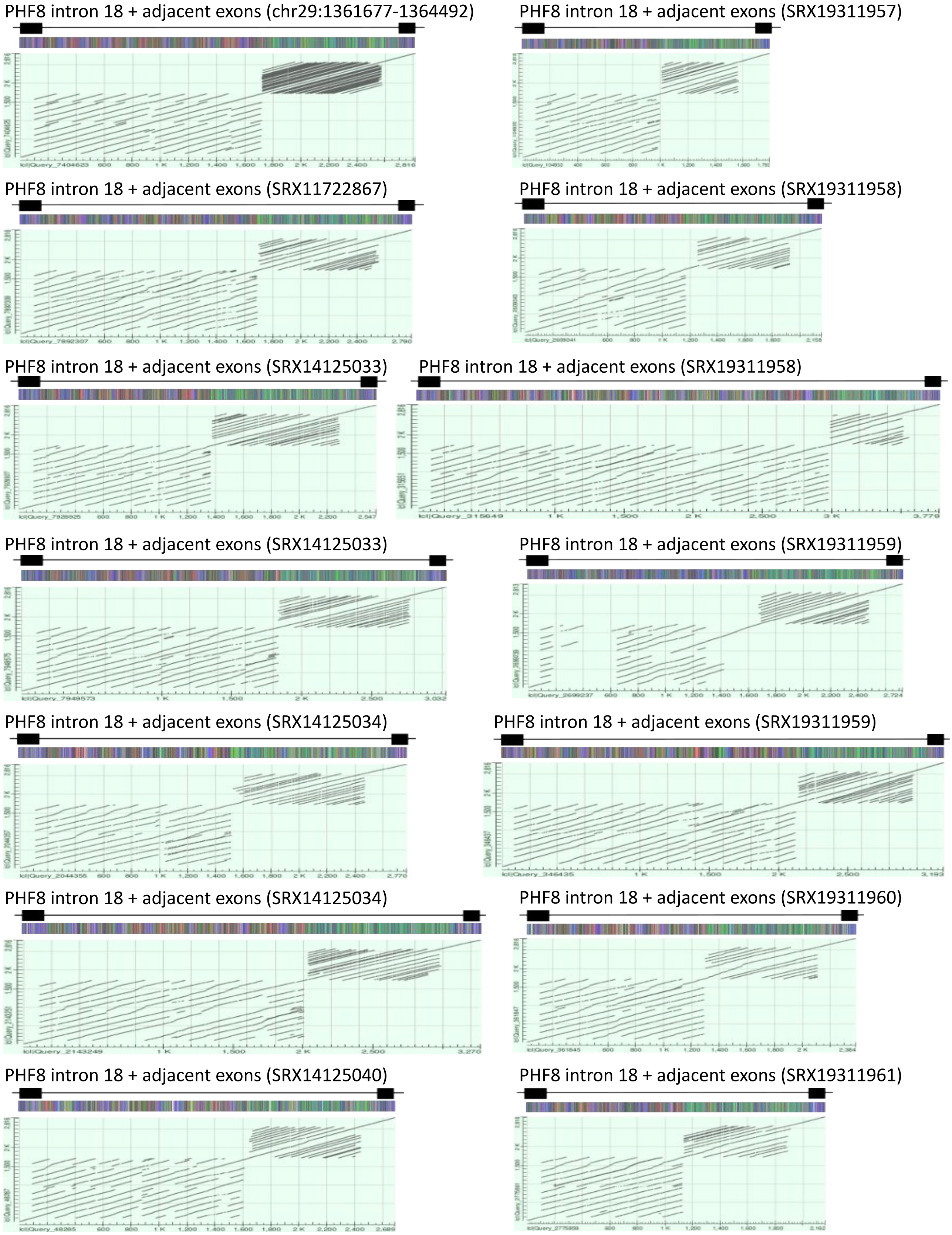

PHF8 intron 18 + adjacent exons (chr29:1361677-1364492)
PHF8 intron 18 + adjacent exons (SRX19311957)
PHF8 intron 18 + adjacent exons (SRX11722867)
PHF8 intron 18 + adjacent exons (SRX19311958)
PHF8 intron 18 + adjacent exons (SRX14125033)
PHF8 intron 18 + adjacent exons (SRX19311958)
PHF8 intron 18 + adjacent exons (SRX14125033)
PHF8 intron 18 + adjacent exons (SRX19311959)
PHF8 intron 18 + adjacent exons (SRX14125034)
PHF8 intron 18 + adjacent exons (SRX19311959)
PHF8 intron 18 + adjacent exons (SRX14125034)
PHF8 intron 18 + adjacent exons (SRX19311960)
PHF8 intron 18 + adjacent exons (SRX14125040)
PHF8 intron 18 + adjacent exons (SRX19311961)

B

>GCA_024206055.2_dnA rAnGe=CP100583.2:1361677-1364492 2541
CCACGTAGTGCTCGACGTCGCGGGGGCCGAAGGTGGGGGGGGGCAGCGTCATCCCCAGCCCCTCTTTGCGGGCCACCAGGATGGGGACACTGAAGCTCTTCTCCTCCAGGTACT
CCACCGTCAGCTGCGCCCCACTGGGCTTCAGCAGCACCTCGTCTGCGCTATGGGGCAGAGGGGGGGGACGTGGGGGGGTTATGGGGGGGTGTTATGGGGTCTATGAAGGTCTAT
GGGGGGCTATGAGGGGGTTATTGGGGGGTATGGGGGGGTATGGGGGCTCCTCCAGGTACTCCACCGTCAGCTGCGCCCCGCTGGGCTTCAGCAGCACCTCGTCTGCGCTATGGG
GCAGAGGGGGGGGACGTGGGGGGGTTATAGGGTCTATGGGGGGGTTATAGGGGGCTTTGGGGGGGTTATGGGGCCTATGGGGTCTATGGGGTCTCCTCCAGGTACTCCACCGTC
AGCTGCGCCCCACTGGGCTTCAGCAGCACCTCGTCTGCGCTATGGGGCGGAGGGGGGGACGTGGGGGGCGTTATGGGGTCTATGGGGGGGTTATAGGGGGCTTTGGGGGGGTTA
TGGGGTCTATGGTGGTCTATGGGGGCTCCTCCAGGTACTCCACCGTCAGCTGCGCCCCACTGGGCTTCAGCAGCACCTCGTCTGCGCTATGGGGCAGAGGGGGGGGACGTGGGG
GGGTTATGGGGGGTGTTATGGGGGTCTATGGGGGGCTATGAGGGGGTTATTGGGGGGTATGGGGGGTATGGGGGCTCCTCCAGGTACTCCACCGTCAGCTGCGCCCCGCTGGGC
TTCAGCAGCACCTCGTCTGCGCTATGGGGCAGAGGGGGGGACGTGGGGGGCGTTATGGGGGGTGTTATGGGGGGGTTATAGGGGGCTTTGGGGGGGGTTATGGGGTCTATGAAGG
TCTATGGGGGGCTATGAGGGGGTTATGGGGGGGTATGGGGAGGTATGGGGGGTTATGGGGGGGTTATGGGGTCTATGGGGGAGTTATGGGGTTGATGGGGGGTATGGGGTCTCC
TCCAGGTACTCCACCGTCAGCTGCGCCCCGCTGGGCTTCAGCATTACCTCGTCTGCGCTATGGGGCAGAGGGGGGGGACATAGGGGGGGTTATGGGGGGCTATGGGGGGGTTAT
GGGGGGCTTTGGGGGGGTTATGGGGTCTATGGGGGCTCCTCCAGGTACTCCACCGTCAGCTGCGCCCCACTGGGCTTCAGCAGCACCTCGTCTGCGCTATGGGGCGGAGGGGGG
GACGTGGGGGGGCATTATGGGGTCTATGGGGGGGTTATAGGGGGCTTTGGGGGGGTTATGGGGTCTATGGTGGTCTATGGGGGCTCCTCCAGGTACTCCACCGTCAGCTGCGCC
CCACTGGGCTTCAGCAGCACCTCGTCTGCGCTATGGGGCAGAGGGGGGACATGGGGGGGTTATGGGGGTCCATGGGGGGCTATGGAGGGGGGTTATGGGGTCTATGGGGGGGT
TATGGGGTCTATGAAGGTCTATGGGGGGGCTATGAGTGGGTTATGGGGTCTATGGGGGGCTATGGGGGGCTATTGGGGGTATGGGGGCTCCTCCAGGTACTCCACCGTCAGCTGC
GCCCCGCTGGGCTTCAGCAGCACCTCGTCTGCGCTATGGGGCAGAGGGGGGACATGGGGGGGTTATGGGGGTCCATGGGGGCTATGGGGGGCTATGGGGGGGTTATGGGGTCT
ATGGGGGGCTATATGGGAGAAACCGTATAGGGGAACGAGAAACCCTACAGGGGAACGAGAAACCCTATAGGGGAACGAGAAACCCTACGGGGGAATGAGAAACCCTACAGGGGAA
TGGGAAACCCTACAGGGGAACGAGAAACCCTACAGGGCAATGAGAAGCCCTACAGGGGAACGAGAAACCCTACGGGGGAACGAGAAAGCCCTACAGGGGAACGAGAAACCCTACA
GGGCAATGAGAAGCCCTACAAGAGAATGAGAAACCCTACAGGGGAATGGAAACCCTACAGGGGAACTAGAAACCCTATAGGAGAATGGAAACTCTACAGTGGAATGGAAAACCC
TACAGGGCAATGGAAACCCTACAGGGCAATGGAAACCCTACAAGGAAATGAGAAACCCTACAGGAGAATGGGGAACCCTACAGGGGAATGGGGAACCCTACAGGGGAACGAGA
AACCCTACAGGGGAACGAGAAACCCTACAGGGGAACGGGAAACCCTACAGGGGAACGAGAAACCCTACAGGGGAACGAGAAACCCTACAGGGGAACGGGAAACCCTACAGGGGA
ACGGGAAACCCTACAGGGGAACGGGAAACCCTACAGGGGAACGAGAAACCCTACAGGAGAATGGGAAACCCTACAGGAGAACGGGAAACCCTACAGGGGAATGGGAAACCCTACA
GGAGAATGAGAAACCCTACAGGGGAATCAGAAACCCTACAGGGGAATGGGAAACCCTACAGGGGAACGGGAAACCCTATAGGAGAATGGAAACTCTACAGTGAAATGGAAACCC
TACAGCGAAATGAGAAACCCTGCAGGGGAATGGGATACCCTATAGAGGAACGATAAACCCCATAAGACAACAAAGAGATTTACGGGGAAACAAAAGACCCCATAGGGGATCAGCCG
ACCCTATAAGAACCTGAACGACCCCACAGAGGAGTATGGGACCCTATAAGCGCCCTATAGGGTCGCCTCACCTGGGGAAGGTGCGGCCCCGCAGCTCCCGCACGAACTGAGCGCT
GCCGGTCCTTATGGGGCGCCCCACATCCGCGTCCCCCGCAGCGCCTTTGGGGGCGGCCCGGCGCCGCTTCA
>SRX11722867
CACGCAGTGCTTCCACGTCGCGGGGCCGCATGTGGGGGCAGTCATCCCCAGCCCCTCTTTGCGGGCCACCAGGATGGTAAGGACTTACCAGACTTTTTTCCTCCAGGTACTCCACC
GTCAGCTGCGCCTACTGGGCTTCAGCAGCAGTACCGTCTGCGCCATGGGGCAGAGGGTGGGGACGTGGGGGGATGCAGGGGGGCAGGGGCCTATGAAGGCTCATGGGGCTAAG
AGGGGTTATTGGGGGTCAATGGGGGGGTAAGGGGGTTTCTCCAGGTACTTCCACTCATCAGCTGCGCCTCGCTGGGCTTCAGCAGGCACCTGCGCCCGCGCTATGGGGCAGAGG
GGAGGACGTGGGGGGTTATAGGGGCCTATGTGGGGTTATAGGGGCTTTGGGGTTATGCATGCAGGGGCCTATGGGGCTCATGGGTTCTCCGTGTGCTCCACCTGTTGCTGCGCCT
ACTGGGCTTTCAGCACTGCCTCTCGGGCGCTATGGGGGCTAGAGGGGGACGGGAGAGGCAGGCTCATGGGTCTGGGGGTTATAGAGGGCTTTGGAGGGTTATGGGGTCTATGGT
GTGGCTCATGGGGTTTCTCCAGGTACTCCACCGTCAGCTGTGTTCCACTGGGCTTCAGTAGCACTCGTCTGCGCCATGGGGCAGAGGGGGACGGGGGGGTTATGTGGGGGTGTTA
TGGGGGGCTCATGGGGGGCTATGAGGACCCAAGGTTATTGGGGGTATGGGGGCAGCGGGGTTTCTCCAGGTGCTCCACTGATCAGCTGCGCCCCGCTGGGCTTTCAGCAAAACCT
TGCCCGCGCTAAGGGGGCAGAGGGGGACGCGGGGCGCAGGGGGTGTTATGGGGTTATAGGGGCTTTGGGGTTATGGGGTCATGAAGGCTCATGAAAGGTGAGGGGGTTATGGG
GGGGGTATGGGGAGGCAATAAGGGGGTGGGGGGTTATGGGGAAGCAGGCTCATGGGAGAGTTATGGGGTTGATGGGGGGTATGCGGGGTCTCCTCCAGGTACTTCACTGTCAAC
AGGCGCTCCGCTGCGCTTCAGCATTAACTGGCCCGCGCTTATGGGGCAGAGGGGGGACATAAGGGGGTTATGGGGGGGGGCTATGGGGGGTTATGGGGCTTTGGGGGTTATGGG
GTCTATGGGGCCTTCCAGAGTGTACTTCATCATCAGCTGCGCCCCAATTGGCTTCAGCAGCACCTTGTCTAAGCTAAGGGGGCGGAGGGGGACGGGGGCATTATGTCTATGGGGGT
TATAGGGAGCTTTGGGGGGTGTAAGATGCTATGGGGCCCGTGGCCTGCGGCCTCTCCCAGGCAGCCCACCGTCAGCGGCGCCCACCAAGCTTCAGCAGCACCTCGTCTGGGTGC
GCCATGGGGCAGAGGGGAGACATAAGGGGGTTATGGGGGTCCATGGGGCGTTATGAGGGCAGTGGGCTCATGGGGGGGTTATGGGGTCTATGAAGGCTCATGGAAGCGGTGAGT
GGGTTATGTCTATGGGGGGGCTATGGGGGGCTATTGGTGGGGGTATGGGAGGTTCTCCAGGTACTCCACTCACCGCTGCGCCCGCTAAGCTTCAGCAGCACCTTGTCTGCGCGAT
GGGGCAGAGGGGGGACATGGGGGGTTGGTGGGGGTCCATGAAGAGGGGCTATGGGGCTATGGGGGGGTTATGGGGTCTATGGGGCTATATATGGGAGAAACTGCATAGGGGAA
CGAGAAACCCCACAGGGGGAACTAGAAACCCTATAGGGGAACGAGAAACCCCACGGAATGAAGAGAAACCCTACAGGGAATGGAAACCCTACAGGGGAACGAGAAACCCTACAG
GGCAAATGAGAAGCCCTACAGGGGAACTAGAAACCACAGGGCAATGAGAAGCTCAGGGAACGAGAGAACCTCACAGGGCAGCAAATGAGAAGCTCTACAAGAGAATGAGGGAAAC
CCTACAGGGGAATGAAACCCTACAGGGAACTAGAAGCCTTACAGAGAATGGGAAACCCTATGGATTGAATGGAAAGCCCTACAGGGCAATGGGAAACCCTACAGGGCAATGGGAAA
CCCTACAAGGAAATGGGAGAAACTCACAGAGAATGGGGAACCCTGCAGGGGAATGGGAAGACCCTACAGGGGACACGAACCTCCACAGGGGAACGAGAAACCTACAGGGGAACG
GGAAAGCCCTACAGGGGAACGGTGAACTCCTACAGGGGAACGAAACCCCACAGGGGAACGGGAAACCCTACAGGGGAACGGAACCTCACAGGGGAACGGGAAACCCTACAGGGG
AACGAGAAACCCTACAGGAGAATGGGAAACTTACAGAGAACCCGGGAAACCCACAGGGGAATGGGAAACCCTACAGGAGAATGAGAACCCTACAGGGGAATCAGAAAGCCCCACA
GGAATGTGGAAAACCCTACAGGGGAATGGGAAACCCTATAGGAGAATGGGAAACTCTACAGTGAAATGGGAAACCCTACAGGCGAAATGAGAAACCCTGCAGGAATAGTGGGATAC
CCTATAGAGGAACGTGATAAACTTTACAAGACAACAAAAGAGATTTACGGGAAACAAAAGACCCCCATAGGGGATCAGCCGACCCTATAAGAACCTGGAACGACCACAGAGGAGGTA
TGGGACCCTATAATCTTGCGCCCTATAGGGTCGCCTCACAACTGGGAAGGTGCGGCCTCCAGCTCTGCAGACGAATCTCGAGCGCTGCCGCCCCTTAAGGGCGCCTCACATCCGC
CTCCCCGCCAGCGCTTTGGGGCGCCCAGCGCCGCTTCA
>SRX14125033_1
CACGTAGTGCnCTCTGGACGTCnGCGGGGGCCGAAGGTGGGGGGGCAGCGTCATCCCCAGCCCCCTTCTTGCGGGCCACCAGGATGTGGGACACTGAAGCTCTTTCTCCTCCAGG
TACTTCCACCGTCAGCTGCGCCCCATGGGCTGGGCTTCAGCAGCACCTCGCTCTGGGCGCTTATGGGGCAGAGAGAGGGAGGGGGGACGTnGGGGGGTTATGGGGAGTCCATGG
GGGACTATGGGGGCGnnnTATGGAGGGGGTTATGGGGGGCTTTGGGGGGTTATGGGGTCTATGGGGGnCTCCTCCAGGTACTCCCACCGTCAGCTGCGCGCCCGCTGGGCCTTCA
GCAGCACCTCGTCTGCGCnTATGGGGGCAGAGGGGGGGGACGTGGGGGGTTATAGGGTCTATGGGGGGGGGTTATAGGGGGGGCTTTGGGGGGGGTTATGTGGGGTCTATGGTG
GTCTATGnGGGGCTCnCTCCAGGTACCATCCACCGTCAGCTGCGCCCCGCTGAGGCTTCAGCAGCACCTGGnCGTCTGGCGCnTATGGGAGGCAGAGGGGGGGnGnGTGGGGGTT
ATAAGGGTCTATGGGGGGGGTTATAGGGGGGCTTTGGGGGGTTATGGGGTCTATGGTGGTCTATGGGGGCTCCCTCCAGGTACTCCACACGTCGGGnGCTGCGCCCCGCATGGG
CTTCAGCAGCACCCTCGTCTnGCGCTTATGGGGCGGAGAGGGGGGACGTGGAGGGGTTATGGGGGGGGTGTTATGGGGTCTnATGAAGGTCTATGGGGGCCnATGGnGGGGGGTT
ATTGGGGGGGTATGGGGGGCTCCTCCCTAAGTACTCCACCGTCAGCTGCGCCCCCGCTGGGCTTCAGCAGCACTCCTCGTCTGCGCTATGGGnGGCGGAGGGGGAGCCATGGGG
GGGGTTATGGGGTCTATGGGGAGGTTTATGGGGGTCTATGAAGGTCTATGGGGGnnGCTGATAGAGGGGGGTTnTATAGGGGTCTATGGGGGGGGTTATnGGGGGGCTATAGGAGG
GGGGTTATGGGGTCCTATGGGGTCTCCCTCCAGGTACTCCACnCGGTCAGCTGCGCCCCCACTGGGCTTCCAGCAGCAACCCTCGTCTnGCGCnTATGGGGCAGAGGGGGGGACG
TGGGGGGCGTTATGGGGGGCTATAGGGGnTTTATGGGGGTTATGGGGTCTATGGTGGGGTTATGGGGTCTATGGGGTCTCCTCCAGGTACTCCACCTAGTCGGAGCTGCGCCCCG
CTGGGCCTTTCAGCAGCACCTCGnTCTGCGCTTATGGGGCGGAGGGAGGGGGGACATGGGGGGGTTATGGGGGGCTATGGGGGGGGGTTATGGGGTCTATGGGGGGCTATATAG
AAGGAATCCTATAGGGGnAACGAGAAGCCCTATTAAGCGAAATAGAAAAACCCTATAGGAGAACGAAAAATTCTACAGGGGGAATGAGAAAAACCCCATAAGAGAACGCAAGAAACC
CTATAGGGGAACGAGAAACCCTACAGGGCAATGGGGAAACCCTACnGGGAATGGAAACCCTATAGGGGAATGAGAAATCCTATAGGGGAATGAGAAACCCTACAGGAAAATGGGG
AACCCTACAGGAGAATGGGGGAACCCTACAGGAGATATGGGGAACnCCTACAGGGGGAATGAGGGAACCCTACAGGGGAACGAGAAACCCTACAGGGGAACTTTGAGAGAAACCC
TACAGTGGCAATGATAAGCCCTACAAGAGAATAAGAGAAACCCTACCAGGGCAATGGGAAACCCTACnCAGGGCAATGGGAnAACCCTACAGGGCAATnGAGAAGCCCTACAAGAGA
ATGGGGAAAGCCCCTACAGGGGAATnGAGAAACCCTACAGGGGAATGGGGAAACCCTACAGGGGAATGGGAAACCCTTATAGGAGAATGGGAAAnTCTCTACAGTGGAATGGGGAA
ACCCTACAGGGGCAATGGGAAACCCTnTACAAGGAAATGAGAGAACCCTACAAGGAAAATGAGAAACCCTTGGACAGGGGAATGGGAAACCCCTATAGGAGAATGGGAAACTCTCTA
CAGTGGGAATGAGGAAACCCTACAGGGCAATGGGAAACCCTACAAGGAAATGAGAAACCCTACCAGGAGAAGGCTTGGGGAAAACCCCTACAGGAGAATGGGAAnCCCTnnACAGG
GGAAATGGAAAGAAACTCTTACAGGGGAACAAGAGAAACCCTACAGGGGAGAATGGGGAACCCTACAGGGGAAGCGCGAGAAACCCCATAAGGCAACAAAAGAGATTCTACGTTGA
TAAACAGAGATCCCTAATAGGGGATCAGCCnGACCCTATAAGAACCTGGAACGACCCCACAGAGGAGTATGGGACCCTATAAAGGCGCCCTATGAGGGTCGCCTCACnCTGGGGAA
GGTGCGGCCCCGCAGCAGCTCCCGCACGCCAACTGAGCGCCTGCnCGGTCCCTAGCATGGGGGCGCCCCACATCCCGCGTCCCCCCGCTTATAGCGCCTTGnGnGGGGCGGCCC
GGCGCTCGCTTCA

>SRX14125033_2
CACGTAGTGCTCGAGATCGTCGCGGGGGCCGnGAAGGTGGGGGGGGGGCAGCGTCCATCCCCAGCTCCCTCTTTGCGGGCCACCAGGATGGGGACACTGGAAGCTCTTCCTCCT
CCAGGTACTCCACCGTCAGCTGCGCCCCCACTGGGCTTCAGCAGGCACCCTCGTCTGGGCGCTAGTGGGGCAGAGGGGGGGGACGTGGGGGGGTTATAnGGGGGTGTTATGGG
GTCTATnGAAGGTCTACATGGGGGCTATGAGTGGGGTTATTGGGGGGTAnTGGGGGGTATGGGGGCTCCTCCAGGTACTCCACCGTCAGCTGCGCCCGCCGGGCTTCCAGCAGGC
ACCTCGTCTGGGCGCTATGGGGCAGAGTGGGGGACGTGGGGGGGGTTATATGGTAGGGTCTCATGATGGGGGAGTTATAGGGGGGGCTTTnGGGGGGGTTATGGGGCCTATGGGG
TCTATATGGGGTCTCCTCCAGGTACTCCACCCGTCAGGGCTGCGCCCCACTGGGCTTCAGCAGCACCCTCGTCTGCGCCTATGGGGCGGAGGGGGGGGACGTGGGGGGCnGTTAT
GGGGTCTATGTGGGGGTTATAGGGGGCTTTTGGGGGGTTATGGGGnTCTAGTGGTGGTCTATGGGTAGGGCTCCTTCCAGGTACTCCACCGTCAGCTGCGCCCCCACTGGGCTTC
AGCAGCACTCCTCnGTCTGCGCTATGGGGCAGAGGGGGGGACGACGGGGGGGTTATGGGGGGnTGTTATGnGGGGTCTATGTGGGGGGCTATGAGGGGGTTATTGGGGGGTATGG
GGGGGTATGGGGGCTCCTCCAGGTACTCCACCGCTCGGGAGCTGCGCCCCCGCTGGGCTTCAGCAGCACCTCGTCTGCGCTTATGGGGCAGAGTGGGGGACGTGGGGGGCGTT
ATGGGGGGTGTTATGGGGGGGGTTATAGGGGCTTTGGGGGGGTTATGGGGTCTATGAAGGTCTCATGGGGGCTnATGAGGGGGTTATGGGGnGGTATGGGGAGGTATGGGGGGTTA
TGGGGGGGTTATGGGGTCTATGGGGGGAGTTATGGGGTTGGGATGGGGGGGTATGGGGGTCTCACTCCAGGTACTCCACCCGTCAGCTGCGCCCCGCTGGGCTTCCAGCATTAC
CTCGTCTGGCGCTTATGGGGCAGAGnGGGGGACATAGGGGGGGGTTATGGGGGGCTATGGGGGGTTATGGGGGCTTTGGGGGTTGGTATGGGGTCTATGGGGGCTCCTTCCAGGT
ACTCCACCGTCAGCTGCGCCCCACTGGGCTTCAGCAGCACCTCGTCTGGCGCTTATGGGGCnGGAGGGGGGGGGACGTGGGGGGGCATTATGGGGTCTATGGGGGGGGGGTTATA
GGGGGCTTTGGGGGGTTATGGGGTCTATGGGTGTGGTCTAGTTGGGGGCTCCTCCAGGTACTCCACCGTCAGCTGCGCCCCACTGGGCTTCAGCAGCACCTCGTCTGCGnCTATC
AnGGGGCAGAGGGAnGnGGGGACATGGGGGGGTTATGGGGGTCCATGGGGGGGCTATGGAGAGGGGGTTGATGGGGCTCTATGGGGGGTTATGGGGTCTAGTGAAGGTCTATGG
GGGGCTATGAGTGGGTTATGGGGTCTATGGGGGGCTATGGGGGGCTATTGGGGGGTATGGGGGGnCTCCTCCAGGTACTCCCACCGnnnTCAGCTGCGGCCCCGCTGCGGCTTCA
GCAGCACCCTCGTCTGCGnCTATnGGGGCAGAGGGGGGGACATAGAGnGnGGGGGTTATGGGGGTCCATGGGGGGCTATGGGGGGGCTATGnGGGGGGGTTAnGGGGGTnnTTTGG
GGGCTATATGGGAGAGAAACCGTGATAGGGGAAATCGAGAAACnCCTACAGGGGAACGAGAAACCCTAnnGGGGAACGAGAAACCCCACGGGGGAATGTGAGAAAACCCTACACAG
GGGAATGTGGGAAACCCTACAGGGGGAACGAGAAACCCTATAGCAGGGCAATGAGAAGCTCCTACAGGGGAACGAGAAACCCTACAGGGCAATGAGAAGCCCTACAGAGGGAACG
AGGAAAACCCTACAGGAGCAAATGTAGTGAGAAGCCCTACAAGAGAATGTAGnAGAAACCCTACAGGGGAATGGGGAAACCCTACAGGGGAACTAGnAAACCCTATGAGGAGAATA
GGGAAACTCTACAGTGGAATGGAAAACCCTACAGGGCAAATGGGGAAAACCCTACAGGGCAAATGGGAAACCCTACAAAGGAAATGAGAAAACCCTACAGGAGAATGGGGAACTCC
TACAGGGGAATGGGGAACCCTACAGGGGAACGAGAAAACCCTACAGGGGGAACGAGAAACCCTACAGGGGAACGGGAAACCCTACAGGGGAACGGCGAGGAAACCCTACAGGGG
AACGGnAGAAACCCTACAGGGGAACGGGAAACTCCTACAGGGGAACGGGAAACCCTACAGGGGAACGGGAAACCCCGTACAGGGGAACnCGAGAAACCCTACAGGGAGAATGGGA
AAACCCTAAGCAGGAGAACGGGAAACCCTACAGGGGAATGGGAAACCCTACAGGAGAATGAGAAAAnnCCCTACAGGGGGAATCGGAGAAAACCCTACAGGGGGAATGGAAAACCC
TACAGGGGAACAGGGAAACCCTATAGGAGAAATGGGAAACTCTTACAGTGAAATGGGAAACCCTAACAGCGAAATGAGAAAAAACTCCTGCAGGGGAATGGATACCCTATAGAGG
AAACGATAAACCCCATAAGACAACAAAGAGATTTACGGGGAAACAAAAGAGnCCCCATAGGGGATCAGCCAGACCCTATAAGAACCTGAGAACGACCCCACAGAGGAGTATGGGAAT
CCTTATAAGCGCCCCTATAGGGTTCGCCTCTACCTGGGGAAGGTGCGCGGGCCCCGCAGCTCCCGCACGAACTGAGCGCCTGnnCCGGTCCTTGTATGGGGCGCCCCCACATnCC
GCGCGTCCCCGCAGCGCCTTTGGGGGCGTGCCCGGGGCGCTCGCTTCA
>SRX14125034_1
CACGTAGTGCCCTCGACGATCGCGTGGGGCCGAAGGTGGGGGGAGGGCAGGnCGTCCATCCCCAGCCCCTCTTTGCCGGGCCACCAGGATGGGGnACACTGGAAGCTCTTCTCC
TCCCCAGGTACTnCCACCGTCAGCCTGCGCCCCCGCTGGnGCTTCAGCAGCACCCTGCCGTCTCGGCGCTTATGGGGCGAGAGGGGGGGnnGnCGGGGGGGTTnATGGGGGTCCA
TGGGGGGACTATGAGGGGAGGCTATGGGAGGGGGTTATGGGGGCTTTTGGGGGGGGTTATGGTAGGGTCTAAnTGGGGGCTTCCTCCAGGTACTCCAnCCGTCAGCCTGCGnCCC
CCCGCTGGGCTnTCAGCAGCACCTCCGnTCTGCGCTTATGGGGCAGAGGGGGGGGGACGTGGGGGGGTnTATAGGGTCTATGTGAGGGGnGGGTTATAGGGGGGCTTTGGGGGnT
GTTATGGGGTCTATGnnnGTGGTCTATGGGGGnnCTCCTCCCAGGTCACTCCACCGTCnnAGCTGCGCCCCGCTGGGCTTCTCAGCAGCACCTCGCGnTCTGCGCCTATGGGGGCAG
AGGGGGGACnTGTGGGGGGGTTATAGGGGnTCTATGGGGGGGGGGGGTTATAGGGGGGGCTTTGGGAAGGGAGTTATGGGGnnTCTGATGGTGGTCTATGGGGGGCTCCTCCCA
GGTACTCCAACnGTCAAAGGCTGCGCCCCCCTGTGGGCTTCAGCAGCAGCACnCCTCGGTCTGCGCTATGnGGGCGGAGAGGGGGGGACGCnTGGGGGGGTTAnGTTGGGGGTGT
TATGGGGGTCTATGAAGAGAGAAGGTCTATGAGGGGGCTATGTGAAGGGGGTTATTGGGGGGAGTATGGGGGAnCTCnCTCCAnAGTACTCCCACnCGTCnnAGCTCGCGCCCCGC
GTGGGCTTGCAGCCAGCACCTTCGTCTGCAGCTnTATTGGGGCGGAGGGGTGGGGGCCATGGGAGGGGGTTATGGGGTCCCTnATGGGGAGnGGnnTTATAGGGGTCCTATGAAA
AGGTCTATGnGGGGGCTATAGAGGGGAGTTATAGGGGGGTCTATGGGGGnGGGGGTTATnGGGGGGAGCTATAGGGGnGGTTATGGGGnTCTATGGGGTCCTCCTCCAGGTACTC
CACnGnnGTCAGCTGCGCCCCACTGGGCTTCAGCTAGCAATCCTGAAGTCTCACGTCTGCGGCTATnTGGGGCAGAGGGGGGGGGGGACGTGGGGGGGCGTTATnATGAGGGGGGC
TATAGGGGGGGTTATGnGGGGGGGGGTTATGGnGGTCTAGTnAGGTGGGGTTAnTGAGGGGTCTATAGAnGGGGTCTCCCTCTCCAGAGTACTCnnCACnCGTnCCAGCTCGCGCCC
GCCGCTGGGCTTCCTTTAnGCAGCAnCCTCnnGTCTGGCGCTTATGGGGAGGCGGAAGGGGGGGGACATGGnGGGGGTTATATnnGGGGGGCTATGGGGGGGGGTTACTGTTnGG
GGTCTATGGGGGGGGCTAGTATAGAAAnGGAATCCTATAGGGGGAAnCGAGAnAGCCCTATAAGCGAAATGAAAAAAACCCTATAGAGGAGAAACGCGAAAAAATTCTnACAGGGGA
ATGAGAAACCCCCATAAGAGAACGCAGAAAnCCTnCTATGAGGGGAACGAGAGAAACCCTACAGGGGCAATGTGGGAAAACCCTACGGGAAAATGGGGAAACCCTATAAGGGGAAT
GGAGAAATCCTGGATAGGGGGGAATAGAGAAAACCCCCTACAGGAAAATGGGGGAACCCTACAGGGAGAATGGGGGAACCCTACAGGGAGAATGGGGGAACCnCTAnACAGGGGA
ATGGnGGTAGAACCCnTACAGGGGAACGAGAAAACCCTACAGGGGGAACGAGnAAACCCTACTGAGGGCAAATGTGATAAGCCCCTACAAGAGAAATTTCTGAGAAAACCCTACATG
GGCAATnTGGGAAACCCTACAGGGCAAATGGGAAACCCTACAGnGGCAGCATGAGAAGCCCTAnCAGAAGAGAATGGGGAAACCCTACAGGGGAATAGAGGGAAACCCTACAGGG
GAATGGGGAAACCCTACAnGGGGAATGGGAAACCCTATAGGAGAATGGGAAACTCTCnnACAGTGGAATGGGAAACCTCCTGCTTACAGAGGCAATGGGAAAnCCCTACAAAGGAAA
TGAGAGAAACCCTACAAGGAAATnGAGAAACCCTACCnAGGGGAATGGGnAAACCCTATAGGAGAATGGGGAAACTCTAAGCAGTGGAATnTGGGAAACTCCTACAGGGCnAATnGG
GAAACCCTACAGAGGAAATnnGAGAAACCCTACAGGAGAGATGGGGAAACCCTACAGGAGGGAAATGGGGnAAACCCTACAGGGGAATGGGAAAACTCTCACAGGGGAACAAGAAA
CCCTACAGGAGAATAGAGGGGGAACCCTAGCAGGGGAACGAGAGAGAAACCCCACTTAAGGCAACACAAAGAGATCTACGGGATAAACAGAGnnATCCCTATAGGGAnTCAGCCTG
GACCCTATAnAGAACTCTGGAACGnnnACCCCACAnGAnGGAGTATGGGGGACCCTATATAAGCGCCCCGTATAGGnnnnnnGTCGnCCTCACCTnGGGGAAGGTGCGGCCCCnAGCA
GCTCCCCGCACGAACTGGAGCGCTTCGCCGGGTCCTTnnTGGGGCGCCCCCACATCCnGCGTCCCCCCGCCAGCGCCTTTAGGGGGCGnGCCCGGGCGCTTCCGCTTCA
>SRX14125034_2
CACGTAGTGCTCnCGACGGnTCGCCGGGGGCCGAGnnAGGTGnGGGGGGGGCAAnGCTGTCATCCCCCAGCCCCnCTCTTTGCGGnGCCACCAGGATGGGGCACACTGGAAGCTC
TTCTCCTTCCAGGTACTCCACCGTCGTCAGCTGCGCCCCCACTATGGGCTTCAGCAGCACCTnCGTCTGCGCCGCTATGGGGCAnAGAGGGGGGGGACGTGGGGGGGGTTATGGG
GGGGTGTTATnGGGAGTCTATGGAAAGGTCTATnGGGGGGGCTGATGAGGGGGTTATTGGGGGGAGTATGGGGAAGGnnTATGGGGGCTCCTCCAGGTACTCCACAnCGTCnnAGC
TGCGCCCCGCTGGGnnCTTCAGCAGCACCCTCGTCTGCGnCTATGGGGCAGAGGGGGGGGACGTGGGGGGGTAnTATAGGGTCTATGGGGGGGGGGTTATAGGGGGCTTTnGGG
GGGGTTATGGGnGCCTATGGGGnTCTATGGGGTCTCCCTCCAGGTACTCCACTCCGTCAGnGCTGCTGGCCCCACTGGGCTTCAGCAGCACCTCGTCCTGCGCTTATnGGGGGCGG
AGGGGGGGACGTGGGGGnGGCGTTATGGGGGTCTATGGGGGGGGGGGTTATAGGGGGGCCTTTGGGGGGGGGTTATAGnGGGTCTATGGTGGGTCTATGGGGGCTCCCTCCAGG
TACTCCACCGTCTAGCTGCGCCCCCACTGGGCTTnCAGGGCAGCACCTnnnCGTCTnGCGCTTATGGGGCAGAGGGGGGGGACGTGGGGGnnGGTTATGGGGGGGTGTTAnTnGGG
GGTCTATGGGGGGGCTATGAGGGGGGGTTATTGnGGGGnGTATGTAAGGGGGGGGTATGGGGGAGCTCCTCCCAGGTACTCCCACCGGGTCAGGGCTGCGCCGCCCCGCTGGnG
CTTCAGCAGCACCnCTCGTCTGCGCTTATGGGGCAGAGGGGGGGGGGGACGTnTGGGGGCGTTATAnGGGGGGTGTTATGGGGGGGTTATAGGGGGGCTTTnGGGAGGGGTTATnG
GGGTCTATGAAAGAAGGTCTATTTGTAGAGGGGGGGCTATGGnGAGGGGGTTATGGGGGGGTATGGGGGAGAGTATGGGGGGGGTTATGGGGGGGGTTnATGGGGTCTATGGGGG
GAGTTATGGAGGGGTTGATGGGGGGGGTATGGGGTCTTCCTCCAGGTACTnCCACCGGTCAGGCTGCGCnCCCGCGCTGGGCTTTCAGCATTACCCTCGTCTGGCGCTAATGGGG
CAGAGGGGGGGGACATAGGGGGGGGTTATGnGGGGGGGGAGCTATGGGGGGGGTTATGGGGGGAGCTTTGGGGGGAGGTTATACTGGGGTCTATGGGGGCTCCCTCCAGGTAC
TCCACCGTCnnAGCTGCGCCCCCACTGGGCTTCAGCAGCACCTGCGTCTCTGCGCTnATGGGGCGGGGGGGGGGAACGTTGGGGGGCATTATnnGGGGTCTATGGGGGGGGGGTT
ATAGGGGGGGGGCTTTGGGGGAGGGTTATGGGGTCTATGGTGGTCTATnGGGGGCTCCTCCAGGTAnCTCCACCGTCnGGAGCTGCGCCCCCACTGTGGGCTTCAGCAGCACTCC
TCGTCTCGCGCTTATGGGGCAGAGGGGGTGTGGGACATGGGGGGnGTTATnGGGGGTCCATGGGGGGGGCTGGATGGAGGGGGGGTTATGGGnGTCTATGGGGGGGGGTTATACC
GGGGTCTATGAAGAGTCTATGnGGGGGCTATGnGAGTGGGGTTATGTGGGGTCTATGAGnGGGGGGCTATAGGGGGGGGCTATTGGGGGnGTATGGGGGnnCTCCTTCCAGGTACCT
CCACCGGTCnnAGCTGCGCCCCGCTGGCTGCTTCAGCAGCACCTCGTCTGCGCAAATATGGGGCAGAGGAGGGGGGACATGGGGGGGGTTATGGGGGGGTCCATGnnnnnnTGGG
GGAGGGCTATGGGGGGCTATATGGGGGGGTTATGGGGGGTCTATGTGGGGGCTATATnTGGGAGnAAACCGTAnATGGGGGGTGAGAACGAGAAAACCCTACAGGGGGAACGAGG
GAAACCCTATAGGGGGAACGAGAAACCCTACGGGGGAATGAGAAACCCCTACAGGGGGAATGnGGAAACCCTAAGCAGGGGGAACGAGAAACCCTACnAGGGCAATGAGAAGCCC
CTACAAGGGGAACGAGnAAACCCTACAGnGGGCAATGAGAAGCCCTACAAGGGGAACGAGGAGAAACCCTACAGGAGCAATGAnGAAAGCCCTACAAGAGAATGTGAGAAACCnCA
TACAGGGGGAATGGAAAACCCTACAGGGGGAACTAGAAACCCTATAGGGAGAATGGGAATCACTCTACAGTGGGAATGGGGAAAACCCTCTCTACAGAGGGCAATGGGAAACCCT
ACAGAGGGCAATGTGGAAACCCTACAAGGAAATGAGnAAACCCTACAGGGAGAAnTGnGGGGAACCCTACAGGGGGAATnGGGGAACCCTACAGGGGGGAACGAGAGAGAAACCC
TACAGGGGAACAAAAGAGAAACCCTACAGGGGCAACGGGAAAACCCCTACAGGGGAAnCGAGAAAACCCTACAGGGGGGAACGAGAAAnCCCTACAGGGGAACGGGAAGGAAACC
CTACAGGGGAACGGGGAAACCCTAAGCAGGGGAACAGGGGAAACCCTACAnGGGGAACGAGAGAAACCCTACAGGGAGAATGGAAAnCCCnTACAGGAGAACGGGAAACTCCTA
CAGGGGAATGnnGGAAACTCCTACAnAGGGGAAGAATGAGAAACCCTnACAGGGGAATCCAAGAAACCCTACAGGGGAATTGGGGAAACCCTACAGGGGAACGGGGAAACCCTATA
GGAGAATGGGAAACTCTACACAGTGAAAATGGGAAACCCCTACAGCnGTAAATGnAGAAAAnCCCCTGCAGGGGGGAATGGGATnAACCCTATAGAGGAnACGTGATAAAGCCCCAT
AAGACAACAAAGAGATTTTACGGGGAAACAAAAAGACCCCATAGGGGGATCAGCCnCGACCCCTATAAGAACCTGnAACGACTCCCACAGAGGGGAGTAGTGGGACCCTATAAnnGC
GCCCTATAGGGTCGnCCTCACCTGnGGGAAGGTGCGGCGGCCCCCGCAGCTCCCGCAACnnGAACTGAGCnnGCTGCCACGGTnCCTTATGGGGGCGCCCCCACATCCCGCGnCT
CCCCAnCGCAGCGGCnnCCTTTGGGGGCGGCCCGGGCGCCCGCTTCA

>SRX19311940
CACGTAGTGCTCGACGTCGCCGGGGCCGAAGGTGGGGGCAGTCACCCCAGCCCCTCTTTATAGGCCACCAGGATGGGGACACTGGAGCTCCTCTTCTCCAGGTACCCATCTnnGC
TGCGCCCCACTGGCTTTAGCAGCACCTCGTCTGCGnnTATGGGGCAGAGGGGGACGTGGGGGGTnnGGGGGTGTTGTGGGGTCTATAGAAGGTCTATGGGGGCTAAGTGAGGGGn
TGTnGAGTATGGGGGTATGGGGGCTCCTCCAGGTACTCCACnGTCAGCTAnnCCTGGGCTACnnnGCAGCACCTCGTCCGCGCTTATGGGGCAGAGGGnnnnGGGGGTnATAAGGTC
TATGGGAnGnTATAGGGGCTTTAGAGGnTATGTAGGGCCTATGGGGTCTATGGGGGTCTCCTCCAGGTACTCCACACCGTCAGCTGCGCCCACTGGGCTTCAGCAGCACCTCGTCT
GGCGCCATGGGGCTnnnGnGGnCGGGnnGnAAGCAGGGGCTCGTGGGGGnGTTATAGGGnnTTTGGGGGGGTTATGGGGTCTGCAGTTATACAAGATATAGTCAGTCTnTGGGGGCC
CCTCCAnGTACCCACCGTCGCTGCAnnTGCGCCCACCGGGCnTCAGCAGCACCCTCGTCTnGCCCATGGGGCAGAGGGGnnTGGGGGGTATGGAGGTGTTATGAGGTGTnTGGGGA
CTATGAGGCTATTGGGGGnnTGGGnnnGCATGGGGCTCCTCAGTACACCACGTCCACTGGGCTCnnnTGGCGCCAGCAGCACCTCGTCTnnCATATGGGGCAGAGGGGGACGTGGG
GATAnAAGTGGTnnnTAGGGGGACAAGGGGGGCTTTGGGGGTACAAGGTnnATGGAAGGTCTGTGGGGCTATGAGGGGCAGTGGGGGGGCATGGAGGTATGGGGGGTTATGGGGG
GGGTTATAGGCTCnATGGGGAGTTAnGGGGTTGATGGGGGCATnGGGGTCTCCTCCAGGCACTCCATCGCCAGCCGCGCCCGCCTGGCCTCCAGCATCACTGCCTGCGCTATGG
GGCAGnGGAGGACATAGGGGnnGCTATGGGGCCATAAAGGTGnnGGGGCTTTAGGAAnnnnnnnnCTATAGCTCCTCCAGGCACnCCACTGCnGCnGCGCCCnATTGCCTGGAAACA
GCAGCACCTCGCCTTCTGCGCGTATGGGGGCGGAAGGGGGACGGGGGCATTATGGGGTCTGTTGGGGTATATAGGnnnCAGAAGTTATGGGTCTCATGGAGGTCTATGGGGCTCC
TCCAGGTACTCACCTGGnCAGCCGCTGCCCACTGGGCTTCAGTAGTACTCGTCTGCnnTTATGGGGCAAGAGGnGGGATATGGGGTGAAAAnGGGTCCATGGGGnnnGGTGTAGGG
GTTATGGGGnGTCTATGGGGGnnATGGGGTCTATGAnGCTGTCTATnGGGGCTGnTGGTGGGTTATGGGGTCTATGGGGGCTGTGGGnnGCTATTGTGGGGTATGGGGTTCCCCCAT
TCACTCCACAGTCAGCTGCGCCTGCTGGCnGCAGCAGCATTCCTCATCCGCGCnTATGGGGCAGAGGGGACATGGGGGTAnnGGGGTCCATGGGGGCTAAGGAGGCAGAAGAGGT
TGTGAGGGCCnTATGGGGCTnTnGAGAAACCAGGTAGGGGAGCAGAACCCTnnAGGGAACGAGAAACCCTATAGGGGAACTGAGAAAACCCnACGGGGAATGAGAAACCnTACAGG
GAATGGGAAACCCTACAGGGAACGAAACCCTACAGGGCAATGAGAAGCCCTnnnnGGnGAGAGAGAACCTACAGGGCAAATGTGAGGCCCTACAGGGGAACGAGAACCCnACAGGG
CAATGAGAGAAGCCCTACAAGAGAATGAGAAACCCTACAGGGAATGGGAAACCCCACAGGAACTATAGAAACCTAnnGGAGGCTCTAATGGGAAACnCTACAGGGnAATGGAAAACC
TACAGGnnATGGnAAnCCnGGACAATGGGAACCCTACAAGGAAATGAGAACCCATAGGAGAATGGGAACCCCACAGAAAnAGGGGAACTCTACAGGAACGAGAAACCCTAAGGGAAC
GGAGCCCTCACAGGGGAACGTAAAACCCCACAGGGGAACGAGAAnCCTACAGGGGAACGTGAAACCCCTACAGGGAnACnGGAACCCTACAGGGAACGGAACCCCTACAGGGGAA
CAAGAAACCCTACGAGGAGAATGGGAACCCnnCTAGAAGAACGAAACCCTACAGGGGAATGGGAGAACCCACAGGAGAATGAGAAAnCCTCAGGGGAATCAGAACCCCCACAGGGG
AATGGGAAGCTACGGGGCAnnGGAAACCTATAGGAGAATGGGAAACTCTACAGTGAAnnGGGAACCCTACAGGTAAATGAGAAACCGCAnnGGAATGGATATTCTATAGAGGAACGAA
AAACCCCCATAAGACAACAAAGAGATTTACGGGGAAACAAAAAAGACCCCATAGGGGGATCAGCCGACCCTATAAGAACCTGAACTTnACCCCACAGAGGAGTATGGATTTCnnnTGC
nnCnCCAGGGTCACTnnACTGGGGAAGGTGCGCCCCTGGCAGCTCCCGTGATCGAACCGAGCGCTGCCGGTCGCCTnTGGGGCGCCCAnACCCGTCCCCGCGAnGCCTTTGGGGC
GTGCCCGGCGCCGCTTCA
>SRX19311957
CACGTAGTGCCTGGGACGCCCATGGGGCCAAGGTGGGGGCAAAGGTCATCCCCCAGCCCTTTGCGGCCACCAGGATGGGGACACTGGAAGCTCTTCTCCTCCAGGTACTCCATCA
CCGGGCCATGCCTGGCCAGTCAGCAGCACCTCGTCTGCGCTTATGGGGCAGAGGGGGGGGACGGGGGGTTATGGGGTCCATGGGGCCATGGAGGCTATGGGGGTCTATGGGGC
TTATTGGGGCATGGGGTATGGGGCTCCCTCCAGGTACCCACTGGGGCCGCGCCCGCTGGGCTTCAGCAGCACCGTCTGCGCCATGGGGCAGAGGGGACGTGGGGGCTTTGGGG
TCCATGGGGGCTATGGAGGGGGTTATGGGGCTATGGGGGGGCTTATTAAAGGCGGGGGGTAAGTGGGGCCTCCACTCCATCACAGCTGCGCCTGGCTGGCTTCAGCAGCACTGC
CTGCCTATGGGGCTGAGGGGGGACGTGGGGGGTTATGGGGGGTGTTAAGGGGTCTATGAAGGTCTATGGGGGCTGTGTGGGGCTATTAAAAAAGTGGGGGCTCATGGGGCTCCT
CCAGTACTCCACTGTTGGCTGTGCCCCGCTGGGCTTCAGTAGCACCTCCTCGTCTGCGCTTAAGGGGCAGAGGGGGGGACGGGGGGCGAGTGGGGTGCAGGGGGTTATAGGG
GCTTTGGGGCAGGGGGTCTATGGTGGTCTATGGGGCTCCTCCATACCATCAGCTGCGCCCGCTGGGGCTCAGCACTGGGTCCGCGCTATGGGGGCAGAGGGGGGACGGGGACTA
GAGGGGTTCATGGGGGCCATGGGGGGCAGGGGTATGGGGAGGCATGGTACAGGTCTGGGTCTCCACCATCGTCAGCTGCGCTCATTGGGCTTCAGCAGCACCCTGCCTGGGCGC
TATGGGGGGATGTGGGGGGGACGTGGGGGGGGCTATGGGGGGGCTATGAGGCCATGGGGGTCTATGGGGGTTATGGGGTCTATGGGGAGCTAAATAGGAGAAACCCCATAGGAA
TCCAGAAACCCCATAGCTAAATGAGAAACCCTATAGGGAATGAGAACCTACAGGGCAACAGAAAACCCTGGCAAGGAAATGAGAAACCCTACAGTAGAATGGGAAACCCTACAGGG
GAACGGAAACCCTACAGGGGAACGGGAAACTTACAGGAACGGGAACCCTACAGGGAACGTGGAACCCTAGGGAAACGAAACCCTACAGGGGAACGAGAAACCCTACAGGGCAATG
AGAAGCCCTACAGAGAATGGGAAACCCTACAGGAATGGGAAACCCACAGGGGAACGAGAAACCCCAGGGAATGGGAAACCCTACAGGGAATGGGAAACCCTACAGGGAAATGGGA
AACCCTATAGAGAATGGAACTCTACAGTGGAATGGGAACCCTACAGGGCAATGGGAAACTTACAAGGAAATGAGAAACCCTACAGGAGAATGGGGAATTACAGGAGAATGGGAAAC
CCTACAGGGGAATGGAAACCCTACAGGGAACAAGAACTTATAGAGAATGGGGAACCCTACAGGAACGAGAAACCCCTATGGGGCGCAACAAAGAGACTTACGACAAACAGAGATCC
TCTTATAGATCAGCCGACTTATAAGAACCTGAACGTAATTCTCGAGAGTATGAGACTATAAGCGCCCATAGGGTCGCTCACTGGGAAGGTAAAGCGCCCGCAGCTCCGCACGAACTG
AGCGCTGCCGGCCCAGTGGGCGCCCACATCCGGGCTGGGCCCTCTAGCGCTTGGGGCGGCCCGCGCCGCTTCA
>SRX19311958_1
CACGTGAGTGCCCTCGACGTCATGGGGCCGAAGGTGAGGGTAAGGGCAACATCCCCAGCCCCTCTTTGCGTGGCCACCAGGATGGGAGACACTGGAAGCTCTTCTCCTCCAGGTA
CTCCGTCAGCTGCACCCCACTGGGCTTCAGCAGCACCTCGTCTGCACTATGGGGGCAGAGGGGAGACGTGGGGGGTTATGAAGTGTTATGGGATCTATGAAGGTCTATGTAGAGA
GCTATGAAGGGGGTTGTGGGGGTATGGGGGGTATGGGGGCTCCTCCAGGTACTCCACCGTCAGCTGCGCCGCTGAGCCTTCCAGCAGCACCTGCGTCTGCGCTATGGGGCAGAG
GGGACATTAAGGGGATTATAGGGTCTATAGGGGGGGCTATGGGGGGGGTTATGGGGGGGTATGGGGAGGTATGGTGGGGTTATGGGGGTCTATGGGGTCTCCACTCCCAGGTAC
TCCCACCGTCAGCTACGCCCCCCACCTGGGCTCTCAGCAGCACCTCATCTGGCGCTATGGGGCAGAGGGAGACGTGTAAGAGGTTGGATGGGAGTGTTATGGAGGGAGTTGATAG
GGAGCTTTAGGGAGGTTATGGGGTCTATGAAGGTCACATAGGGGGCTATAGGTGGGTTATGGGGGGGTATGGAGAACCAGGGGGGTTATGGGGGGGGTTATGGGGTCTATGGGG
GAGTTGCTGGGGTTGATGGGGGGTATGGGGGTCTCCTCAGTACTCCGTCAACTGCGCCGCGCTGGGCTTCAGCATTACCTCGTCTGCACTATGGGGCCGGAGGGGGCACGTAGAG
AGGTTATGGGGGGGTGTTATGGGGGTCTATATGGGGGGCTATGAGAAGTTATTGGGGGTATGGGGGGAGTATGGGAGACTCCTCCAGGTACTCCACGTCCTTCACTTGCCCCGCT
GGTACTTCAACAGCACCTCGTCTGCCATATGGGGGCGGAGGGGGATGGGGGGTTATGAGGGGTCTATGGAGAGCTATGGGGGGGGTGTAGGGTCTATGGGGGGGATTAGGTGGT
CTATAGAGGGGTTTCCTGCAGGCACTCCACCATCAGCTGCGCCCGCTGGGCTTCAGCAGCACCTCGTCTGCGCTATGGGGGCGGAGGGACGTGGGGGTTGTATGAGAGAGCTATG
GGGGGCGTTTATGGGGTCTATGGGAGCTATATAGAAAGAATCCTATAGAGGAACCGAGAAGGCCTATGGCAGAAATGAAAAACCTATAAGAAGAATCCGAAAATTCTGGCCAGGGGA
ATGAGAGAAGCCTATAAGAGAGACAGAAACCTATAGGGGGAAATCGAGAAACCCTATAGAGGGAACGAGAAACCCCTACAGGGCAATGGGGAAACCCTGCAGGGAATGGGAAGCC
CTATGGGGAATGAGAAATCCTGTATGGGGGAATGGGAGAAGCCCTACAGGGAAAGTGGGAGCCCTACAGGAGAGTAAGAGGGAACCCTACAGGGGAATGAGGAGCCCTACAGGG
GAACAGAGAGAGAGCCCTGCAGGGGGAACAGAAACCTATAGGGGAGAGAGAGCCCTACAGGGCAATGATAAGCCCTACAAGAGAATGAGAAACCCTACAGGGCAGTGGGGAAGCCCT
ACACAGGGCAATGGGGAAACCCTACAGACACTGACCAAACCACTACAAGAGAATGGGAAACCCTATAGGAAATGGGGAAACTCTACAGTGGAATGGGGAAACCCTACAGGGGCAAT
GGGAAACCCTACAAAGGAAATGAGAAACCCTACAGGGAGAATGGGGAACCCTACAGGAGAATGGGAACCCTGCAGGAAGTGGAAAACCCTACAGGGGAATGGAAAACCCTACAGG
GGAATGGAAACCCTACCCAGGGGAACAAAGAAACCCTATAGGAGAATGAGGGAACCCTACAGAGATGAAAGAGAAACCCCATAAGGCAACAAAGAGATCTGCGGATAAACAGGGGT
CCTATGAGGGATCAGCCGACCTATAAGAACCTGAATGACCCCGCGGAGGTATGGGACCCTATAAGCGCTCTATAGGGTCGCCCTTACCTGGGGAAGGTGCGGCCCGCAGCCTCCC
GCACGCAACTGAGCGCTGCCCGGTCCATATGGGGCGCCCTGCTGATCTCGGCTCCATCGCGTCCCCAGGCTGCTCTTGCGCCTTTGGGGnnnn
>SRX19311958_2
CACGTnGTGCCCCCCTCGGCGTCGCGGGGGGGCCGnAAGGTGnGGGGGCAGCGTCATCnCCTAGCCCnTTTTGCGGGCCACCAGGATGGGGACACTGAAGCTCTTCTCCTCCAGG
TACTCCACCGTCAGCGCTGCnnCCCCGCTGGTGTTCAGCAGCACCTGCGTCTGCGCTAGTGGGGCGGAGGGGGCGGGGGGAGTTATAGGGGGGATTATnGGGTCTATGGGGGGG
GCTATGAAnGGGnnnnGGGGGCTATAGGnCCTATGGGGGGCTCCTCCAGGTACTCCCACGGTCAGCTGCGCCCnGCTGGTGnnnCAGCAGCACCTCCGTGTCTGCGCTATGGGGCG
GAGGGGGGTTnCnnnGGGGGGTTATGAGGTCACCATGGnGGACTATGGAGGGCTATGGAAAGGGTTATGnnGGAGGCTATGAAGGGTTATGAGCGGTGTCTGTGGGGTCTnCTCCA
AGGTACTCCACCGTCAGCTGCnnCTACGCTGGTGTTnCAGCACCTGCGTCTCGCGCTATGGGGGCGGAGGGGGAGGCGTGGGGGGTTATGTGAGGGGATTATAAGGTCTATGGGG
GGGGCTGGATGGAAAGGGGTTATGTGGAGGCTTTGAAAAGGGGTTATGGGGnTCTGTGGGTGTCTCCTGCACTGAGGTCTCCACCGTCAGCTnnGCCCGCTnGnTACCAGCAGCAC
CTGCGTCTGCGCTATGGGGGCGnnGAGGGGGACGTGGGGGGATTACACAGTGGGGGTCTATGAGGGnnTGAGGGGGGCTTATGnnGGGTnTnGGGnGCTTATGAGAGTCTnTGTnn
GTCTCCTGnTAGTACTCCnCACCGGCCCAGCTGCCCGCTGGnCTTCAGCAGCACCTnCGTCTGCGCTATGGGGCGGAGGGGGGACATAGAGGAGGTTATGGGGTCTATGGGGGnT
GCTATAGGGGGCTTTGGGGGnTTATAnAGGGACTATAGGAGTCTATGGGGGAATTCTCCAGGTACTACACCAGTCAGCTGCGCCCCCACTGGGCTTCAGCAGCACCTGnnnATTCTG
GCTCnATGGGGGGGGGGGGGGGACGAAAAAAAATAGnAGGGGTTATGGGnGGnTATAnGGGTCTATGAGGGGCTATGGGnGnnnTTnnGnGTCTATAGGGTCTATnnGGGTCTGGCG
GAGCTCCTCCCAGGTACTCCACCGTCAGCTGTGCCnnTnnnGACCTTCCAGCAGCACCCAAATCTCTnnATnTGGGGCAGGAGGGGGGGGCGTGAGGAGGCGTTATGAGGGGGCTAT
GGAGAGACTATGTCGGGATCTATGGGGGGATTATGnGGnGACTATAGGGGTTTATGGGGnnATGAGGGTTAnTGGGGGTATGAGGGGGTATnGGGGGCTCCTCCAGGTACTCCACA
CCGTCAGCTGCnCCGCTGGGCTTCAGCAGCACnTTnCGTCTGCGCTATGGGGCAGAGGGGGGGGATnGGGGGGGATTATAGGGTCTnTGGGGGnGCTATGGGGGGGTTTTGGGGGT
ATGAGGAGGnTnTGGTGGGGTTAnTGGGGTCTATGGnGTCTCCTCAGnGTACTCCACCCGTCAGCTCGCCCCACTGGGCTTCAGCAGCACCTCATCTGCGCTATGGGGCAGAGGGG
GACGACGTGGGGGGTTATGGGGnGGTGCTATGGGGGGGGTnCCTTTATAGnGGCTTTGGAGGTTnTAGGGnTCTATGAAAGGTCTATGGGGGGCTCCTnCCAAGGTACTCCACCGT
CTAGCTGCGCGCTGCTGTGnTTCAGCAGCACCTnCGTCTnCGCTATGGGGCnGAGAGGGGACATGGGGGGTTATGGGGGTCCATGTAGAGGGGGCTATGGAGGGGTTATGGGGTC
TATGGGGGAGCTATAGGnGGGTCTATGnGnGCTCCTCCnGGTACTCCACCGTCAGCTGCGnCCCGCTGGCCTTnnGCAAGCACCTGCGTCTGCGCGCTATGGGGCAGAGAGGGGGG
ACGTGGGGGCGTTATGGGGGGGTGTTATGGGAGGTnnTATAGAGGGCTTTGGGGGnnnTnCATATTTGGGGTCTATGTGAAGAAAGGTCTAnnGGGGGCTACTGAGGGnTTATGCCAn
GGGAGGTATGAGGAnAGTATGGGGAGTTATCAGAGAGGGTTACTCGGGGGTTATGAnGGTCTATGAAGGGAGTTATGGGGTTGATGGGGGGTAnnTGGGGTCTCCTCCAGGTGACT
CCCACCACGTCAGCTGTGCCCCGCTGGACTTCCCAGCATTACCTGGACGTCTGCGCTATGGGGCAGAGGGGGGGACATAAGGGGGGTTATGnGGGGGCTATGGGGGTnGTTATAG
GGGACTTTGGGGGGTTATCGGGCnTATAGGGTCTATGTGGGGCTCCTCCAGGTACTCnCCGTCAGCTGTGCCCCTTGAGCCAGCAGCACCTnnCGTCTGCGCTATGGGGGCAGAGn
GGGGCGnGAGGCGTTATGTGGGGGCTATGGAGGACTATCGAAAGGATCTATACTGGGGGTTTAAGGCGGCTATGTGGGGTTTATGnGGGGTnCTATGGGAGGGGGTTATGGGGGC
TTTGGGGGTTATGCTGGGGTCTATGnGGGCTCCTCCCAAGAAGTGCTCCCACCGTCAGCTGCnTTGTnTGTTTCAGCAGCACCTCGTCTGCGCTATGGGGCGGnAGGGGGGGCGTG
GGGGGTTATGGGGGGTCATAGGGGAGGGnTTATGCTGGGTCTGTGAnGGTCTATGGGGGAGCTATAGAGGGGTTATGGGGGGTCTATGGGGGTTTATGGGGGGCTATAGGGGAC
CCTTATGGGGCTCTGTGGGGAGTCTCTCCTCCnAGGTACTCCCnCCGTCAGCTGCTGnCCTCTGGnnTCCAGCAGCACCTGCGTCTGCGCTATGnAGAGnGGGGGGGACATGGGGG
GTTATGnGGGGGGTTATGCTGAGGGCAGGCTATGAGGGGCTATGnGAGGTCTATnGAGAGGGGGTTATGTGGGTCTATGGGGAGCTAAATAGGAGAGAAACCCCATAGGGGAGCA
GAAACCCTAGnTGGCGAAATGAGAAACCCTATAAGAGACTACAAGAAATCCTACAGGAAAATAAGAAATCCTATAAAGAGAACAAGAAACCCTATGAGGAGCCAAAAACTCTGATGGG
GAATGAGAAACCTACAGAAAATGGGGAAACCCTACAGGAAAATGAGGAAACCCTACCTAGGGAATGAAAACCCTACAGGAGAATGGGGAACCCACTACAGGAAAATGnGGGGAACT
CCCTACAGnGGAATGAGAAGCCCTACAGAGGAAATCCAGTnGAGAAACCCACAGGGGnCAATGAAGGAAGCCnTACAGGAGnAATGGGAAAACCCTACAGGnCACAATGGGGAAACC
CTATGGGAGAATGGGAAGCCTTGCAGGGGAGCGAGAAACCCTACAGGGGGAAACGAGAAnnAGCCCTACAGGGAGAATGGGGAAACCCTACAGGGGGGAATGGGAAACCCTACAG
CCAATGnGGAAnCCTnCAGnGGAACnAGAAACCCCATAAGGCAACAAAGAGATCTACnGGATAAACAGAGAGnCCTATAnGGGATCAGCCCAAGGCCCTATAAAGAACCTGAGCGACC
CCnnAnCnTTGAAnCGATAnCAGGACCCTATAAAGCGCCCCTATAGGTGnnnGCCTCACTCACCTGGGGAAGGTGCTGGGCCCCGCAGCTCCCGCAnCGAACTGGnCGCTGCCGGTC
CTTATGGGGGCGCCCCACATCCAAGCGTCCCCTGCAGCGCCTTTGGGGGGnGGCnnCGnCGCCGCTTCA

>SRX19311959_1
AGTGCTCGACGTCGGGGCCGAAGTGGGGTAGCTGGCTATCCCCAGCCCCTCTTTGCGGGCCACCAGGATGGGGACATCAAGCCTTTCTCTCCTCCAGGTACTCCACTGTCAGCTG
CGCCCCGCTGGGCTTCAGCAGCACCCCGTCTGCGCCATGGGGCAGAGGGGATGGGGGGTGCTATGGCCCATGCCCGCCCCTGGCTCAGCAGCACCGTCTGCGCCAAGTGGGC
AGAGGGGCTGGTGGGGGGCTATGGGGTCTGGGGCAGGGGGGGCCAGTGGGGCCATGGGGTCCAGTGGGGTCCTCCAGGCACCACTGTCAGCCGCGCTGGCCCAGCAGCACC
CCGTCCGCGCCATGGAGGGGACATGGGGCCAAGGGGCCAAGTGAGGCCATTGGGGCATGGGGGCAAGGGGTCCAGGCACCCAATCATCAAATATCCCACTGCCTCAGCAACCTC
ATGTATGAGGGGACGGGGCCAAGTGCCAGGGGTCCATGAATATCCATGGGGCCATGAGGCCATTGGGGGCAGGGGCACAGTCCTCCAGGCACCAATCGTCAGCCCCGCTGGGCT
CAGCAGCAACCGTCCGCGCCCATGAGTGCACGGGGCCATAGGGTCCATGGGGCCAGGCTCTTGGGGGCAGGCCCAATGGGGCTCCTCCAGGTACCCACTGTCAGCCGCGCCCCC
ACTGGGCTTCCAGCAGCACCTCGTCTGCGCCATGGGGCGGAGGGGGACGGGGGTTATGGGGTCCATGGGGCAGGGGCTTTGGGGGGCTAGGGGCCCTGCAGGTCTCATGGGGC
CTCCAGGTACTCCACCGTCAGCTGCGCCCCACTGCCCAGAAGCAGTTAACCGTCCGCGCGCCCATGGGGCAGAGGGACCGGGGGCTATGGGGGTGCTATGGGGGTCCAAATGAG
GCATGGGGGCATGGGGTCCAGGCATTCCACCGCGCCTCCTGGGCCCAGCAGCCAACCGTCCGCGCCATGGGGCCAAGAGGGACGGGCACAACAGGGGGCCAGGGTCCATGGGG
CCATGAGGGCCATGGGCATGGGGAGGCATGGGGCCATGGGGCCATGGGGTCCATGGGGAGCTATGGGGTTGATGGGGCATAACTCCAGGTACTCCACTGCCCGCCGCGCCCCG
CTGGGCCTGAGACACCTCGTCTGTTATGGGCAGAGGGGGGATGGGGCCAGGGGCCATGGGGGCTATGGGGGGCCTGGGGGGCCAGGTCCATGGGGCCCAGGCACCCACCGTCA
GCTGCCTCCCACCGGGCTCCAGCAGCAACCGTCCGCGCCATGGAGGGGACGGAAGCATCATAGTCCTGGGGGCCATAGGGGCCCTTGGGGCTATGTGTCCATGGTGGTCCATGG
GGGTCTCCCAGGCACCACCAAATGCGTCCCCAACTGGGCCCAGCAGCAACCCCCGTCTGCGCCCAAGGAGGGACAATGGGGGCCAGGGTCCATGGGGCCAAGTGAGGCCAAGTG
TCCAAGGGGCTCATGAATATCCATGGGGCCAAGGAGGCCAGGGTCCAGAAATGGGGCCATTGGGGTTCCTCCATACTCCACTGTCACGCGCCCGCTGGGCTCAGCACCGTCTGCG
CCTATGGGGCAGGAGTGAAGGCTGGGGCTGGGGGTCCATAAAATAAGTGGGGCCAAGTAAAATAAGGGTCTATGGGGCCATATGAGAGAAACCGTATAACCGAGAAACCCCACAG
GAACGAGAAACCCTATAGGGGAACGAGAAACTCACGGGGAACGAGAACCCCACGGGAACGAGAAACCCTACGGGGAACCCTACAGGGGAACGAGAAACCCTACAGGGCAATGAGA
GTCTCAGGAACGAGAAACCCTACAGGCAATGAGAAGCCCTACAGGAAATCTGAGAAACCCTACAGGGCAAATGAGAAGCCCCACAAGAGAATGAAACCCCAATAGAATGGGAAACC
CCACAGGAACTAGAAACCCTATAGGAGAAACTTCTACAGTGGAATGGAAACCCTACAGGGCAATGGGAAACCCTACAGGGCAATGGGAAACCCGTATAAGGAAAATGAGAAACCCCA
CAGGAGAATGGGGAACCTACAGGGAATGGGGAACCCACAGGGGAACGAGAAACCCTACAGGGGAACGGGAAACCCACAGGGGAACGGGAAACCCTACAGGGAACGTGAGAAACC
CTACAGGAAATCCAGAAACCCTACAGGGGAACGGGAAACCCTACAGGGAACGGAAACCCTACAGAGGGAACGGGAAACCCTACAGGGAACGAGAACCCCACAGGAGAATGAAACC
CCACAGGAGAACGGGAACCCTACAGGAATGGAAACCCTACAGGAGAATGAGAACCTACAGGGAATCAGAAACCTTTACAGGGAATGGGAAACCCAGGGGAACGGGAAACCTATAG
GAGAATGGGAACTCTACAGTGAAATGGAACCTTACAGCGAAATGAGAAACCCTAGGAATGGGATACCTATAGAGGAACGAACGATAAACTTACAAGACAACAAAGAGATTTACGGGG
AAACAAAGGGGCAAGACCCCATAGGGATCACGACCCTATAAGAACCTGAAACGACCCCACAGAGGAGTATGGGACCCATAAGTGCCCTATAGGGTCGCCCCTCACCTGGAAGGTG
TGGCCCCGCAGCCCTGCACTAACTGAGCGCTGTCCTTATGGGGCGCCCACATCCATGCCCCCGCAGCGCCTTTGGGGGCGGGCCCATGCCGCT
>SRX19311959_2
TCGACGTCTGCGGGGGCCGGAGGTGGGGGGCGCCTATATCCCCAGCCCCTCTTTGCGGGCCACCAGGATGGGGACACTGAAGCCTCTTCTCCTCCAGGTACTCCACCGTGTCAGC
TCAAGCCCCGCTGGCTGCCAGCAGCACCTCGTCTGCCTTTATGGGGCAGAGGGGGACGTGGGGGCGTTAGCAGGTCTGTAAAGGGGTGCCATACTGGGGTCTGGATAGAGGAGTT
ATGGGGTTGATATAGAGAGGTATAGAGTCTCCTCCGGGTACTCACTGCATCAGCTCTTGGCTGTGCTTCAGCGGCACCTGAGTCTGCGCTATGGGGCGGAGGGGACATGGGGGGT
TATGGGGGGTGTGAAGTCTATAGAGAATGTTATGGGGGGTTATGTGAGGCTATAAGTCTATATAGGGTCTCTTCCAGGTACTCACACACGTCAGCTAAGCCCCGCTGGGCGCTTCCA
GCAGCACCTCCGTCTACACTATGGGGGCGGAGGGGGACATAAGGGGTGTGAGGTCTATGAGGGGAGCTATGGGGGTGTGGGGGAGTAATAGAGGTGAGGCTCCTCCAGGTACTC
ACGCGTCAGCTGCGCGCCCACTGGGCTTCAGCAGCACCTCGTCTGCGCTATGGGGGCGGAGGGGACGTGGGGGTTATTATGAAGTGTTATAGGGGTCTATGAAGGTCTGTGGGGG
CTGTAAGCAGGGGGTTGTTAGGTATGGGGGTATGGGGAAAAGCTCCTCAGGTACTCCACAGTCAGCTGCGCCCCGCTGGCTTCAGCAGCACCTCGTCTGCAGTATGGGGCAGAGG
GGGACGTGGGGTGCTCAAGGGTCTGTCAGGAGGTTATAGGGAAGCTTTGGGGTTATGGGGCCTATGGGGTCCATGGGGTCTCACTCAGGTACTCCAACGTCAGCGCCTCGCTGGA
AGCCAGCAGCACCTCGCATCTGCGCTATGGGGCGGAGGGAGATGTGGGGAGGCGTTATAGGGGTCTATGGGAGTTACTAGGGCTTTTAGGGGTTATGCGGTCTATGGTGGTCTAT
GGGGGCTCCTCCAGGTACTCCACCGTCAGCTGCGCCTGGGCTTCAGCAGCACCTGCGTCTGCGCTATAGAGGGCAGAGGGCGAATGAAGTTATAGAAGTGTTATGGGGTCTACTG
GGGGCTATGAGGGTTGTTGGGTATGAAGGTATACGGGGCTCCTCCAGGTACTCCACCGTCAGCTGCGCCCGCTGGCGCCAGCCAGCACCTCGTCTGCGCTCTGGGGCCAGAGGG
GGCGATGAGGGAGCGTTATGAGGGGGTGTTATGAGGGTTATATAGGGGCTTTGGGGGTTATGGGGTCTATGAGAAGTCTATAGGGGGCTATGAGGGGTTATGGGGAAATTGTAGG
GGAGGCTATGGGGGGGTTATGGGGTTTATGGGGTCTATGGGGGAGTTATGGGGTTGGGATGGGGGGTATGAGGGTCTCCTCCGTTACTCCACCCGTCAGCTGCGCCCGCTGAGCT
TCGGCGTTACCTCGTCTGCGCTATGGGGCAGAGGGGGACATAAAGGGGGTATGGGGGGCTATGGGGGTATGGGGGAGCTTAAAGAAGTTATGGGGTCTGTAGAAACCACTCCGGG
TACTCCACCGTCAGCTGCGCCCCACCAGGCTTCAGCACCTCGTCTGCGCTTGTATGGGGCGGAGGGACGTGGGGGCATTATCAGGTCTATGGGGGTTTATAGGGGCTTTGGGGTT
ATGGGGTCTGTGGTGGTCTATGGGGGCTCCTCCAGGTACTCCACGTCAGCTGCGCCCCACTGGGCTTCAGCAGCACCTCGTCTGCGCTATGGGGCAGAGGGACATGGGGGGTTAT
GGGGGTCCATGGGGGCTATGGAGGTTATTAGGGGTCTATGGGGGTTGTAGGGTCTATGAAGGTCTATGAGGGGGCTATGGGTGGGTTACTTGGCTGGGGTCTATGTGGGGGGCTA
GCATTCAGCCTGGGGGTATGAGGGGCTCCTCCAGGTACTCCACCGTCAGCTGCGCCCCGCTGGTAGCAACACCGTCTTTGTATGGGGCCAGAGGGACATGGAGGTTGTGGGGGTC
CATGGGGGCTATGGGGGCTATGGGGGTGCATGGGGTCTTATGGGGGGCTATATGGGAGAAACACATTATAAGGAGCGAGAAACCCTACAGGGCACGAGAAACCCTGTAGGGAACG
AGAAACCCTGCGGAGGCGAGCCCTAGGGGCGAGAAACCTAGGGGCGAGAAACCCTACAGAGAACCGAGAAACCCTAAGGACGAGGCCAGGGCAATGAGAACGCTACGGGAACGA
GAGGCACACAGGGCAATAGGAAGCCCTACAGGGGAACGAGAAACCCTGCAGGGCAATGAAAACCTCCTACAGAGAATGAGAAACCCTACAGGGGAATGGGAAACCCCTACGGGGA
ACTAGAAACCCTATGGGAGAATAGGAAACTCTACGGTGGAATGGAAAACCCTACAGGGCAATGAGAAACCCTACACAGAACAGTAGGAAGCCACAAAGGAAATGAGAAACCCTACAG
GAGAATGGGGGAACCCCTACAGAATGGGGGAACCCTACAGAGACGAGAAACCCTACAGGGCCGGGAAACCCCTACAGGGGGCGGGAAACCCTACAGGGAGCGAGAAACCCTAAG
GCAAAGAAACCCAGAGAACGAGAAACCCTACAGGGAGCGGGAAACCCTACAGAGGACACGGAAGCCCTACGGGGGCGATACTACGGGGGCGAGAAACCCTACAGGAGGTGAGAG
AGCCTACAGGAGAGACGAGAGCCACTACAGAGATGGGAAACCCTACAGGAGAATGAGAAACCCTGCAGGAGTCAGAAACCCGGAGAATGGAAGCCTGAGGCAGGAAACCCTATA
GGAGAATGGGAAACTCTACAGTGAAATGGGAAACCCTACAGCGAAATGAGAAACCCGCAAGAGATGGGATACCCTATAGAGGAGCGATAAACCCCATAAGACAACAAAGATTTGCAG
GAAACAAAAGACCCCATAGGGGATCAGCCGACCCTATAAGAACTAACGACCCCACAGAGGTTATGGGACCTATAAAGCGCCTATGGTTAACTCACCTGGGAAAGGTCTGTTGCCAAC
TCCCATGCGAACTAGTAGCTGCCGGTCCTTGTAGGCGCCCATCGCGTCCCCGCGGCGCCATTTGAGGCGACCCAGCGCCGCTTCA
>SRX19311960
CACGTnAGTGnnnCTCnGACGTCGCGGGGGCnGAAGnnnnTCAGGGGGGGCAGCGTCATCCCCAGCCCCTCTTTGGCGGGCCACCAGGATGGGGTACACTGAAGnCTCTTCTCCTCC
AGGTACTCCACCCCGTCGAGCTGCGCCCCACTGGGCTTCAGCAGCACCCTCCGGTCTGCnGCTTATGGGGCAGnAGGGGGGGACGTGGnAGGnGGTTATGGGGGGTGTTATnGGG
GTCTATGTGAAGGTCTATGGGGGGGCTATGAGGGGGGTTATTGGGGGGTATGGGGGGGTATGTAGGGGCTCCTCCAGGTACTTCCACCGTnCAGCTGGGCAGCCCGCTGGnGCTT
CAGCAGCACCTCAACGTCTnGCGCTATTGGGGCAGAGGGGGGGGACGTGGGGGGATTATAAGCGCAGGGTCTATGGGGGCCATGGCTATGGGGGGGTTATGGGGGTATGnGGGAG
GTATGGnCTGGGGTTATGGGGTCTATGGGGTCTCCTCCAGGTACTCCACCGTCAGCTGCGCCnCCACTGGGCTTCAGTGCAGCACCTCCCATCTCATGCnGCTATGGGAGCAGAGG
GGGGACGTnGGGGGGGGTTATGGGGGGGTGTTATnnGGGGGGTTATAGGAGGGCTTTnGGGGGGTTATGGGGTCTAnATGAAGGTCTATnGGGGnGGGCTATGnGGTGAGGTnnTA
TnnGGGGGGTATGGGGGAGGTATnnGGGGGGGTTATGGGGGGnnnTnnnGGGGTCTATGGGGGAGTTATGGGAGGTTGATGGGGGGTATGGGGTCnnnTCCTCCAGGTACTCCCAC
CGTCnCAGCTGCGCCCGCTCnnGGCGTCAGCATTACCTGCGTCTGCGCTATGGGGGCGGAGGGGGCACGTGGGGGTATTnTGGGGGTGTTACTnGGGGGTCTATGGGGGGnCTAT
GAGGGGnGTTATTGGnnGGGTAnATGGGGGTATnGGGGGCTCCTCCAGGTACTCCACCnGTCAGCTGCGCCCCCGCTGGnnTCCAGCAGCACCTGCGTCTGCGCTATGnGGGGCnG
nGGGGGGGACGTGGGGGGTATATGGGGTCTATGnnGGGGGGCTATGGGGGGTnTTATnAGGGGGTCTATGGGGGGGTTATGGGGTCTATnnGGGGTTTCCTGCAGGCACTCCAC
CATCCCAGnnnCTGCGCCCnGCTGGGCTTCAGCAGCACCTnCCGTCTGCGCTTATGGGAGCGGCAGGGGGACATGGGGGGGTnnTATGTAAGGGGGGGCTATGGGGGGGGTTATA
TnGGGGTCTATGnGGGGGCTATATAGAAGGGAATCCTATAGGGGAAACAGAGAGAAGCCCTATAGCGGAAATGAAAAAACCCTATAGAAGAAATCCGAAAAATTCTACAGGGGGAAT
GAGAAACCCCTATAAGAGAAATCCCGAGAAACCCTATATATGGGGAACAAGAGAAACCCAnnnGGGGAACAGAGAAACnCCCnTACnAGGGCAATGGGAAACCCCTACGnGGGAATG
GGAAACCCTATAGGGGAATGGGAGAAATCCTATAGGGGAATGAGAAAGCCCTACAGnGAAAATGGGGAACCCCTACAGGAGAATGGGGAACCCTACAGGGGAATGnGGGGGACCC
CTCCAGGGAnGAGAAACCCTACAGGGGAAACGAGAAACnCCTACAGGGGGGAACGAGAGAAACCCTACCAnGGGGCAAATGTGATACAGCCCTACAAnAGAGAATGAGAAACCCTA
CAGGGCAATGnGAGGAAACCCTACACAGAGGCAATGTnGGAAACCCTACAGnGGCAATGAGAAGCCCCTACAGAGAGAGAATGGGAAAACCCTAnTAGGAGAnTATGGGAAAACTCT
CTACAGTGGAGATGGGAAAACCCTACAGGGCAATGGGAAACCCTACAAGGAAAnTGAGAAACCCTTACAGGAGGAATGGGGGAACCCTACAGGAGAGATAGGGGGGAACCCAGGn
CTACAGGGAAGATGGAAAACCCTACAGGGAAnnnnnGGGAAACCCTACAGGGGAATGGAGnAAAAACCCCCTACAGGGGGAAGCCGAGAAACCCTATATGGGAGAATnTGGGGAACC
CnTACAGGGGAACGGTAGAAACCCCAnTAAGGCAACTnAAAGAGAnTCTACGGATAAACAGAGATCCCTATnAGGGATCCAGCCGACCCCTATAAGAAACCTGGAATGACCCnnnnnGC
GGAGGAGTATGGGACCTATAAGCnGCCCTATAGGGGTCGCCTnACACCTGGGGAAAGGTGCGGCCCCGGCAGCTCCnCnCGCAnnGAACTGAAGCGCTTCTGnCCCGGTCCATATG
nGGGCGCTCCCACATCCGCGTCCCCTCCCGCAGCGCCTTTGGGGGCGnGCCCGGCGCCAGCTTCA
>SRX19311961
CACGTAGTGCTCGACGTCGCGGGGGCCGAAGAAGGGGGGGCAGCGTCATCCCCAGCCCTCTTnTGCGGGAGCCACCAGGATGGGGACACTGCTAAGCTTTCTTCTCCTCCCAGGT
ACTCCACCGTCAGCCGnnCnCCCACTGGGCTTCAGCAGCACCTTCCCGTCTGCGCTATGGGGGCAGAGGGGGGnCnnTGGGGGGGGTTATGGGGGTGTTATGGAGGGGTCTAT
GAAGAGGTCTATGGGnGGCTATGAGGGTTATTGGGGGTATGGGGGnAnGTATGGGGCTCCTCCAGGTACTTCCACCGTCAGCCGCGCCCGCTGGGCTTCAGCAGCACACCnCTGG
ACGTCTGCGCTAnTGGGGCAGAGAGGGGGACGTGGGGGATTATAGGGTCTATnnGGGGGCTATGGGGGGTTATGGGGGGTATGGGGAGGTATGGTGGGAGTTATGGGTCCTnTGG
GGTCTCCTCCAGGTACTCCCACCGAATCAGCTGCGCCCCACTGGGCTTCAGCAGCACCTCATCTAGCGCTATGGGGGGCCAGAGGGGGCnnGTGGGGGGTTATGGGGGGTGTTAT
GGGGGGGTTATGAGAGGGGCTTCGCTGGGGGnTTTACCTGGGGTCTGATGGTGGTCTATGGGGGCTCCTCCAGGTACTCCACCGTCAGCTGCGCTCnCACTGGGCTTCAGCAGCA
CCTCTCGTCTGGGCGCTATGGGGGGAGnGGAGGGACGATTGGGGGTATGGTGGGGGGATTATAGGGTCTATGGGGGGGCTATGGGGGGTTATGGGGGGCTATGGGGTCTGGCAT
GGGGGCTCCTCCAGGTACTCCACCGGTCAGCTGCGCCCCGCTGGGCTTCAGCAGCACACCCTCATCTGGGCGCTTATGGGGCGGAGGGGGTGACGTGGGGGGGGGATTATAGG
GTCTATGGGGGCTGTGGGGGGGTTATGGGGGGTCTATGGGGGGGGGTTATGGGGTCTATnGGGGTTTCCTGCAGGCACTCCAACCATCGAGGGCTGCATGCCCGTTGGGCnTCAG
CAGCACCTGCGTCTGCCTnnTATGGGGnCGGGAGGGGGGACATGnGGGGTTATGGGGGGGCTATnnGGGGGGGGTTATGGGGGTCTATGnGGGGCTATATAGAAGGAATCCTATAG
GGGAACGAGAAGCTCCTATAGCGAGAATGAAAACCCCCTATAAGnAGAACGAAAAATTTTCTACAGGGAATGAGAGnAAACCCTATAAGAGAACGAGAAAACCCTATAAGGGGGAAC
GAGAAACCCTACAGGGCAATATTGGGAAAnCnnCTTACGGGAATGGGAAACCCTATAGGGGnAATGAGAAATCTTATAGGGGAATGTGGAGAAACCCTACAGGAGAATGGGGGAACC
CCTACAGGGGGAATGGGAACCCTACAATGGGGAACnnGAGAAAACCCTACAGGGCAATGAGAAGCCCCTACAGGAGAATGGGAGAGAAGCCCACTACAGGAGAATGGGGAAACCC
TACAGGAGAAnnTGGGAAAACCnnTACAGGAGAATGGGAACCCTnACAGGGGAACAAGAAACCCTACAGGGGAAACGAAAAGCTCCTACAGGAGAATGTGGAAACCCTACAGGGAA
nTGGGGAAACCCTACAGnnGAAATGGGAAACCCTACAGGGGCCAATAGAGAGAACCCnTACAGGGCGAATGAGAGGAACCCTTTACAGGGCCCAATGGAAAAACCCTATACAGGGA
ATGGAnAAACTCTATACGGGGAATGGAAAACTCTATAGAGGAACGAGGAAACCCTTATAGGAGAATGGGAAAnCTCTACAGTGGAATGGGAAAACCCTACAGAACGAAATAGAGAAAC
CCTTAATACAGAGGGAATGGGATACTCCTATAGAAGGGAACGTGATAnAACCCCATAAGACAACAAAAAGAGAGATCTACGGnnGGGGAAACAAAAAAnCCCCCATAGGGATCAGCCA
GACCCTATAAGAACCTGGAACGACCCCAnCAGAGGAGTATGGGACCCTATAAGCGTCCCTATAGGGTCGCCTCACCTGGGGAAGGTGCGGnCCCGCAGCTCCCCGCACTnTAACnG
AGCGCTGCCGGTCTCTTnTGGGGCGCCCCCACATCCGCGTCCCCnCGCAGCGCCTTTGGGGGCGGCCCGGCGCCGCTTCA

Supplementary Figure 6. Sequence structure of individual alleles of chicken PHF8 intron 18. (A) Sequence dot plots are shown for blastn self-alignments of allele sequences comprising intron and adjacent exons. First chart represents Ggswu reference sequence. Allele sequences were reconstructed from Nanopore sequencing of chicken individuals. Allele sequences are shown in a colored schemes above each chart where each nucleotide is represented by a different color (A - green, C - blue, G - grey, T - red). (B) Reconstructed allele sequences in fasta format.

Paralogons	Spotted gar	Reedfish	Xenopus	Human	Spotted gar	Reedfish	Xenopus	Human	Spotted gar	Reedfish	Xenopus	Human	Spotted gar	Reedfish	Xenopus	Human
	A	A	A	A	B	B	B	B	C	C	C	C	D	D	D	D
1	96	85	84	94	73	67	67	70	70	69	57	70	96	96	88	96
2	23	22	23	22	6	15	8	16	11	10	2	10	21	20	22	23
3	75	70	67	75	34	42	33	44	71	71	69	73	46	39	32	44
4	20	28	25	30	35	31	34	33	37	34	33	37	6	13	13	11
5	66	64	65	68	47	43	39	45	35	40	34	39	60	56	55	59
6	48	47	39	44	39	39	25	36	45	46	43	44	4	14	18	19
7	69	66	62	64	57	53	48	52	43	45	40	47	70	59	62	67
8	33	30	33	32	30	27	23	27	35	35	34	35	26	23	21	23
9	26	22	25	26	1	6	7	10	27	25	24	28	22	20	17	22
10	5	12	11	19	50	49	44	47	45	42	34	39	51	46	45	50
11	60	59	52	60	43	40	38	38	48	48	30	46	63	54	60	62
12	32	32	29	32	26	25	23	27	25	24	24	19	34	34	33	34
13	16	14	15	14	23	22	23	23	6	6	8	11	25	20	16	24
14	94	89	86	88	72	69	57	68	61	58	48	58	93	87	82	91
15	53	52	49	56	42	42	40	38	41	38	37	31	55	51	50	56
16	21	18	11	18	43	40	38	42	40	34	40	41	44	42	40	42
17	14	14	8	14	18	18	19	19	21	20	17	22	12	11	10	14

Summary (average) for all four species				
Paralogons	A	B	C	D
1	89.8	69.3	66.5	94
2	22.5	11.3	8.25	21.5
3	71.8	38.3	71	40.3
4	25.8	33.3	35.3	10.8
5	65.8	43.5	37	57.5
6	44.5	34.8	44.5	13.8
7	65.3	52.5	43.8	64.5
8	32	26.8	34.8	23.3
9	24.8	6	26	20.3
10	11.8	47.5	40	48
11	57.8	39.8	43	59.8
12	31.3	25.3	23	33.8
13	14.8	22.8	7.75	21.3
14	89.3	66.5	56.3	88.3
15	52.5	40.5	36.8	53
16	17	40.8	38.8	42
17	12.5	18.5	20	11.8

Chromosomal location of paralogons				
Paralogons	A	B	C	D
1	7	33	27	2
2	19	38	5	15
3	3	37	5	32
4	1	14	18	31
5	1	26	29	12
6	1	26	5	31
7	Z	28	25	10
8	Z	28	8	30
9	17	16	8	30
10	34	11	20	2
11	3	23	20	2
12	3	23	4	1
13	36	24	25	1
14	4	13	22	6
15	4	13	4	1
16	35	9	4	1
17	35	9	21	1

Supplementary Figure 7. Biased fractionation of numbers of identified ohnologs suggest that chicken dot chromosomes come from the recessive subgenome of the second WGD karyotype. The table on the left shows the number of ohnologs found for each paralogon and quad in four non-archosaurian species. Data comes from the Supplementary table 1B published by (1). The second table averages the numbers through all four species. Because quad pairs A-B and C-D were generated to reflect the second WGD, one of the quads of A-B pair or C-D pair comes from the dominant genome while the other of the same pair from the recessive genome. Biased fractionation indicates that the quad containing higher number of ohnologs (on green background) comes from dominant while the quad with lower number of ohnologs (on reddish brown background) comes from the recessive subgenome. The table on the right shows the chromosomal location of each paralogon/quad in chicken. Dot chromosomes are indicated by red ink on a yellow background.

1. Lamb, Trevor D. 2021. “Analysis of Paralogons, Origin of the Vertebrate Karyotype, and Ancient Chromosomes Retained in Extant Species.” Genome Biology and Evolution 13 (4). https://doi.org/10.1093/gbe/evab044.
