# Supplementary material for: Decoding the Avian Missing Gene Mystery: Dot Chromosomes Unmask Extensive Gene Loss and Novel Genetic Instability
Source: Genome Biol Evol. 2026 Feb 17;18(3):evag038. doi: 10.1093/gbe/evag038 (PMC12954441; doi:10.1093/gbe/evag038)
Supplement: evag038_Supplementary_Data [file evag038_supplementary_data.zip › Supplementary_tables.pdf]

Detection of ohnologs pertaining to paralogons mapping to chicken dot chromosomes and to their isoparalogons located on other chromosomes.

Absence/presence of genes in a specific taxon is shown by - / + signs placed on color-coded background. Absence means that the gene was not found in any species in a specific taxon. Presence is indicated when at least one species in a taxon possesses the gene. Presence does not guarantee that the sequences of the gene are complete and the gene is functional.

Chromosome 19

Paralogon 2A	Homo sapiens	Amphibia	Lepidosauria	Testudines	Crocodylia	Aves	Palaeognathae	Neognathae	Gallus gallus	Notes
ALDOC	+	+	+	+	+	+	+	+	+	
ANKRD13B	+	+	+	+	+	+	+	+	+	
ATP2A3	+	+	+	+	+	+	+	+	+	
BCL7B	+	+	+	+	+	+	+	+	+	
CORO6	+	+	+	+	+	+	+	+	+	
DOC2B	+	+	+	+	+	+	+	+	+	
DTX2	+	+	+	+	+	+	+	+	+	
LHX1	+	+	+	+	+	+	+	+	+	
MYL10	+	+	+	+	+	+	+	+	+	
ORAI2	+	+	+	+	+	+	+	+	+	Misidentified as ORAI3 in bGalGal1.mat.broiler.GRCg7b genome annotations.
P2RX5	+	+	+	+	+	+	+	+	+	
RTN4RL1	+	+	+	+	+	+	+	+	+	
SEPTIN4	+	+	+	+	+	+	+	+	+	
SEZ6	+	+	+	+	+	+	+	+	+	
SH2B2	+	+	+	+	+	+	+	+	+	Misidentified as SH2B1 in bGalGal1.mat.broiler.GRCg7b genome annotations.
SMTNL2	+	+	+	+	+	+	+	+	+	
TAF15	+	+	+	+	+	+	+	+	+	
TAOK1	+	+	+	+	+	+	+	+	+	
TMEM132E	+	+	+	+	+	+	+	+	+	
TNFAIP1	+	+	+	+	+	+	+	+	+	
VPS37D	+	+	+	+	+	+	+	+	+	
YPEL2	+	+	+	+	+	+	+	+	+	
Number of genes	22	22	22	22	22	22	22	22	22	

Dot chromosome 38

Paralogon 2B	Homo sapiens	Amphibia	Lepidosauria	Testudines	Crocodylia	Aves	Palaeognathae	Neognathae	Gallus gallus
ALDOA	+	+	+	+	+	+	+	+	+
ATP2A1	+	+	+	+	+	+	+	+	+
BCL7C	+	+	+	+	-	-	-	-	-
CORO1A	+	+	+	+	-	-	-	-	-
DOC2A	+	+	+	+	-	-	-	-	-
FBXL19	+	+	+	+	+	+	+	+	-
FUS	+	+	+	+	+	+	+	+	+
KCTD13	+	+	+	+	+	-	-	-	-
MYL11 (MYLPF)	+	+	+	+	+	+	+	+	+
ORAI3	+	+	+	+	-	-	-	-	-
SEPTIN1	+	+	+	+	-	-	-	-	-
SEZ6L2	+	+	+	+	+	+	+	-	-
SH2B1	+	+	+	+	+	+	+	+	+
TAOK2	+	+	+	+	+	+	+	+	+
TGFB1I1	+	+	+	+	+	+	+	+	-
YPEL3	+	+	+	+	+	-	-	-	-
Number of genes	16	16	16	16	11	9	9	8	6

Paralogon 3A	Homo sapiens	Amphibia	Lepidosauria	Testudines	Crocodylia	Aves	Palaeognathae	Neognathae	Gallus gallus	Notes
ACTN2	+	+	+	+	+	+	+	+	+	
AKT3	+	+	+	+	+	+	+	+	+	
ATL2	+	+	+	+	+	+	+	+	+	
B3GNT2	+	+	+	+	+	+	+	+	+	
BCL11A	+	+	+	+	+	+	+	+	+	
CAPN2	+	+	+	+	+	+	+	+	+	Misidentified as CAPN1 in bGalGal1.mat.broiler.GRCg7b genome annotations.
CCDC85A	+	+	+	+	+	+	+	+	+	
CCDC88A	+	+	+	+	+	+	+	+	+	
CDC42BPA	+	+	+	+	+	+	+	+	+	
CHRM3	+	+	+	+	+	+	+	+	+	
CNIH3	+	+	+	+	+	+	+	+	+	
DEGS1	+	+	+	+	+	+	+	+	+	
EFEMP1	+	+	+	+	+	+	+	+	+	
EGLN1	+	+	+	+	+	+	+	+	+	
EHD3	+	+	+	+	+	+	+	+	+	
EML4	+	+	+	+	+	+	+	+	+	
ENAH	+	+	+	+	+	+	+	+	+	
EPAS1	+	+	+	+	+	+	+	+	+	
ESRRG	+	+	+	+	+	+	+	+	+	
FAM98A	+	+	+	+	+	+	+	+	+	

Region on chromosome 3

FERMT1	+	+	+	+	+	+	+	+	+	
FOSL2	+	+	+	+	+	+	+	+	+	
FOXA2	+	+	+	+	+	+	+	+	+	
GPR137B	+	+	+	+	+	+	+	+	+	
HHIPL2	+	+	+	+	+	+	+	+	+	
HNRNPU	+	+	+	+	+	+	+	+	+	
IRF2BP2	+	+	+	+	+	+	+	+	+	
ITPKB	+	+	+	+	+	+	+	+	+	Misidentified as IPTKC in bGalGal1.mat.broiler.GRCg7b genome annotations.
JAG1	+	+	+	+	+	+	+	+	+	
KCNK1	+	+	+	+	+	+	+	+	+	Misidentified as KCNK7 in bGalGal1.mat.broiler.GRCg7b genome annotations.
KCNK12	+	+	+	+	+	+	+	+	+	
KCNK2	+	+	+	+	+	+	+	+	+	
KLC4	+	+	+	+	+	+	+	+	+	Misidentified as KCL2 in bGalGal1.mat.broiler.GRCg7b genome annotations.
LTBP1	+	+	+	+	+	+	+	+	+	
MAP4K3	+	+	+	+	+	+	+	+	+	
MARK1	+	+	+	+	+	+	+	+	+	
MEIS1	+	+	+	+	+	+	+	+	+	
MERTK	+	+	+	+	+	+	+	+	+	
MTA3	+	+	+	+	+	+	+	+	+	
NFKBIE	+	+	+	+	+	+	+	+	+	
NRXN1	+	+	+	+	+	+	+	+	+	
OTX1	+	+	+	+	+	+	+	+	+	
PAK5	+	+	+	+	+	+	+	+	+	
PCNX2	+	+	+	+	+	+	+	+	+	
PELI1	+	+	+	+	+	+	+	+	+	Misidentified as PELI3 in bGalGal1.mat.broiler.GRCg7b genome annotations.
PLCB1	+	+	+	+	+	+	+	+	+	
PLD5	+	+	+	+	+	+	+	+	+	
PLEKHG1	+	+	+	+	+	+	+	+	+	
PPM1B	+	+	+	+	+	+	+	+	+	
PPP1R14C	+	+	+	+	+	+	+	+	+	
PPP2R5A	+	+	+	+	+	+	+	+	+	
PRKD3	+	+	+	+	+	+	+	+	+	
PROX1	+	+	+	+	+	+	+	+	+	
PRPH2	+	+	+	+	+	+	+	+	+	
PYGB	+	+	+	+	+	+	+	+	+	
RASGRP3	+	+	+	+	+	+	+	+	+	
RCOR3	+	+	+	+	+	+	+	+	+	
REL	+	+	+	+	+	+	+	+	+	
RHOU	+	+	+	+	+	+	+	+	+	
RIN2	+	+	+	+	+	+	+	+	+	
RPS6KC1	+	+	+	+	+	+	+	+	+	
RTN4	+	+	+	+	+	+	+	+	+	
SIPA1L2	+	+	+	+	+	+	+	+	+	
SLC24A3	+	+	+	+	+	+	+	+	+	
SLC8A1	+	+	+	+	+	+	+	+	+	
SNX5	+	+	+	+	+	+	+	+	+	
SPRED2	+	+	+	+	+	+	+	+	+	
SPTBN1	+	+	+	+	+	+	+	+	+	
STRN	+	+	+	+	+	+	+	+	+	
TGFB2	+	+	+	+	+	+	+	+	+	
TMEM151B	+	+	+	+	+	+	+	+	+	
TRERF1	+	+	+	+	+	+	+	+	+	
VSX1	+	+	+	+	+	+	+	+	+	
ZBTB18	+	+	+	+	+	+	+	+	+	
ZFP36L2	+	+	+	+	+	+	+	+	+	
Number of genes	75	75	75	75	75	75	75	75	75	

Paralogon 3B	Homo sapiens	Amphibia	Lepidosauria	Testudines	Crocodylia	Aves	Palaeognathae	Neognathae	Gallus gallus
ACTN3	+	+	+	+	+	+	+	-	-
ATL3	+	+	+	+	+	-	-	-	-
CAPN1	+	+	+	+	+	+	+	+	+
CCDC85B	+	+	+	+	-	-	-	-	-
CCDC88B	+	+	+	+	+	-	-	-	-
CDC42BPG	+	+	+	+	+	-	-	-	-
CHRM1	+	+	+	+	+	-	-	-	-
CNIH2	+	+	+	+	+	+	+	+	+
EFEMP2	+	+	+	+	+	+	+	+	+
EHD1	+	+	+	+	+	-	-	-	-
EML3	+	+	+	+	+	+	+	+	+
FERMT3	+	+	+	+	+	+	+	+	+
FOSL1	+	+	+	+	+	-	-	-	-

Dot chromosome 37

GPR137	+	+	+	+	-	-	-	-	-
HNRNPUL2	+	+	+	+	+	+	+	+	+
KCNK4	+	+	+	+	+	-	-	-	-
KCNK7	+	+	+	+	-	-	-	-	-
KLC2	+	+	+	+	-	-	-	-	-
LTBP3	+	+	+	+	+	+	+	+	+
MAP3K11	+	+	+	+	+	+	+	+	+
MAP4K2	+	+	+	+	+	+	+	-	-
MARK2	+	+	+	+	+	+	+	+	+
MTA2	+	+	+	+	+	+	+	+	+
NRXN2	+	+	+	+	+	+	+	+	+
PACS1	+	+	+	+	+	+	+	+	+
PCNX3	+	+	+	+	+	+	+	+	-
PELI3	+	+	+	+	-	-	-	-	-
PLCB3	+	+	+	+	+	+	+	+	+
PPP1R14B	+	+	+	+	+	+	+	+	+
PPP2R5B	+	+	+	+	+	-	-	-	-
PYGM	+	+	+	+	+	+	+	+	+
RASGRP2	+	+	+	+	+	+	+	+	-
RCOR2	+	+	+	+	+	+	+	+	+
RELA	+	+	+	+	+	+	+	+	+
RIN1	+	+	+	+	-	-	-	-	-
ROM1	+	+	-	-	-	-	-	-	-
RTN3	+	+	+	+	+	+	+	+	+
SIPA1	+	+	+	+	+	+	+	+	+
SNX15	+	+	+	+	+	+	+	-	-
SNX32	+	+	-	+	-	-	-	-	-
SPTBN2	+	+	+	+	+	+	+	+	+
TMEM151A	+	+	+	+	-	-	-	-	-
TTC9C	+	+	+	+	+	+	+	-	-
ZBTB3	+	+	+	+	+	+	+	+	+
Number of genes	44	44	42	43	35	27	27	23	21

Dot chromosome 29

Paralogon 5C	Homo sapiens	Amphibia	Lepidosauria	Testudines	Crocodylia	Aves	Palaeognathae	Neognathae	Gallus gallus	Notes
ARAF	+	+	+	+	-	-	-	-	-	
ARHGAP4	+	+	+	+	+	-	-	-	-	
ATP2B3	+	+	+	+	+	+	+	+	-	
AVPR2	+	+	+	+	-	+	+	+	+	
BGN	+	+	+	+	+	+	+	-	-	
CACNA1F	+	+	+	+	+	+	+	+	+	
CCDC120	+	+	+	+	+	+	+	+	+	
CDK16	+	+	+	+	+	+	+	+	+	
DUSP9	+	+	+	+	+	-	-	-	-	
ELK1	+	+	+	+	+	-	-	-	-	
FAM3A	+	+	+	+	+	+	+	+	+	
FGD1	+	+	+	+	-	-	-	-	-	
FLNA	+	+	+	+	+	+	+	+	+	
FOXP3	+	+	+	+	+	+	+	+	+	
GATA1	+	+	+	+	+	+	+	+	+	
GDI1	+	+	+	+	+	-	-	-	-	
GRM6	+	+	+	+	+	+	-	+	+	
IQSEC2	+	+	+	+	+	+	+	+	+	
IRAK1	+	+	+	+	+	+	+	+	-	
ITIH6	+	+	+	+	+	+	+	+	+	
KCND1	+	+	+	+	+	-	-	-	-	
KDM5C	+	+	+	+	+	+	+	+	+	
L1CAM	+	+	+	+	+	+	+	+	+	
MAGIX	+	+	+	+	-	+	-	+	-	
OPN1LW	+	+	+	+	+	+	+	+	+	
PDZD4	+	+	+	+	+	+	+	+	+	
PFKFB1	+	+	+	+	-	+	+	+	+	
PHF8	+	+	+	+	+	+	+	+	+	
PLXNA3	+	+	+	+	+	+	+	-	-	
PLXNB3	+	+	+	+	+	-	-	-	-	
PNCK	+	+	+	+	-	-	-	-	-	
PRICKLE3	+	+	+	+	+	+	+	+	+	
SLC38A5	+	+	+	+	+	-	-	-	-	
SRPK3	+	+	+	+	+	+	+	+	+	
SYN1	+	+	+	+	+	+	+	+	+	
SYP	+	+	+	+	+	+	+	+	+	

Chromosome 12

	Homo sapiens	Amphibia	Lepidosauria	Testudines	Crocodylia	Aves	Palaeognathae	Neognathae	Gallus gallus	
TFE3	+	+	+	+	+	+	+	+	+	
USP11	+	+	+	+	-	-	-	-	-	
WNK3	+	+	+	+	+	+	+	+	-	
Number of genes	39	39	39	39	32	28	26	26	22	

Paralogon 5D	Homo sapiens	Amphibia	Lepidosauria	Testudines	Crocodylia	Aves	Palaeognathae	Neognathae	Gallus gallus	
AMIGO3	+	+	+	+	+	+	+	+	+	
ARHGEF3	+	+	+	+	+	+	+	+	+	
ASPN	+	+	+	+	+	+	+	+	+	Missidentified as BGN in bGalGal1.mat.broiler.GRCg7b genome annotations.
ATP2B2	+	+	+	+	+	+	+	+	+	
ATXN7	+	+	+	+	+	+	+	+	+	
BRPF1	+	+	+	+	+	+	+	+	+	
BSN	+	+	+	+	+	+	+	+	+	
CACNA1D	+	+	+	+	+	+	+	+	+	
CAMK1	+	+	+	+	+	+	+	+	+	
CELSR3	+	+	+	+	+	+	+	+	+	
CHL1	+	+	+	+	+	+	+	+	+	
CNTN4	+	+	+	+	+	+	+	+	+	
CPNE9	+	+	+	+	+	+	+	+	+	
DUSP7	+	+	+	+	+	+	+	+	+	
FAM3D	+	+	+	+	+	+	+	+	+	
FGD3	+	+	+	+	+	+	+	+	+	
FLNB	+	+	+	+	+	+	+	+	+	
FOXP1	+	+	+	+	+	+	+	+	+	
FRMD4B	+	+	+	+	+	+	+	+	+	
GATA2	+	+	+	+	+	+	+	+	+	
GNAI2	+	+	+	+	+	+	+	+	+	
GNAT1	+	+	+	+	+	+	+	+	+	
GRM7	+	+	+	+	+	+	+	+	+	
IQSEC1	+	+	+	+	+	+	+	+	+	
IRAK2	+	+	+	+	+	+	+	+	+	
ITIH3	+	+	+	+	+	+	+	+	+	ITIH3 qualifies as 5D paralog rather than ITIH1.
ITPR1	+	+	+	+	+	+	+	+	+	
LHFPL4	+	+	+	+	+	+	+	+	+	
LMOD3	+	+	+	+	+	+	+	+	+	
LRIG1	+	+	+	+	+	+	+	+	+	
LRRN1	+	+	+	+	+	+	+	+	+	
MAGI1	+	+	+	+	+	+	+	+	+	
MITF	+	+	+	+	+	+	+	+	+	
MKRN2	+	+	+	+	+	+	+	+	+	
OMD	+	+	+	+	+	+	+	+	+	
OXTR	+	+	+	+	+	+	+	+	+	
PDZRN3	+	+	+	+	+	+	+	+	+	
PFKFB4	+	+	+	+	+	+	+	+	+	
PHF2	+	+	+	+	+	+	+	+	+	
PLXNA1	+	+	+	+	+	+	+	+	+	
PLXNB1	+	+	+	+	+	+	+	+	+	Missidentified as PLXNB3 in bGalGal1.mat.broiler.GRCg7b genome annotations.
PPARG	+	+	+	+	+	+	+	+	+	
PPM1M	+	+	+	+	+	+	+	+	+	
PRICKLE2	+	+	+	+	+	+	+	+	+	
RAF1	+	+	+	+	+	+	+	+	+	
RASSF1	+	+	+	+	+	+	+	+	+	
RHO	+	+	+	+	+	+	+	+	+	
RHOA	+	+	+	+	+	+	+	+	+	
SEMA3F	+	+	+	+	+	+	+	+	+	
SLC38A3	+	+	+	+	+	+	+	+	+	
SLC41A3	+	+	+	+	+	+	+	+	+	
SLMAP	+	+	+	+	+	+	+	+	+	
SRGAP3	+	+	+	+	+	+	+	+	+	
SYN2	+	+	+	+	+	+	+	+	+	
SYNPR	+	+	+	+	+	+	+	+	+	Missidentified as SYP in bGalGal1.mat.broiler.GRCg7b genome annotations.
TMCC1	+	+	+	+	+	+	+	+	+	
UCN2	+	-	-	+	+	+	+	+	+	
USP4	+	+	+	+	-	+	+	+	+	
WNK2	+	+	+	+	+	+	+	+	+	
Number of genes	59	58	58	59	58	59	59	59	59	

Paralogons 8C9C	Homo sapiens	Amphibia	Lepidosauria	Testudines	Crocodylia	Aves	Palaeognathae	Neognathae	Gallus gallus	Notes
ABCA4	+	+	+	+	+	+	+	+	+	
ANGPTL1	+	+	+	+	+	+	+	+	+	

Chromosome 8

ARHGAP29	+	+	+	+	+	+	+	+	+	
ATP6V1G3	+	+	+	+	+	+	+	+	+	
BCAR3	+	+	+	+	+	+	+	+	+	
BRDT	+	+	+	+	+	+	+	+	+	
CACNA1E	+	+	+	+	+	+	+	+	+	
CAMSAP2	+	+	+	+	+	+	+	+	+	
CNN3	+	+	+	+	+	+	+	+	+	
COL11A1	+	+	+	+	+	+	+	+	+	
COLGALT2	+	+	+	+	+	+	+	+	+	
DAB1	+	+	+	+	+	+	+	+	+	
DENND1B	+	+	+	+	+	+	+	+	+	
DIRAS3	+	+	+	+	+	+	+	+	-	
DNAJB4	+	+	+	+	+	+	+	+	+	
DNM3	+	+	-	+	+	+	+	+	+	
DOCK7	+	+	+	+	+	+	+	+	+	
ELAVL4	+	+	+	+	+	+	+	+	+	
FNBP1L	+	+	+	+	+	+	+	+	+	
FOXD2	+	+	+	+	+	+	+	+	+	
FUBP1	+	+	+	+	+	+	+	+	+	
GADD45A	+	+	+	+	+	+	+	+	+	
GIPC2	+	+	+	+	+	+	+	+	+	
HOOK1	+	+	+	+	+	+	+	+	+	
JAK1	+	+	+	+	+	+	+	+	+	
JUN	+	+	+	+	+	+	+	+	+	
KANK4	+	+	+	+	+	+	+	+	+	
KDM4A	+	+	+	+	+	+	+	+	+	
LHX4	+	+	+	+	+	+	+	+	+	
LRP8	+	+	+	+	+	+	+	+	+	
LRRC8C	+	+	+	+	+	+	+	+	+	
MAST2	+	+	+	+	+	+	+	+	+	
MOB3C	+	+	+	+	+	+	+	+	+	
MTF2	+	+	+	+	+	+	+	+	+	
NFIA	+	+	+	+	+	+	+	+	+	
NOTCH2	+	+	-	+	+	+	+	+	+	
OLFM3	+	+	+	+	+	+	+	+	+	
PALMD	+	+	+	+	+	+	+	+	+	
PBX1	+	+	+	+	+	+	+	+	+	
PDE4B	+	+	+	+	+	+	+	+	+	
PIK3R3	+	+	+	+	+	+	+	+	+	
PKN2	+	+	+	+	+	+	+	+	+	
PLPP3	+	+	+	+	+	+	+	+	+	
PLPPR5	+	+	+	+	+	+	+	+	+	
PRRC2C	+	+	+	+	+	+	+	+	+	
PTBP2	+	+	+	+	+	+	+	+	+	
PTGFR	+	+	+	+	+	+	+	+	+	
PTPRF	+	+	+	+	+	+	+	+	+	
RAB3B	+	+	+	+	+	+	+	+	+	
RASAL2	+	+	+	+	+	+	+	+	+	Missidentified as SYNGAP1 in bGalGal1.mat.broiler.GRCg7b genome annotations.
RGL1	+	+	+	+	+	+	+	+	+	
RPE65	+	+	+	+	+	+	+	+	+	
RXRG	+	+	+	+	+	+	+	+	+	
S1PR1	+	+	+	+	+	+	+	+	+	
SGIP1	+	+	+	+	+	+	+	+	+	
SLC25A24	+	+	+	+	+	+	+	+	+	
SLC44A5	+	+	+	+	+	+	+	+	+	
SSBP3	+	+	+	+	+	+	+	+	+	
STXBP3	+	+	+	+	+	+	+	+	+	
TTC39A	+	+	+	+	+	+	+	+	+	
VAV3	+	+	+	+	+	+	+	+	+	
ZSWIM5	+	+	+	+	+	+	+	+	+	
	62	62	60	62	62	62	62	62	61	

Paralogons 8D9D	Homo sapiens	Amphibia	Lepidosauria	Testudines	Crocodylia	Aves	Palaeognathae	Neognathae	Gallus gallus
ANGPTL6	+	+	+	+	-	-	-	-	-
AP1M2	+	+	+	+	+	+	+	+	+
BRD4	+	+	+	+	+	+	+	+	+
CACNA1A	+	+	+	+	+	+	+	+	+
CAMSAP3	+	+	+	+	+	+	+	+	+
CNN1	+	+	+	+	+	+	+	+	+
COL5A3	+	+	+	+	+	+	+	+	-
COLGALT1	+	+	+	+	+	+	+	+	+

Dot chromosome 30

DENND1C	+	+	+	+	+	-	-	-	-
DNAJB1	+	+	+	+	+	+	+	+	+
DNM2	+	+	+	+	+	+	+	+	+
DOCK6	+	+	+	+	+	-	-	-	-
ELAVL3	+	+	+	+	+	+	-	+	+
GIPC1	+	+	+	+	+	+	+	+	+
GMIP	+	+	+	+	+	+	+	+	+
HOOK2	+	+	+	+	+	+	+	+	+
JUNB	+	+	+	+	+	+	+	+	+
KANK2	+	+	+	+	+	+	+	+	-
KHSRP	+	+	+	+	+	+	+	+	+
LDLR	+	+	+	+	+	+	+	+	+
LRRC8E	+	+	+	+	+	-	-	-	-
MAST1	+	+	+	+	+	+	+	+	+
NFIX	+	+	+	+	+	+	+	+	+
NOTCH3	+	+	+	+	+	+	+	+	-
OLFM2	+	+	+	+	+	+	+	+	+
PALM3	+	+	+	+	+	-	-	-	-
PBX4	+	-	+	+	+	+	+	+	+
PDE4A	+	+	+	+	+	+	+	+	-
PKN1	+	+	+	+	+	-	-	-	-
PLPPR2	+	+	+	+	+	+	+	+	+
PTGER1	+	+	+	+	+	-	-	-	-
RAB3D	+	+	+	+	+	-	-	-	-
RAD23A	+	+	+	+	+	+	+	+	+
RASAL3	+	+	+	+	+	+	+	+	+
RFX1	+	+	+	+	+	+	+	+	+
RGL3	+	+	+	+	-	-	-	-	-
S1PR2	+	+	+	+	+	+	+	+	+
SH2D3A	+	+	+	+	+	-	-	-	-
SLC25A23	+	-	+	+	+	-	-	-	-
SLC44A2	+	+	+	+	+	+	+	+	+
STXBP2	+	+	+	+	+	+	+	+	-
TRIP10	+	+	+	+	+	-	-	-	-
TYK2	+	+	+	+	+	+	+	+	+
VAV1	+	+	+	+	+	+	+	+	+
ZSWIM4	+	+	+	+	-	-	-	-	-
	45	43	45	45	42	32	31	32	27

Chromosome 17

Paralogon 9A	Homo sapiens	Amphibia	Lepidosauria	Testudines	Crocodylia	Aves	Palaeognathae	Neognathae	Gallus gallus	Notes
ANGPTL2	+	+	+	+	+	+	+	+	+	
ATP6V1G1	+	+	+	+	+	+	+	+	+	
BRD3	+	+	+	+	+	+	+	+	+	
CACNA1B	+	+	+	+	+	+	+	+	+	
CAMSAP1	+	+	+	+	+	+	+	+	+	
CERCAM	+	+	+	+	+	+	+	+	+	
COL5A1	+	+	+	+	+	+	+	+	+	
DAB2IP	+	+	+	+	+	+	+	+	+	
DENND1A	+	+	+	+	+	+	+	+	+	
DNM1	+	+	+	+	+	+	+	+	+	
FNBP1	+	+	+	+	+	+	+	+	+	
FUBP3	+	+	+	+	+	+	+	+	+	
LHX3	+	+	+	+	+	+	+	+	+	
LRRC8A	+	+	+	+	+	+	+	+	+	
NOTCH1	+	+	+	+	+	+	+	+	+	
OLFM1	+	+	+	+	+	+	+	+	+	
PBX3	+	+	+	+	+	+	+	+	+	
PHF19	+	+	+	+	+	+	+	+	+	
PKN3	+	+	+	+	+	+	+	+	+	
PRRC2B	+	+	+	+	+	+	+	+	+	
RALGDS	+	+	+	+	+	+	+	+	+	Misidentified as RGL4 in bGalGal1.mat.broiler.GRCg7b genome annotations.
RXRA	+	+	+	+	+	+	+	+	+	
SH2D3C	+	+	+	+	+	+	+	+	+	
SLC25A25	+	+	+	+	+	+	+	+	+	
STXBP1	+	+	+	+	+	+	+	+	+	
VAV2	+	+	+	+	+	+	+	+	+	
	26	26	26	26	26	26	26	26	26	

16

Paralogon 9B	Homo sapiens	Amphibia	Lepidosauria	Testudines	Crocodylia	Aves	Palaeognathae	Neognathae	Gallus gallus
ATP6V1G2	+	+	+	+	+	-	-	-	-

Dot chromosome	BRD2	+	+	+	+	+	+	+	+	+
	COL11A2	+	+	+	+	+	+	+	+	+
	NOTCH4	+	+	+	+	-	-	-	-	-
	PHF1	+	+	+	+	+	+	+	+	+
	PRRC2A	+	+	+	+	+	+	+	+	+
	RGL2	+	+	+	+	+	+	+	+	+
	RXRB	+	+	+	+	+	+	+	+	+
	SLC44A4	+	+	+	+	+	-	-	-	-
	SYNGAP1	+	+	+	+	+	+	+	+	+
		10	10	10	10	9	7	7	7	7

	Paralogon 3C	Homo sapiens	Amphibia	Lepidosauria	Testudines	Crocodylia	Aves	Palaeognathae	Neognathae	Gallus gallus	Notes
Region on chromosome 5	ACTN1	+	+	+	+	+	+	+	+	+	
	AKT1	+	+	+	+	+	+	+	+	+	
	ATL1	+	+	+	+	+	+	+	+	+	
	BCL11B	+	+	+	+	+	+	+	+	+	
	CAPN3	+	+	+	+	+	+	+	+	+	
	CCDC85C	+	+	+	+	+	+	+	+	+	
	CCDC88C	+	+	+	+	+	+	+	+	+	
	CDC42BPB	+	+	+	+	+	+	+	+	+	
	CHRM5	+	+	+	+	+	+	+	+	+	
	CNIH1	+	+	+	+	+	+	+	+	+	
	DEGS2	+	+	+	+	+	+	+	+	+	
	EGLN3	+	+	+	+	+	+	+	+	+	
	EHD4	+	+	+	+	+	+	+	+	+	
	EML1	+	+	+	+	+	+	+	+	+	
	ESRRB	+	+	+	+	+	+	+	+	+	
	EVL	+	+	+	+	+	+	+	+	+	
	FAM98B	+	+	+	+	+	+	+	+	+	
	FBLN5	+	+	+	+	+	+	+	+	+	
	FERMT2	+	+	+	+	+	+	+	+	+	
	FOS	+	+	+	+	+	+	+	+	+	
	FOXA1	+	+	+	+	+	+	+	+	+	
	FOXG1	+	+	+	+	+	+	+	+	+	
	GPR137C	+	+	+	+	+	+	+	+	+	
	HHIPL1	+	+	+	+	+	+	+	+	+	
	HIF1A	+	+	+	+	+	+	+	+	+	
	IRF2BPL	+	+	+	+	+	+	+	+	+	
	ITPKA	+	+	+	+	+	+	+	+	+	
	JAG2	+	+	+	+	+	+	+	+	+	
	KCNK10	+	+	+	+	+	+	+	+	+	
	KCNK13	+	+	+	+	+	+	+	+	+	
	KLC1	+	+	+	+	+	+	+	+	+	
	LTBP2	+	+	+	+	+	+	+	+	+	
	MAP3K9	+	+	+	+	+	+	+	+	+	
	MAP4K5	+	+	+	+	+	+	+	+	+	
	MARK3	+	+	+	+	+	+	+	+	+	
	MEIS2	+	+	+	+	+	+	+	+	+	
	MIDEAS	+	+	+	+	+	+	+	+	+	
	MTA1	+	+	+	+	+	+	+	+	+	
	NFKBIA	+	+	+	+	+	+	+	+	+	
	NRXN3	+	+	+	+	+	+	+	+	+	
	OTX2	+	+	+	+	+	+	+	+	+	
	PACS2	+	+	+	+	+	+	+	+	+	
	PAK6	+	+	+	+	+	+	+	+	+	
	PCNX1	+	+	+	+	+	+	+	+	+	
	PELI2	+	+	+	+	+	+	+	+	+	
	PLCB2	+	+	+	+	+	+	+	+	+	
	PLD4	+	+	+	+	+	+	+	+	+	
	PLEKHG3	+	+	+	+	+	+	+	+	+	
	PPM1A	+	+	+	+	+	+	+	+	+	
	PPP1R14D	+	+	+	+	+	+	+	+	+	
	PPP2R5E	+	+	+	+	+	+	+	+	+	
	PRKD1	+	+	+	+	+	+	+	+	+	
	PROX2	+	+	+	+	+	+	+	+	+	
	PYGL	+	+	+	+	+	+	+	+	+	
	RASGRP1	+	+	+	+	+	+	+	+	+	
	RCOR1	+	+	+	+	+	+	+	+	+	
	RHOV	+	+	+	+	+	+	+	+	+	
	RPS6KL1	+	+	+	+	+	+	+	+	+	

RTN1	+	+	+	+	+	+	+	+	+
SIPA1L1	+	+	+	+	+	+	+	+	+
SLC24A4	+	+	+	+	+	+	+	+	+
SLC8A3	+	+	+	+	+	+	+	+	+
SNX6	+	+	+	+	+	+	+	+	+
SPRED1	+	+	+	+	+	+	+	+	+
SPTB	+	+	+	+	+	+	+	+	+
STRN3	+	+	+	+	+	+	+	+	+
TGFB3	+	+	+	+	+	+	+	+	+
TTC9	+	+	+	+	+	+	+	+	+
TYRO3	+	+	+	+	+	+	+	+	+
VSX2	+	+	+	+	+	+	+	+	+
ZBTB42	+	+	+	+	+	+	+	+	+
ZFP36L1	+	+	+	+	+	+	+	+	+
	72	72	72	72	72	72	72	72	72

Dot chromosome 32

Paralogon 3D	Homo sapiens	Amphibia	Lepidosauria	Testudines	Crocodylia	Aves	Palaeognathae	Neognathae	Gallus gallus
ACTN4	+	+	+	+	+	+	+	+	+
AKT2	+	+	+	+	+	+	+	+	+
AXL	+	+	+	+	+	+	+	+	+
B3GNT8	+	+	+	+	+	-	-	-	-
CRX	+	+	+	+	+	+	+	+	+
DMPK	+	+	+	+	+	+	+	+	+
EGLN2	+	+	+	+	-	-	-	-	-
EHD2	+	+	+	+	+	+	+	+	+
EML2	+	+	+	+	+	-	-	-	-
FAM98C	+	+	+	+	+	+	+	-	-
FOSB	+	+	+	+	+	+	+	+	+
FOXA3	+	+	+	+	+	+	+	-	-
HIF3A	+	+	+	+	+	+	+	+	+
HNRNPUL1	+	+	+	+	+	+	+	+	+
IRF2BP1	+	+	+	+	+	+	+	+	-
ITPKC	+	+	+	+	-	-	-	-	-
KCNK6	+	+	+	+	-	+	+	+	-
KLC3	+	+	+	+	+	+	+	-	-
LTBP4	+	+	+	+	+	+	+	+	+
MAP3K10	+	+	+	+	+	+	+	-	-
MAP4K1	+	+	+	+	+	+	+	+	+
MARK4	+	+	+	+	+	+	+	+	+
MEIS3	+	+	+	+	+	-	-	-	-
NFKBIB	+	+	+	+	+	+	+	+	+
PAK4	+	+	+	+	+	+	+	+	+
PLD3	+	+	+	+	+	+	+	+	+
PLEKHG2	+	+	+	+	+	-	-	-	-
PPM1N	+	+	+	+	-	-	-	-	-
PPP1R14A	+	+	+	+	+	+	+	+	-
PRKD2	+	+	+	+	-	-	-	-	-
RASGRP4	+	+	+	+	+	+	+	+	-
RELB	+	+	+	+	+	+	+	+	+
RTN2	+	+	+	+	+	+	+	+	+
SIPA1L3	+	+	+	+	+	+	+	+	+
SLC8A2	+	+	+	+	+	+	+	+	+
SPRED3	+	+	+	+	+	+	+	+	-
STRN4	+	+	+	+	+	+	+	+	+
TGFB1	+	+	+	+	+	+	+	+	+
TTC9B	+	+	+	+	-	+	+	-	-
VASP	+	+	+	+	+	+	+	+	+
ZFP36	+	+	+	+	+	+	+	+	+
ZNF296	+	+	-	-	-	-	-	-	-
ZNF541	+	+	+	+	+	+	+	+	+
	43	43	42	42	36	34	34	29	24

mosome 36

Paralogon 13A	Homo sapiens	Amphibia	Lepidosauria	Testudines	Crocodylia	Aves	Palaeognathae	Neognathae	Gallus gallus	Notes
APLP1	+	+	+	+	+	-	-	-	-	
ARHGAP33	+	+	+	+	+	-	-	-	-	
ARHGEF1	+	+	+	+	+	+	+	+	+	
ATP1A3	+	+	+	+	+	+	+	+	+	
BCAM	+	+	+	+	+	-	-	-	-	
CADM4	+	+	+	+	+	+	+	+	+	Assigned to chicken chr36 after correction of Ggswu GenBank sequence using data from additional chicken genomes.
GRAMD1A	+	+	+	+	+	-	-	-	-	

Dot chror

GRIK5	+	+	+	+	+	+	+	+	+
HSPB6	+	+	+	+	+	-	-	-	-
IGLON5	+	+	+	+	+	+	+	-	-
KIRREL2	+	+	+	+	+	-	-	-	-
NECTIN2	+	+	+	+	+	+	+	-	-
PHLDB3	+	+	+	+	+	+	+	+	+
POU2F2	+	+	+	+	+	+	+	+	+
	14	14	14	14	14	8	8	6	6

Chromosome 24

Paralogon 13B	Homo sapiens	Amphibia	Lepidosauria	Testudines	Crocodylia	Aves	Palaeognathae	Neognathae	Gallus gallus
ADAMTS8	+	+	+	+	+	+	+	+	+
APLP2	+	+	+	+	+	+	+	+	+
ARHGAP32	+	+	+	+	+	+	+	+	+
ARHGEF12	+	+	+	+	+	+	+	+	+
BCO2	+	+	+	+	+	+	+	+	+
BTG4	+	+	+	+	+	+	+	+	+
CADM1	+	+	+	+	+	+	+	+	+
CBL	+	+	+	+	+	+	+	+	+
CRYAB	+	+	+	+	+	+	+	+	+
DRD2	+	+	+	+	+	+	+	+	+
GRAMD1B	+	+	+	+	+	+	+	+	+
GRIK4	+	+	+	+	+	+	+	+	+
JAM3	+	+	+	+	+	+	+	+	+
KCNJ1	+	+	+	+	+	+	+	+	+
KCNJ5	+	+	+	+	+	+	+	+	+
KIRREL3	+	+	+	+	+	+	+	+	+
MCAM	+	+	+	+	+	+	+	+	+
NECTIN1	+	+	+	+	+	+	+	+	+
NTM	+	+	+	+	+	+	+	+	+
PHLDB1	+	+	+	+	+	+	+	+	+
POU2F3	+	+	+	+	+	+	+	+	+
ST14	+	+	+	+	+	+	+	+	+
TAGLN	+	+	+	+	+	+	+	+	+
	23	23	23	23	23	23	23	23	23

Dot chromosome 31

Paralogons 4D6D	Homo sapiens	Amphibia	Lepidosauria	Testudines	Crocodylia	Aves	Palaeognathae	Neognathae	Gallus gallus	Notes
CACNG8	+	+	+	+	+	+	+	+	+	
CPT1C	+	+	+	+	+	+	+	+	+	
CYTH2	+	+	+	+	+	+	+	+	+	
EMP3	+	+	+	+	+	+	+	+	+	
EPN1	+	+	+	+	+	-	-	-	-	
EPS8L1	+	+	+	+	+	-	-	-	-	
GRIN2D	+	+	+	+	+	+	+	+	+	
KCNC3	+	-	-	+	+	+	+	+	+	
KCNJ14	+	+	+	+	+	-	-	-	-	
LIN7B	+	+	+	+	+	+	-	+	+	
LRRC4B	+	+	+	+	+	+	+	+	+	
MYBPC2	+	+	+	+	+	+	+	+	+	
MYH14	+	-	-	-	-	+	-	+	+	
NAPSA	+	-	-	+	+	+	-	+	+	
PLEKHA4	+	+	+	+	+	+ 1	-	+ 1	+ 1	1 In neognath birds relocalized to the paralogon 2D (chicken chr15).
PPFIA3	+	+	+	+	+	+	+	+	+	
PRKCG	+	+	+	+	+	+	+	+	+	
RCN3	+	+	+	+	+	+	+	+	+	
SCAF1	+	+	+	+	+	+	+	+	+	
SHANK1	+	+	+	+	+	+	+	+	+	
SHISA7	+	+	+	+	+	+	+	+	+	
SLC17A7	+	+	+	+	+	+	-	+	+	
SLC6A16	+	-	+	+	+	+	+	+	-	
SYT3	+	+	+	+	+	+	+	+	+	
SYT5	+	+	+	+	+	-	-	-	-	
TEAD2	+	+	+	-	+	-	-	-	-	
TMC4	+	+	+	+	+	-	-	-	-	
TNNT1	+	+	+	+	+	+	+	+	+	
TRIM28	+	+	+	+	+	+	+	+	+	
TTYH1	+	+	+	+	+	-	-	-	-	
	30	26	27	28	29	23	18	23	22	

Paralogons 4C6C	Homo sapiens	Amphibia	Lepidosauria	Testudines	Crocodylia	Aves	Palaeognathae	Neognathae	Gallus gallus
ABTB2	+	+	+	+	+	+	+	+	+

Chromosome 18 + Region on chromosome 5

ADAP2	+	+	-	+	+	+	+	+	+	
ALX4	+	+	+	+	+	+	+	+	+	
ANO1	+	+	+	+	+	+	+	+	+	
ARHGAP44	+	+	+	+	+	+	+	+	+	
ARHGDIA	+	+	+	+	+	+	+	+	+	
BAIAP2	+	+	+	+	+	+	+	+	+	
CACNA1G	+	+	+	+	+	+	+	+	+	
CACNG4	+	+	+	+	+	+	+	+	+	
CCND1	+	+	+	+	+	+	+	+	+	
CD81	+	+	+	+	+	+	+	+	+	
CHRM4	+	+	+	+	+	+	+	+	+	
CPT1A	+	+	+	+	+	+	+	+	+	
CRY2	+	+	+	+	+	+	+	+	+	
CSRP3	+	+	+	+	+	+	+	+	+	
CTSD	+	+	+	+	+	+	+	+	+	
CYB5R2	+	+	+	+	+	+	+	+	+	
CYTH1	+	+	+	+	+	+	+	+	+	
DENND2B	+	+	+	+	+	+	+	+	+	
EPN3	+	+	+	+	+	+	+	+	+	
EPS8L2	+	+	+	+	+	+	+	+	+	
GALR2	+	+	+	+	+	+	+	+	+	
GGA3	+	+	+	+	+	+	+	+	+	
GPRC5C	+	+	+	+	+	+	+	+	+	
GRB2	+	+	+	+	+	+	+	+	+	
GRIN2C	+	+	+	+	+	+	+	+	+	
GSG1L2	+	+	+	+	-	+	+	+	+	
HIPK3	+	+	+	+	+	+	+	+	+	
HRAS	+	+	+	+	+	+	+	+	+	
KCNC1	+	+	+	+	+	+	+	+	+	
KCNJ2	+	+	+	+	+	+	+	+	+	
KCTD2	+	+	+	+	+	+	+	+	+	
LGR4	+	+	+	+	+	+	+	+	+	
LIN7C	+	+	+	+	+	+	+	+	+	
LRRC4C	+	+	+	+	+	+	+	+	+	
MAFG	+	+	+	+	+	+	+	+	+	
MYBPC3	+	+	+	+	+	+	+	+	+	
MYH10	+	+	+	+	+	+	+	+	+	
MYOCD	+	+	+	+	+	+	+	+	+	
NAV2	+	+	+	+	+	+	+	+	+	
NPTX1	+	+	+	+	+	+	+	+	+	
PACSIN3	+	+	+	+	+	+	+	+	+	
PDE6G	+	+	+	+	+	+	+	+	+	
PHRF1	+	+	+	+	+	+	+	+	+	
PIK3C2A	+	+	+	+	+	+	+	+	+	
PKP3	+	+	+	+	+	+	+	+	+	
PLEKHA7	+	+	+	+	+	+	+	+	+	
PMP22	+	+	+	+	+	+	+	+	+	
PNPLA2	+	+	+	+	+	+	+	+	+	
PPFIA1	+	+	+	+	+	+	+	+	+	
PRKCA	+	+	+	+	+	+	+	+	+	
PTPN5	+	+	+	+	+	+	+	+	+	
RAC3	+	+	+	+	+	+	+	+	+	
RASSF7	+	+	+	+	+	+	+	+	+	
RBFOX3	+	+	+	+	+	+	+	+	+	
RCN1	+	+	+	+	+	+	+	+	+	
RCVRN	+	+	-	-	-	-	-	-	-	In all sauropsids and in some amphibians, RCVRN-paralog visinin (paralogon 4D) misidentified often in genome annotations as RCVRN (paralogon 4C).
RFNG	+	+	+	+	+	+	+	+	+	
SCUBE2	+	+	+	+	+	+	+	+	+	
SEPTIN9	+	+	+	+	+	+	+	+	+	
SHANK2	+	+	+	+	+	+	+	+	+	
SHISA6	+	+	+	+	+	+	+	+	+	
SLC17A6	+	+	+	+	+	+	+	+	+	
SLC5A12	+	+	+	+	+	+	+	+	+	
SOX6	+	+	+	+	+	+	+	+	+	
SOX9	+	+	+	+	+	+	+	+	+	
SSTR2	+	+	+	+	+	+	+	+	+	
SYNGR2	+	+	+	+	+	+	+	+	+	
SYT8	+	+	+	+	+	+	+	+	+	
SYT9	+	+	+	+	+	+	+	+	+	
TCP11L1	+	+	+	+	+	+	+	+	+	
TEAD1	+	+	+	+	+	+	+	+	+	

	Homo sapiens	Amphibia	Lepidosauria	Testudines	Crocodylia	Aves	Palaeognathae	Neognathae	Gallus gallus	Notes
TMC8	+	+	+	+	+	-	-	-	-	
TNNI2	+	+	+	+	+	+	+	+	+	
TNNT3	+	+	+	+	+	+	+	+	+	
TNRC6C	+	+	+	+	+	+	+	+	+	
TRIM66	+	+	+	+	+	+	+	+	+	
TSPAN4	+	+	+	+	+	+	+	+	+	
TTYH2	+	+	+	+	+	+	+	+	+	Misidentified as TTYH1 in bGalGal1.mat.broiler.GRCg7b genome annotations.
UTS2R	+	+	+	+	+	+	+	+	+	
	80	80	78	79	78	78	78	78	78	

Dot chromosome 34

Paralogon 10A	Homo sapiens	Amphibia	Lepidosauria	Testudines	Crocodylia	Aves	Palaeognathae	Neognathae	Gallus gallus	Notes
AJUBA	+	+	-	-	-	-	-	-	-	
ARHGEF40	+	+	+	+	+	-	-	-	-	
CARMIL3	+	+	+	+	+	+ 1	-	+ 1	+ 1	1 Missing in all birds except the order Galliformes. In Galliformes relocated to the paralogons 5A6A (chicken chr1).
CBLN3	+	+	-	-	-	-	-	-	-	
CHD8	+	+	+	+	+	+	+	+	+	
CPNE6	+	+	+	+	+	-	-	-	-	
EFS	+	+	+	+	+	-	-	-	-	
HNRNPC	+	+	+	+	+	+	+	+	+	
IRF9	+	+	+	+	+	+	+	+	-	Missing only in Galliformes.
LRP10	+	+	+	+	+	-	-	-	-	
MMP14	+	+	+	+	+	+	+	+	+	
NDRG2	+	+	+	+	+	+	+	+	+	
NRL	+	+	+	+	+	-	-	-	-	
PPP1R3E	+	+	+	+	+	-	-	-	-	PPP1R3EL from paralogon 10B (chicken chr11) often misidentified in genome annotations as PPP1R3E.
REM2	+	+	+	+	+	+	-	+	-	
SLC12A6	+	+	+	+	+	+	+	+	+	
TOX4	+	+	+	+	+	+	+	+	+	
TPPP2	+	+	+	+ 2	+ 2	+ 2	+ 2	+ 2	+ 2	2 Relocated to the paralogons 7A8A (chicken chrZ) in all Archelosauria.
	18	18	16	16	16	10	8	10	8	

Chromosome 11

Paralogon 10B	Homo sapiens	Amphibia	Lepidosauria	Testudines	Crocodylia	Aves	Palaeognathae	Neognathae	Gallus gallus
BCAR1	+	+	+	+	+	+	+	+	+
BCO1	+	+	+	+	+	+	+	+	+
CARMIL2	+	+	+	+	+	+	+	+	+
CBFA2T3	+	+	+	+	+	+	+	+	+
CBLN1	+	+	+	+	+	+	+	+	+
CDH1	+	+	+	+	+	+	+	+	+
CDH8	+	+	+	+	+	+	+	+	+
CEBPA	+	+	+	+	+	+	+	+	+
CHD9	+	+	+	+	+	+	+	+	+
CNGB1	+	+	+	+	+	+	+	+	+
COX4I1	+	+	+	+	+	+	+	+	+
CPNE2	+	+	+	+	+	+	+	+	+
CPNE7	+	+	+	+	+	+	+	+	+
CRISPLD2	+	+	+	+	+	+	+	+	+
DOK4	+	+	+	+	+	+	+	+	+
ELMO3	+	+	+	+	+	+	+	+	+
IRF8	+	+	+	+	+	+	+	+	+
IRX3	+	+	+	+	+	+	+	+	+
JPH3	+	+	+	+	+	+	+	+	+
LRP3	+	+	+	+	+	+	+	+	+
MAF	+	+	+	+	+	+	+	+	+
MC1R	+	+	+	+	+	+	+	+	+
MMP15	+	+	+	+	+	+	+	+	+
MYLK3	+	+	+	+	+	+	+	+	+
NDRG4	+	+	+	+	+	+	+	+	+
NFATC3	+	+	+	+	+	+	+	+	+
PARD6A	+	+	+	+	+	+	+	+	+
PDP2	+	+	+	+	+	+	+	+	+
PIEZO1	+	+	+	+	+	+	+	+	+
PLEKHG4	+	+	+	+	+	+	+	+	+
RGS9BP	+	+	+	+	+	+	+	+	+
RIPOR1	+	+	+	+	+	+	+	+	+
RRAD	+	+	+	+	+	+	+	+	+
SALL1	+	+	+	+	+	+	+	+	+
SLC12A4	+	+	+	+	+	+	+	+	+
SNAI3	+	+	+	+	+	+	+	+	-
SNTB2	+	+	+	+	+	+	+	+	+
SPIRE2	+	+	+	+	+	+	+	+	+

Dot chromosome 35

SS18L2	+	+	+	+	+	+	+	+	+
ST3GAL2	+	+	+	+	+	+	+	+	+
TOX3	+	+	+	+	+	+	+	+	+
TPPP3	+	+	+	+	+	+	+	+	+
TSHZ3	+	+	+	+	+	+	+	+	+
WTIP	+	+	+	+	+	+	+	+	+
WWP2	+	+	+	+	+	+	+	+	+
ZNF536	+	+	+	+	+	+	+	+	+
	46	46	46	46	46	46	46	46	45

Paralogons 16A17A	Homo sapiens	Amphibia	Lepidosauria	Testudines	Crocodylia	Aves	Palaeognathae	Neognathae	Gallus gallus	Notes
ACAP1	+	+	+	+	+	+	+	+	+	
AP1S1	+	+	+	+	+	+	+	+	+	
ARRB2	+	+	+	+	+	+	+	+	-	
ATP1B2	+	+	+	+	+	+	+	+	+	
CD68	+	+	+	+	+	-	-	-	-	
CHD3	+	+	+	+	+	+	+	+	+	
DLG4	+	+	+	+	+	+	+	+	+	
DVL2	+	+	+	+	+	-	-	-	-	
EFNB3	+	+	+	+	+	+	-	+	-	
ENO3	+	+	+	+	+	+	+	+	+	
EPHB4	+	+	+	+	+	+	+	+	+	
FGF11	+	+	+	+	+	+	-	+	+	
FXR2	+	+	+	+	+	+	+	-	-	
GNB2	+	+	+	+	-	-	-	-	-	
GPC2	+	+	+	+	-	-	-	-	-	
GUCY2D	+	+	+	+	+	+	+	+	+	
KCNAB3	+	+	+	+	+	+	+	+	+	
KIF1C	+	+	+	+	+	+	+	+	+	
LRCH4	+	+	+	+	+	+	+	+	+	
MINK1	+	+	+	+	+	+	+	+	-	
MOGAT3	+	+	+	+	+	+	+	+	+	
NLGN2	+	+	+	+	+	+	+	+	+	
PER1	+	+	+	+	+	+	-	+	-	
PLOD3	+	+	+	+	+	+	+	+	+	
SERPINE1	+	+	+	+	+	+	+	+	-	
SLC2A4	+	+	+	+	+	+	+	+	+	
STAG3	+	+	+	+	+	+	+	+	+	
TP53	+	+	+	+	+	+	+	+	+	
TSC22D4	+	+	+	+	+	+	+	+	+	
VAMP2	+	+	+	+	+	+	+	+	+	
YBX2	+	+	+	+	+	+	+	+	-	
ZBTB4	+	+	+	+	+	+	+	+	+	
	32	32	32	32	30	28	25	27	21	

Paralogons 16B17B	Homo sapiens	Amphibia	Lepidosauria	Testudines	Crocodylia	Aves	Palaeognathae	Neognathae	Gallus gallus	
ACAP2	+	+	+	+	+	+	+	+	+	
AMOTL2	+	+	+	+	+	+	+	+	+	
AP1S3	+	+	+	+	+	+	+	+	+	
ARHGEF4	+	+	+	+	+	+	+	+	+	
ATP11B	+	+	+	+	+	+	+	+	+	
ATP1B3	+	+	+	+	+	+	+	+	+	
CAB39	+	+	+	+	+	+	+	+	+	
CLCN2	+	+	+	+	+	+	+	+	+	
CLSTN2	+	+	+	+	+	+	+	+	+	
COL4A3	+	+	+	+	+	+	+	+	+	
CP	+	+	+	+	+	+	+	+	+	
DGKD	+	+	+	+	+	+	+	+	+	
DLG1	+	+	+	+	+	+	+	+	+	
DOCK10	+	+	+	+	+	+	+	+	+	
DVL3	+	+	+	+	+	+	+	+	+	Misidentified as DVL2 in bGalGal1.mat.broiler.GRCg7b genome annotations.
ECE2	+	+	+	+	+	+	+	+	+	
EPHB1	+	+	+	+	+	+	+	+	+	
FARP2	+	+	+	+	+	+	+	+	+	
FGF12	+	+	+	+	+	+	+	+	+	
FNDC3B	+	+	+	+	+	+	+	+	+	
FXR1	+	+	+	+	+	+	+	+	+	
GNB4	+	+	+	+	+	+	+	+	+	
GPC1	+	+	+	+	+	+	+	+	+	
GRK7	+	+	+	+	+	+	+	+	+	

Chromosome 9	HS6ST1	+	+	+	+	+	+	+	+	+
	HTR2B	+	+	+	+	+	+	+	+	+
	IRS1	+	+	+	+	+	+	+	+	+
	ITM2C	+	+	+	+	+	+	+	+	+
	KCNAB1	+	+	+	+	+	+	+	+	+
	KIF1A	+	+	+	+	+	+	+	+	+
	LAMP3	+	+	+	+	+	+	+	+	+
	LRCH3	+	+	+	+	+	+	+	+	+
	MASP1	+	+	+	+	+	+	+	+	+
	MBNL1	+	+	+	+	+	+	+	+	+
	MCF2L2	+	+	+	+	+	+	+	+	+
	NGEF	+	+	+	+	+	+	+	+	+
	NLGN1	+	+	+	+	+	+	+	+	+
	P3H2	+	+	+	+	+	+	+	+	+
	PAK2	+	+	+	+	+	+	+	+	+
	PER2	+	+	+	+	+	+	+	+	+
	PHC3	+	+	+	+	+	+	+	+	+
	PLOD2	+	+	+	+	+	+	+	+	+
	PLS1	+	+	+	+	+	+	+	+	+
	RAP2B	+	+	+	+	+	+	+	+	+
	RBP1	+	+	+	+	+	+	+	+	+
	SAG	+	+	+	+	+	+	+	+	+
	SERPINE2	+	+	+	+	+	+	+	+	+
	SLC2A2	+	+	+	+	+	+	+	+	+
	SLC9A9	+	+	+	+	+	+	+	+	+
	SLITRK3	+	+	+	+	+	+	+	+	+
	SOX2	+	+	+	+	+	+	+	+	+
	ST6GAL1	+	+	+	+	+	+	+	+	+
	STAG1	+	+	+	+	+	+	+	+	+
	STK25	+	+	+	+	+	+	+	+	+
	TNIK	+	+	+	+	+	+	+	+	+
	TP63	+	+	+	+	+	+	+	+	+
	TRPC1	+	+	+	+	+	+	+	+	+
	TSC22D2	+	+	+	+	+	+	+	+	+
	ZBTB38	+	+	+	+	+	+	+	+	+
	ZIC1	+	+	+	+	+	+	+	+	+
		60	60	60	60	60	60	60	60	60

Supplementary Table 1 is based on the data retrieved from NCBI Orthologs resources in Nov. - Dec. 2024.
Gene orthologs came from the genomes of the following species:

amphibia	*Bombina bombina*
amphibia	*Bufo bufo*
amphibia	*Bufo gargarizans*
amphibia	*Eleutherodactylus coqui*
amphibia	*Geotrypetes seraphini*
amphibia	*Hyla sarda*
amphibia	*Hyperolius riggenbachi*
amphibia	*Microcaecilia unicolor*
amphibia	*Nanorana parkeri*
amphibia	*Pelobates fuscus*
amphibia	*Pleurodeles waltl*
amphibia	*Pseudophryne corroboree*
amphibia	*Rana temporaria*
amphibia	*Rhinatrema bivittatum*
amphibia	*Spea bombifrons*
amphibia	*Xenopus laevis*
amphibia	*Xenopus tropicalis*
aves	*Acanthisitta chloris*
aves	*Accipiter gentilis*
aves	*Agelaius phoeniceus*
aves	*Ammospiza caudacuta*
aves	*Ammospiza nelsoni*
aves	*Anas acuta*
aves	*Anas platyrhynchos*
aves	*Anomalospiza imberbis*
aves	*Anser cygnoides*
aves	*Antrostomus carolinensis*
aves	*Apaloderma vittatum*

aves	*Aphelocoma coerulescens*
aves	*Aptenodytes forsteri*
aves	*Apteryx mantelli*
aves	*Apteryx rowi*
aves	*Apus apus*
aves	*Aquila chrysaetos*
aves	*Athene cunicularia*
aves	*Aythya fuligula*
aves	*Balearica regulorum*
aves	*Buceros rhinoceros*
aves	*Calidris pugnax*
aves	*Caloenas nicobarica*
aves	*Calypte anna*
aves	*Camarhynchus parvulus*
aves	*Cariama cristata*
aves	*Catharus ustulatus*
aves	*Centrocercus urophasianus*
aves	*Chaetura pelagica*
aves	*Chamaea fasciata*
aves	*Charadrius vociferus*
aves	*Chiroxiphia lanceolata*
aves	*Chlamydotis macqueenii*
aves	*Chroicocephalus ridibundus*
aves	*Cinclus cinclus*
aves	*Colius striatus*
aves	*Columba livia*
aves	*Corapipo altera*
aves	*Corvus brachyrhynchos*
aves	*Corvus cornix*
aves	*Corvus hawaiiensis*
aves	*Corvus kubaryi*
aves	*Corvus moneduloides*
aves	*Coturnix japonica*
aves	*Cuculus canorus*
aves	*Cyanistes caeruleus*
aves	*Cygnus atratus*
aves	*Cygnus olor*
aves	*Cyrtonyx montezumae*
aves	*Dromaius novaehollandiae*
aves	*Dryobates pubescens*
aves	*Egretta garzetta*
aves	*Empidonax traillii*
aves	*Eurypyga helias*
aves	*Falco biarmicus*
aves	*Falco cherrug*
aves	*Falco naumanni*
aves	*Falco peregrinus*
aves	*Falco rusticolus*
aves	*Ficedula albicollis*
aves	*Fulmarus glacialis*
aves	*Gallus gallus*
aves	*Gavia stellata*
aves	*Geospiza fortis*
aves	*Grus americana*
aves	*Gymnogyps californianus*
aves	*Haemorhous mexicanus*
aves	*Haliaeetus albicilla*
aves	*Haliaeetus leucocephalus*
aves	*Harpia harpyja*
aves	*Hirundo rustica*
aves	*Indicator indicator*
aves	*Lagopus leucura*
aves	*Lagopus muta*
aves	*Lathamus discolor*
aves	*Lepidothrix coronata*
aves	*Leptosomus discolor*
aves	*Lonchura striata*
aves	*Malurus melanocephalus*
aves	*Manacus candei*
aves	*Manacus vitellinus*
aves	*Melanerpes formicivorus*

aves	*Meleagris gallopavo*
aves	*Melopsittacus undulatus*
aves	*Melospiza georgiana*
aves	*Melospiza melodia*
aves	*Melozone crissalis*
aves	*Merops nubicus*
aves	*Mesitornis unicolor*
aves	*Molothrus aeneus*
aves	*Molothrus ater*
aves	*Motacilla alba*
aves	*Myiozetetes cayanensis*
aves	*Neopelma chrysocephalum*
aves	*Neopsephotus bourkii*
aves	*Nestor notabilis*
aves	*Nipponia nippon*
aves	*Nothoprocta perdicaria*
aves	*Numida meleagris*
aves	*Nyctibius grandis*
aves	*Oenanthe melanoleuca*
aves	*Onychostruthus taczanowskii*
aves	*Opisthocomus hoazin*
aves	*Oxyura jamaicensis*
aves	*Parus major*
aves	*Passer domesticus*
aves	*Passer montanus*
aves	*Patagioenas fasciata*
aves	*Pelecanus crispus*
aves	*Pezoporus flaviventris*
aves	*Pezoporus occidentalis*
aves	*Pezoporus wallicus*
aves	*Phaethon lepturus*
aves	*Phalacrocorax carbo*
aves	*Phasianus colchicus*
aves	*Pipra filicauda*
aves	*Poecile atricapillus*
aves	*Pogoniulus pusillus*
aves	*Prinia subflava*
aves	*Pseudopipra pipra*
aves	*Pseudopodoces humilis*
aves	*Pterocles gutturalis*
aves	*Pygoscelis adeliae*
aves	*Pyrgilauda ruficollis*
aves	*Rhea pennata*
aves	*Rissa tridactyla*
aves	*Serinus canaria*
aves	*Strigops habroptila*
aves	*Struthio camelus*
aves	*Sturnus vulgaris*
aves	*Sylvia atricapilla*
aves	*Taeniopygia guttata*
aves	*Tauraco erythrolophus*
aves	*Tinamus guttatus*
aves	*Tympanuchus pallidicinctus*
aves	*Tyto alba*
aves	*Vidua chalybeata*
aves	*Vidua macroura*
aves	*Zonotrichia albicollis*
aves	*Zonotrichia leucophrys*
crocodylia	*Alligator mississippiensis*
crocodylia	*Alligator sinensis*
crocodylia	*Crocodylus porosus*
crocodylia	*Gavialis gangeticus*
lepidosauria	*Ahaetulla prasina*
lepidosauria	*Anolis carolinensis*
lepidosauria	*Anolis sagrei*
lepidosauria	*Candoia aspera*
lepidosauria	*Crotalus tigris*
lepidosauria	*Elgaria multicarinata*
lepidosauria	*Eublepharis macularius*
lepidosauria	*Euleptes europaea*
lepidosauria	*Gekko japonicus*

lepidosauria	*Hemicordylus capensis*
lepidosauria	*Heteronotia binoei*
lepidosauria	*Lacerta agilis*
lepidosauria	*Notechis scutatus*
lepidosauria	*Pantherophis guttatus*
lepidosauria	*Podarcis muralis*
lepidosauria	*Podarcis raffonei*
lepidosauria	*Pogona vitticeps*
lepidosauria	*Protobothrops mucrosquamatus*
lepidosauria	*Pseudonaja textilis*
lepidosauria	*Python bivittatus*
lepidosauria	*Rhineura floridana*
lepidosauria	*Sceloporus undulatus*
lepidosauria	*Sphaerodactylus townsendi*
lepidosauria	*Thamnophis elegans*
lepidosauria	*Thamnophis sirtalis*
lepidosauria	*Tiliqua scincoides*
lepidosauria	*Varanus komodoensis*
lepidosauria	*Zootoca vivipara*
testudines	*Caretta caretta*
testudines	*Chelonia mydas*
testudines	*Chelonoidis abingdonii*
testudines	*Chrysemys picta*
testudines	*Dermochelys coriacea*
testudines	*Emydura macquarii*
testudines	*Emys orbicularis*
testudines	*Gopherus evgoodei*
testudines	*Gopherus flavomarginatus*
testudines	*Malaclemys terrapin*
testudines	*Mauremys mutica*
testudines	*Mauremys reevesii*
testudines	*Pelodiscus sinensis*
testudines	*Terrapene triunguis*
testudines	*Trachemys scripta*

Supplementary table 2

Table 2A

Ohnologs missing from DOT chromosomes in all birds				
Genes	On DOT chromosomes		n isoparalogons on non-DOT chromosomes	
Total number searched	291		525	
	Missing (count)	Percent	Missing (count)	Percent
Missing in all sauropsids	4	1.4%	1	0.2%
Missing in all archosaurs	31	11%	1	0.2%
Missing in all birds	85	29%	2	0.4%
Missing in chicken	127	44%	4	0.8%

Table 2B

Ohnologs missing from DOT chromosomes in all birds				
Genes missing in all birds			Missing in Crocodylia	Missing in all Sauropsids
Gene	Paralogon	Chromosome		
AJUBA	10A	chr34	YES	YES
ANGPTL6	8D9D	chr30	YES	.
APLP1	13A	chr36	.	.
ARAF	5C	chr29	YES	.
ARHGAP33	13A	chr36	.	.
ARHGAP4	5C	chr29	.	.
ARHGEF40	10A	chr34	.	.
ATL3	3B	chr37	.	.
ATP6V1G2	9B	chr16	.	.
B3GNT8	3D	chr32	.	.
BCAM	13A	chr36	.	.
BCL7C	2B	chr38	YES	.
CBLN3	10A	chr34	YES	YES
CCDC85B	3B	chr37	YES	.
CCDC88B	3B	chr37	.	.
CD68	16A17A	chr35	.	.
CDC42BPG	3B	chr37	.	.
CHRM1	3B	chr37	.	.
CORO1A	2B	chr38	YES	.
CPNE6	10A	chr34	.	.
DENND1C	8D9D	chr30	.	.
DOC2A	2B	chr38	YES	.
DOCK6	8D9D	chr30	.	.
DUSP9	5C	chr29	.	.
DVL2	16A17A	chr35	.	.
EFS	10A	chr34	.	.
EGLN2	3D	chr32	YES	.
EHD1	3B	chr37	.	.
ELK1	5C	chr29	.	.
EML2	3D	chr32	.	.
EPN1	4D6D	chr31	.	.
EPS8L1	4D6D	chr31	.	.
FGD1	5C	chr29	YES	.
FOSL1	3B	chr37	.	.
GDI1	5C	chr29	.	.
GNB2	16A17A	chr35	YES	.
GPC2	16A17A	chr35	YES	.
GPR137	3B	chr37	YES	.
GRAMD1A	13A	chr36	.	.
HSPB6	13A	chr36	.	.
ITPKC	3D	chr32	YES	.
KCND1	5C	chr29	.	.
KCNJ14	4D6D	chr31	.	.
KCNK4	3B	chr37	.	.
KCNK7	3B	chr37	YES	.
KCTD13	2B	chr38	.	.
KIRREL2	13A	chr36	.	.
KLC2	3B	chr37	YES	.
LRP10	10A	chr34	.	.
LRRC8E	8D9D	chr30	.	.
MEIS3	3D	chr32	.	.
NOTCH4	9B	chr16	YES	.
NRL	10A	chr34	.	.
ORAI3	2B	chr38	YES	.
PALM3	8D9D	chr30	.	.
PELI3	3B	chr37	YES	.
PKN1	8D9D	chr30	.	.
PLEKHG2	3D	chr32	.	.
PLXNB3	5C	chr29	.	.
PNCK	5C	chr29	YES	.
PPM1N	3D	chr32	YES	.
PPP1R3E	10A	chr34	.	.
PPP2R5B	3B	chr37	.	.

Table 2D

Additional ohnologs missing from DOT chromosomes in chicken						
Additional genes missing in chicken			Missing in other Galliformes	Missing in Anseriformes	Missing in Neoaves	Missing in Palaeognathes
Gene	Paralogon	Chromosome				
ACTN3	3B	chr37	YES	YES	YES	.
ARRB2	16A17A	chr35	YES	YES	.	.
ATP2B3	5C	chr29	YES	YES	.	.
BGN	5C	chr29	YES	YES	YES	.
COL5A3	8D9D	chr30	YES	.	.	.
EFNB3	16A17A	chr35	YES	.	.	YES
FAM98C	3D	chr32	YES	YES	YES	.
FBXL19	2B	chr38	YES	.	.	.
FOXA3	3D	chr32	YES	YES	YES	.
FXR2	16A17A	chr35	YES	YES	YES	.
IGLON5	13A	chr36	YES	YES	YES	.
IRAK1	5C	chr29	YES	YES	.	.
IRF2BP1	3D	chr32	YES	YES	.	.
IRF9	10A	chr34	YES	.	.	.
KANK2	8D9D	chr30	YES	.	.	.
KCNK6	3D	chr32	YES	YES	.	.
KLC3	3D	chr32	YES	YES	YES	.
MAGIX	5C	chr29	YES	YES	.	YES
MAP3K10	3D	chr32	YES	YES	YES	.
MAP4K2	3B	chr37	YES	YES	.	.
MINK1	16A17A	chr35	YES	.	YES	.
NECTIN2	13A	chr36	YES	YES	YES	.
NOTCH3	8D9D	chr30	YES	.	.	.
PCNX3	3B	chr37	YES	YES	.	.
PDE4A	8D9D	chr30	YES	.	.	.
PER1	16A17A	chr35	YES	YES	.	YES
PLXNA3	5C	chr29	YES	YES	YES	.
PPP1R14A	3D	chr32	YES	.	.	.
RASGRP2	3B	chr37	YES	YES	.	.
RASGRP4	3D	chr32	YES	.	.	.
REM2	10A	chr34	YES	YES	.	YES
SERPINE1	16A17A	chr35	YES	YES	.	.
SEZ6L2	2B	chr38	YES	YES	.	.
SLC6A16	4D6D	chr31	YES	YES	.	.
SNX15	3B	chr37	YES	YES	YES	.
SPRED3	3D	chr32	YES	YES	.	.
STXBP2	8D9D	chr30	YES	.	YES	.
TGFB1I1	2B	chr38	YES	.	.	.
TTC9B	3D	chr32	YES	YES	YES	.
TTC9C	3B	chr37	YES	YES	YES	.
WNK3	5C	chr29	YES	.	.	.
YBX2	16A17A	chr35	YES	.	.	.

Table 2E

Additional genes missing from other chromosomes						
Additional genes missing in chicken			Missing in other Galliformes	Missing in Anseriformes	Missing in Neoaves	Missing in Palaeognathes
Gene	Paralogon	Chromosome				
DIRAS3	8C9C	chr8	YES	.	.	.
SNAI3	10B	chr11	YES	.	.	.

Table 2F

Additional ohnologs missing from other chromosomes		
Genes missing in all birds		
Gene	Paralogon	Chromosome
APH1B	7D	chr10
AVIL	1B	chr33
BTG3	13D	chr1
C2CD4D	7C13C	chr25
CALCOCO1	1B	chr33
CRTC3	7D	chr10
CYB5R1	5B6B	chr26
DKK4	14C	chr22
DUSP2	14C	chr22
KLF16	7B8B	chr28
LINGO4	7C13C	chr25
MARCHF9	1B	chr33
PAX8	14C	chr22
RASSF4	14D	chr6
RXFP4	7C13C	chr25
TMEM30B	11B12B	chr23
TNFAIP8L2	7C13C	chr25
UBE2D4	14C	chr22

Table 2G

Additional ohnologs missing from other chromosomes		
Genes missing in all birds		
Gene	Paralogon	Chromosome
ARHGEF19	17C	chr21
COX4I2	10C	chr20
ELF4	15C	chr4
F12	14B	chr13
PDE6A	14B	chr13
TSPAN17	14B	chr13

PRKD2	3D	chr32	YES	.
PTGER1	8D9D	chr30	.	.
RAB3D	8D9D	chr30	.	.
RGL3	8D9D	chr30	YES	.
RIN1	3B	chr37	YES	.
ROM1	3B	chr37	YES	YES
SEPTIN1	2B	chr38	YES	.
SH2D3A	8D9D	chr30	.	.
SLC25A23	8D9D	chr30	.	.
SLC38A5	5C	chr29	.	.
SLC44A4	9B	chr16	.	.
SNX32	3B	chr37	YES	.
SYT5	4D6D	chr31	.	.
TEAD2	4D6D	chr31	.	.
TMC4	4D6D	chr31	.	.
TMEM151A	3B	chr37	YES	.
TRIP10	8D9D	chr30	.	.
TTYH1	4D6D	chr31	.	.
USP11	5C	chr29	YES	.
YPEL3	2B	chr38	.	.
ZNF296	3D	chr32	YES	YES
ZSWIM4	8D9D	chr30	YES	.

Table 2C

Genes missing from isoparalogons on non-DOT chromosomes				
Genes missing in all birds			Missing in Crocodylia	Missing in all Sauropsids
Gene	Paralogon	Chromosome		
RCVRN	4C	chr18	YES	YES
TMC8	4C	chr18	.	.

Supplementary table 3

PHF8 / PHF2 Ohnologs

Gene PHF8 located on Gallus gallus breed Huxu chromosome 29 (dot chromosome)

Results from genome assembly GGswu				Results from public Oxford Nanopore sequencing data					
Intron name	Position	Intron length [bp]	Coverage by simple repeats	Samples analyzed (after filtering)	Intron lengths [bp]	# samples with single length allele	# samples with different length allels	# intron length alleles	Source of intron length variation
1	CP100583.2:1309390-1311848	2458	94.67%	1, 4, 5, 6, 7, 8, 9, 10, 11	1294, 1602, 1779, 1973, 2215, 2453	7	2	6	
2	CP100583.2:1312228-1315480	3252	94.71%	1, 4, 5, 6, 7, 9, 10, 11	1996, 2427, 2739, 3045, 3716, 6639	3	5	6	
3	CP100583.2:1315589-1318272	2683	89.15%	1, 4, 6, 7, 9, 10, 11	1267, 1394, 1611, 1943, 2044, 2455, 2861	3	4	7	
4	CP100583.2:1318368-1320093	1725	69.45%	1, 4, 5, 7, 8, 9, 10, 11	1198, 1324, 1654, 1878, 2415, 2722, 4756	3	5	7	
5	CP100583.2:1320365-1324006	3641	70.91%	1, 4, 7, 8, 9, 10, 11	1766, 2094, 2351, 2872, 3369	6	1	5	
6	CP100583.2:1324110-1325567	1457	55.73%	4, 6, 7, 8, 9, 10, 11	1085, 1183, 1361, 1530	6	1	4	
7	CP100583.2:1325652-1332701	7049	98.71%	1, 4, 5, 6, 7, 8, 10, 11	870, 1137, 1428, 1663, 2011, 6008	7	1	6	
8	CP100583.2:1332844-1333712	868	52.65%	1, 4, 5, 6, 7, 8, 10, 11	519, 596, 667	7	1	3	
9	CP100583.2:1333788-1335405	1617	54.98%	1, 4, 5, 6, 7, 8, 9, 10, 11	1082, 1274, 1471, 2795, 3000, 3418, 5512	6	3	7	
10	CP100583.2:1335594-1338461	2867	97.31%	1, 4, 5, 6, 8, 9, 10, 11	1719, 2048, 2686, 3594, 4245, 5088	7	1	6	
11	CP100583.2:1338550-1343365	4815	61.54%	1, 4, 5, 6, 7, 8, 9, 10, 11	1197, 1344, 1711, 1826, 2244, 2867, 3850	5	4	7	
12	CP100583.2:1343458-1344195	737	79.38%	1, 4, 5, 6, 7, 8, 9, 10, 11	664, 855, 1294, 1605, 1889, 2264, 2529	4	5	7	
13	CP100583.2:1344297-1345544	1247	87.65%	1, 4, 5, 6, 7, 9, 10, 11	1080, 1227, 1366, 1679, 1874, 2092, 2489, 3639	2	6	8	
14	CP100583.2:1345630-1348150	2520	90.99%	1, 4, 5, 7, 9, 10, 11	1758, 2448, 2940, 3760, 4620	2	5	5	
15	CP100583.2:1348313-1352696	4383	81.61%	1, 4, 5, 6, 8, 9, 10, 11	1788, 2686, 3069, 3540, 4254, 5385, 7700	4	4	7	
16	CP100583.2:1352881-1357696	4815	85.92%	1, 4, 6, 7, 8, 9, 10, 11	3741, 4270, 4921, 5330, 6187	6	2	5	
17	CP100583.2:1357836-1361677	3841	91.49%	1, 4, 5, 6, 7, 8, 9, 10, 11	1293, 1435, 2008, 2723, 3253, 3656, 5374	5	4	7	
18	CP100583.2:1361837-1364378	2541	89.57%	1, 4, 5, 6, 7, 8, 9, 10, 11	1582, 1746, 2102, 2427, 2890	5	4	5	
19	CP100583.2:1364492-1366878	2386	89.15%	1, 4, 5, 6, 7, 8, 9, 10, 11	2008, 2242, 2484	8	1	3	
20	CP100583.2:1366963-1370116	3153	93.78%	1, 4, 5, 6, 7, 8, 9, 10, 11	1433, 1851, 2247, 2523, 3394	5	4	5	

* SRX11722867[1], SRX11722868[2], SRX11722871[3], SRX14125033[4], SRX14125034[5], SRX19311940[6], SRX19311957[7], SRX19311958[8], SRX19311959[9], SRX19311960[10], SRX19311961[11]

Gene PHF2 located on Gallus gallus breed Huxu chromosome 2 (normal chromosome)

Results from genome assembly GGswu				Results from public Oxford Nanopore sequencing data					
Intron name	Position	Intron length [bp]	Coverage by simple repeats	Samples analyzed (after filtering)	Intron lengths [bp]	# samples with single length allele	# samples with different length allels	# intron length alleles	Source of intron length variation
1	CP100566.1:6939275-6941562	2287	1.71%	1, 2, 3, 4, 6, 7, 8, 9, 10, 11	2239	10	0	1	
2	CP100566.1:6941823-6942955	1132	0.00%	1, 2, 3, 4, 5, 6, 8, 9, 10, 11	1106	10	0	1	
3	CP100566.1:6943064-6943711	647	0.00%	1, 2, 3, 4, 5, 6, 8, 9, 10, 11	636	10	0	1	
4	CP100566.1:6943806-6944301	495	0.00%	1, 2, 3, 4, 5, 6, 7, 8, 9, 10, 11	485	11	0	1	
5	CP100566.1:6944578-6951104	6526	0.00%	1, 2, 3, 4, 5, 6, 7, 8, 9, 10, 11	6341	11	0	1	
6	CP100566.1:6951261-6953010	1749	0.00%	1, 2, 3, 4, 5, 6, 7, 8, 9, 10, 11	1723	11	0	1	
7	CP100566.1:6953083-6953300	217	0.00%	1, 2, 3, 4, 5, 6, 7, 8, 9, 10	212	10	0	1	
8	CP100566.1:6953478-6954094	616	0.00%	1, 2, 3, 4, 5, 6, 7, 8, 9, 10, 11	532, 606	6	5	2	single insertion
9	CP100566.1:6954212-6955168	956	5.13%	1, 2, 3, 4, 5, 6, 8, 9, 10, 11	922	10	0	1	
10	CP100566.1:6955287-6956058	771	3.76%	1, 2, 3, 4, 5, 6, 7, 8, 9, 10, 11	741	11	0	1	
11	CP100566.1:6956429-6957664	1235	0.00%	1, 2, 3, 4, 5, 9, 10, 11	1218	8	0	1	

**12**	CP100566.1:6957753-6959052	1299	0.00%	2, 3, 4, 5, 7, 9, 10, 11	1272	8	0	1	
**13**	CP100566.1:6959143-6960335	1192	0.00%	1, 2, 3, 4, 5, 6, 7, 8, 9, 11	1168	10	0	1	
**14**	CP100566.1:6960441-6960933	492	0.00%	1, 2, 3, 4, 5, 6, 7, 8, 10, 11	486	10	0	1	
**15**	CP100566.1:6961020-6961947	927	3.02%	1, 2, 3, 4, 5, 6, 7, 9, 10, 11	907	10	0	1	
**16**	CP100566.1:6962109-6962637	528	0.00%	1, 2, 3, 4, 5, 6, 7, 8, 9, 10, 11	520	11	0	1	
**17**	CP100566.1:6962823-6964366	1543	0.00%	1, 2, 3, 4, 5, 6, 7, 8, 9, 10, 11	1519	11	0	1	
**18**	CP100566.1:6964507-6967322	2815	1.78%	1, 2, 3, 4, 5, 6, 7, 8, 9, 10, 11	2732	11	0	1	
**19**	CP100566.1:6967482-6971685	4203	0.00%	1, 2, 3, 4, 5, 6, 7, 8, 9, 10, 11	4111	11	0	1	
**20**	CP100566.1:6971799-6976584	4785	1.11%	1, 2, 3, 4, 5, 6, 7, 8, 9, 10, 11	4665	11	0	1	
**21**	CP100566.1:6976669-7019492	42823	1.08%	1, 2, 3, 5, 6, 7, 9, 10	41772	8	0	1	

* SRX11722867[1], SRX11722868[2], SRX11722871[3], SRX14125033[4], SRX14125034[5], SRX19311940[6], SRX19311957[7], SRX19311958[8], SRX19311959[9], SRX19311960[10], SRX19311961[11]

AKT2 / AKT1 Ohnologs

**Gene AKT2 located on Gallus gallus breed Huxu chromosome 32 (dot chromosome)**									
**Results from genome assembly GGswu**				**Results from public Oxford Nanopore sequencing data**					
**Intron name**	**Position**	**Intron length [bp]**	**Coverage by simple repeats**	**Samples analyzed (after filtering)**	**Intron lengths [bp]**	**# samples with single length allele**	**# samples with different length allels**	**# intron length alleles**	**Source of intron length variation**
**1**	CP100586.2:2596369-2598200	1831	76.41%	1, 2, 3, 4, 5, 6, 7, 8, 9, 10, 11	1790, 2388, 2927, 3380, 4015	5	6	5	
**2**	CP100586.2:2598328-2600206	1878	85.04%	1, 2, 3, 4, 5, 6, 7, 9, 10, 11	982, 1236, 1353, 1635, 1848	5	5	5	
**3**	CP100586.2:2600318-2600451	133	51.13%	1, 2, 3, 6, 8, 9	104, 117, 126, 139	1	5	4	
**4**	CP100586.2:2600606-2602123	1517	91.56%	1, 2, 3, 4, 5, 6, 7, 8, 10, 11	901, 966, 1109, 1261, 1427	4	6	5	
**5**	CP100586.2:2602254-2603282	1028	67.80%	1, 2, 3, 4, 5, 6, 7, 8, 9, 10, 11	715, 869, 1008, 1210, 1453, 1831	3	8	6	
**6**	CP100586.2:2603347-2604361	1014	54.34%	1, 2, 3, 4, 5, 6, 7, 8, 9, 10, 11	922, 1012	9	2	2	
**7**	CP100586.2:2604429-2604551	122	51.64%	1, 2, 3, 6, 8, 9, 11	102, 114, 128	2	5	3	
**8**	CP100586.2:2604673-2605544	871	44.20%	1, 2, 3, 4, 6, 7, 8, 9, 10	676, 779, 829	8	1	3	
**9**	CP100586.2:2605672-2606144	472	0.00%	1, 2, 3, 4, 5, 6, 7, 8, 9, 10, 11	363, 466	6	5	2	
**10**	CP100586.2:2606357-2606921	564	42.20%	1, 2, 3, 5, 6, 8, 9, 10, 11	480, 538	5	4	2	
**11**	CP100586.2:2607008-2608433	1425	96.63%	1, 2, 3, 4, 5, 6, 7, 8, 9, 10, 11	1277, 1368, 1744	9	2	3	
**12**	CP100586.2:2608535-2608648	113	39.82%	1, 3, 5, 7, 8	96, 110, 122	1	4	3	

* SRX11722867[1], SRX11722868[2], SRX11722871[3], SRX14125033[4], SRX14125034[5], SRX19311940[6], SRX19311957[7], SRX19311958[8], SRX19311959[9], SRX19311960[10], SRX19311961[11]

**Gene AKT1 located on Gallus gallus breed Huxu chromosome 5 (normal chromosome)**									
**Results from genome assembly GGswu**				**Results from public Oxford Nanopore sequencing data**					
**Intron name**	**Position**	**Intron length [bp]**	**Coverage by simple repeats**	**Samples analyzed (after filtering)**	**Intron lengths [bp]**	**# samples with single length allele**	**# samples with different length allels**	**# intron length alleles**	**Source of intron length variation**
**1**	CP100559.1:51151033-51151746	713	0.00%	1, 2, 3, 4, 5, 6, 8, 9, 10, 11	696	10	0	1	
**2**	CP100559.1:51151848-51156894	5046	0.00%	1, 2, 3, 4, 5, 6, 7, 8, 9, 10, 11	4919	11	0	1	
**3**	CP100559.1:51156981-51163634	6653	1.22%	1, 2, 3, 4, 5, 6, 7, 8, 9, 10, 11	6501	11	0	1	
**4**	CP100559.1:51163848-51167911	4063	0.69%	1, 2, 3, 4, 5, 7, 8, 9, 10, 11	3984	10	0	1	
**5**	CP100559.1:51168039-51169318	1279	0.00%	1, 2, 3, 4, 5, 8, 9, 10, 11	1256	9	0	1	
**6**	CP100559.1:51169443-51170147	704	0.00%	1, 2, 3, 4, 5, 6, 7, 8, 9, 10, 11	696	11	0	1	
**7**	CP100559.1:51170215-51171832	1617	0.00%	1, 2, 3, 4, 7, 8, 10, 11	1588	8	0	1	
**8**	CP100559.1:51171897-51172065	168	0.00%	2, 3, 4, 9, 11	168, 192	4	1	2	diffuse variation
**9**	CP100559.1:51172196-51173724	1528	0.00%	1, 2, 3, 4, 5, 6, 8, 10, 11	1504	9	0	1	

**10**	CP100559.1:51173871-51176767	2896	0.00%	1, 2, 3, 4, 5, 6, 7, 8, 9, 10, 11	2833	11	0	1	
**11**	CP100559.1:51176878-51183327	6449	0.67%	1, 2, 3, 4, 5, 6, 7, 8, 9, 10, 11	6305	11	0	1	
**12**	CP100559.1:51183455-51213332	29877	0.60%	1, 2, 3, 4, 5, 6, 7, 8, 9, 10, 11	22710, 29230	10	1	2	
**13**	CP100559.1:51213518-51219768	6250	1.10%	1, 2, 3, 4, 5, 6, 7, 8, 10, 11	6072	10	0	1	

* SRX11722867[1], SRX11722868[2], SRX11722871[3], SRX14125033[4], SRX14125034[5], SRX19311940[6], SRX19311957[7], SRX19311958[8], SRX19311959[9], SRX19311960[10], SRX19311961[11]

LFNA / LFNB Ohnologs

**Gene LFNA located on Gallus gallus breed Huxu chromosome 29 (dot chromosome)**									
**Results from genome assembly GGswu**				**Results from public Oxford Nanopore sequencing data**					
**Intron name**	**Position**	**Intron length [bp]**	**Coverage by simple repeats**	**Samples analyzed (after filtering)**	**Intron lengths [bp]**	**# samples with single length allele**	**# samples with different length allels**	**# intron length alleles**	**Source of intron length variation**
**1**	CP100583.2:672913-676953	4040	42.50%	1, 2, 3, 4, 5, 6, 7, 8, 9, 10, 11	3725	11	0	1	
**2**	CP100583.2:677165-678755	1590	90.31%	1, 2, 3, 4, 5, 6, 7, 8, 9, 10, 11	1425, 1543, 1840, 1976	7	4	4	
**3**	CP100583.2:678852-680869	2017	79.97%	1, 2, 3, 5, 7, 8, 9, 10, 11	1326, 1556, 1695, 1894, 2025	5	4	5	
**4**	CP100583.2:681017-682267	1250	76.64%	1, 2, 3, 4, 6, 7, 8, 9, 10, 11	936, 1122	9	1	2	
**5**	CP100583.2:682385-682987	602	62.62%	1, 2, 3, 4, 5, 6, 7, 8, 10, 11	539, 595, 662	6	4	3	
**6**	CP100583.2:683064-685304	2240	90.49%	1, 2, 3, 4, 5, 6, 7, 8, 9, 10, 11	1831, 2148, 2414, 2610, 3597, 4433, 5730	6	5	7	
**7**	CP100583.2:685466-686270	804	76.49%	1, 2, 3, 4, 5, 7, 10, 11	741, 825	8	0	2	
**8**	CP100583.2:686464-687090	626	40.42%	1, 2, 3, 4, 5, 6, 7, 8, 9, 10, 11	535, 578, 613	7	4	3	
**9**	CP100583.2:687184-688844	1660	91.57%	1, 2, 3, 4, 5, 6, 7, 8, 9, 10, 11	869, 961, 1586, 1650	5	6	4	
**10**	CP100583.2:688981-690415	1434	83.61%	1, 2, 3, 4, 5, 6, 7, 8, 9, 10, 11	1131, 1357	6	5	2	
**11**	CP100583.2:690538-691353	815	67.12%	1, 2, 3, 4, 5, 7, 9, 10, 11	544, 646, 720, 789	4	5	4	
**12**	CP100583.2:691488-692677	1189	73.76%	1, 2, 3, 4, 5, 6, 7, 8, 9, 10, 11	886, 970, 1105, 1294, 2028	3	8	5	
**13**	CP100583.2:692870-695224	2354	86.32%	1, 2, 3, 4, 5, 6, 7, 8, 9, 10, 11	1319, 1756, 2009, 2217, 2826	8	3	5	
**14**	CP100583.2:695337-696484	1147	70.79%	1, 2, 3, 5, 6, 7, 8, 9, 10, 11	847, 930, 1051, 1156	4	6	4	
**15**	CP100583.2:696627-697599	972	88.27%	1, 2, 3, 4, 5, 7, 8, 9, 10, 11	642, 872, 937, 1122	6	4	4	
**16**	CP100583.2:697722-698743	1021	95.79%	1, 2, 3, 4, 5, 6, 7, 8, 9, 10, 11	837, 1092, 1591, 2204, 2705, 3077, 4576, 5496, 6109, 7598	3	8	10	
**17**	CP100583.2:698902-702241	3339	97.06%	1, 2, 3, 4, 5, 6, 7, 8, 9, 10, 11	851, 1013, 1112, 1278, 1522, 1737, 2084, 3065, 3709	4	7	9	
**18**	CP100583.2:702331-703851	1520	95.39%	1, 2, 3, 4, 5, 6, 7, 8, 9, 10, 11	1497, 1718, 1979	10	1	3	
**19**	CP100583.2:704016-704961	945	96.40%	1, 2, 3, 4, 5, 6, 7, 9, 10, 11	871, 907, 1129, 1211, 2130	4	6	5	
**20**	CP100583.2:705078-706033	955	93.51%	1, 2, 3, 4, 5, 6, 7, 8, 9, 10, 11	682, 778, 904, 991	6	5	4	
**21**	CP100583.2:706296-707458	1162	80.98%	1, 2, 3, 4, 5, 6, 7, 8, 9, 10, 11	1047, 1138	9	2	2	
**22**	CP100583.2:708053-709986	1933	90.12%	1, 2, 3, 4, 6, 7, 8, 9, 10, 11	1106, 1442, 1607, 1785, 2082	7	3	5	
**23**	CP100583.2:710160-711543	1383	88.86%	1, 2, 3, 4, 5, 6, 7, 8, 9, 10, 11	808, 854, 1145, 1331	5	6	4	
**24**	CP100583.2:711705-714191	2486	96.98%	1, 2, 3, 4, 5, 6, 8, 9, 10, 11	1976, 2375, 2585, 4353	4	6	4	
**25**	CP100583.2:714535-715342	807	87.61%	1, 2, 4, 7, 8, 9	518, 573, 696, 889	3	3	4	
**26**	CP100583.2:715465-716479	1014	90.93%	1, 2, 3, 4, 5, 7, 8, 9, 10	560, 592, 890, 1002, 1185	1	8	5	
**27**	CP100583.2:716635-717637	1002	90.52%	2, 3, 4, 5, 6, 7, 8, 9, 10, 11	578, 650, 704, 844, 918	1	9	5	
**28**	CP100583.2:717826-719130	1304	24.08%		NaN	NaN	NaN	NaN	
**29**	CP100583.2:719153-720726	1573	88.11%		NaN	NaN	NaN	NaN	
**30**	CP100583.2:720972-723219	2247	73.88%	1, 2, 3, 4, 5, 8, 9, 10, 11	1626, 2027, 2304, 3164	4	5	4	
**31**	CP100583.2:723318-724684	1366	77.67%	1, 2, 3, 4, 5, 7, 8, 9, 10	908, 1010, 1167, 1262	5	4	4	
**32**	CP100583.2:724824-726715	1891	74.93%	1, 2, 3, 4, 5, 6, 7, 8, 9, 10, 11	1332, 1546, 1869, 1962, 2294, 2646, 2981, 3724	4	7	8	

**33**	CP100583.2:726843-728632	1789	91.73%	1, 2, 3, 4, 5, 6, 7, 8, 9, 10, 11	1363, 1614, 1705	10	1	3	
**34**	CP100583.2:728805-731414	2609	88.62%	1, 2, 3, 4, 5, 6, 7, 9, 10, 11	1668, 1954, 2240, 2515, 2842, 3139	4	6	6	
**35**	CP100583.2:731575-732967	1392	95.83%	1, 2, 3, 4, 5, 7, 8, 9, 10, 11	747, 1071, 1290, 1379	6	4	4	
**36**	CP100583.2:733168-733613	445	15.96%	1, 2, 3, 4, 5, 7, 8, 9, 10	395, 417	9	0	2	
**37**	CP100583.2:733765-734597	832	77.88%	1, 2, 3, 4, 5, 6, 7, 8, 9, 10, 11	641, 825, 1025, 1192, 1247, 1569, 1782, 2227	2	9	8	
**38**	CP100583.2:734716-735541	825	86.18%	1, 2, 3, 4, 5, 6, 7, 8, 9, 10, 11	798, 857	11	0	2	
**39**	CP100583.2:735804-737282	1478	70.84%	1, 2, 3, 4, 5, 6, 7, 8, 9, 10, 11	1079, 1277, 1409, 1580	6	5	4	
**40**	CP100583.2:737419-738898	1479	95.61%	1, 2, 3, 4, 5, 6, 7, 8, 9, 10, 11	880, 1040, 1215, 1435, 1688, 1936	3	8	6	
**41**	CP100583.2:739013-742837	3824	67.94%	1, 2, 3, 4, 5, 6, 7, 8, 9, 10, 11	2805, 3491, 3812, 4097	6	5	4	
**42**	CP100583.2:742969-743845	876	67.69%	1, 2, 3, 4, 5, 6, 7, 8, 9, 10, 11	766, 849	6	5	2	
**43**	CP100583.2:744021-745117	1096	79.20%	1, 2, 3, 4, 5, 6, 8, 9, 10, 11	952, 1038	8	2	2	
**44**	CP100583.2:745334-745975	641	61.78%	1, 2, 3, 4, 5, 6, 7, 8, 9, 10, 11	554, 611, 678, 729	3	8	4	
**45**	CP100583.2:746218-747038	820	63.29%	1, 2, 3, 4, 7, 10, 11	759, 948, 1142	1	6	3	

* SRX11722867[1], SRX11722868[2], SRX11722871[3], SRX14125033[4], SRX14125034[5], SRX19311940[6], SRX19311957[7], SRX19311958[8], SRX19311959[9], SRX19311960[10], SRX19311961[11]

Gene LFNB located on Gallus gallus breed Huxu chromosome 12 (normal chromosome)

**Results from genome assembly GGswu**				**Results from public Oxford Nanopore sequencing data**					
**Intron name**	**Position**	**Intron length [bp]**	**Coverage by simple repeats**	**Samples analyzed (after filtering)**	**Intron lengths [bp]**	**# samples with single length allele**	**# samples with different length allels**	**# intron length alleles**	**Source of intron length variation**
**1**	CP100566.1:9504893-9527472	22579	0.40%	1, 2, 3, 4, 5, 6, 7, 8, 9, 10, 11	21943	11	0	1	
**2**	CP100566.1:9527720-9528671	951	0.00%	1, 2, 3, 4, 5, 6, 7, 8, 10, 11	930	10	0	1	
**3**	CP100566.1:9528768-9529836	1068	0.00%	1, 2, 3, 4, 5, 7, 8, 9, 10, 11	1052	10	0	1	
**4**	CP100566.1:9529983-9532473	2490	0.00%	1, 2, 3, 4, 5, 6, 8, 9, 10, 11	2446	10	0	1	
**5**	CP100566.1:9532591-9533307	716	3.77%	1, 2, 3, 4, 5, 6, 7, 8, 9, 10, 11	567, 701	10	1	2	single insertion
**6**	CP100566.1:9533384-9534016	632	0.00%	1, 2, 3, 4, 5, 6, 7, 8, 9, 10, 11	542, 621	10	1	2	diffuse variation
**7**	CP100566.1:9534178-9535423	1245	0.00%	1, 2, 3, 4, 5, 6, 7, 8, 9, 10, 11	1224	11	0	1	
**8**	CP100566.1:9535620-9537276	1656	0.00%	1, 2, 3, 4, 5, 6, 7, 8, 9, 10, 11	1633	11	0	1	
**9**	CP100566.1:9537413-9538476	1063	0.00%	1, 2, 3, 4, 5, 6, 7, 8, 9, 10, 11	1026	11	0	1	
**10**	CP100566.1:9538602-9539077	475	0.00%	1, 2, 3, 4, 5, 6, 7, 8, 9, 10, 11	464	11	0	1	
**11**	CP100566.1:9539213-9539506	293	0.00%	1, 2, 3, 4, 6, 7, 10	286	7	0	1	
**12**	CP100566.1:9539699-9540048	349	0.00%	1, 2, 3, 4, 5, 6, 7, 8, 9, 10, 11	344	11	0	1	
**13**	CP100566.1:9540161-9540694	533	6.38%	1, 2, 3, 4, 5, 6, 8, 9, 10, 11	524	10	0	1	
**14**	CP100566.1:9540837-9541096	259	0.00%	1, 2, 3, 4, 6, 8, 9, 10, 11	256	9	0	1	
**15**	CP100566.1:9541219-9541469	250	0.00%	1, 2, 3, 4, 5, 7, 8, 11	248	8	0	1	
**16**	CP100566.1:9541629-9542187	558	0.00%	1, 2, 3, 4, 5, 7, 8, 9, 10, 11	548	10	0	1	
**17**	CP100566.1:9542277-9542517	240	0.00%	1, 2, 3, 4, 5, 6, 8, 9, 10	237, 248	8	1	2	diffuse variation
**18**	CP100566.1:9542686-9544898	2212	0.00%	1, 2, 3, 4, 5, 7, 8, 9, 10, 11	2172	10	0	1	
**19**	CP100566.1:9545015-9545621	606	5.28%	1, 2, 3, 4, 5, 6, 8, 9, 10, 11	593	10	0	1	
**20**	CP100566.1:9545883-9546497	614	0.00%	1, 2, 3, 4, 5, 7, 8, 9, 10, 11	619	10	0	1	
**21**	CP100566.1:9547094-9547329	235	0.00%	1, 2, 3, 4, 5, 6, 7, 9, 10, 11	233	10	0	1	
**22**	CP100566.1:9547502-9547889	387	0.00%	1, 2, 4, 5, 6, 7, 8, 9, 10, 11	377	10	0	1	
**23**	CP100566.1:9548051-9548420	369	0.00%	1, 2, 3, 4, 5, 6, 9, 10, 11	365	9	0	1	
**24**	CP100566.1:9548580-9549578	998	0.00%	1, 2, 3, 4, 5, 6, 7, 8, 9, 10, 11	981	11	0	1	
**25**	CP100566.1:9549745-9550851	1106	1.63%	1, 2, 3, 4, 6, 7, 8, 9, 10, 11	1086	10	0	1	

**26**	CP100566.1:9550974-9551180	206	0.00%	1, 3, 4, 5, 6, 7, 8, 9, 11	204	9	0	1	
**27**	CP100566.1:9551336-9552574	1238	0.00%	1, 2, 3, 4, 5, 6, 7, 8, 9, 10, 11	1215	11	0	1	
**28**	CP100566.1:9552763-9554452	1689	0.00%	1, 2, 3, 4, 5, 8, 9, 10, 11	1658	9	0	1	
**29**	CP100566.1:9554699-9555622	923	0.00%	1, 2, 3, 4, 5, 6, 7, 8, 9, 10, 11	899	11	0	1	
**30**	CP100566.1:9555696-9556423	727	0.00%	1, 2, 3, 4, 5, 6, 7, 8, 9, 10, 11	713	11	0	1	
**31**	CP100566.1:9556525-9557372	847	0.00%	1, 2, 3, 4, 5, 6, 7, 8, 9, 10, 11	832	11	0	1	
**32**	CP100566.1:9557512-9559034	1522	0.00%	1, 2, 3, 4, 5, 6, 7, 8, 9, 10, 11	1490	11	0	1	
**33**	CP100566.1:9559162-9559675	513	0.00%	1, 2, 3, 4, 5, 6, 7, 8, 9, 10, 11	505	11	0	1	
**34**	CP100566.1:9559848-9560002	154	0.00%	1, 2, 3, 4, 8, 9, 10	153, 170	6	1	2	diffuse variation
**35**	CP100566.1:9560160-9560510	350	0.00%	1, 2, 4, 5, 6, 7, 8, 10, 11	341	9	0	1	
**36**	CP100566.1:9560713-9561277	564	0.00%	1, 2, 3, 4, 5, 6, 7, 8, 9, 10, 11	559	11	0	1	
**37**	CP100566.1:9561429-9561746	317	0.00%	1, 2, 3, 4, 5, 6, 7, 8, 9, 10	313	10	0	1	
**38**	CP100566.1:9561868-9562405	537	0.00%	1, 2, 3, 4, 5, 7, 8, 9, 10, 11	530	10	0	1	
**39**	CP100566.1:9562671-9563023	352	0.00%	1, 2, 3, 4, 5, 6, 7, 8, 9, 10	342	10	0	1	
**40**	CP100566.1:9563160-9563251	91	0.00%	1, 4, 6, 8	90, 99	2	2	2	diffuse variation
**41**	CP100566.1:9563366-9565151	1785	0.00%	1, 2, 3, 4, 5, 6, 7, 8, 9, 10, 11	1752	11	0	1	
**42**	CP100566.1:9565283-9566322	1039	0.00%	1, 2, 3, 4, 5, 6, 7, 8, 9, 10, 11	1020	11	0	1	
**43**	CP100566.1:9566498-9568707	2209	0.00%	1, 2, 3, 4, 5, 6, 7, 8, 10, 11	2150	10	0	1	
**44**	CP100566.1:9568925-9569811	886	0.00%	1, 2, 3, 4, 5, 6, 7, 8, 9, 10, 11	865	11	0	1	
**45**	CP100566.1:9570014-9570305	291	0.00%	1, 2, 3, 4, 5, 7, 10, 11	294	8	0	1	

* SRX11722867[1], SRX11722868[2], SRX11722871[3], SRX14125033[4], SRX14125034[5], SRX19311940[6], SRX19311957[7], SRX19311958[8], SRX19311959[9], SRX19311960[10], SRX19311961[11]
